# Two different types of tandem sequences mediate the overexpression of *TinCYP51B* in azole-resistant *Trichophyton indotineae*

**DOI:** 10.1128/aac.00933-23

**Published:** 2023-10-12

**Authors:** Tsuyoshi Yamada, Mari Maeda, Hiroaki Nagai, Karine Salamin, Yun-Tsan Chang, Emmanuella Guenova, Marc Feuermann, Michel Monod

**Affiliations:** 1 Teikyo University Institute of Medical Mycology, Tokyo, Japan; 2 Asia International Institute of Infectious Disease Control, Teikyo University, Tokyo, Japan; 3 Nihon Nohyaku Co., Ltd., Tokyo, Japan; 4 Department of Dermatology, Centre Hospitalier Universitaire Vaudois, Lausanne, Switzerland; 5 Faculty of Biology and Medicine, University of Lausanne, Lausanne, Switzerland; 6 Swiss-Prot group, SIB Swiss Institute of Bioinformatics, Geneva, Switzerland; University Children's Hospital Münster, Münster, Germany

**Keywords:** dermatophytes, *Trichophyton indotineae*, *TinCYP51B*, gene duplication, itraconazole, voriconazole, antifungal resistance

## Abstract

*Trichophyton indotineae* is an emerging dermatophyte that causes severe tinea corporis and tinea cruris. Numerous cases of terbinafine- and azole-recalcitrant *T. indotineae*-related dermatophytosis have been observed in India over the past decade, and cases are now being recorded worldwide. Whole genome sequencing of three azole-resistant strains revealed a variable number of repeats of a 2,404 base pair (bp) sequence encoding *TinCYP51B* in tandem specifically at the *CYP51B* locus position. However, many other resistant strains (itraconazole MIC ≥0.25 µg/mL; voriconazole MIC ≥0.25 µg/mL) did not contain such duplications. Whole-genome sequencing of three of these strains revealed a variable number of 7,374 bp tandem repeat blocks harboring *TinCYP51B*. Consequently, two types of *T. indotineae* azole-resistant strains were found to host *TinCYP51B* in tandem sequences (type I with 2,404 bp *TinCYP51B* blocks and type II with 7,374 bp *TinCYP51B* blocks). Using the CRISPR/Cas9 genome-editing tool, the copy number of *TinCYP51B* within the genome of types I and II strains was brought back to a single copy. The azole susceptibility of these modified strains was similar to that of strains without *TinCYP51B* duplication, showing that azole resistance in *T. indotineae* strains is mediated by one of two types of *TinCYP51B* amplification. Type II strains were prevalent among 32 resistant strains analyzed using a rapid and reliable PCR test.

## INTRODUCTION

The spread of antifungal resistance in dermatophytes is an emerging health problem worldwide. The growing incidence of terbinafine-resistant strains of several species of *Trichophyton* has been the subject of multiple reports from many countries (1 – 5). So far, in all cases, terbinafine resistance results from missense mutations in the squalene epoxidase (*SQLE*) gene. Given the limited number of effective antifungals currently available to treat dermatophytes, the fact that antifungal resistance is not only related to terbinafine but also to azole compounds is a major concern. Azole resistance associated with the overexpression of genes encoding multidrug transporters of the ABC family was first reported in individual cases of *Trichophyton rubrum* (6 – 8). Notwithstanding, azole resistance appears to be more common, especially in the emerging species *Trichophyton indotineae* (formerly called *Trichophyton interdigitale* or *Trichophyton mentagrophytes* type VIII) (4, 9 – 11). About 25% of *T. indotineae* isolated from skin dermatophytosis lesions in India were found to be less susceptible to itraconazole (ITC) and voriconazole (VRC) (10).

We recently found that the reduced susceptibility of four *T. indotineae* strains to azoles was mainly due to the overexpression of the *CYP51B* gene (*TinCYP51B*) encoding a sterol 14α-demethylase, a target of azole compounds such as ITC and VRC (12). The overexpression of *TinCYP51B* resulted from additional copies of this gene in tandem, where 100% identical 2,404 base pair (bp) block repeats harboring *TinCYP51B* were inserted end-to-end (12). Each block included 631 bp of the original promoter and almost the entire coding region of the sterol 14α-demethylase—only the last five codons were missing. Overexpression of the gene encoding the drug target is an effective mechanism to acquire resistance by counteracting the effects of the drug, altering the balance in favor of its target. Sterol 14α-demethylases play a crucial role in ergosterol biosynthesis in fungi, by catalyzing the oxidative removal of 14α-methyl groups from sterol precursors (13, 14)

The present study aimed to examine whether all *T. indotineae* strains less sensitive to azoles had the same resistance mechanism as that recently described (12). Examination of a large panel of strains deemed resistant revealed without exception overexpression of *TinCYP51B*, but many did not contain tandem duplications of the 2,404 bp sequence containing *TinCYP51B*. We found that resistance in these strains was due to extra copies of *TinCYP51B* by large segmental duplication of a 7,374 bp DNA fragment. Thus, two different types of tandem sequences (i.e., single-gene duplications and large segmental duplications) mediate the overexpression of *TinCYP51B* in *T. indotineae*.

## RESULTS

### Two different types of azole-resistant strains of *T. indotineae* with multiple copies of *TinCYP51B*

Twenty-nine *T. indotineae* strains deemed resistant to ITC and VRC (ITC MIC ≥ 0.25 µg/mL; VRC MIC ≥ 0.25 µg/mL; Table S1) were tested to examine whether resistance to azole in *T. indotineae* was always mediated by tandem duplications of 2,404 bp chromosome blocks harboring the *TinCYP51B* gene. PCR was performed using the P1-sense and P2-antisense primers, designed at the 3′ and 5′ ends of *TinCYP51B*, respectively, and genomic DNA as a target. A 956 bp DNA fragment was only to be amplified with these primers when the 2,404 bp tandem duplicates were present. The three previously characterized resistant strains TIMM200116, TIMM200118, and TIMM 200119 (Table 1; Table S1), which are known to harbor 5–7 alleles of *TinCYP51B* in as many 2,404 bp blocks in tandem (12), were used as positive controls. Two previously characterized susceptible strains, TIMM200114 and TIMM 200115 (Table 1; Table S1), harboring one *TinCYP51B* gene (12) were used as negative controls.

**TABLE 1:** Phenotypic and genotypic characteristics of *T. indotineae* strains used in this study

*T. indotineae* strains^*a*^	TBF MIC_80_ (μg/mL)^*d*^	ITC MIC_80_ (μg/mL)^*d*^	VRC MIC_80_ (μg/mL)^*d*^	Fold expression of *TinCYP51B* (mean ± SD)^*b*^	Number of *TinCYP51B* alleles (mean ± SD)^*c*^	Strain type
TIMM20114	0.125	0.06	0.015	1	1	Azole susceptible
(**UKJ1676/17**; IFM 67092)						
TIMM20115	8	0.06	0.03	1.0 ± 0.4	0.9 ± 0.2	Azole susceptible
(**UKJ1700/17II**; IFM67093)						
TIMM20116	0.125	0.5	0.5	34.0 ± 5.3	5.3 ± 0.8	Type I
(**UKJ1708/17**; IFM 67094)						
TIMM20118	8	0.5	1	29.8 ± 4.4	5.5 ± 0.6	Type I
(**UKJ1687/17**; IFM 67096)						
TIMM20119	8	1	1	89.9 ± 26.2	8.9 ± 0.6	Type I
(**200123/18**; IFM 67097)						
TIMM20117	0.125	0.5	0.5	9.9 ± 3.8	10.8 ± 0.6	Type II
(**200087/18**; IFM 67095)						
TIMM20120	0.125	0.25	1	12.5 ± 5.0	10.0 ± 1.2	Type II
(**250082/18**)						
TIMM20121	8	0.5	1	23.4 ± 11.6	14.0 ± 0.05	Type II
(**250084/18**)						
TIMM20122	8	0.5	0.5	22.0 ± 8.0	7.9 ± 0.3	Type II
(**250108/18**)						
TIMM20123	0.125	1	0.5	6.2 ± 2.5	6.4 ± 0.4	Type II
(**600097/19**)						

^*a*^ All strains were from a previously published resistance study in India, with the numbering in bold (10). They were then preserved in the culture collection of Teikyo University Institute of Medical Mycology (TIMM), some of which were also preserved in the culture collection of Medical Mycology Research Center, Chiba University (IFM), through the National Bio-Resource Project, Japan (http://www.nbrp.jp/).

^*b*^ The results represent expression levels from three independent real-time PCR experiments. The expression levels of *TinCYP51B* genes were indicated as relative fold changes compared to the ΔCt mean of the data from TIMM20114 (control with a single copy of *TinCYP51B*). SD, standard deviation.

^*c*^ Data were obtained from three independent qPCR experiments using genomic DNA as a target. The *TinCYP51B* gene copy number of each strain was indicated as relative fold change compared to the ΔCt mean of the data from TIMM20114.

^*d*^ TBF, terbinafine; ITC, itraconazole; VRC, voriconazole.

**Fig 1:**
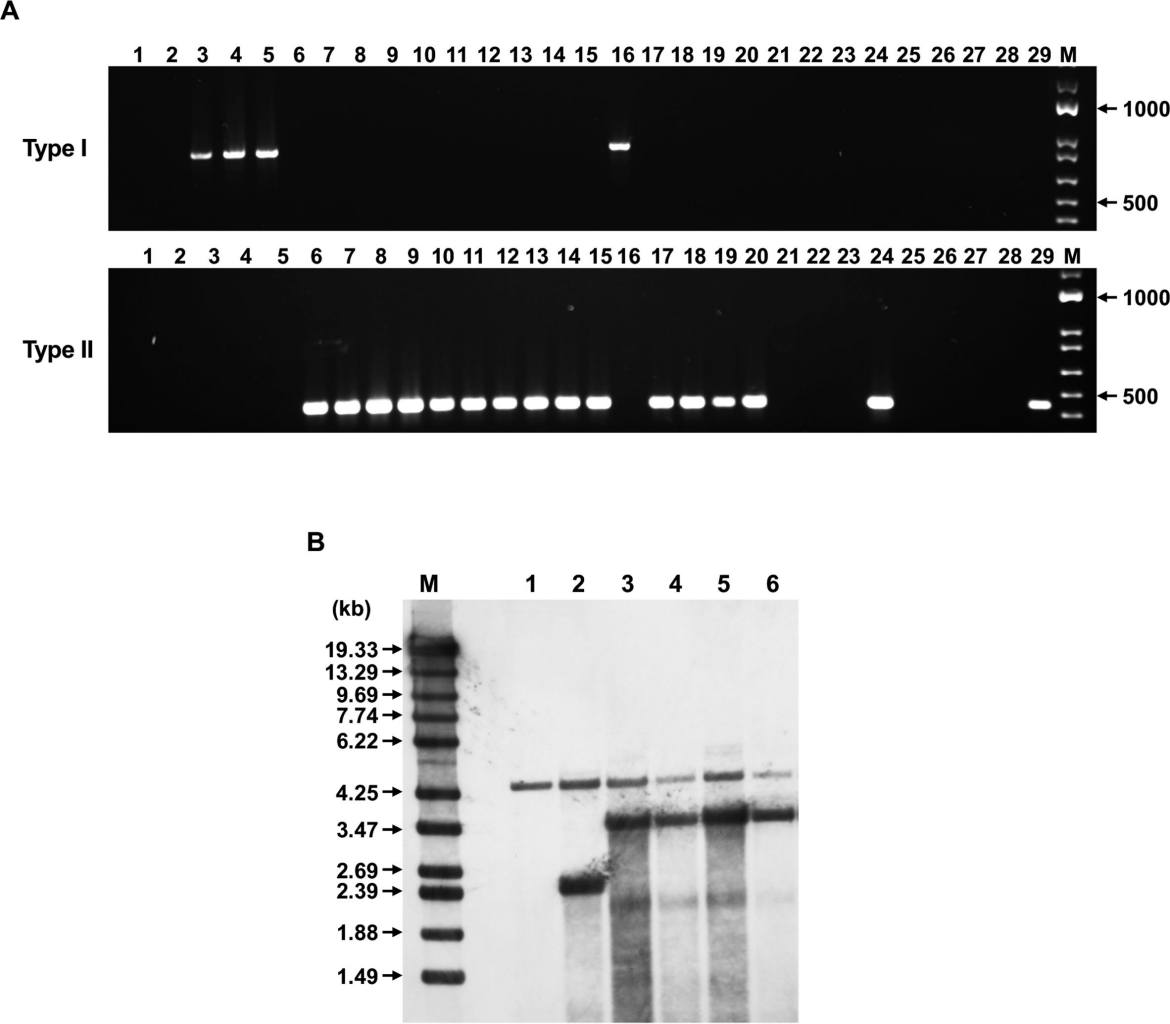
Identification of types I and II strains in *T. indotineae*. (**A**) Types I and II strains were identified by amplification of genomic DNA with primer pairs P1–P2 and P3–P4, respectively. TIMM200114 and TIMM 200115, which are susceptible, were used as negative controls (lanes 1 and 2). Azole-resistant strains TIMM200116, TIMM200118, and TIMM 200119, which are known to harbor 5–7 alleles of CYP51B in as many 2,404 bp blocks in tandem, were used as positive controls for the type I strains (lanes 3–5). Lanes 6–29: Search for types I and II tandem sequences in the *T. indotineae* strains in Table S1. Lanes 6–20: Strains deemed to be resistant to azoles (ITC MIC ≥ 0.25 µg/mL; VRC MIC ≥ 0.25 µg/mL) marked by an asterisk (*) in Table S1. Strain 216510/17 was type I (lane 16). All other strains were type II. Lanes 21–29: Strains deemed to be susceptible to azoles (ITC MIC ≤ 0.25 µg/mL; VRC MIC ≤ 0.25 µg/mL) marked with a hash (#) in Table S1. Two of the later strains (UKJ 1673/17 and 250063/18) were of type II (lanes 24 and 29). (**B**) Southern blotting analysis of genomic DNA samples from six *T. indotineae* strains (TIMM20114, TIMM20118, TIMM20120, TIMM20121, TIMM20122, and TIMM20123). Genomic DNA from each strain was digested with XhoI and separated by electrophoresis on a 0.8% (w/v) agarose gel. Lanes 1–6: genomic DNA samples from TIMM20114, TIMM20118, TIMM20120, TIMM20121, TIMM20122, and TIMM20123, respectively. An internal fragment (about 410 bp) of the *TinCYP51B* gene was amplified by PCR with P21-P16 primers (Table S2) and used as a hybridization probe. The DNA standard fragment sizes are shown on the left.

As expected, no genomic DNA amplification was obtained with the azole-susceptible control strains TIMM20114 and TIMM20115 (Fig. 1A upper panel, lanes 1–2), and an amplicon of the predicted size was obtained with TIMM20116, TIMM20118, and TIMM20119 (Fig. 1A upper panel, lanes 3–5). However, we surprisingly detected DNA amplification specific for 2,404 bp tandem duplicates in only one out of all 29 new strains included in the analysis (strain 216510/17; Fig. 1A upper panel, lane 16). No amplicon was obtained from all strains deemed susceptible to azole compounds (ITC MIC ≤ 0.25 µg/mL; VRC MIC ≤ 0.25 µg/mL; Fig. 1A upper panel, lanes 21–29). To sum up, our results pointed out toward the existence of another yet unknown mechanism of azole resistance in *T. indotineae*. We therefore referred to all strains for which an amplicon was obtained as “type I” azole-resistant strains and provisionally coined the term “type II” azole-resistant strains for all newly discovered resistant strains.

Four type II strains, TIMM20120–TIMM20123, were chosen to further characterize the mechanism involved in azole resistance. Just as in type I strains, qRT-PCR revealed strong overexpression of *TinCYP51B* in these strains (Table 1). We examined whether the TIMM20120–TIMM20123 strains contained multiple copies of *TinCYP51B* in their genome by quantitative PCR (qPCR) using their genomic DNA as a target. The azole-susceptible strain TIMM20114, with a single copy of *TinCYP51B*, was used for comparison. The genomes of TIMM20120–TIMM20123 were estimated to contain 6 to 14 copies of *TinCYP51B* (Table 1).

The strains TIMM20120-TIMM 20123 differed from the susceptible strain TIMM20114 and the type I resistant strain TIMM20118 also in Southern blotting experiments, using a probe designed downstream of the XhoI site in the *TinCYP51B* open reading frame (ORF; Fig. 1B). As expected, a single 4.4 kb band was detected in the susceptible strain TIMM200114 with a single *TinCYP51B* gene (12). A band with the same electrophoretic mobility and a stronger 2.4 kb band were also revealed in the previously characterized type I resistant strain TIMM20118 (12), in agreement with its deposited genome sequence data (GenBank accession numbers: OK500344 and JAJVHJ000000000). The 4.4 kb band was also detected in the strains TIMM20120–TIMM20123, but there was an additional, more intense band corresponding to a 3.4 kb fragment and specific for type II strains.

### Genome sequencing of type II azole-resistant strains

**Fig 2:**
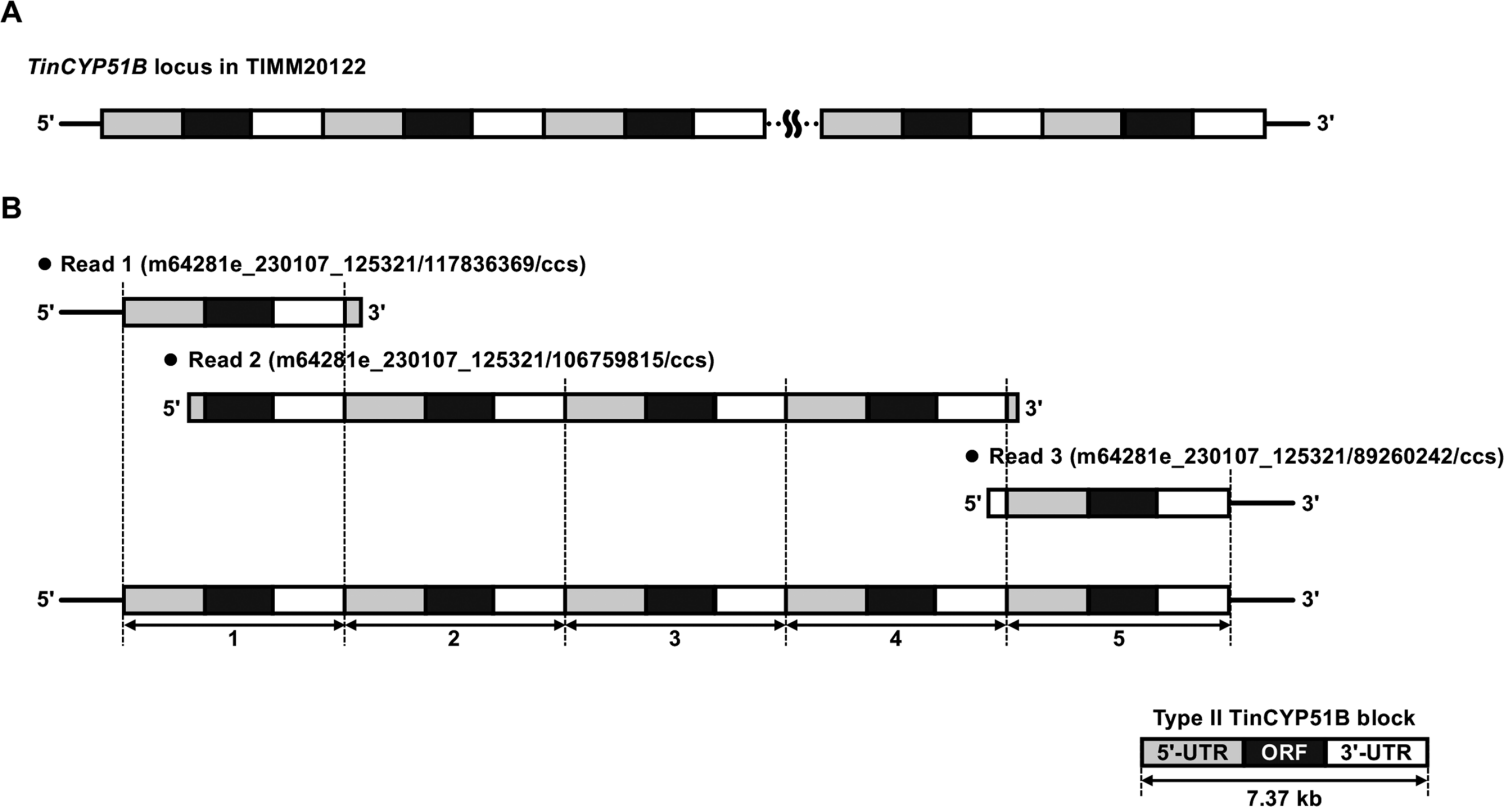
PacBio sequencing reveals direct tandem repeats of 7,374 bp blocks in type II strain TIMM20122. (**A**) Scheme describing the organization of the tandem blocks within TIMM20122. The black boxes correspond to the *TinCYP51B* open reading frame. Upstream and downstream sequences including neighboring genes are shown by gray and white boxes, respectively. (**B**) Examples of the reads obtained, including reads covering the 5′ border of the duplicated regions (read m64281e_230107_125321/117836369/ccs), reads covering the 3′ border of the duplicated region (read m64281e_230107_125321/106759815/ccs), and reads localized within the duplicated region demonstrating the presence of at least five repeats in this type II strain (read m64281e_230107_125321/89260242/ccs). Sequences of these three reads are given in the supplementary materials.

The whole genomes of the type II azole-resistant strains TIMM20121, TIMM20122, and TIMM20123 were sequenced using the PacBio sequencing technique (Table S3). Analysis of the sequences revealed that, similar to the type I resistant strains, the type II strains contained tandem duplications of the *CYP51B* locus. However, the duplicated blocks in the type II strains had a size of 7,374 bp instead of 2,404 bp. PacBio sequencing provided three types of reads with the *TinCYP51B* raw sequence data (Fig. 2): (i) reads with a DNA sequence identical to that upstream of the 7,374 bp block in TIMM20114, followed by one or two complete blocks, and ending with a partial block; (ii) reads with 1–3 complete blocks between the end and beginning of a partial block sequence; and (iii) reads starting with the end of a block, followed by 1–3 complete blocks, and ending with a DNA sequence identical to that of the unique 7,374 bp block sequence in TIMM20114. Sequences of the three read types for strains TIMM20121, TIMM20122, and TIMM20123 are given in the supplementary materials.

**Fig 3:**
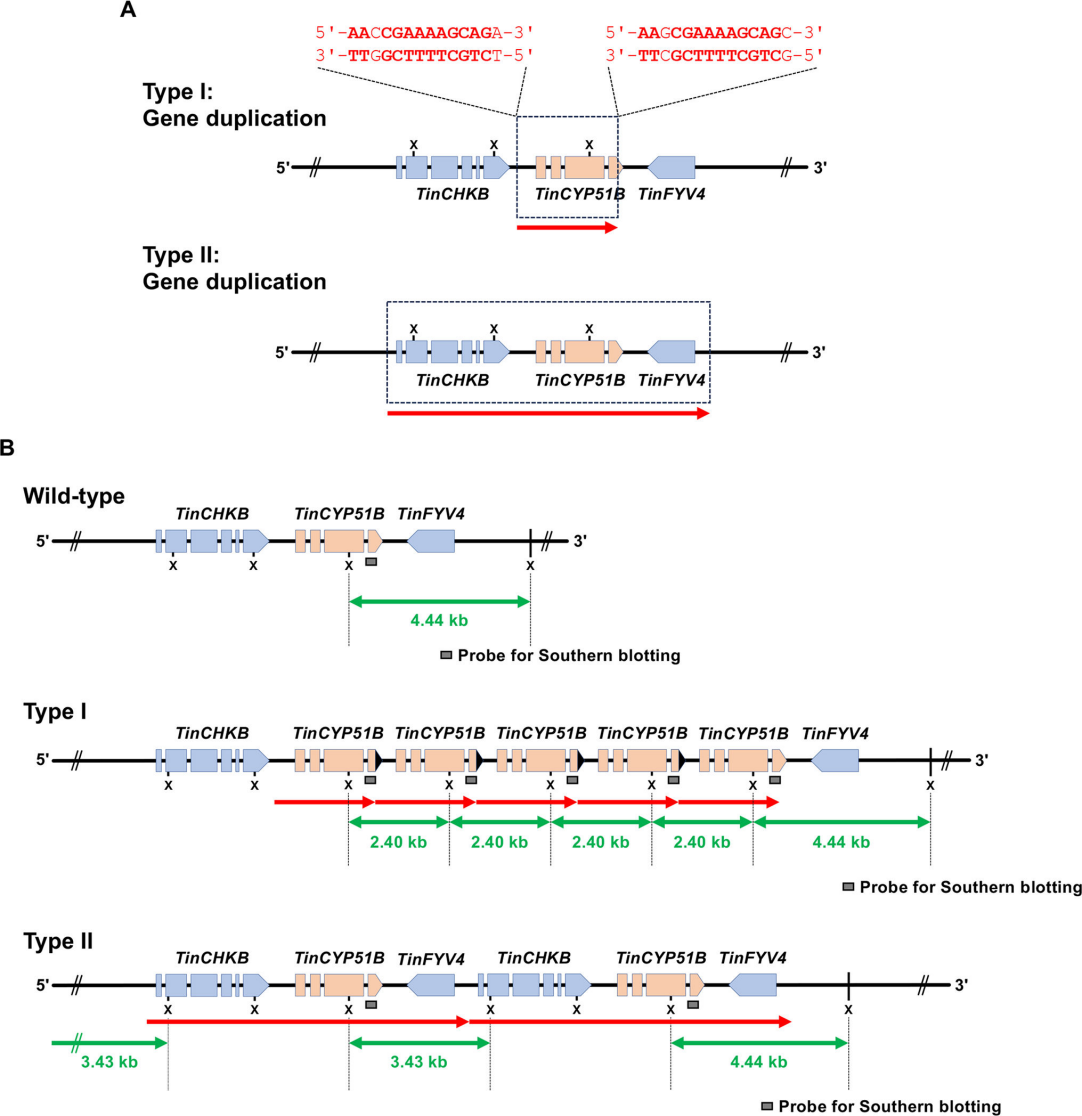
Comparison of the 2,404 bp duplicated blocks within type I strains and the 7,374 bp blocks found in type II strains. Type I strains include only the *TinCYP51B* gene (**A**). Type II strains contain *TinCYP51B* (skin-colored boxes) and the neighboring genes *TinCHKB* and *TinFYV4* (blue boxes) (**B**). The coverage of each type of block is marked with a red arrow and the sequences of the borders of the original block are also shown in red. The conserved nucleotides are marked in bold, revealing the absence of any conservation at the borders of the type II 7,374 bp duplicated blocks. The results of the PacBio sequencing results and southern blotting analysis are consistent. X, XhoI site.

Although the duplicated blocks in type II strains were centered on *TinCYP51B*, they also contained two adjacent genes, *TinCHKB*, encoding the homolog of *A. nidulans* chkB (AN4279) kinase (15, 16) (upstream), and *TinFYV4*, homologous to *Saccharomyces cerevisiae FYV4* (17) (downstream). Consequently, duplicated blocks in the type II strains were considered as large segmental duplications (Fig. 3A). Because of the large size of the duplicated blocks and the fact that there were more than five of them, the single PacBio reads could not cover the entire duplicated region. The 100% sequence identity between the blocks prevented efficient assembly of the individual reads to obtain the sequence covering an entire duplicated region (complete tandem repeat sequence) in the genomes. For strain TIMM20122, several reads covered three complete blocks of 7,374 bp, as well as parts of the two adjacent blocks, showing that this strain contains at least five blocks organized in tandem repeats. For strains TIMM20121 and TIMM20123, the PacBio sequencing revealed the presence of at least four blocks. The reads covered the 5′ and 3′ boundaries of the duplicated region for all three strains (see the supplementary materials). By combining the PacBio results with the qPCR results mentioned above, we concluded that 6 to 14 copies of the 7,374 bp block, organized in tandem repeats, were present within the genome of the type II strains TIMM20121, TIMM20122, and TIMM20123. Whatever the number of *TinCYP51B* alleles in the type II strains, the end-to-end 7,374 bp block sequences were consistent with the results obtained by Southern blotting analysis (Fig. 3B).

### Reduction of the *TinCYP51B* copy number to a single copy increases the susceptibility of *T. indotineae* to azole compounds

**Fig 4:**
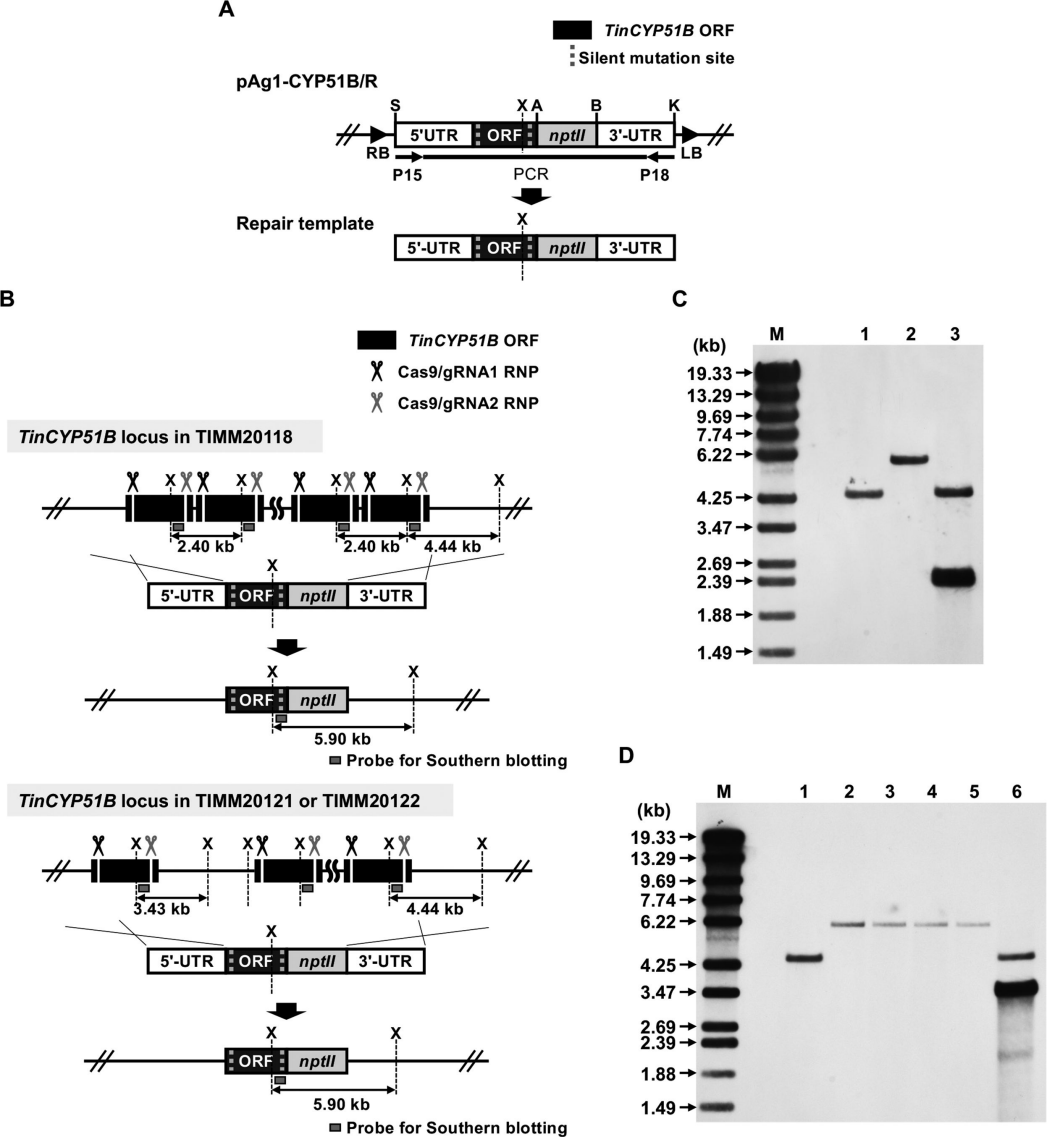
CRISPR/Cas9-mediated genetic modification of the *TinCYP51B* locus in the type I azole-resistant strain TIMM20118 and the type II azole-resistant strains TIMM20121 and TIMM20122. (**A**) Schematic representation of the binary vector pAg1-*TinCYP51B*/R. The *nptII* cassette is composed of the promoter sequence of *Aspergillus nidulans* tryptophan C (trpC) gene (*PtrpC*), *nptII*, and the termination sequence of *A. fumigatus cgrA* gene (*TcgrA*). The two silent mutations on the repair template cannot be cleaved by the RNP nuclease. Plasmid DNA of pAg1-*TinCYP51B*/R was used as a template for PCR with P15–P18 primers, to amplify the sequence (repair template) indicated by the thick line. (**B**) Schematic representation of the *TinCYP51B* locus before and after modification. The cleaved *TinCYP51B* locus is replaced by the repair template, resulting in the introduction of the desired modification. The replaced genomic DNA cannot be cleaved by the RNP nuclease. (**C AND D**) Southern blotting analysis. Aliquots of approximately 5 µg of genomic DNA from each strain were digested with XhoI and separated by electrophoresis on 0.8% (w/v) agarose gels. (**C**) Lane 1, TIMM20114 (azole susceptible); lane 2, 18 MM_25; lane 3, TIMM20118 (parent strain); M, DNA standard fragments (λ-EcoT14I/BglII digest). (**D**) Lane 1, TIMM20114 (azole susceptible); lanes 2 to 5, 21 MM_16–1, 21 MM_16–2, 21 MM_23–12, and 21 MM_23–13; lane 6, TIMM20121 (parent strain); M, DNA standard fragments. DNA standard fragment sizes are shown on the left. An internal fragment (about 410 bp) of the *TinCYP51B* gene was amplified by PCR with P21–P16 primers (Table S2) and used as a hybridization probe.

We previously showed the importance of *TinCYP51B* overexpression in the azole resistance of *T. indotineae* through RNA interference (RNAi)-mediated downregulation of the *TinCYP51B* gene (12). However, it was difficult to achieve complete inhibition of *TinCYP51B* gene expression in *TinCYP51B*-overexpressing strains with low azole susceptibility. Here, using the CRISPR/Cas9 genome editing tool, we attempted to reduce the copy number of the *TinCYP51B* gene within the genome of *TinCYP51B*-overexpressing strains to a single copy. For this purpose, two kinds of Cas9/single guide RNA (sgRNA) ribonucleoprotein complexes (RNPs) and the repair fragment were introduced into one type I azole-resistant strain, TIMM20118, and two type II azole-resistant strains, TIMM20121 and TIMM20122 (Fig. 4). The protoplasts/polyethylene glycol (PEG)-mediated transformation of these strains produced many G418-resistant transformants on the selective medium, 28 to 30 of which were chosen at random and tested for their growth properties on Sabouraud dextrose agar (SDA) supplemented with 2.0-µg/mL ITC.

**Fig 5:**
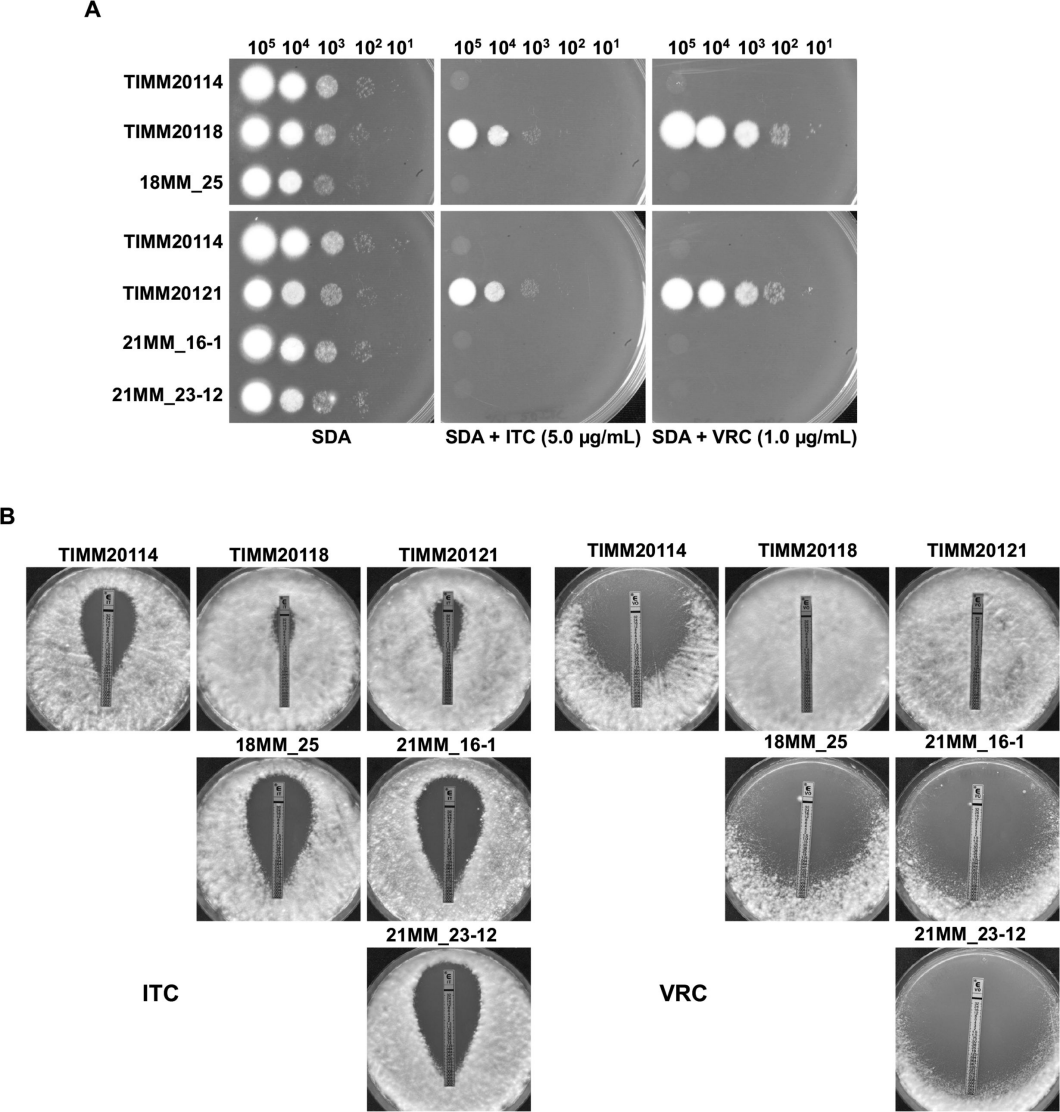
Evaluation of ITC and VRC susceptibility in the genetically modified *T. indotineae* strains by the serial dilution drug susceptibility assays and Etests. For the serial dilution drug susceptibility assays, spores from each strain were spotted at different dilutions on SDA plates, as previously described (4, 12). The plates were incubated at 28°C for 3 to 5 d.

The identification of the expected clones harboring a single copy of *TinCYP51B* was carried out in three steps. First, since overexpression of *TinCYP51B* confers resistance in *T. indotineae* (12), we selected the transformants showing the most significant growth inhibition on ITC, which were most likely to have a reduced copy number of *TinCYP51B* (14 clones for TIMM20118, 12 clones for TIMM20121, and 12 clones for TIMM20122). PCR with P22–P23 primers for TIMM20118 transformants and P24–P25 primers for TIMM20121 and TIMM20122 transformants then resulted in the exclusion of, respectively, 13 clones derived from TIMM20118 and eight clones derived from TIMM20121 which still carried several copies of the *TinCYP51B* gene in their genome. All clones derived from TIMM20122 still contained several copies of *TinCYP51B* and were not further investigated. Finally, Southern blot analysis enabled us to confirm that one clone derived from TIMM20118, and four clones derived from TIMM20121 harbored one single *TinCYP51B* gene, as expected (Fig. 4C and D). The ITC and VRC susceptibility of these clones, assessed by serial dilution drug susceptibility tests and Etests, was similar to that of the azole-susceptible strain TIMM20114 used for comparison (Fig. 5).

### Strains of *T. indotineae* with low susceptibility to azoles are mainly type II

The P3-sense and P4-antisense primers were designed 126 upstream and 309 bp downstream of the 7,374 bp block junctions in TIMM20120–TIMM20123 (Table S2). PCR reactions were performed using genomic DNA from these four strains as a target. As expected, a 435 bp amplicon was obtained for all four strains (Fig. 1A lower panel; Table 1). In total, 28 of the 32 strains with low susceptibility to azoles (MIC ITC ≥ 0.25 µg/mL; MIC VRC ≥ 0.25 µg/mL), which were not of type I, were of type II (Fig. 1A lower panel; Table S1). In conclusion, strains with low susceptibility to azoles were either types I or II, with a predominance of type II. Blocks of 7,374 bp in tandem could also be detected in *T. indotineae* strains that were more susceptible to ITC and VRC. Surprisingly, of the 16 strains deemed susceptible to azoles, three were found to be type II by PCR (Table S1). One of these strains, UKJ1673/17 underwent further examination and was found to contain three 7,374 bp blocks by qPCR using genomic DNA as a target (data not shown), whereas all the low susceptible type II strains contained at least five copies. These results show that strains appearing to be susceptible to azole compounds may harbor a reduced number of *TinCYP51B* in tandem.

## DISCUSSION

Examination of a large panel of *T. indotineae* azole-resistant strains revealed the existence of two types of strains (types I and II) containing repeats of several *TinCYP51B* in tandem. Type I strains harbored *TinCYP51B* duplications with 2,404 bp end-to-end blocks containing only this gene (12). Type II strains revealed large segmental duplications with blocks of 7,374 bp, which contained *TinCYP51B* as well as the adjacent upstream and downstream genes that we named *TinCHBK* and *TinFYV4*, respectively. Whole genome sequencing could not reveal the exact number of duplications in type II strains due to technological limitations, but this number could be estimated by qPCR analysis. Here, the newly described type II strains were by far more prevalent than type I strains. In our previous study, we selected the most resistant strains based on MICs and Etests to test ITC susceptibility (Fig. 5), which is why we first characterized type I strains (12).

The case of type II strain UKJ1673/17 is interesting as it was estimated to contain only three copies of the 7,374 blocks and did not show any significantly lower susceptibility than wild-type strains containing only a single *TinCYP51B* gene. Therefore, in evolutionary terms, it represents an interesting link between wild-type strains with high azole susceptibility and low susceptible type II strains. These strains will be the subject of a further study.

Using the CRISPR/Cas9 genome editing tool, the copy number of *TinCYP51B* within the genome of the types I and II strains was brought back to a single copy. The azole resistance of these modified strains was similar to that of susceptible strains without *TinCYP51B* duplications, demonstrating that the reduced susceptibility of the *T. indotineae* strains was caused by the amplification of the *TinCYP51B* gene (Fig. 5). The effect of amplifying adjacent genes remains to be evaluated. The *A. nidulans* chkB protein kinase plays a role in protecting against UV damage in this fungus (15). *S. cerevisiae* FYV4 is involved in telomere length regulation (17) and is required for survival upon exposure to K1 killer toxin (18). However, as shown in Fig. 5A, a reduction of the large segmental duplications containing *TinCHKB* and *TinFYV4* does not appear to have any significant effect on the cell growth of type II strains at least on SDA.

### Short conserved sequences are missing at the borders of the duplicated blocks in type II strains

Duplications are produced by homologous recombination (HR) between directly oriented repeat sequences, insertion sequences, or transposable elements, with the intervention of a recombinase enzyme. Such a mechanism might be the cause of the duplications observed in type I strains in which short conserved sequences have been found at the borders of the duplicated blocks, called HR sites HR1 and HR2 (12). No HR sequences, such as those found at the borders of the 2,404 bp blocks in type I strains, were identified at the borders of the 7,374 bp blocks in type II strains. Duplications that lack HR sequences at the duplication block junctions arise frequently in bacteria (19). This implies that spontaneous genetic duplications can occur *via* mechanisms that, in the absence of any repeated sequence, can use short homologous sequences or homology-independent random end joining (20). A similar process is responsible for the tandem duplications observed in the type II strains and also seems to occur frequently in *T. indotineae*.

Why *T. indotineae* favors the duplication mechanism for azole resistance, in contrast to other dermatophyte species, remains to be clarified. This bias may be simply the result of a lack of knowledge of gene duplications, which have only recently been discovered. There is no reason why such phenomena should not occur in other dermatophytes. A large-scale survey of dermatophyte azole-resistant strains might bring some answers to this question.

### Alteration in copy numbers of *CYP51* genes in fungi other than *T. indotineae*

The copy number variation (CNV) of *CYP51* genes is an effective way for fungi to acquire azole resistance (Table 2). The CNV can originate from whole chromosome duplications, as shown for chromosome 5 from *Candida albicans* (21, 22) and *Candida glabrata* (23), or for chromosome 1 from *Cryptococcus neoformans* (24), harboring drug target genes such as *ERG11*/*CYP51*. Large segmental duplications mediating the CNV of *CYP51* genes have been observed in fungi other than *T. indotineae*, such as *Rhynchosporium commune*, a major fungal pathogen of barley, and the recently emerged multidrug-resistant *Candida auris*, which is the focus of intensive studies. In *R. commune*, the large segmental duplication of *CYP51A* contributes to the emergence of azole resistance (25). In *C. auris*, several resistance mechanisms have been described, including aneuploidy, gene copy accumulation, and new mutations in known and unknown antifungal drug resistance genes (26 – 29). For example, the segmental duplication of chromosome 1 containing *ERG11*, the *CYP51* ortholog, and a whole chromosome 5 duplication, which contains *TAC1b* associated with an increased expression of *ERG11*, *TAC1b*, and *CDR2*, have been observed (26, 27). Gene duplications of *CDR1*, encoding a multidrug efflux transporter, or *ERG11* were further associated with fluconazole resistance (28, 29).

**TABLE 2:** Amplification of drug target genes in the literature

Species	References	Amplified genes	Drug	Mechanism	Detection technique
Fungi					
*Candida albicans*	(21, 22)	*ERG11/CYP51^a^*	Azoles	Chromosome 5 duplication (aneuploidy)	Comparative genome hybridization and karyotype analysis
*Candida glabrata*	(23)	*ERG11/CYP51*	Azoles	Chromosome 5 duplication (aneuploidy)	Hybridization experiments on chromosomal blots
*Candida auris*	(26, 27)	*ERG11/CYP51*	Azoles	Chromosome 5 duplication (aneuploidy)	Whole-genome sequencing (Illumina and MiSeq)
*Candida auris*	(28)	*ERG11/CYP51*	Azoles	Large segmental duplication^*h*^	Whole-genome sequencing (Illumina and MiSeq)
*Candida auris*	(29)	*ERG11/CYP51* and *CDR1^b^*	Azoles	Transient gene duplication	Real-time quantitative reverse-transcription PCR (qRT-PCR)
*Cryptococcus neoformans*	(24)	*ERG11/CYP51* and *AFR1^b^*	Azoles	Chromosome 1 duplication (aneuploidy)	Comparative genome hybridization and qRT-PCR
*Rhynchosporium commune*	(25)	*CYP51A*	Azoles	Large segmental duplication	Whole-genome sequencing (Illumina)
*T. indotineae*	(12)	*CYP51B*	Azoles	Gene duplication (tandem)	Whole-genome sequencing (PacBio) and qRT-PCR
*T. indotineae*	This study	*CYP51B*	Azoles	Large segmental duplication (tandem)	Whole-genome sequencing (PacBio) and qRT-PCR
Other species					
*Amaranthus tuberculatu*s	(30)	*EPSPS^c^*	Glyphosate	Gene duplication and chromosome duplication (aneuploidy)	Fluorescence *in situ* hybridization (FISH)
*Amaranthus palmeri*	(31 – 33)	*EPSPS*	Glyphosate	Transient gene duplication	qRT-PCR
*Kochia scoparia*	(34)	*EPSPS*	Glyphosate	Large segmental duplication	FISH
*Grasses*	(35 – 38)	*EPSPS*	Glyphosate	Transient gene duplication	qRT-PCR
*Anopheles stephensi*	(39)	*GSTe2* and *GSTe4^d^*	Dichloro-diphenyl-trichloroethane (DDT)	Large segmental duplication (tandem)	Whole-genome sequencing (Illumina) and PCR
*E. coli*	(40)	*ampC^e^*	Ampicillin	Gene duplication	β-lactamase activity and gel electrophoresis
*Streptococcus agalactiae*	(41, 42)	*folP^f^* and *dfrA^g^*	Sulphonamides and trimethoprim	Large segmental duplication (tandem)	qRT-PCR and DNA array data analysis

^*a*^ Sterol 14α-demethylase gene.

^*b*^ Pleiotropic ABC efflux transporter genes.

^*c*^ 5-enolpyruvylshikimate-3-phosphate synthase gene.

^*d*^ Glutathione S-transferase epsilon 2 and 4 genes.

^*e*^ β-lactamase gene.

^*f*^ Dihydropteroate synthase gene.

^*g*^ Dihydrofolate reductase gene.

^*h*^ Large segmental duplications contain several genes in contrast to gene duplications that contain only the target gene.

Duplications within the promoter region of the *CYP51* gene and missense mutations in the coding sequence (CDS) of this gene can also lead to overexpression and confer resistance. In *Aspergillus fumigatus*, the overexpression of *AfuCYP51A* is mediated by the presence of two copies of a 34 bp tandem sequence in the *AfuCYP51A* promoter and by the concomitant presence of an A for T substitution at position 364 in the CDS of the gene. This nucleotide change led to the mutation Leu98His in the protein (43). A similar case is found within *Blumeriella jaapii*, in which PCR analyses of the region upstream of *CYP51* in azole-resistant isolates indicated that DNA inserts ranging from 2.1 to 5.6 kb were present upstream of *CYP51* (44).

### Gene amplification, a mechanism of resistance not restricted to fungi

Amplification of the gene encoding the target molecule of a toxic chemical also occurs in plants, insects, and bacteria (Table 2). Fluorescence *in situ* hybridization (FISH) and qPCR have revealed that, as in the case of azole resistance in *T. indotineae*, the resistance of various plants against the herbicide glyphosate is mediated by amplification of the 5-enolpyruvylshikimate-3-phosphate synthase (EPSPS) gene encoding the target of this herbicide (Table 2) (30 – 38). Glyphosate-resistant *Amaranthus palmeri* plants contain from fivefold to more than 160-fold copies of the *EPSPS* gene within their genomes compared to susceptible plants (31 – 33). In glyphosate-resistant *Kochia scoparia* plants, from three to more than 10 extra *EPSPS* copies are present as a tandem gene duplication at one locus (31, 34).

More recently, amplification of the gene encoding the target molecule of a chemical substance has also been described in *Anopheles stephensi*, where tandem duplication of the 3.62 kb genomic region encoding the glutathione S-transferase epsilon 2 and 4 genes was shown to be involved in DDT resistance in a strain from India. The tandem duplicated region contained two functional paralogs of *GSTe2* and three functional paralogs of *GSTe4* (39).

Bacteria also use gene duplications and large segmental duplications to develop resistance (19). As early as 1977, *Escherichia coli* K-12 mutants overproducing beta-lactamase due to repetitions of the chromosomal beta-lactamase genes were demonstrated to show ampicillin resistance (40). More recently, large segmental duplications have been described in strains of *Streptococcus agalactiae* resistant to sulphonamides and trimethoprim (41, 42).

### Probable cause and direct identification of azole-resistant dermatophytosis by PCR

ITC is considered the main azole compound effective against dermatophytoses caused by *T. indotineae* (45). Fluconazole and VRC have also been used against this dermatophyte (46, 47). Other over-the-counter topical azoles, by targeting *TinCYP51B*, could predispose to the selection of strains with the same resistance characteristics. However, we found only two reports in PubMed on topical treatments of *T. indotineae* with miconazole (48, 49) under *T. mentagrophytes* type VIII and one report with topical clotrimazole in addition to pulsed ITC therapy (50). No reports with the use of other topical azole compounds such as econazole and oxiconazole were found. Thus, ITC, via repeated topical and systemic treatments, is probably the main contributor to the development of resistance in *T. indotineae*.

Resistance of *T. indotineae* resistant to azoles as well as to terbinafine should be monitored before starting treatment. The broth microdilution method (51) and the Etest assays are performed with *T. indotineae* in the same way as for yeasts and filamentous fungi. However, these tests are time-consuming to prepare and take 5–6 days to obtain MIC results after spore inoculation. An alternative way to test for azole resistance would be to carry out a direct search for tandem repeat sequences by standard PCR, using DNA extracted from cultures or directly from skin scales, as susceptibility to terbinafine can be determined by the detection of selected mutations using direct PCR and sequencing of *SQLE* gene amplicons (52).

## MATERIALS AND METHODS

### Strains and growth media

*T. indotineae* strains (*N* = 48), which had been previously characterized for susceptibility to terbinafine, ITC, and VRC, were used in this study (Table S1). The strains selected for the study of azole resistance mechanisms and the control strains are listed in Table 1. Glycerol 15% (v/v) stocks of these strains, stored at 80°C, were used for conventional culture on SDA and Sabouraud dextrose broth (Bio-Rad) at 28–30°C. Spore formation was promoted at 28°C using 1/10 SDA [0.1% (w/v) Bacto peptone (BD Bioscience) and 0.2% (w/v) dextrose (Fujifilm Wako Pure Chemical Corporation), 1.5% (w/v) Bacto agar] supplemented with 500-µg/mL cycloheximide and 50-µg/mL chloramphenicol (Fujifilm Wako Pure Chemical Corporation). *Escherichia coli* DH5α (Nippon Gene) was used for molecular cloning.

### Chemicals

ITC (Fujifilm Wako Pure Chemical Corporation) and VRC (Tokyo Chemical Industry) were dissolved in dimethyl sulfoxide (DMSO; Fujifilm Wako Pure Chemical Corporation) to constitute stock solutions (1.0 mg/mL for less soluble compounds). Stock solutions were stored at −20°C until use.

### Drug susceptibility assays

The MICs were determined using spore suspension stocks, according to the Clinical and Laboratory Standards Institute (CLSI) guidelines for the broth microdilution method (51), except for the use of SDA instead of RPMI1640 medium (4, 12). After incubation, the plates were visually evaluated and also read at 595 nm using a microtitration plate spectrophotometer (Multiskan Ascent, Thermo Fisher Scientific). The MIC_80_ was defined as the lowest concentration of the drug present in the wells showing a growth inhibition of ≥80% compared with the absorbance values obtained without antifungal agents.

The serial dilution drug susceptibility assays and Etest assays were performed as previously described (4, 12).

### Rapid PCR identification of azole-resistant types I and II strains of *T. indotineae*

For rapid strain typing of azole-resistant *T. indotineae* by PCR, genomic DNA was extracted from the growing mycelia using the DNeasy Plant Mini Kit (Qiagen), according to the manufacturer’s protocol. PCR was performed according to a standard protocol, using P1–P2 and P3–P4 sense and antisense primers (Table S2).

### Southern blotting analysis

Genomic DNA was extracted from the growing mycelia, according to a method described by Girardin et al. (53), with several minor modifications. Aliquots of 50–100 ng of the genomic DNA were used as templates for PCR. PCR was performed using PrimeSTAR HS or PrimeSTAR GXL DNA polymerases (Takara Bio). The nucleotide sequences of the primers used for PCR are listed in Table S2. For the Southern blotting analysis, aliquots of approximately 5 µg of the genomic DNA were digested with an appropriate restriction enzyme, separated by electrophoresis on 0.8% (w/v) agarose gels, and transferred onto Hybond-N^+^ membranes (Cytiva). Southern hybridization was performed using an ECL direct nucleic acid labeling and detection system (Cytiva), according to the manufacturer’s instructions.

### Real-time quantitative PCR

For the relative quantification of the *TinCYP51B* gene expression in *T. indotineae* strains by real-time quantitative reverse-transcription PCR (qRT-PCR), total RNA extraction from the growing mycelia and first-strand cDNA synthesis were performed, as described previously for *T. rubrum* (4). The synthesized cDNAs were treated with DNase I (Qiagen) before use.

For the relative determination of the *TinCYP51B* gene copy numbers in type II azole-resistant strains by real-time quantitative PCR (qPCR), genomic DNA extraction was performed from the growing mycelia using the method described by Girardin et al. (53).

The qRT-PCR, with cDNA as a target, and the qPCR, with genomic DNA as a target, were performed using Power SYBR Green PCR master mix on a StepOne real-time PCR system (Thermo Fisher Scientific) under standard conditions, according to the manufacturer’s recommendations. The 2^-ΔΔCt^ method was used to compare the ΔCt [threshold cycle (Ct) of the target gene (*TinCYP51B*) minus the Ct of the endogenous control gene (actin gene, *TinACT*)] values of the test strains (with unknown target gene copy numbers) with the ΔCt value of a calibrator strain (TIMM20114), which had a single copy target gene. The glyceraldehyde-3-phosphate dehydrogenase gene (*TinGAPDH*) was added as a target gene to confirm that there was virtually no difference in PCR reaction efficiency among the analyzed strains. The primers used to amplify *TinCYP51B*, *TinGAPDH*, and *TinACT* are listed in Table S2. Dissociation curves of the amplified products were plotted to confirm the absence of nonspecific products or primer dimers.

### Genome sequencing and assembly

The whole genome sequencing and data analysis of the *T. indotineae* strains were performed by Bioengineering Lab. Co., Ltd. (Japan). Genomic DNA was extracted from the growing mycelia according to the method described by Girardin et al. (53), with several minor modifications. After the elimination of short DNA fragments (<10 kb) using the Short Read Eliminator XS kit (Circulomics), the resulting DNA was sheared to 10–20 kb on the g-TUBE (Covaris) prior to library preparation. HiFi sequencing libraries were prepared using the SMRTbell Express Template Prep Kit 2.0 (Pacific Biosciences) and bound to the sequencing polymerase enzyme using the Sequel II Binding Kit 2.0 (Pacific Biosciences), according to the manufacturer’s protocol. Shotgun genomic DNA sequence data were collected on the Sequel IIe system (Pacific Biosciences) and assembled using the IPA HiFi Genome Assembler (version 1.8.0; Pacific Biosciences).

### Construction of repair templates

Repair templates harboring the *E. coli* neomycin phosphotransferase gene (*nptII*) cassette were generated using a binary vector pAg1-*TinCyp51B*/T (Table 3). The following two DNA fragments were generated from the genomic DNA of *T. indotineae* TIMM20118 by PCR: an approximately 4.32 kb fragment contains the 5-UTR and ORF of *TinCYP51B* using the P15-P16 primers, and an approximately 2.52 kb fragment contains the 3′-UTR of *TinCYP51B* using the P17–P18 primers. The silent mutations, which can’t be cleaved by the two RNP complexes (Cas9/gRNA1 RNP and Cas9/gRNA2 RNP), were introduced by overlap extension PCR and nested PCR with the primers listed in Table S2. The two obtained fragments were digested with SpeI/ApaI or BamHI/KpnI, respectively, and cloned into the corresponding restriction sites of pAg1-*TinCYP51B*/T, resulting in the generation of pAg1-*TinCYP51B*/R (Table 3; Fig. 4A).

**TABLE 3:** Plasmids used in this study

Plasmid	Description	References
pAg1	A streamlined version of the binary vector pBIN19 containing sequences necessary for replication in *E. coli* and *Agrobacterium tumefaciens* (*oriV* and *trfA*), *E. coli* neomycin phosphotransferase gene (*nptII*), and the transferable DNA (T-DNA) region, with a multiple cloning site within the T-DNA region	(54)
pAg1-*TinCyp51B*/T	The 5′ UTR of the *TinCYP51B* gene (2.52 kb; GenBank accession no. OK539858), the *nptII* cassette [the promoter sequence of *Aspergillus nidulans* tryptophan C (*trpC*) gene (*PtrpC*; GenBank accession no. X02390), *nptII*, the termination sequence of *Aspergillus fumigatus cgrA* gene (*TcgrA*; GenBank accession no. EAL84894)], the 3′ UTR of the *TinCYP51B* gene (2.51 kb)	(12)
pAg1-*TinCYP51B*/R	The 5′ UTR and ORF of the *TinCYP51B* gene (4.32 kb), the *nptII* cassette (*PtrpC*, *nptII*, *TcgrA*), the 3′ UTR of the *TinCYP51B* gene (2.51 kb)	This study

All primers used for the amplification of the specific DNA fragments from *T. indotineae* were designed based on the whole-genome sequence of *T. indotineae* TIMM20118 (12). The nucleotide sequences of the primers used are listed in Table S2. Where necessary, the amplified fragments were gel purified with a QIAEX II gel extraction kit (Qiagen), subcloned into HincII-digested pUC118, and sequenced.

### Ribonucleoprotein (RNP) complex formation

Two 23-nt nucleotide sequences specific to the target, which contain the protospacer adjacent motifs (PAMs; 5′-NGG-3′) near the translation initiation and termination codons of the *TinCYP51B* ORF [5′-GCAGAAACGAGAGACAATGTCGG-3′ (− strand); 5′-TTGGCGACCCAATGGTCTCGTGG-3′ (+ strand)], were manually chosen to synthesize two Alt-R crRNAs (Integrated DNA Technologies). Two guide RNAs (gRNA1 and gRNA2) were then prepared by mixing equimolar amounts of each Alt-R crRNA and Alt-R tracrRNA (Integrated DNA Technologies) in IDT Duplex Buffer (30-mM HEPES, pH 7.5, 100-mM potassium acetate; Integrated DNA Technologies), heating to 95°C, and slowly cooling to room temperature. Two RNP complexes (Cas9/gRNA1 RNP and Cas9/gRNA2 RNP) were assembled by combining the CRISPR-Cas nuclease (HiFi Cas9 Nuclease V3, Integrated DNA Technologies) and the gRNA at a 1:1 molar ratio of gRNA and protein and incubating at room temperature for 20 min.

### Fungal genetic transformation

The plasmid DNA of pAg1-*TinCYP51B*/R was used as a template for PCR, to amplify the sequence indicated in Fig. 4A. A pair of primers P15–P18 was used for PCR. The amplified DNA fragment was purified using the QIAquick PCR purification kit (Qiagen) and used as the repair template indicated in Fig. 4A. The repair template (2.5–5.0 µg) and two RNP complexes (55–57 nM) were then introduced into each host cell by the protoplast/PEG method, as described previously, with several minor modifications (55). After the PEG treatment, protoplasts were inoculated onto SDA supplemented with 1.2 M D-sorbitol and 1.0% (w/v) yeast extract. The plates were overlaid 24 h later with 10-mL SDA containing 250-µg/mL G418 (Fujifilm Wako Pure Chemical Corporation) and then incubated for 4–7 days. From the colonies regenerating on the selective plates, 28 to 30 clones were chosen at random and tested for their growth properties on SDA supplemented with 2.0-µg/mL ITC. Only clones showing a significantly reduced growth rate in the presence of ITC were selected and screened for their *TinCYP51B* loci by PCR and Southern blotting. Two pairs of the PCR primers P22–P23 and P24–P25 (Table S2) were designed to detect the type I (2,404 bp) and type II (7,374 bp) duplicated blocks within the genome of selected transformants, respectively.

## Data Availability

The whole-genome sequences of three *T. indotineae* strains (TIMM20121, TIMM20122, and TIMM20123) were deposited in GenBank with the following accession numbers: JAUJAB000000000, JAUJAA000000000, and JAUIZZ000000000, respectively. The details are shown in Table S3.

## SUPPLEMENTAL MATERIAL

NGS raw sequence reads aac.00933-23-s0001.pdf Nucleotide sequence of the TinCYP51B locus in TIMM20121.

NGS raw sequence reads aac.00933-23-s0002.pdf Nucleotide sequence of the TinCYP51B locus in TIMM20122.

NGS raw sequence reads aac.00933-23-s0003.pdf Nucleotide sequence of the TinCYP51B locus in TIMM20123.

Table S1 aac.00933-23-s0004.docx Phenotypic and genotypic characteristics of *T. indotineae* strains used in this study.

Table S2 aac.00933-23-s0005.docx Oligonucleotide primers used in this study.

Table S3 aac.00933-23-s0006.docx Overall genetic features of three *T. indotineae* strains.

ASM does not own the copyrights to Supplemental Material that may be linked to, or accessed through, an article. The authors have granted ASM a non-exclusive, world-wide license to publish the Supplemental Material files. Please contact the corresponding author directly for reuse.
