# Supplementary material for: Two different types of tandem sequences mediate the overexpression of TinCYP51B in azole-resistant Trichophyton indotineae
Source: Antimicrob Agents Chemother. 2023 Oct 12;67(11):e00933-23. doi: 10.1128/aac.00933-23 (PMC10648874; doi:10.1128/aac.00933-23)
Supplement: NGS raw sequence reads — Nucleotide sequence of the TinCYP51B locus in TIMM20121. [file aac.00933-23-s0001.pdf]

***TinCYP51B* locus in TIMM20121**

[Read No.m64281e_230311_112734/112723290/ccs] [Sense (+) strand]

~TAT (Upstream sequence of the 7374 block)

AAT~ACC (5’UTR)

ATG~TAG (*chkB* protein kinase genomic DNA) (Forward sequence)

ATG~TGA (*TinCYP51B* gene) (Forward sequence)

TAA~GCT (3’UTR)

CTA~CAT (*FYV4* genomic DNA) (Reverse sequence)

CAAACACATTCTACTTGCATATTTGGCTGGCGAGAGGCTCAAGTTGTGAAGAGAAATAATAGGTATTACGATG
CATCTTTGGTTAGATTTCAATCTTAAAAGGCCGGCCTAGCTAGTTAGTTAATTGGGAGTTTGATGGCCATGAC
TATAAGGACGCTCACTCCCGATTCCCTATGTCACCACACATCAGGGGGAAGATGAATAGAGGAAGAGATAGAG
GAAATAATCAGATAGAGAGACAGGGAAAGGGATAAAAAAAGAAAGACAGACGAAGGTTCGAACGCGCGGCAAA
AAGCGAAGCCGCAGCGCTGTCCACACGAATATTGTGTGGCCAATACGCTCCGGAAAGAACTTCTAAGTGGGTT
ATTTGGCCTCTTCGCTACTAGACTTCAGTGTGACAGCATACATAACGCCCCGAATGGCTCTCGGGGCTGCGGA
ATTGGTCGTCACAGGTGGGAACATGTGTGGGTACCGTTGACCAACTAGAGAGAGATGTGACTACTCAGTGATA
AGTGGTGGTAGTAATGACTCGCAGATGCGAATGCTATTAAATAAAATACTCTATATTAAATCAAATAAAGTCT
ATCAAGTTGCCCAAATGGCGGAAAGCTGAAGATATGAAACAAAGATGGTAGGACGGGAAATAGACTGCTTATG
TCAAGTGGCCTGTCAATCAATAGGGGATGTTGTTCGTCATGAGTTGGAACTGATCATAGCCCTTGGTCGACAT
GCTGGCGTAACTCTTCCTGATGAGTTCTCTCATATACTGCGCTGCCTCTTGTTCACTCTTCTCTAGGACAAAT
CGGTCGCGGAAGTTGGTCAGCGTATGGGGTGTGAAGCAAGGAAGACCGCTGTCCAGCATGAGGGTTACGATGT
TTGAAAGCTTCATAGTATAAGGTCTCGAAGCAAGGAAAGCTTTAACCACAAGTGATTCGAACCAACGGTAGGA
TTGAGAGTTGTTGTACGATGCCGAATGAGTGGTGGATGTAGTAGCTGTAGAAGCTGTGGCTGAGGCAGCAGTT
GACGAAGACGGATGGTATCCTCCCATGACAGCAATCATCTCCGTGGTCAGTTTGAACGGAGCACGCTCGAACC
GTACACCACCAGGCGCAATATCGAAACAGAATCCAAAGTCAATGTGGATGATATGTCCTGCGTCATCAATCAT
GATGTTGCCGTTATGTCGGTCTTTGAACTGCAGCAGGTATGAAATGACTGAATAAGCAGCCATACTCTTCACG
AAGTTGCTTCGGGCTTCTTGGAATTTGATCGACTGCTCTCCTCCGTACTTCGATACGAAGTAGTCGTACAGGC
CGTTGACAGCTTCACGGCCCAGCATGTCTCGAGATATCGAGTTGGGTAAGACATCGATCACGCCGCATCCAGG
GGCGGTGGCCACAACACGGTACGGGAAGACAAATACGTCTAATCCAACGCTCGCAAATATTTCGCGGAATGCA
GCAATCATTTGAAGTGCGAGGACATCTTGACGGCAGTCATCTCCGACCTTGAAGATAGCAGACTGCCATACCT
CATATGTCCCCGCTTGAGACGCAGCTAGGCTCTCGCGTTTGCTTTGTACGGCGGATTCCTTAACTTGGGACAG
CTGCAAATTCGCTTCCTCATGGCTAGCCTCTTCGGCAGGAGTAACGGCGTTGCTGCCGTCTTCAATATTCCTC
GTCTTCTTGATTCGGAACGTTGCCATATAAGGTGCCTTTGCGTGACTCTGCAAGGGCTTTCCGGACTTTCGAT
CGATGCCAACAACAACCCCATCTGGGTTACTTGGCAGGTACACGCCAACCTCAACCTTAATCTTTCGCAGCTC
CTCCGTAATCTTCTCCTTCTTCTCTTGTTTGGTGCGTTTAATGTATGGCCGTAGTTTGCCCGAAATACCGGTG
ATCTCGTTGAAGAAGGCGAATTCCCTCTCATAGAATTCTTTGTCTTCGTCCGTGAAGCTTGCAATAACAGAGT
CCATGAATCGGTCCAGCACCGGTTTAAGTGGATCAGGCTATTTTCTCGTTAGCATGACATTTGGATATCCATA
ACACTAATCAAGGTACTTACAATCTGAGAGTCTTCGTCCTTGTAGGCGTTTGCCTTCATATTCCAGATGACTT

GGTGGGCAAACATCTGTGACAGCTGAGCTGTATTTGAAAGGTAACGTTCTATATATCCCAGTGCATCATAGCG
AAGGGCCTGCACGAGCTGCGGAACATAGAAAAGGCCAACATCCACGGGGAAGCTCTCGAGAGATCGAAGGGCA
TATTGCAAGATGAACGGATGGTTACCATATGCTGGTAACAGATAAGTTACGGCTGAGATCGGATCGATCGGCT
CCCAATAGAGGAGGTACTGCATTGCCGTTAGGAGAGTGAAGACACACCTGGGGGTAAAACGCGAAACTTACCT
TGAGCTGGAATGAAACGTCCTCTGGAAGAGCTGACCCAAGTAGAATTTCCAAAGCATTTGGCTCCTTAAGTGC
TTTTTCCGGGAAGTTAAGTAGAAGCCATCGGATATCAGTTCGCAGTTTAGCAGATGGGAACCGTGATCCTAGC
TGGAGTGCTAGGCTTGGGTTTTCATTCCATGCCAATCGAAGAAGTCCCGCAACGGTCTCCTAAAGCATATAAT
TAGCCACTGGTTGGCCCGAAACAGTGATGTAAGGAACGTACCTCGGTGACGGTTTTGTTACCAGTTCCATAGA
AGAAGTACGATTTCTTTTCTAGGTCTAGTGGAGAAAGCCAGACTTTGAGTCGAGAGCGTTCATTTCCAATCAG
AATGAGGAGTAGATCGTGCTTTGCTTGTAGTGATTTATGAGACTTTGAGTCCACTACCTCTTTCCAAGACAAG
GTCTCCAACATAGATTCAATATCGTGGAGGACCTGATCTTCAGCTTTGAGCTGCAAGCGATTCCCTCCAAACG
ACCACCTAAAAAATGCTTGGTTAGCTATCTTCCTAGGAAGTGAGATTAAAATGATAACGTACCTTGGCGAGTG
GTTGAACCAGCTCAGTGCAGCAGAGAGAATTTGATCTTTGAGCTTCCACGAACCAACATTACTCTGTCCGGAG
AATGAATGGAGCACCTTGAGACTGAAAAGGATGATATGGAAGTGAATCTCGCGAGCTAGCGGGTGGCCAGTAG
AATCCCTCAAGGCCGCCAACGTCTCACTGACCAGGCGTCTAAATACCTGTTGCGTATTTAAGCTGCCTAGCCT
ACTTGCGCTGAAATGGCTTTCTAGGAACTGCAAAACCAAGAAATGAGGAGATATGATGTTTTGGGCTTTCTTA
TGACGCCGGACAATCGCTTCCTTATCGGATGGGGATGATTCAAGCTTGTTCATAAAGGGATTTTTATGTCTAA
AAAACGTTAGTTAGTCAGGGCAGGATGCAAGGAAACTGAGAGAAGATAGCTTACTTGAAGTTCGGATCGAAGA
TTCCTTGCTTGCGACGAATGGTACGTTCCCATGCCTGCGCTACTTCTACCAATATTCTGGATTCCATAGCTGG
TTTTTCATTGATAACTCCTAGCCACAGAGCAATTCCCATCCTGATGGACTCCTTGGTAAATATCTGGAAGGGA
ATGCTGACTAGGTAAGCAATAATTGCCGGATGGCTATCATCAGTATTACACAAGAGAGCAGCAGTGCGGCGGA
GAATATGCCGAGGAAGATCCAGCGAGATATGCTTCTCTGTTCTAATTTGATGCTCGAGAGCTGCGAGAGCTTC
TTGTACAGTGTCAGGGTAATCAGTCACCCGGGCGGTGGTGCTTTGACCTATTGAAGATATTAGAAGGTTGGTT
CAATGGCTCAAATCAGACATATAAATACCATAGGATATCGCTAGAGGAGACTTTCCTCTGCGAGCTGGGCGGC
TTCAGAGTACCGGTACTCTTGTCGAGTGGTATACTGAGAAATGAAGTCGGAAGCAACATTGAGAACAGTGCCA
CCTGGGTTATCAATTGATCCTATCAGCTGTTAATGAAAGCTCAGAGCATAAATTATTGATATAAGAACTGATA
TACCTAGTCGCTGGTCGGCAAAAGGGATTGCTGAGCCCATCTCAAGTGCTAACGAACGGCCCATGTTTATATT
GCCGTACACGCCATCATCATCGTAATCAGAAAGGTACGTCTATGAAAGAACATATATTAGTGAAGTTGATGGC
ATCGGTGGGAATAATAACTCACCTGCAGTAGCCCCTTGATATCTAATGGTGCGATGTTCATAACATTTGAGAT
CCAAGTGCGCGCTGAGTTATGGAGATTCTTCAAGGTACGTCTCCTGAAATCATAATTGTCTGATAAAACAACC
TGGACCGTCCGCCTGCTCGATGTGAAAGTAGAGTTCCATGCAAACTCATCCATTTCCTCGTCCATGCACGATG
ACCAGAGCAAAGTAATCAAATCAAGCAGAGTAAACAACGAGAGTTTCTGGCAAAGTGCTGACGGGCATTGCAT
GATTATCTTATTTGCAGCTAGAGTAGCAGTTTGTTGGACTTGGATGATACGATGGCAACAAGCAGTGAAAGTA
TCAGCTAGCTGTTTGGCAATATAAGGGGCCGAGAATTTCACGGATGTGCTTCGGAGAGTTTTCTCAAGGTAGA
CGGTCACAACCTTGTCCGCAATTGCTCTCATGCAATTGCCCATTTCCGAGGCACCCAAGGTTGGGTCGAGGAA
GTATGTGAACACTTTGGTGCAATCACCAGAATTGGCACGGAGGGTTTCTATGAGTAGCGTTGCATTGAGAAAG
AGCGCCTTGGGGTAGTTCAGGCGTCGGATATCATGTTCTTGTGCCGGCAGTTCAGCAGTCAGGTTCTTTTTCT
TTCAACGGTTGTCTGAGGGCTCTGGCCGCGTCTTAGAATTGTATTTAATTCAATGTCACTCTCGAGCAGTTCA

GCACGGTTCTCGGAGACTAGCGGAGGAGAATGTTCGGCGAGGATACGTAGTTGTTCGTAGTTCGCCTTTCCAA
GCTCTGAGTGAAGTGATATATCATGGGCTGCGATGTTGAACCACGCATCCCGGAGCATATCTGTGATATCATC
ATCGTCACACACCCCAGAGGCTAATTGATCTAAGCGGCCTGCCCGGGCTAGTAGTATAGCAAGAGGTTTCAAT
ATTGGGGCAATTTGTTTAGCAGTTGCCTCAAGATCCTCTGGATGAACCTTTTCGCGTTCGACCGCAGCTCCCT
GGTTAACTAAGCTCTCGAGGAGGAACACAAGATAAATCTTGAAGACTTCACTGTTTCGGTCGAGGTTGGCTGC
AAGGTAATTTCTTGCTTTGTGTATCTATGTATACGTTAGAATTCCCACTGACAAGGTCGAACAGCTAGATGAA
AGGGGGGCTTGGACTACATACCGCCTCCAGCATGAGGTTATTCCCCTGAGCAACAGTATCTCGCAGTAGACGA
GAATAGAACTTTAGCAAGAGACGGAACTCCGCAGGCTCACTCTTCAGCGAAAGAGCCGCAGTCTCCTGGATAA
TGTATGCATCAACTATGACATTCGCCTTGCCAATCTTCTGCACCAGAATGGACTGTGCAAGGGCAACGATCTT
GCTATCTTCACTATAAGTGGCGATTGTTACAATGGCATGTACCACATTTCTAGCTGTGACGGAAGTATCCCCC
TCCCCATCAATAGCAAGGGATAAGAAGCTCTCATTCGTGGGGGGAGCACGGTTCTCTATCGAATGAGAATAGC
CAGTGCCTGACTCGGCAGTTAGAGCGGTGGCAGCCTTATCAGCTGCCGATCCGGAGCTAAGGACGTTTCCGAG
AGAATATAGGGTGCCTATGACGGTATCTTGGGAAAGAAGCCGGAGGTTCTGGGCCAAACATTGGGCAGCCAGT
GCGACGACAGGACTGCTTTGGCCATTTTGGACAATGAAGCGAAGAAGAGAGCGGGTTAGGCCGTTTGCACTGC
CTCGGGATAGCCTTGCTAGAATGACAGCAGCCTTAAGGACAACGGTAGCTAGGTCCTTGTTCATCATTTGGTC
TGGATCTGAAATGGTATTGTCCAGGGATGTGAAGAGCAGGTCTAGATCCACTGAATCCTGGTCAACCATGATG
CAGTTGATCGAGCACATTATCGACAAAGCCTTGACCAAGAATGCAAGGCGTTGTTGCCAAGTGGAGCTGACTT
GTAGGTAATCCGACTCTTCTTCGAGGAGATTGATCTGGTCACCCGCGATTTCTGCGGCGTATTTTACCAGGCT
AATCATCCCGCGGTCTGAGGCTTTAGCTACATAACCACTGTCTGCATATCGGGAGAGAAGGGTATCATCTGGC
AGCGGTGGGGTTTGATCATTTTCAACAGGAGATGTGCAGGACTTTAGAAAGCCCATGAAAGCCTGTTGTAGCA
ACATGGAGCCAAGGGGTCGTCCGTCAGCCGCATACTGGCGGGGTGTACCGTCTCCAGTCGTGCAGCAGAGGAT
CTGGAGAGCGAGAGTTCCGTATGCCGGAAGAGATGGTCTCCACAGCTACCAGGAACCGCTCTGATAGAATGAT
GTGTAACTGCTTTACAATAGCAAGGCGATCCAGCGGTGCCCAAAAATGGGGGAATTCGGACGAGGCCCGGAGA
AATCCCACTAGCGATACCATTAGAGTAGTCACATCCGTAGATTCATGGCTCTCGACCTGTATCCCTGTCCCAT
CTGCATCAAAAACGGCCGGCAGGCGGTCTGTTGCCTGGTGACAATTCTCCAAGTACTCGTCAATGGCTTCGTG
GACGTCATCCTGCAGCGACTTGTGCCTAGAGCCCAGTGAAAGCAACGCCGTAACCACATCATGGGTCAAGGTC
TCCCACGGCGAGGGTTTGTAAATATGTAGGAAAGGAGACGAATGGATGCGGAGCGTATGGAGCGTAAGCAGGT
ATCCGACCAGTAGAGAAACCAGCTTTGCAGCATGGTCACCACGCTGAACAGAGCCAGCTGCCTTGCACAGAGC
AAGCAGTACGTCTAGTTCTCGAACCCCCTATCTTCCTCATTAGCCACTTTCTCGCGCATATGCCATTGAAGGA
GGGGTGTGTGTATGTATTGAGGCAGTAAAAGGGGGGGGCAGTACACTCGAGGCGCAGCGAGCCTGGGAGCCA
CTGGAAGCCGCCTTGGAGCCATTGCAGGCACTTATGAGCCTCTCGACGTCTGAATTGCCCTGATCATCGTCCT
GCCGGGGGTGTTTTGTGGCTAGTATCGCAAGTTTCTCGAGCGCCGCACGCCGGATATACGATGGTGAATGATG
CCTGAAGCTCTCCCCCGGCGGTTAGTTAGCAAATGTCTGCGCCCAACGTAGCACCAACACAGGGCGAGAACAC
CTACATATCCGGTCAACCAGCCTCGCCGTCATCCAGGAGGCCTTTCTTTTTCGTGGTTCCAAGGGGTGGGGAT
GCACCGAACGTGCAGTCGTGTGTTTTGGGGGCGTGGTCAGGAGTGTATCAGAAGTGCTCTGCCTGCTGGAGAT
GCGAGTCGCTGGTAGGATCAACCCGCCAAGCCGAGGCCCGTTTAGTGACGCCGGGGCAGAGAAACGACTGGGC
AGCAAGACGGCAAAGCGAGAGAGATGCATGAAAGGGCATTTTGATACACTCGATACGGACGCAGCCAGGGTTG
CCAACTTGTTGAATTACTCTGGACAAGCCACTCTTCCTCTACAGGGTTTTTCTCTTGCGCCTCCGGGCCTGTC

TCTGTCTCCCGGGGGGTTGAGCCCGCTACATGCAAGCCAGGCATTGAAAAACAGTCTGCGCGAGAAAACCTCG
ATGATCGACTTTTGTGCCTTAGGCTATGATACGGAAATGCTTTTACAAGATAGCTGGATATTTGCATCCAAGC
AGGAGTTGAAATAACACAAAGTGACGAAGGCAAAAAATAATAAGAAACTGCGGGATGCGGCTCTAACGGGCGG
CCAACCTTATATAAAGAGCGACTTCGCATCTTTCGACAGAAAAAAAAGGTCGAGCAAGGGAAGTTCGCTCAGC
CTGGCCTGCCATTGACTCTAAGCGCCGTCGTTCTCTTGCTTGGGGGCTTGCTGCAGCTTGGCCTGGGCTTGTC
CGGCCTGGAACTCAGGGAGCCGAAAGATAACTAGTTATTTATTTCGTCTCCGCACACCTCGGCGGCTGTCTCA
TAGTCGGGATATACATAGTACGGGGTAGCAGGTAGACTCACACGCTGCCTCTCTCTGCTGAACTGCTTCTACC
ACCTTCGACAGCGACACTGTTCGGTCTTGCAACTACGGTAGAGTGTGTTGGGGCTTGATCTTGCCTGCCATCG
ACTTGGAGTTGAAGTTGCGAAGCCATTGGCAGTCCATCCGGGATATTCGTAACTGCCGTCAACTTCTTCACGC
CGGGCCAAAAC**ATG**CCTCCGCAGAGGGAGAGAAGCAATCTGAAGAGAGAAAGGGTAAGATATGAACACGTTAT
ATCCAATTAGTTTCTCAGAAGGATGAAAATATTCAAGCTGCTAACTTCTTTTCCCAAACCTGCAGGGCAACGC
AGCTCCAGACCATGAATCAAAGAAGCCACGGAGATCTCCTCGAATCTCATCTCAATTGCAGCAGGATGCCGGA
TATCTACCTTCTCCCCTAGCTAATGAAGTAATCTCAGAAGAAAAGCGTGAAGTTGCTACAGTTTCTCCCCCAG
AAGGCCGCCGAAACCAGGCGCCTCGAGGTCAATCGCCAGACGAGTCGTCATGTCCCACCCAGGGTTTCTCTCA
GTATCCCCCAATCCAATCCATTGACGATGTGGAAGACGAGGTGGCCCAGGGCATATGGGGATATCTTACTCCG
CTAGATAACAATTTTGGAGGCCAATTGGTTCTGAAGAAGCGGGACAGCACAAATACTACACCGGTAATTGGAG
CAGCATCAGACATATCAACGCCGGAAAGCAACGCCTCAAAACGTGGAACTAGAGAGATAAACCGCGCTGGATA
TCTTATAGGCGGCATGCAGAGTGCGGTATGCCTTTACCCATTGCATACTCTCTGCCGTCTAGTGATCAATTGC
TAACCATCTGGGTACTGCAGATTTCAGGATCAAGCTCCCCACGATATCCAACCGCCATTGCCTGATATTCCAC
GAAAACAGGGGAGGAGACTTTGTTGCAATTGTGGAAGATCTCTCTATCAATGGCACTTTCATCAACGACGCCA
TCATCGGCAAGAACAAACGCCGAGAGCTCGAAAACGGCGACGAGATTACCATCCTGCAAGAATCCAGATTCAT
CTTCAACTACCCGCGGAGTCGGAACACAAGCAAGTTCCGACAGCAGTATAAGCTCTTTGATATGCTGGGTAGG
GGACACTTTGCCTCGGTTTATCTATGCGCTGAACGTTCTACGGGGATCAAGTATGCCGTCAAACATTTCGAGA
AACGCCCGGGCGCAGTTCAGAGGCTAGACCGTGAAGCACTACAGCAAGAGATTTCAATGCTGATGAGTGTCAA
CCATCCGAACATGCTGTGCCTAAAGGATACCTTTGACGAGAGCGATGGTGTCTTCCTGATTCTTGAGCTTGCG
CCAGAGGGCGAACTGTTCAACTGGATCATTAGGCATCAGAAACTCAGCGAGGAGGAGACTCGAAAAGTATTCA
TTCAGCTTTTTGAAGGTTTGAGATACCTGGTATGTCGTTCGTTTTCTGGTTGATCAGACTCATGGCAGTGTAT
ATGGCTAACCGTTGTCGTAGCATGAACGAAACATCATTCACCGTGACATAAAGCCAGAGAACATTCTGGTTGT
CGACAAAGATCTTACCGTAAAATTAGCAGACTTCGGCCTTGCAAAGATTGTCGGCGAACACTCCTTTACCACC
ACGCTGTAAGTTCACCTACCTTCTGCCGTTCAAATCTGGGGTCAATCTTACTAAGAGATCATAAAATCTAGAT
GTGGAACGCCTGGCTGTAAGTTTCCCTGCTGCTCTGTTAACGCTGGTGCTTTCATCCACCAAATGTGAATCTA
ACAATTCTTATCAGATGTTGCTCCTGAGATACTGGCAGAAAACGCTGAAGCCAGAATGTACACCCGAGCGGTT
GACATCTGGTCACTCGGAGTGGTACTCTACATCTGCCTGTGCGGATTCCCTCCTTTCTCAGATGAACTGTACC
ACCCAAGAGACTACCCATACACTCAGAAAGAGCAGATCCAGCTCGGAATATTTAAGTATCCTCTTCCATACTG
GGACTCAATCGATTATAGAGCCATGGACCTTATCGACTCCATGATCGAAGTCGACGTGAAGGAGCGTTTGAAA
GTAGATGAGTGTCTCCAGCACCCATGGATCACTGGCATCGACCCCGATGAGCCTAGGGTGGCAGACAGCACCG
ATGACCTAGCCGGGGCCATCGGTGGCCTGAGGGTGTCCAAAGAACGCCGGCCAACTATGGTCCGCAGGCTGTT
GAGTGACATACATGAAGTCACGGTCAAGCAGGTGGTGAAGACCAAGGAAGACGACCCTGCTTCCCAGGACATA

ACTATATACCAGAATCATCCTGTGCCTCGAGCCCAGGAGCCCAAACCGGCTGGCAACCGGAACTCAAAGGAGT
TCATGGAACTCGGTGGAAGTGGAGACCAGGTGTTATTCGATGAGCAGTCTGACGACTCGCTGGAGCTTGAGAG
TGAGGCACGGAAGCCAGTTGTCCACCAAGACGACGCAGACAAGACTCCACGAGAAAGAAAGAGG**TAG**CAGAAG
CCCGATCCGTCTTGGGATGGTATTCTGCTAGCTATACCGTGCTATTCCGGACTTTTGTTTCCAATTCCTTTTT
CGTTTATATTGATTGTGATCTTTCTTAATTTTTTCTTTAACTATACCCCCCGGTTGAAAGGTGATAGGGGGCC
AACTAACCGAAAAGCAGCAGAAAAACCGGCTTGGTCGCTGCCTTGGAGACGACGGCGATTCTCGTGTTTAGGA
TTTCATTTTTGAGTGATATAACTATGGGTACAATAGCGTCCACTGGGCAATGTAATTACAGTCTCTTCTCACT
CACCACCGGACAATAATAACAATATTAATATTGACGGACCGTTGATAGCTTAACCTGCCATGACTAAGCCGGC
CTCCCTGAAGCCGCGATGGCCTGATGATGCGCCCACCGTCAGGTCTCGCTCGCTCAGGCAGCAGAGAATCCAG
CGGAGGAGATGACGCCCTGCACGAACACCGTCCAGGGACCGTCTCCGCCCCGTCGCGAGCTCGAATTGTAATC
ATCTCGACGGCCGTTGGCCTGCTCGCCGCAAGGGAGAAAAAGGGGGGCAGAAGGGCAGCGGTAATTTTGGTAT
TGGAGATGCAGCTGTAAACCAATAAAAAACGACGTAAAATACAGGAACAATGCATTATCTTACAGTACCGTAC
GCCCCTTGCATAAAATGCGAGTTCGATCTCAAGATGCAACTTTTTTTTTCCCTTGCTTCGTCCGCAGCAGCTT
CAAGGCATTCATCACAAACAACGAACAAGTCTTGAGCGGAAGCAGGAGGATAAGACGATCACC**ATG**GGCCTCT
TAGCCGACATTGTCTCTCGTTTCTGCGAGAACTGCTCGACCCTGTCCACCGCCGCGCTCGTCGCAAGTGCCGT
ATCGGCTTTTATCGTCCTCTCCATTGTTATCAACGTCCTGCAGCAGCTCCTGTTCAAGGACCCTACAAAGCCT
CCGGTGGTCTTCCACTGGGTTCCGGTCATTGGAAGCACGATCTCCTATGGAATTGACCCGTACAAGTTCTTTG
ACGACTGCAAGGAGAAGGTTAGTTGGTGCAATTCAACTTAGCTATATACCAGGTTGCGCAAAGCTAACGTATA
AATAATTTCGGTACTCTAGTATGGAGACATCTTCACATTCATACTGCTGGGCAAGAAGACGACTGTTTTTCTC
GGTACAAAGGGAAATGATTTCATTTTGAACGGCAAGCTCAAGGATGTTTGCGCGGAGGATGTCTACTCCCCCT
CACCACCCCAGTGTTCGGACGACATGTGGTGTATGATTGCCCAAACTCCAAGCTCATGGAGCAGAAGAAGGTG
TGTACAGCAATTTATTCCCGTCTACATCTCCTGGGCCTCCATTGCTAATTTAATCGTCTACCAGTTCGTCAAG
TTCGGCCTCACCTCTGAAGCTCTCCGATCCTATGTCACCCTGATCACCAAGGAAGTTGAGCAGTTCTTCGAGT
CCTCCCCCGTCTTCAAGGCGACTCCGGAGTTTTCAACGTCAGCAAGGTCATGGCTGAAATCACCATCTACACC
GCCTCTCGATCTCTACAGGGCAAGGAGGTGCGAGGAAAGTTCGATTCCAGCTTTAGCGGAACTCTACTCCGAT
CTCGACATGGGCTTCGCCGCCATCAACTTCATGTTCCCATGGTTCCCCTTCCCACACAACCGCAAGCGTGACC
GTGCTCAAAAGAAGATGGCCCAGGTTTACACCGACATCATCCGTCAGCGACGTGAGGCTGGTGGAGAGAAAGA
CTCCGAGGACATGGTATGGAACTTGATGTCGTCCGTGTACAAGAATGGAACGCCAATTCCAGATATCGAAGTC
GCCCACATGATGATTGCTCTTCTTATGGCTGGCCAGCACTCTTCTTCCTCCACCGGCTCCTGGATCGTTCTCC
GCCTTGCCAGCCGTCCAGATATTCTCGAGGAACTCTACGAGGAACAGAAACGTGTTCTCGGCGAGGATCTTCC
ACCACTCACCTACGAATCTCTCCAGAAACTTGATCTTCACAACAATGTAATCAAGGAGACTCTCCGCCTCCAC
GCTCCCATCCACTCTATCCTCCGTGCTGTTAAATCCCCTATGCCCGTTGAAGGAACTAACTATGTTGTCCCAA
CCTCTCACAACCTCCTTGCCGCTCCTGGTGTTCCCTCACGAGACCCTCAGTACTTCCCTGACCCTCTTGTTTG
GAACCCTCACCGATGGGAGAACAACGTCGGTGTCACCGTAGTCGAGGCCAGTGAAGAAAAGACAGATTACGGA
TACGGTTTGGTTAGCAAGGGTGCCAACAGCCCTTACCTCCCATTCGGCTCAGGCAGACACAGATGCATCGGTG
AACAATTTGCATATGTTCAGCTTGGAACCGTAACAGCTACGTTAGCCAGACTAATGAAATGGAAGCAAGTTGA
AGGCACCAAAGATGTTGTGCCGCCAACTGACTATTCGGTAAGTTTGGCATATTACGCTCCTTTTTATTTTGCT
CGATTTTACTAACTTTGCATTAATAGTCCCTCTTCTCGAAGCCCCTTGGCGACCCAATGGTCTCGTGGGAGAA

GCGAAAGCAGCCTTCCCAGAAA**TGA**TAATCACGACTGCGGATTGAATCATGTTAAATTTCTTGCTTCTTGCTA
ATTTATTTCTTCTCTTTTCTTACGTTTATACACCGAGCCATATTTGCACTTTATGGTCACGCAAATAGGTTTT
CGGAATAGAGCATATGTAATAAAAACTTTCTTTTGACCATGCTTTTGAAGATGGGGGATGTAGATAATAGTAT
TATTCTAATACAGCCATTTATTACAATACTCTAGATGAACAGGAAAGTTGCAGTTACTCCGTTAGCTTTGGTG
CAGAAACAAAATGGTAATATACATTGGAAATAGTACATCTTCCCGTAGCTGGGATAATCTAGCATCTAAACAG
CGATATCCAAGCCAGTAATGTCAGGAAGTAACCAGCCAGCAACGTCGTTAATCCCGTGCCCCCTTTCAACTCC
GACGCTACAAAACGC**CTA**AGCAGCTGCCTGAGCATTCCTCCTCTCCGCCAACGCTCTCTTCGCTCTGATCTCC
TTCTGTCTTCTCTGACCACCGTCAATCTTTCTGCCACGCTTATGCTCCCACATGCCATCAGTAACCTGAACCT
TGACGGCGGTACTCCATGTGTTTGGAATAATCTGAGCATACGGCCCCTTGATCATATTCCCGTGGTGCAATTT
ATAATGGGCAAACTTTTTGAGTGGCTTGGAAGTATCCTTGAGCTCTGTTTCGCCTGGTGGTAGATTGACAATC
GCATAACGCATTCCAGGTGTAAGGGTAGCGGTGCCCATTGTGCTAACATCTAGGGACTCTCCGTCGGTGGCAT
TGGTAGTTTTTGCACTCTTTGACTTTTCGACGGGGACCTCAACAGCTCTCAGCTCAGCAACACCGTCAACAAC
GTTTTCGAAATCACCTCCTGGGCCGTAGTCCTGCTGTCTGAATTTCTCTCTCCACCGCAGTATGTATCGTCTG
TCCTTGTCATTTCTATGCCCAATTCTCGAAGCTCAGCCGAGTCCGCTGTAAACAATTGCTTCCATCCGGTGAT
TTTGCTGGAATATCTGGACATGTTTCGCCCAATTAGGCGTAGGAATGTTTCGACATCGGGCACGAATGGGGTA
GGTGATGGCACTGTTGCGGGTTTTCGAGATTGATGAAGGTACCGTACGCATGGCTGATAATGGGGGTTTGTGA
CGGAAGTGAAGAGCCGTAGTGGAAGGGATGTTCTGGCATTAAAGCCCGGTATGCGCGCCGCAGC**CAT**AGTGGT
TATATCGCCTTATATCAAACCGTTCAATGTCGAGATCTGGGGGGTGAAGATTTTAATTTAGCTCGTGGTGTAA
GAACACCATCGGCGGGCGTGACGAAAGCGAAAAAAGCCACGGCACCAAGGGCCCAGGTAAACCGGTAAGGGAC
TCAAGGCGAGTCAATCCAATGCTCAATGGCGCTGTATAGAAGATGTTCAACTGGTTCTTCGACTTCGTTCAAG
ATTTTGCACCAAAATTTTGAATTAGGTCCGACGGCCTTAGCTCGGATCAACAAAACGCCGATCTCCGGTGAGC
TCGGGCCAGCCGAAGCTTCCATCGCAACATTCAAAGATGAAAGACGACGATATATATTTTGATAACAAACAAA
GCTGTATTGTTGATTGAATACTAGGAGAGCTGCTCAGAGCAGTGTCTCACTCTTGTGCCACTCTATCCTTTGT
CTCGCGAAATTGGTGGAAGCCAATTTCGCTTGACATTATGGCAGTGTCGCCGCTTCCCTCTCAATTCCCTCCC
ATCACTACTACCGGTGCTCCAAAGTCGTTAGCGATGAGAAATATTGCTTCTCAAGAGCCTCGGACGGCGCCTA
GTGGTGATCCTGCAGTTCCTAAGCCCTCCATGCCCGAACCAGAGCTGGCGGAGCAGTTAAACGCTGATGTACG
GCGGAAATATGTCAAAGGTGGGCGGGCAAATACACCCCAACCATTGGGACCCCATAATGCTAACATTTGGTTA
TTCTGAAAGATAAGAAGCTCGGTGAAGGTACATATGCAGTTGTTTACCTAGGCCATCTACGAGATGATCCCAC
TTCGCTAATAAAGAGCGACTTCGCATCTTTCGACAGAAAAAAAAGGTCGAGCAAGGGAAGTTCGCTCAGCCTG
GCCTGCCATTGACTCTAAGCGCCGTCGTTCTCTTGCTTGGGGGCTTGCTGCAGCTTGGCCTGGGCTTGTCCGG
CCTGGAACTCAGGGAGCCGAAAGATAACTAGTTATTTATTTCGTCTCCGCACACCTCGGCGGCTGTCTCATAG
TCGGGATATACATAGTACGGGGTAGCAGGTAGACTCACACGCTGCCTCTCTCTGCTGAACTGCTTCTACCACC
TTCGACAGCGACACTGTTCGGTCTTGCAACTACGGTAGAGTGTGTTGGGGCTTGATCTTGCCTGCCATCGACT
TGGAGTTGAAGTTGCGAAGCCATTGGCAGTCCATCCGGGATATTCGTAACTGCCGTCAACTTCTTCACGCCGG
GCCAAAAC**ATG**CCTCCGCAGAGGGAGAGAAGCAATCTGAAGAGAGAAAGGGTAAGATATGAACACGTTATATC
CAATTAGTTTCTCAGAAGGATGAAAATATTCAAGCTGCTAACTTCTTTTCCCAAACCTGCAGGGCAACGCAGC
TCCAGACCATGAATCAAAGAAGCCACGGAGATCTCCTCGAATCTCATCTCAATTGCAGCAGGATGCCGGATAT
CTACCTTCTCCCCTAGCTAATGAAGTAATCTCAGAAGAAAAGCGTGAAGTTGCTACAGTTTCTCCCCAGAAGG

CCGCCGAAACCAGGCGCCTCGAGGTCAATCGCCAGACGAGTCGTCATGTCCCACCCAGGGTTTCTCTCAGTAT
CCCCCAATCCAATCCATTGACGATGTGGAAGACGAGGTGGCCCAGGGCATATGGGGATATCTTACTCCGCTAG
ATAACAATTTTGGAGGCCAATTGGTTCTGAAGAAGCGGGACAGCACAAATACTACACCGGTAATTGGAGCAGC
ATCAGACATATCAACGCCGGAAAGCAACGCCTCAAAACGTGGAACTAGAGAGATAAACCGCGCTGGATATCTT
ATAGGCCGGCATGCAGAGTGCGGTATGCCTTTACCCATTGCATACTCTCTGCCGTCTAGTGATCAATTGCTAA
CCATCTGGGTACTGCAGATTTCAGGATCAAGCTCCCCACGATATCCAACCGCCATTGCCTGATATTCCACGAA
AACAGGGGAGGAGACTTTGTTGCAATTGTGGAAGATCTCTCTATCAATGGCACTTTCATCAACGACGCCATCA
TCGGCAAGAACAAACGCCGAGAGCTCGAAAACGGCGACGAGATTACCATCCTGCAAGAATCCAGATTCATCTT
CAACTACCCGCGGAGTCGGAACACAAGCAAGTTCCGACAGCAGTATAAGCTCTTTGATATGCTGGGTAGGGGA
CACTTTGCCTCGGTTTATCTATGCGCTGAACGTTCTACGGGGATCAAGTATGCCGTCAAACATTTCGAGAAAC
GCCCGGGCGCAGTTCAGAGGCTAGACCGTGAAGCACTACAGCAAGAGATTTCAATGCTGATGAGTGTCAACCA
TCCGAACATGCTGTGCCTAAAGGATACCTTTGACGAGAGCGATGGTGTCTTCCTGATTCTTGAGCTTGCGCCA
GAGGGCGAACTGTTCAACTGGATCATTAGGCATCAGAAACTCAGCGAGGAGGAGACTCGAAAAGTATTCATTC
AGCTTTTTGAAGGTTTGAGATACCTGGTATGTCGTTCGTTTTCTGGTTGATCAGACTCATGGCAGTGTATATG
GCTAACCGTTGTCGTAGCATGAACGAAACATCATTCACCGTGACATAAAGCCAGAGAACATTCTGGTTGTCGA
CAAAGATCTTACCGTAAAATTAGCAGACTTCGGCCTTGCAAAGATTGTCGGCGAACACTCCTTTACCACCACG
CTGTAAGTTCACCTACCTTCTGCCGTTCAAATCTGGGGTCAATCTTACTAAGAGATCATAAAATCTAGATGTG
GAACGCCTGGCTGTAAGTTTCCCTGCTGCTCTGTTAACGCTGGTGCTTTCATCCACCAAATGTGAATCTAACA
ATTCTTATCAGATGTTGCTCCTGAGATACTGGCAGAAAACGCTGAAGCCAGAATGTACACCCGAGCGGTTGAC
ATCTGGTCACTCGGAGTGGTACTCTACATCTGCCTGTGCGGATTCCCTCCTTTCTCAGATGAACTGTACCACC
CAAGAGACTACCCATACACTCAGAAAGAGCAGATCCAGCTCGGAATATTTAAGTATCCTCTTCCATACTGGGA
CTCAATCGATTATAGAGCCATGGACCTTATCGACTCCATGATCGAAGTCGACGTGAAGGAGCGTTTGAAAGTA
GATGAGTGTCTCCAGCACCCATGGATCACTGGCATCGACCCCGATGAGCCTAGGGTGGCAGACAGCACCGATG
ACCTAGCCGGGGCCATCGGTGGCCTGAGGGTGTCCAAAGAACGCCGGCCAACTATGGTCCGCAGGCTGTTGAG
TGACATACATGAAGTCACGGTCAAGCAGGTGGTGAAGACCAAGGAAGACGACCCTGCTTCCCAGGACATAACT
ATATACCAGAATCATCCTGTGCCTCGAGCCCAGGAGCCCAAACCGGCTGGCAACCGGAACTCAAAGGAGTTCA
TGGAACTCGGTGGAAGTGGAGACCAGGTGTTATTCGATGAGCAGTCTGACGACTCGCTGGAGCTTGAGAGTGA
GGCACGGAAGCCAGTTGTCCACCAAGACGACGCAGACAAGACTCCACGAGAAAGAAAGAGG**TAG**CAGAAGCCC
GATCCGTCTTGGGATGGTATTCTGCTAGCTATACCGTGCTATTCCGGACTTTTGTTTCCAATTCCTTTTTCGT
TTATATTGATTGTGATCTTTCTTAATTTTTTCTTTAACTATACCCCCGGTTGAAAGGTGATAGGGGGCCAAC
TAACCGAAAAGCAGCAGAAAAACCGGCTTGGTCGCTGCCTTGGAGACGACGGCGATTCTCGTGTTTAGGATTT
CATTTTTGAGTGATATAACTATGGGTACAATAGCGTCCACTGGGCAATGTAATTACAGTCTCTTCTCACTCAC
CACCGGACAATAATAACAATATTAATATTGACGGACCGTTGATAGCTTAACCTGCCATGACTAAGCCGGCCTC
CCTGAAGCCGCGATGGCCTGATGATGCGCCCACCGTCAGGTCTCGCTCGCTCAGGCAGCAGAGAATCCAGCGG
AGGAGATGACGCCCTGCACGAACACCGTCCAGGGACCGTCTCCGCCCCGTCGCGAGCTCGAATTGTAATCATC
TCGACGGCGTTGGCCTGCTCGCCGCAAGGGAGAAAAAGGGGGGCAGAAGGGCAGCGGTAATTTTGGTATTGGA
GATGCAGCTGTAAACCAATAAAAAACGACGTAAAATACCAGGAACAATGCATTATCTTACAGTACCGTACGCC
CCTTGCATAAAATGCGAGTTCGATCTCAAGATGCAACTTTTTTTTTCCCTTGCTTCGTCCGCAGCAGCTTCAA

GGCATTCATCACAAACAACGAACAAGTCTTGAGCGGAAGCAGGAGGATAAGACGATCACC**ATG**GGCCTCTTAG
CCGACATTGTCTCTCGTTTCTGCGAGAACTGCTCGACCCTGTCCACCGCCGCGCT

[Read No.m64281e_230311_112734/100860170/ccs] [Sense (+) strand]

AAT~ACC (5'UTR)

ATG~TAG (*chkB* protein kinase genomic DNA) (Forward sequence)

ATG~TGA (*TinCYP51B* gene) (Forward sequence)

TAA~GCT (3'UTR)

CTA~CAT (*FYV4* genomic DNA) (Reverse sequence)

TAATTGGAGCAGCATCAGACATATCAACGCCGGAAAGCAACGCCTCAAAACGTGGAACTAGAGAGATAAACCG
CGCTGGATATCTTATAGGCCGGCATGCAGAGTGCGGTATGCCTTTACCCATTGCATACTCTCTGCCGTCTAGT
GATCAATTGCTAACCATCTGGGTACTGCAGATTTCAGGATCAAGCTCCCCACGATATCCAACCGCCATTGCCT
GATATTCCACGAAAACAGGGGAGGAGACTTTGTTGCAATTGTGGAAGATCTCTCTATCAATGGCACTTTCATC
AACGACGCCATCATCGGCAAGAACAAACGCCGAGAGCTCGAAAACGGCGACGAGATTACCATCCTGCAAGAAT
CCAGATTCATCTTCAACTACCCGCGGAGTCGGAACACAAGCAAGTTCCGACAGCAGTATAAGCTCTTTGATAT
GCTGGGTAGGGACACTTTGCCTCGGTTTATCTATGCGCTGAACGTTCTACGGGGATCAAGTATGCCGTCAAAC
ATTTCGAGAAACGCCCGGGCGCAGTTCAGAGGCTAGACCGTGAAGCACTACAGCAAGAGATTTCAATGCTGAT
GAGTGTCAACCATCCGAACATGCTGTGCCTAAAGGATACCTTTGACGAGAGCGATGGTGTCTTCCTGATTCTT
GAGCTTGCGCCAGAGGGCGAACTGTTCAACTGGATCATTAGGCATCAGAAACTCAGCGAGGAGGAGACTCGAA
AAGTATTCATTCAGCTTTTTGAAGGTTTGAGATACCTGGTATGTCGTTCGTTTTCTGGTTGATCAGACTCATG
GCAGTGTATATGGCTAACCGTTGTCGTAGCATGAACGAAACATCATTCACCGTGACATAAAGCCAGAGAACAT
TCTGGTTGTCGACAAAGATCTTACCGTAAAATTAGCAGACTTCGGCCTTGCAAAGATTGTCGGCGAACACTCC
TTTACCACCACGCTGTAAGTTCACCTACCTTCTGCCGTTCAAATCTGGGGTCAATCTTACTAAGAGATCATAA
AATCTAGATGTGGAACGCCTGGCTGTAAGTTTCCCTGCTGCTCTGTTAACGCTGGTGCTTTCATCCACCAAAT
GTGAATCTAACAATTCTTATCAGATGTTGCTCCTGAGATACTGGCAGAAAACGCTGAAGCCAGAATGTACACC
CGAGCGGTTGACATCTGGTCACTCGGAGTGGTACTCTACATCTGCCTGTGCGGATTCCCTCCTTTCTCAGATG
AACTGTACCACCCAAGAGACTACCCATACACTCAGAAAGAGCAGATCCAGCTCGGAATATTTAAGTATCCTCT
TCCATACTGGGACTCAATCGATTATAGAGCCATGGACCTTATCGACTCCATGATCGAAGTCGACGTGAAGGAG
CGTTTGAAAGTAGATGAGTGTCTCCAGCACCCATGGATCACTGGCATCGACCCCGATGAGCCTAGGGTGGCAG
ACAGCACCGATGACCTAGCCGGGGCCATCGGTGGCCTGAGGGTGTCCAAAGAACGCCGGCCAACTATGGTCCG
CAGGCTGTTGAGTGACATACATGAAGTCACGGTCAAGCAGGTGGTGAAGACCAAGGAAGACGACCCTGCTTCC
CAGGACATAAACTATATACCAGAATCATCCTGTGCCTCGAGCCCAGGAGCCCAAACCGGCTGGCAACCGGAAC
TCAAAGGAGTTCATGGAACTCGGTGGAAGTGGAGACCAGGTGTTATTCGATGAGCAGTCTGACGACTCGCTGG
AGCTTGAGAGTGAGGCACGGAAGCCAGTTGTCCACCAAGACGACGCAGACAAGACTCCACGAGAAAGAAAGAG
G**TAG**CAGAAGCCCGATCCGTCTTGGGATGGTATTCTGCTAGCTATACCGTGCTATTCCGGACTTTTGTTTCCA
ATTCCTTTTTCGTTTATATTGATTGTGATCTTTCTTAATTTTTTCTTTAACTATACCCCCCGGTTGAAAGGTG

ATAGGGGGCCAACTAACCGAAAAGCAGCAGAAAAACCGGCTTGGTCGCTGCCTTGGAGACGACGGCGATTCTC
GTGTTTAGGATTTCATTTTTGAGTGATATAACTATGGGTACAATAGCGTCCACTGGGCAATGTAATTACAGTC
TCTTCTCACTCACCACCGGACAATAATAACAATATTAATATTGACGGACCGTTGATAGCTTAACCTGCCATGA
CTAAGCCGGCCTCCCTGAAGCCGCGATGGCCTGATGATGCGCCCACCGTCAGGTCTCGCTCGCTCAGGCAGCA
GAGAATCCAGCGGAGGAGATGACGCCCTGCACGAACACCGTCCAGGGACCGTCTCCGCCCCGTCGCGAGCTCG
AATTGTAATCATCTCGACGGCCGTTGGCCTGCTCGCCGCAAGGGAGAAAAAGGGGGGCAGAAGGGCAGCGGTA
ATTTTGGTATTGGAGATGCAGCTGTAAACCAATAAAAAACGACGTAAAATACCAGGAACAATGCATTATCTTA
CAGTACCGTACGCCCCTTGCATAAAATGCGAGTTCGATCTCAAGATGCAACTTTTTTTTCCCTTGCTTCGTC
CGCAGCAGCTTCAAGGCATTCATCACAAACAACGAACAAGTCTTGAGCGGAAGCAGGAGGATAAGACGATCAC
C**ATG**GGCCTCTTAGCCGACATTGTCTCTCGTTTCTGCGAGAACTGCTCGACCCTGTCCACCGCCGCGCTCGTC
GCAAGTGCCGTATCGGCTTTTATCGTCCTCTCCATTGTTATCAACGTCCTGCAGCAGCTCCTGTTCAAGGACC
CTACAAAGCCTCCGGTGGTCTTCCACTGGGTTCCGGTCATTGGAAGCACGATCTCCTATGGAATTGACCCGTA
CAAGTTCTTTGACGACTGCAAGGAGAAGGTTAGTTGGTGCAATTCAACTTAGCTATATACCAGGTTGCGCAAA
GCTAACGTATAAATAATTTCGGTACTCTAGTATGGAGACATCTTCACATTCATACTGCTGGGCAAGAAGACGA
CTGTTTTTCTCGGTACAAAGGGAAATGATTTCATTTTGAACGGCAAGCTCAAGGATGTTTGCGCGGAGGATGT
CTACTCCCCCCTCACCACCCCAGTGTTCGGACGACATGTGGTGTATGATTGCCCAAACTCCAAGCTCATGGAG
CAGAAGAAGGTGTGTACAGCAATTTATTCCCGTCTACATCTCCTGGGCCTCCATTGCTAATTTAATCGTCTAC
CAGTTCGTCAAGTTCGGCCTCACCTCTGAAGCTCTCCGATCCTATGTCACCCTGATCACCAAGGAAGTTGAGC
AGTTCTTCGAGTCCTCCCCCGTCTTCAAGGGCGACTCCGGAGTTTTCAACGTCAGCAAGGTCATGGCTGAAAT
CACCATCTACACCGCCTCTCGATCTCTACAGGGCAAGGAGGTGCGAGGAAAGTTCGATTCCAGCTTTGCGGAA
CTCTACTCCGATCTCGACATGGGCTTCGCCGCCATCAACTTCATGTTCCCATGGTTCCCCTTCCCACACAACC
GCAAGCGTGACCGTGCTCAAAAGAAGATGGCCCAGGTTTACACCGACATCATCCGTCAGCGACGTGAGGCTGG
TGGAGAGAAAGACTCCGAGGACATGGTATGGAACTTGATGTCGTCCGTGTACAAGAATGGAACGCCAATTCCA
GATATCGAAGTCGCCCACATGATGATTGCTCTTCTTATGGCTGGCCAGCACTCTTCTTCCTCCACCGGCTCCT
GGATCGTTCTCCGCCTTGCCAGCCGTCCAGATATTCTCGAGGAACTCTACGAGGAACAGAAACGTGTTCTCGG
CGAGGATCTTCCACCACTCACCTACGAATCTCTCCAGAAACTTGATCTTCACAACAATGTAATCAAGGAGACT
CTCCGCCTCCACGCTCCCATCCACTCTATCCTCCGTGCTGTTAAATCCCCTATGCCCGTTGAAGGAACTAACT
ATGTTGTCCCAACCTCTCACAACCTCCTTGCCGCTCCTGGTGTTCCCTCACGAGACCCTCAGTACTTCCCTGA
CCCTCTTGTTTGGAACCCTCACCGATGGGAGAACAACGTCGGTGTCACCGTAGTCGAGGCCAGTGAAGAAAAG
ACAGATTACGGATACGGTTTGGTTAGCAAGGGTGCCAACAGCCCTTACCTCCCATTCGGCTCAGGCAGACACA
GATGCATCGGTGAACAATTTGCATATGTTCAGCTTGGAACCGTAACAGCTACGTTAGCCAGACTAATGAAATG
GAAGCAAGTTGAAGGCACCAAAGATGTTGTGCCGCCAACTGACTATTCGGTAAGTTTGGCATATTACGCTCCT
TTTTATTTTGCTCGATTTTACTAACTTTGCATTAATAGTCCCTCTTCTCGAAGCCCCTTGGCGACCCAATGGT
CTCGTGGGAGAAGCGAAAGCAGCCTTCCCAGAAA**TGA**TAATCACGACTGCGGATTGAATCATGTTAAATTTCT
TGCTTCTTGCTAATTTATTTCTTCTCTTTTCTTACGTTTATACACCGAGCCATATTTGCACTTTATGGTCACG
CAAATAGGTTTTCGGAATAGAGCATATGTAATAAAAACTTTCTTTTGACCATGCTTTTGAAGATGGGGGATGT
AGATAATAGTATTATTCTAATACAGCCATTTATTACAATACTCTAGATGAACAGGAAAGTTGCAGTTACTCCG
TTAGCTTTGGTGCAGAAACAAAATGGTAATATACATTGGAAATAGTACATCTTCCCGTAGCTGGGATAATCTA

GCATCTAAACAGCGATATCCAAGCCAGTAATGTCAGGAAGTAACCAGCCAGCAACGTCGTTAATCCCGTGCCC
CCTTTCAACTCCGACGCTACAAAACGC**CTA**AGCAGCTGCCTGAGCATTCCTCCTCTCCGCCAACGCTCTCTTC
GCTCTGATCTCCTTCTGTCTTCTCTGACCACCGTCAATCTTTCTGCCACGCTTATGCTCCCACATGCCATCAG
TAACCTGAACCTTGACGGCGGTACTCCATGTGTTTGGAATAATCTGAGCATACGGCCCCTTGATCATATTCCC
GTGGTGCAATTTATAATGGGCAAACTTTTTGAGTGGCTTGGAAGTATCCTTGAGCTCTGTTTCGCCTGGTGGT
AGATTGACAATCGCATAACGCATTCCAGGTGTAAGGGTAGCGGTGCCCATTGTGCTAACATCTAGGGACTCTC
CGTCGGTGGCATTGGTAGTTTTTGCACTCTTTGACTTTTCGACGGGGACCTCAACAGCTCTCAGCTCAGCAAC
ACCGTCAACAACGTTTTCGAAATCACCTCCTGGGCCGTAGTCCTGCTGTCTGAATTTCTCTCTCCACCGCAGT
ATGTATCGTCTGTCCCTTGTCATTTCTATGCCCAATTCTCGAAGCTCAGCCGAGTCCGCTGTAAACAATTGCT
TCCATCCGGTGATTTTGCTGGAATATCTGGACATGTTTCGCCCAATTAGGCGTAGGAATGTTTCGACATCGGG
CACGAATGGGGTAGGTGATGGCACTGTTGCGGGTTTTCGAGATTGATGAAGGTACCGTACGCATGGCTGATAA
TGGGGGTTTGTGACGGAAGTGAAGAGCCGTAGTGGAAGGGATGTTCTGGCATTAAAGCCCGGTATGCGCGCCG
CAGC**CAT**AGTGGTTATATCGCCTTATATCAAACCGTTCAATGTCGAGATCTGGGGGTGAAGATTTTAATTTAG
CTCGTGGTGTAAGAACACCATCGGCGGGCGTGACGAAAGCGAAAAAAGCCACGGCACCAAGGGCCCAGGTAAA
CCGGTAAGGGACTCAAGGCGAGTCAATCCAATGCTCAATGGCGCTGTATAGAAGATGTTCAACTGGTTCTTCG
ACTTCGTTCAAGATTTTGCACCAAAATTTTGAATTAGGTCCGACGGCCTTAGCTCGGATCAACAAAACGCCGA
TCTCCGGTGAGCTCGGGCCAGCCGAAGCTTCCATCGCAACATTCAAAGATGAAAGACGACGATATATATTTTG
ATAACAAACAAAGCTGTATTGTTGATTGAATACTAGGAGAGCTGCTCAGAGCAGTGTCTCACTCTTGTGCCAC
TCTATCCTTTGTCTCGCGAAATTGGTGGAAGCCAATTTCGCTTGACATTATGGCAGTGTCGCCGCTTCCCTCT
CAATTCCCTCCCATCACTACTACCGGTGCTCCAAAGTCGTTAGCGATGAGAAATATTGCTTCTCAAGAGCCTC
GGACGGCGCCTAGTGGTGATCCTGCAGTTCCTAAGCCCTCCATGCCCGAACCAGAGCTGGCGGAGCAGTTAAA
CGCTGATGTACGGCGGAAATATGTCAAAGGTGGGCGGGCAAATACACCCCAACCATTGGGACCCCATAATGCT
AACATTTGGTTATTCTGAAAGATAAGAAGCTCGGTGAAGGTACATATGCAGTTGTTTACCTAGGCCATCTACG
AGATGATCCCACTTCGCTAATAAAGAGCGACTTCGCATCTTTCGACAGAAAAAAAAGGTCGAGCAAGGGAAGT
TCGCTCAGCCTGGCCTGCCATTGACTCTAAGCGCCGTCGTTCTCTTGCTTGGGGGCTTGCTGCAGCTTGGCCT
GGGCTTGTCCGGCCTGGAACTCAGGGAGCCGAAAGATAACTAGTTATTTATTTCGTCTCCGCACACCTCGGCG
GCTGTCTCATAGTCGGGATATACATAGTACGGGGTAGCAGGTAGACTCACACGCTGCCTCTCTCTGCTGAACT
GCTTCTACCACCTTCGACAGCGACACTGTTCGGTCTTGCAACTACGGTAGAGTGTGTTGGGGCTTGATCTTGC
CTGCCATCGACTTGGAGTTGAAGTTGCGAAGCCATTGGCAGTCCATCCGGGATATTCGTAACTGCCGTCAACT
TCTTCACGCCGGGCCAAAAC**ATG**CCTCCGCAGAGGGAGAGAAGCAATCTGAAGAGAGAAAGGGTAAGATATGA
ACACGTTATATCCAATTAGTTTCTCAGAAGGATGAAAATATTCAAGCTGCTAACTTCTTTTCCCAAACCTGCA
GGGCAACGCAGCTCCAGACCATGAATCAAAGAAGCCACGGAGATCTCCTCGAATCTCATCTCAATTGCAGCAG
GATGCCGGATATCTACCTTCTCCCCTAGCTAATGAAGTAATCTCAGAAGAAAAGCGTGAAGTTGCTACAGTTT
CTCCCCCAGAAGGCCGCCGAAACCAGGCGCCTCGAGGTCAATCGCCAGACGAGTCGTCATGTCCCACCCAGGG
TTTCTCTCAGTATCCCCCAATCCAATCCATTGACGATGTGGAAGACGAGGTGGCCCAGGGCATATGGGGATAT
CTTACTCCGCTAGATAACAATTTTGGAGGCCAATTGGTTCTGAAGAAGCGGGACAGCACAAATACTACACCGG
TAATTGGAGCAGCATCAGACATATCAACGCCGGAAAGCAACGCCTCAAAACGTGGAACTAGAGAGATAAACCG
CGCTGGATATCTTATAGGCCGGCATGCAGAGTGCGGTATGCCTTTACCCATTGCATACTCTCTGCCGTCTAGT

GATCAATTGCTAACCATCTGGGTACTGCAGATTTCAGGATCAAGCTCCCCACGATATCCAACCGCCATTGCCT
GATATTCCACGAAAACAGGGGAGGAGACTTTGTTGCAATTGTGGAAGATCTCTCTATCAATGGCACTTTCATC
AACGACGCCATCATCGGCAAGAACAAACGCCGAGAGCTCGAAAACGGCGACGAGATTACCATCCTGCAAGAAT
CCAGATTCATCTTCAACTACCCGCGGAGTCGGAACACAAGCAAGTTCCGACAGCAGTATAAGCTCTTTGATAT
GCTGGGTAGGGACACTTTGCCTCGGTTTATCTATGCGCTGAACGTTCTACGGGGATCAAGTATGCCGTCAAAC
ATTTCGAGAAACGCCCGGGCGCAGTTCAGAGGCTAGACCGTGAAGCACTACAGCAAGAGATTTCAATGCTGAT
GAGTGTCAACCATCCGAACATGCTGTGCCTAAAGGATACCTTTGACGAGAGCGATGGTGTCTTCCTGATTCTT
GAGCTTGCGCCAGAGGGCGAACTGTTCAACTGGATCATTAGGCATCAGAAACTCAGCGAGGAGGAGACTCGAA
AAGTATTCATTCAGCTTTTTGAAGGTTTGAGATACCTGGTATGTCGTTCGTTTTCTGGTTGATCAGACTCATG
GCAGTGTATATGGCTAACCGTTGTCGTAGCATGAACGAAACATCATTCACCGTGACATAAAGCCAGAGAACAT
TCTGGTTGTCGACAAAGATCTTACCGTAAAATTAGCAGACTTCGGCCTTGCAAAGATTGTCGGCGAACACTCC
TTTACCACCACGCTGTAAGTTCACCTACCTTCTGCCGTTCAAATCTGGGGTCAATCTTACTAAGAGATCATAA
AATCTAGATGTGGAACGCCTGGCTGTAAGTTTCCCTGCTGCTCTGTTAACGCTGGTGCTTTCATCCACCAAAT
GTGAATCTAACAATTCTTATCAGATGTTGCTCCTGAGATACTGGCAGAAAACGCTGAAGCCAGAATGTACACC
CGAGCGGTTGACATCTGGTCACTCGGAGTGGTACTCTACATCTGCCTGTGCGGATTCCCTCCTTTCTCAGATG
AACTGTACCACCCAAGAGACTACCCATACACTCAGAAAGAGCAGATCCAGCTCGGAATATTTAAGTATCCTCT
TCCATACTGGGACTCAATCGATTATAGAGCCATGGACCTTATCGACTCCATGATCGAAGTCGACGTGAAGGAG
CGTTTGAAAGTAGATGAGTGTCTCCAGCACCCATGGATCACTGGCATCGACCCCGATGAGCCTAGGGTGGCAG
ACAGCACCGATGACCTAGCCGGGGCCATCGGTGGCCTGAGGGTGTCCAAAGAACGCCGGCCAACTATGGTCCG
CAGGCTGTTGAGTGACATACATGAAGTCACGGTCAAGCAGGTGGTGAAGACCAAGGAAGACGACCCTGCTTCC
CAGGACATAACTATATACCAGAATCATCCTGTGCCTCGAGCCCAGGAGCCCAAACCGGCTGGCAACCGGAACT
CAAAGGAGTTCATGGAACTCGGTGGAAGTGGAGACCAGGTGTTATTCGATGAGCAGTCTGACGACTCGCTGGA
GCTTGAGAGTGAGGCACGGAAGCCAGTTGTCCACCAAGACGACGCAGACAAGACTCCACGAGAAAGAAAGAGG
TAGCAGAAGCCCGATCCGTCTTGGGATGGTATTCTGCTAGCTATACCGTGCTATTCCGGACTTTTGTTTCCAA
TTCCTTTTTCGTTTATATTGATTGTGATCTTTCTTAATTTTTTCTTTAACTATACCCCCGGTTGAAAGGTGA
TAGGGGGCCAACTAACCGAAAAGCAGCAGAAAAACCGGCTTGGTCGCTGCCTTGGAGACGACGGCGATTCTCG
TGTTTAGGATTTCATTTTTGAGTGATATAACTATGGGTACAATAGCGTCCACTGGGCAATGTAATTACAGTCT
CTTCTCACTCACCACCGGACAATAATAACAATATTAATATTGACGGACCGTTGATAGCTTAACCTGCCATGAC
TAAGCCGGCCTCCCTGAAGCCGCGATGGCCTGATGATGCGCCCACCGTCAGGTCTCGCTCGCTCAGGCAGCAG
AGAATCCAGCGGAGGAGATGACGCCCTGCACGAACACCGTCCAGGGACCGTCTCCGCCCCGTCGCGAGCTCGA
ATTGTAATCATCTCGACGGCCGTTGGCCTGCTCGCCGCAAGGGAGAAAAAGGGGGGCAGAAGGGCAGCGGTAA
TTTTGGTATTGGAGATGCAGCTGTAAACCAATAAAAAACGACGTAAAATACCAGGAACAATGCATTATCTTAC
AGTACCGTACGCCCCTTGCATAAAATGCGAGTTCGATCTCAAGATGCAACTTTTTTTTTCCCTTGCTTCGTCC
GCAGCAGCTTCAAGGCATTCATCACAAACAACGAACAAGTCTTGAGCGGAAGCAGGAGGATAAGACGATCACC
ATGGGCCTCTTAGCCGACATTGTCTCTCGTTTCTGCGAGAACTGCTCGACCCTGTCCACCGCCGCGCTCGTCG
CAAGTGCCGTATCGGCTTTTATCGTCCTCTCCATTGTTATCAACGTCCTGCAGCAGCTCCTGTTCAAGGACCC
TACAAAGCCTCCGGTGGTCTTCCACTGGGTTCCGGTCATTGGAAGCACGATCTCCTATGGAATTGACCCGTAC
AAGTTCTTTGACGACTGCAAGGAGAAGGTTAGTTGGTGCAATTCAACTTAGCTATATACCAGGTTGCGCAAAG

CTAACGTATAAATAATTTCGGTACTCTAGTATGGAGACATCTTCACATTCATACTGCTGGGCAAGAAGACGAC
TGTTTTTCTCGGTACAAAGGGAAATGATTTCATTTTGAACGGCAAGCTCAAGGATGTTTGCGCGGAGGATGTC
TACTCCCCCCTCACCACCCCAGTGTTCGGACGACATGTGGTGTATGATTGCCCAAACTCCAAGCTCATGGAGC
AGAAGAAGGTGTGTACAGCAATTTATTCCCGTCTACATCTCCTGGGCCTCCATTGCTAATTTAATCGTCTACC
AGTTCGTCAAGTTCGGCCTCACCTCTGAAGCTCTCCGATCCTATGTCACCCTGATCACCAAGGAAGTTGAGCA
GTTCTTCGAGTCCTCCCCCGTCTTCAAGGGCGACTCCGGAGTTTTCAACGTCAGCAAGGTCATGGCTGAAATC
ACCATCTACACCGCCTCTCGATCTCTACAGGGCAAGGAGGTGCGAGGAAAGTTCGATTCCAGCTTTGCGGAAC
TCTACTCCGATCTCGACATGGGCTTCGCCGCCATCAACTTCATGTTCCCATGGTTCCCCTTCCCACACAACCG
CAAGCGTGACCGTGCTCAAAAGAAGATGGCCCAGGTTTACACCGACATCATCCGTCAGCGACGTGAGGCTGGT
GGAGAGAAAGACTCCGAGGACATGGTATGGAACTTGATGTCGTCCGTGTACAAGAATGGAACGCCAATTCCAG
ATATCGAAGTCGCCCACATGATGATTGCTCTTCTTATGGCTGGCCAGCACTCTTCTTCCTCCACCGGCTCCTG
GATCGTTCTCCGCCTTGCCAGCCGTCCAGATATTCTCGAGGAACTCTACGAGGAACAGAAACGTGTTCTCGGC
GAGGATCTTCCACCACTCACCTACGAATCTCTCCAGAAACTTGATCTTCACAACAATGTAATCAAGGAGACTC
TCCGCCTCCACGCTCCCATCCACTCTATCCTCCGTGCTGTTAAATCCCCTATGCCCGTTGAAGGAACTAACTA
TGTTGTCCCAACCTCTCACAACCTCCTTGCCGCTCCTGGTGTTCCCTCACGAGACCCTCAGTACTTCCCTGAC
CCTCTTGTTTGGAACCCTCACCGATGGGAGAACAACGTCGGTGTCACCGTAGTCGAGGCCAGTGAAGAAAAGA
CAGATTACGGATACGGTTTGGTTAGCAAGGGTGCCAACAGCCCTTACCTCCCATTCGGCTCAGGCAGACACAG
ATGCATCGGTGAACAATTTGCATATGTTCAGCTTGGAACCGTAACAGCTACGTTAGCCAGACTAATGAAATGG
AAGCAAGTTGAAGGCACCAAAGATGTTGTGCCGCCAACTGACTATTCGGTAAGTTTGGCATATTACGCTCCTT
TTTATTTTGCTCGATTTTACTAACTTTGCATTAATAGTCCCTCTTCTCGAAGCCCCTTGGCGACCCAATGGTC
TCGTGGGAGAAGCGAAAGCAGCCTTCCCAGAAAT**GAT**AATCACGACTGCGGATTGAATCATGTTAAATTTCTT
GCTTCTTGCTAATTTATTTCTTCTCTTTTCTTACGTTTATACACCGAGCCATATTTGCACTTTATGGTCACGC
AAATAGGTTTTCGGAATAGAGCATATGTAATAAAAACTTTCTTTTGACCATGCTTTTGAAGATGGGGGATGTA
GATAATAGTATTATTCTAATACAGCCATTTATTACAATACTCTAGATGAACAGGAAAGTTGCAGTTACTCCGT
TAGCTTTGGTGCAGAAACAAAATGGTAATATACATTGGAAATAGTACATCTTCCCGTAGCTGGGATAATCTAG
CATCTAAACAGCGATATCCAAGCCAGTAATGTCAGGAAGTAACCAGCCAGCAACGTCGTTAATCCCGTGCCCC
CTTTCAACTCCGACGCTACAAAACGC**CTA**AGCAGCTGCCTGAGCATTCCTCCTCTCCGCCAACGCTCTCTTCG
CTCTGATCTCCTTCTGTCTTCTCTGACCACCGTCAATCTTTCTGCCACGCTTATGCTCCCACATGCCATCAGT
AACCTGAACCTTGACGGCGGTACTCCATGTGTTTGGAATAATCTGAGCATACGGCCCCTTGATCATATTCCCG
TGGTGCAATTTATAATGGGCAAACTTTTTGAGTGGCTTGGAAGTATCCTTGAGCTCTGTTTCGCCTGGTGGTA
GATTGACAATCGCATAACGCATTCCAGGTGTAAGGGTAGCGGTGCCCATTGTGCTAACATCTAGGGACTCTCC
GTCGGTGGCATTGGTAGTTTTTGCACTCTTTGACTTTTCGACGGGGACCTCAACAGCTCTCAGCTCAGCAACA
CCGTCAACAACGTTTTCGAAATCACCTCCTGGGCCGTAGTCCTGCTGTCTGAATTTCTCTCTCCACCGCAGTA
TGTATCGTCTGTCCCTTGTCATTTCTATGCCCAATTCTCGAAGCTCAGCCGAGTCCGCTGTAAACAATTGCTT
CCATCCGGTGATTTTGCTGGAATATCTGGACATGTTTCGCCCAATTAGGCGTAGGAATGTTTCGACATCGGGC
ACGAATGGGGTAGGTGATGGCACTGTTGCGGGTTTTCGAGATTGATGAAGGTACCGTACGCATGGCTGATAAT
GGGGGTTTGTGACGGAAGTGAAGAGCCGTAGTGGAAGGGATGTTCTGGCATTAAAGCCCGGTATGCGCGCCGC
AGC**CAT**AGTGGTTATATCGCCTTATATCAAACCGTTCAATGTCGAGATCTGGGGGGTGAAGATTTTAATTTAG

CTCGTGGTGTAAGAACACCATCGGCGGGCGTGACGAAAGCGAAAAAAGCCACGGCACCAAGGGCCCAGGTAAA
CCGGTAAGGGACTCAAGGCGAGTCAATCCAATGCTCAATGGCGCTGTATAGAAGATGTTCAACTGGTTCTTCG
ACTTCGTTCAAGATTTTGCACCAAAATTTTGAATTAGGTCCGACGGCCTTAGCTCGGATCAACAAAACGCCGA
TCTCCGGTGAGCTCGGGCCAGCCGAAGCTTCCATCGCAACATTCAAAGATGAAAGACGACGATATATATTTTG
ATAACAAACAAAGCTGTATTGTTGATTGAATACTAGGAGAGCTGCTCAGAGCAGTGTCTCACTCTTGTGCCAC
TCTATCCTTTGTCTCGCGAAATTGGTGGAAGCCAATTTCGCTTGACATTATGGCAGTGTCGCCGCTTCCCTCT
CAATTCCCTCCCATCACTACTACCGGTGCTCCAAAGTCGTTAGCGATGAGAAATATTGCTTCTCAAGAGCCTC
GGACGGCGCCTAGTGGTGATCCTGCAGTTCCTAAGCCCTCCATGCCCGAACCAGAGCTGGCGGAGCAGTTAAA
CGCTGATGTACGGCGGAAATATGTCAAAGGTGGGCGGGCAAATACACCCCAACCATTGGGACCCCATAATGCT
AACATTTGGTTATTCTGAAAGATAAGAAGCTCGGTGAAGGTACATATGCAGTTGTTTACCTAGGCCATCTACG
AGATGATCCCACTTCGCTAATAAAGAGCGACTTCGCATCTTTCGACAGAAAAAAAAGGTCGAGCAAGGGAAGT
TCGCTCAGCCTGGCCTGCCATTGACTCTAAGCGCCGTCGTTCTCTTGCTTGGGGGCTTGCTGCAGCTTGGCCT
GGGCTTGTCCGGCCTGGAACTCAGGGAGCCGAAAGATAACTAGTTATTTATTTCGTCTCCGCACACCTCGGCG
GCTGTCTCATAGTCGGGATATACATAGTACGGGGTAGCAGGTAGACTCACACGCTGCCTCTCTCTGCTGAACT
GCTTCTACCACCTTCGACAGCGACACTGTTCGGTCTTGCAACTACGGTAGAGTGTGTTGGGGCTTGATCTTGC
CTGCCATCGACTTGGAGTTGAAGTTGCGAAGCCATTGGCAGTCCATCCGGGATATTCGTAACTGCCGTCAACT
TCTTCACGCCGGGCCAAAAC**ATG**CCTCCGCAGAGGGAGAGAAGCAATCTGAAGAGAGAAAGGGTAAGATATGA
ACACGTTATATCCAATTAGTTTCTCAGAAGGATGAAAATATTCAAGCTGCTAACTTCTTTTCCCAAACCTGCA
GGGCAACGCAGCTCCAGACCATGAATCAAAGAAGCCACGGAGATCTCCTCGAATCTCATCTCAATTGCAGCAG
GATGCCGGATATCTACCTTCTCCCCTAGCTAATGAAGTAATCTCAGAAGAAAAGCGTGAAGTTGCTACAGTTT
CTCCCCCAGAAGGCCGCCGAAACCAGGCGCCTCGAGGTCAATCGCCAGACGAGTCGTCATGTCCCACCCAGGG
TTTCTCTCAGTATCCCCCAATCCAATCCATTGACGATGTGGAAGACGAGGTGGCCCAGGGCATATGGGGATAT
CTTACTCCGCTAGATAACAATTTTGGAGGCCAATTGGTTCTGAAGAAGCGGGACAGCACAAATACTACACCGG
TAATTGGAGCAGCATCAGACATATCAACGCCGGAAAGCAACGCCTCAAAACGTGGAACTAGAGAGATAAACCG
CGCTGGATATCTTATAGGCCGGCATGCAGAGTGCGGTATGCCTTTACCCATTGCATACTCTCTGCCGTCTAGT
GATCAATTGCTAACCATCTGGGTACTGCAGATTTCAGGATCAAGCTCCCCACGATATCCAACCGCCATTGCCT
GATATTCCACGAAAACAGGGAGGAGACTTTGTTGCAATTGTGGAAGATCTCTCTATCAATGGCACTTTCATCA
ACGACGCCATCATCGGCAAGAACAAACGCCGAGAGCTCGAAAACGGCGACGAGATTACCATCCTGCAAGAATC
CAGATTCATCTTCAACTACCCGCGGAGTCGGAACACAAGCAAGTTCCGACAGCAGTATAAGCTCTTTGATATG
CTGGGTAGGGGACACTTTGCCTCGGTTTATCTATGCGCTGAACGTTCTACGGGGATCAAGTATGCCGTCAAAC
ATTTCGAGAAACGCCCGGGCGCAGTTCAGAGGCTAGACCGTGAAGCACTACAGCAAGAGATTTCAATGCTGAT
GAGTGTCAACCATCCGAACATGCTGTGCCTAAAGGATACCTTTGACGAGAGCGATGGTGTCTTCCTGATTCTT
GAGCTTGCGCCAGAGGGCGAACTGTTCAACTGGATCATTAGGCATCAGAAACTCAGCGAGGAGGAGACTCGAA
AAGTATTCATTCAGCTTTTTGAAGGTTTGAGATACCTGGTATGTCGTTCGTTTTCTGGTTGATCAGACTCATG
GCAGTGTATATGGCTAACCGTTGTCGTAGCATGAACGAAACATCATTCACCGTGACATAAAGCCAGAGAACAT
TCTGGTTGTCGACAAAGATCTTACCGTAAAATTAGCAGACTTCGGCCTTGCAAAGATTGTCGGCGAACACTCC
TTTACCACCACGCTGTAAGTTCACCTACCTTCTGCCGTTCAAATCTGGGGTCAATCTTACTAAGAGATCATAA
AATCTAGATGTGGAACGCCTGGCTGTAAGTTTCCCTGCTGCTCTGTTAACGCTGGTGCTTTCATCCACCAAAT

GTGAATCTAACAATTCTTATCAGATGTTGCTCCTGAGATACTGGCAGAAAACGCTGAAGCCAGAATGTACACC
CGAGCGGTTGACATCTGGTCACTCGGAGTGGTACTCTACATCTGCCTGTGCGGATTCCCTCCTTTCTCAGATG
AACTGTACCACCCAAGAGACTACCCATACACTCAGAAAGAGCAGATCCAGCTCGGAATATTTAAGTATCCTCT
TCCATACTGGGACTCAATCGATTATAGAGCCATGGACCTTATCGACTCCATGATCGAAGTCGACGTGAAGGAG
CGTTTGAAAGTAGATGAGTGTCTCCAGCACCCATGGATCACTGGCATCGACCCCGATGAGCCTAGGGTGGCAG
ACAGCACCGATGACCTAGCCGGGGCCATCGGTGGCCTGAGGGTGTCCAAAGAACGCCGGCCAACTATGGTCCG
CAGGCTGTTGAGTGACATACATGAAGTCACGGTCAAGCAGGTGGTGAAGACCAAGGAAGACGACCCTGCTTCC
CAGGACATAACTATATACCAGAATCATCCTGTGCCTCGAGCCCAGGAGCCCAAACCGGCTGGCAACCGGAACT
CAAAGGAGTTCATGGAACTCGGTGGAAGTGGAGACCAGGTGTTATTCGATGAGCAGTCTGACGACTCGCTGGA
GCTTGAGAGTGAGGCACGGAAGCCAGTTGTCCACCAAGACGACGCAGACAAGACTCCACGAGAAAGAAAGAGG
TAGCAGAAGCCCGATCCGTCTTGGGATGGTATTCTGCTAGCTATACCGTGCTATTCCGGACTTTTGTTTCCAA
TTCCTTTTTCGTTTATATTGATTGTGATCTTTCTTAATTTTTTCTTTAACTATACCCCCCGGTTGAAAGGTGA
TAGGGGGCCAACTAACCGAAAAGCAGCAGAAAAACCGGCTTGGTCGCTGCCTTGGAGACGACGGCGATTCTCG
TGTTTAGGATTTCATTTTTGAGTGATATAACTATGGGTACAATAGCGTCCACTGGGCAATGTAATTACAGTCT
CTTCTCACTCACCACCGGACAATAATAACAATATTAATATTGACGGACCGTTGATAGCTTAACCTGCCATGAC
TAAGCCGGCCTCCCTGAAGCCGCGATGGCCTGATGATGCGCCCACCGTCAGGTCTCGCTCGCTCAGGCAGCAG
AGAATCCAGCGGAGGAGATGACGCCCTGCACGAACACCGTCCAGGGACCGTCTCCGCCCCGTCGCGAGCTCGA
ATTGTAATCATCTCGACGGCCGTTGGCCTGCTCGCCGCAAGGGAGAAAAAGGGGGGCAGAAGGGCAGCGGTAA
TTTTGGTATTGGAGATGCAGCTGTAAACCAATAAAAAACGACGTAAAATACCAGGAACAATGCATTATCTTAC
AGTACCGTACGCCCCTTGCATAAAATGCGAGTTCGATCTCAAGATGCAACTTTTTTTTTCCCTTGCTTCGTCC
GCAGCAGCTTCAAGGCATTCATCACAAACAACGAACAAGTCTTGAGCGGAAGCAGGAGGATAAGACGATCACC
ATGGGCCTCTTAGCCGACATTGTCTCTCGTTTCTGCGAGAACTGCTCGACCCTGTCCACCGCCGCGCTCGTCG
CAAGTGCCGTATCGGCTTTTATCGTCCTCTCCATTGTTATCAACGTCCTGCAGCAGCTCCTGTTCAAGGACCC
TACAAAGCCTCCGGTGGTCTTCCACTGGGTTCCGGTCATTGGAAGCACGATCTCCTATGGAATTGACCCGTAC
AAGTTCTTTGACGACTGCAAGGAGAAGGTTAGTTGGTGCAATTCAACTTAGCTATATACCAGGTTGCGCAAAG
CTAACGTATAAATAATTTCGGTACTCTAGTATGGAGACATCTTCACATTCATACTGCTGGGCAAGAAGACGAC
TGTTTTTCTCGGTACAAAGGGAAATGATTTCATTTTGAACGGCAAGCTCAAGGATGTTTGCGCGGAGGATGTC
TACTCCCCCCTCACCACCCCAGTGTTCGGACGACATGTGGTGTATGATTGCCCAAACTCCAAGCTCATGGAGC
AGAAGAAGGTGTGTACAGCAATTTATTCCCGTCTACATCTCCTGGGCCTCCATTGCTAATTTAATCGTCTACC
AGTTCGTCAAGTTCGGCCTCACCTCTGAAGCTCTCCGATCCTATGTCACCCTGATCACCAAGGAAGTTGAGCA
GTTCTTCGAGTCCTCCCCCGTCTTCAAGGGCGACTCCGGAGTTTTCAACGTCAGCAAGGTCATGGCTGAAATC
ACCATCTACACCGCCTCTCGATCTCTACAGGGCAAGGAGGTGCGAGGAAAGTTCGATTCCAGCTTTGCGGAAC
TCTACTCCGATCTCGACATGGGCTTCGCCGCCATCAACTTCATGTTCCCATGGTTCCCCTTCCCACACAACCG
CAAGCGTGACCGTGCTCAAAAGAAGATGGCCCAGGTTTACACCGACATCATCCGTCAGCGACGTGAGGCTGGT
GGAGAGAAAGACTCCGAGGACATGGTATGGAACTTGATGTCGTCCGTGTACAAGAATGGAACGCCAATTCCAG
ATATCGAAGTCGCCCACATGATGATTGCTCTTCTTATGGCTGGCCAGCACTCTTCTTCCTCCACCGGCTCCTG
GATCGTTCTCCGCCTTGCCAGCCGTCCAGATATTCTCGAGGAACTCTACGAGGAACAGAAACGTGTTCTCGGC
GAGGATCTTCCACCACTCACCTACGAATCTCTCCAGAAACTTGATCTTCACAACAATGTAATCAAGGAGACTC

TCCGCCTCCACGCTCCCATCCACTCTATCCTCCGTGCTGTTAAATCCCCTATGCCCGTTGAAGGAACTAACTA
TGTTGTCCCAACCTCTCACAACCTCCTTGCCGCTCCTGGTGTTCCCTCACGAGACCCTCAGTACTTCCCTGAC
CCTCTTGTTTGGAACCCTCACCGATGGGAGAACAACGTCGGTGTCACCGTAGTCGAGGCCAGTGAAGAAAAGA
CAGATTACGGATACGGTTTGGTTAGCAAGGGTGCCAACAGCCCTTACCTCCCATTCGGCTCAGGCAGACACAG
ATGCATCGGTGAACAATTTGCATATGTTCAGCTTGGAACCGTAACAGCTACGTTAGCCAGACTAATGAAATGG
AAGCAAGTTGAAGGCACCAAAGATGTTGTGCCGCCAACTGACTATTCGGTAAGTTTGGCATATTACGCTCCTT
TTTATTTTGCTCGATTTTACTAACTTTGCATTAATAGTCCCTCTTCTCGAAGCCCCTTGGCGACCCAATGGTC
TCGTGGGAGAAGCGAAAGCAGCCTTCCCAGAAA**TGA**TAATCACGACTGCGGATTGAATCATGTTAAATTTCTT
GCTTCTTGCTAATTTATTTCTTCTCTTTTCTTACGTTTATACACCGAGCCATATTTGCACTTTATGGTCACGC
AAATAGGTTTTCGGAATAGAGCATATGTAATAAAAACTTTCTTTTGACCATGCTTTTGAAGATGGGGGATGTA
GATAATAGTATTATTCTAATACAGCCATTTATTACAATACTCTAGATGAACAGGAAGTTGCAGTTACTCCGTT
AGCTTTGGTGCAGAAACAAAATGGTAATATACATTGGAAATAGTACATCTTCCCGTAGCTGGGATAATCTAGC
ATCTAAACAGCGATATCCAAGCCAGTAATGTCAGGAAGTAACCAGCCAGCAACGTCGTTAATCCCGTGCCCCC
TTTCAACTCCGACGCTACAAAACGC**CTA**AGCAGCTGCCTGAGCATTCCTCCTCTCCGCCAACGCTCTCTTCGC
TCTGATCTCCTTCTGTCTTCTCTGACCACCGTCAATCTTTCTGCCACGCTTATGCTCCCACATGCCATCAGTA
ACCTGAACCTTGACGGCGGTACTCCATGTGTTTGGAATAATCTGAGCATACGGCCCCTTGATCATATTCCCGT
GGTGCAATTTATAATGGGCAAACTTTTTGAGTGGCTTGGAAGTATCCTTGAGCTCTGTTTCGCCTGGTGGTAG
ATTGACAATCGCATAACGCATTCAGGTGTAAGGGTAGCGGTGCCCATTGTGCTAACATCTAGGGACTCTCCGT
CGGTGGCATTGGTAGTTTTTGCACTCTTTGACTTTTCGACGGGGACCTCAACAGCTCTCAGCTCAGCAACACC
GTCAACAACGTTTTCGAAATCACCTCCTGGGCCGTAGTCCTGCTGTCTGAATTTCTCTCTCCACCGCAGTATG
TATCGTCTGTCCCTTGTCATTTCTATGCCCAATTCTCGAAGCTCAGCCGAGTCCGCTGTAAACAATTGCTTCC
ATCCGGTGATTTTGCTGGAATATCTGGACATGTTTCGCCCAATTAGGCGTAGGAATGTTTCGACATCGGGCAC
GAATGGGGTAGGTGATGGCACTGTTGCGGGTTTTCGAGATTGATGAAGGTACCGTACGCATGGCTGATAATGG
GGTTTGTGACGGAAGTGAAGAGCCGTAGTGGAAGGGATGTTCTGGCATTAAAGCCCGGTATGCGCGCCGCAGC
CATAGTGGTTATATCGCCTTATATCAAACCGTTCAATGTCGAGATCTGGGGGGTGAAGATTTTAATTTAGCTC
GTGGTGTAAGAACACCATCGGCGGGCGTGACGAAAGCGAAAAAAGCCACGGCACCAAGGGCCCAGGTAAACCG
GTAAGGGACTCAAGGCGAGTCAATCCAATGCTCAATGGCGCTGTATAGAAGATGTTCAACTGGTTCTTCGACT
TCGTTCAAGATTTTGCACCAAAATTTTGAATTAGGTCCGACGGCCTTAGCTCGGATCAACAAAACGCCGATCT
CCGGTGAGCTCGGGCCAGCCGAAGCTTCCATCGCAACATTCAAAGAT

[Read No.m64281e_230311_112734/178194669/ccs] [Antisense (-) strand]

GGT~ATT (5’UTR)

CTA~CAT (*chkB* protein kinase genomic DNA) (Reverse sequence)

TCA~CAT (*TinCYP51B* gene) (Reverse sequence)

AGC~TTA (3’UTR)

ATG~TAG (*FYV4* genomic DNA) (Forward sequence)

ACCTGCTACCCCGTACTATGTATATCCCGACTATGAGACAGCCGCCGAGGTGTGCGGAGACGAAATAAATAAC
TAGTTATCTTTCGGCTCCCTGAGTTCCAGGCCGGACAAGCCCAGGCCAAGCTGCAGCAAGCCCCCAAGCAAGA
GAACGACGGCGCTTAGAGTCAATGGCAGGCCAGGCTGAGCGAACTTCCCTTGCTCGACCTTTTTTTTCTGTCG
AAAGATGCGAAGTCGCTCTTTATTAGCGAAGTGGGATCATCTCGTAGATGGCCTAGGTAAACAACTGCATATG
TACCTTCACCGAGCTTCTTATCTTTCAGAATAACCAAATGTTAGCATTATGGGGTCCCAATGGTTGGGGTGTA
TTTGCCCGCCCACCTTTGACATATTTCCGCCGTACATCAGCGTTTAACTGCTCCGCCAGCTCTGGTTCGGGCA
TGGAGGGCTTAGGAACTGCAGGATCACCACTAGGCGCCGTCCGAGGCTCTTGAGAAGCAATATTTCTCATCGC
TAACGACTTTGGAGCACCGGTAGTAGTGATGGGAGGGAATTGAGAGGGAAGCGGCGACACTGCCATAATGTCA
AGCGAAATTGGCTTCCACCAATTTCGCGAGACAAAGGATAGAGTGGCACAAGAGTGAGACACTGCTCTGAGCA
GCTCTCCTAGTATTCAATCAACAATACAGCTTTGTTTGTTATCAAAATATATATCGTCGTCTTTCATCTTTGA
ATGTTGCGATGGAAGCTTCGGCTGGCCCGAGCTCACCGGAGATCGGCGTTTTGTTGATCCGAGCTAAGGCCGT
CGGACCTAATTCAAAATTTTGGTGCAAAATCTTGAACGAAGTCGAAGAACCAGTTGAACATCTTCTATACAGC
GCCATTGAGCATTGGATTGACTCGCCTTGAGTCCCTTACCGGTTTACCTGGGCCCTTGGTGCCGTGGCTTTTT
TCGCTTTCGTCACGCCCGCCGATGGTGTTCTTACACCACGAGCTAAATTAAAATCTTCACCCCCCAGATCTCG
ACATTGAACGGTTTGATATAAGGCGATATAACCACT**ATG**GCTGCGGCGCGCATACCGGGCTTTAATGCCAGAA
CATCCCTTCCACTACGGCTCTTCACTTCCGTCACAAACCCCCATTATCAGCCATGCGTACGGTACCTTCATCA
ATCTCGAAAACCCCGCAACAGTGCCATCACCTACCCCATTCGTGCCCGATGTCGAAACATTCCTACGCCTAAT
TGGGCGAAACATGTCCAGATATTCCAGCAAAATCACCGATGGAAGCAATTGTTTACAGCGGACTCGGCTGAGC
TTCGAGAATTGGGCATAGAAATGACAAGGGACAGACGATACATACTGCGGTGGAGAGAGAAATTCAGACAGCA
GGACTACGGCCCAGGAGGTGATTTCGAAAACGTTGTTGACGGTGTTGCTGAGCTGAGAGCTGTTGAGGTCCCC
GTCGAAAAGTCAAAGAGTGCAAAAACTACCAATGCCACCGACGGAGAGTCCCTAGATGTTAGCACAATGGGCA
CCGCTACCCTTACACCTGGAATGCGTTATGCGATTGTCAATCTACCACCAGGCGAAACAGAGCTCAAGGATAC
TTCCAAGCCACTCAAAAAGTTTGCCCATTATAAATTGCACCACGGGAATATGATCAAGGGGCCGTATGCTCAG
ATTATTCCAAACACATGGAGTACCGCCGTCAAGGTTCAGGTTACTGATGGCATGTGGGAGCATAAGCGTGGCA
GAAAGATTGACGGTGGTCAGAGAAGACAGAAGGAGATCAGAGCGAAGAGAGCGTTGGCGGAGAGGAGGAATGC
TCAGGCAGCTGCT**TAG**GCGTTTTGTAGCGTCGGAGTTGAAAGGGGGCACGGGATTAACGACGTTGCTGGCTGG
TTACTTCCTGACATTACTGGCTTGGATATCGCTGTTTAGATGCTAGATTATCCCAGCTACGGGAAGATGTACT
ATTTCCAATGTATATTACCATTTTGTTTCTGCACCAAAGCTAACGGAGTAACTGCAACTTTCCTGTTCATCTA
GAGTATTGTAATAAATGGCTGTATTAGAATAATACTATTATCTACATCCCCCATCTTCAAAAGCATGGTCAAA
AGAAAGTTTTTATTACATATGCTCTATTCCGAAAACCTATTTGCGTGACCATAAAGTGCAAATATGGCTCGGT
GTATAAACGTAAGAAAAGAGAAGAAATAAATTAGCAAGAAGCAAGAAATTTAACATGATTCAATCCGCAGTCG
TGATTA**TCA**TTTCTGGGAAGGCTGCTTTCGCTTCTCCCACGAGACCATTGGGTCGCCAAGGGGCTTCGAGAAG
AGGGACTATTAATGCAAAGTTAGTAAAATCGAGCAAAATAAAAAGGAGCGTAATATGCCAAACTTACCGAATA
GTCAGTTGGCGGCACAACATCTTTGGTGCCTTCAACTTGCTTCCATTTCATTAGTCTGGCTAACGTAGCTGTT
ACGGTTCCAAGCTGAACATATGCAAATTGTTCACCGATGCATCTGTGTCTGCCTGAGCCGAATGGGAGGTAAG
GGCTGTTGGCACCCTTGCTAACCAAACCGTATCCGTAATCTGTCTTTTCTTCACTGGCCTCGACTACGGTGAC
ACCGACGTTGTTCTCCCATCGGTGAGGGTTCCAAACAAGAGGGTCAGGGAAGTACTGAGGGTCTCGTGAGGGA

ACACCAGGAGCGGCAAGGAGGTTGTGAGAGGTTGGGACAACATAGTTAGTTCCTTCAACGGGCATAGGGGATT
TAACAGCACGGAGGATAGAGTGGATGGGAGCGTGGAGGCGGAGAGTCTCCTTGATTACATTGTTGTGAAGATC
AAGTTTCTGGAGAGATTCGTAGGTGAGTGGTGGAAGATCCTCGCCGAGAACACGTTTCTGTTCCTCGTAGAGT
TCCTCGAGAATATCTGGACGGCTGGCAAGGCGGAGAACGATCCAGGAGCCGGTGGAGGAAGAAGAGTGCTGGC
CAGCCATAAGAAGAGCAATCATCATGTGGGCGACTTCGATATCTGGAATTGGCGTTCCATTCTTGTACACGGA
CGACATCAAGTTCCATACCATGTCCTCGGAGTCTTTCTCTCCACCAGCCTCACGTCGCTGACGGATGATGTCG
GTGTAAACCTGGGCCATCTTCTTTTGAGCACGGTCACGCTTGCGGTTGTGTGGGAAGGGGAACCATGGGAACA
TGAAGTTGATGGCGGCGAAGCCCATGTCGAGATCGGAGTAGAGTTCCGCAAAGCTGGAATCGAACTTTCCTCG
CACCTCCTTGCCCTGTAGAGATCGAGAGGCGGTGTAGATGGTGATTTCAGCCATGACCTTGCTGACGTTGAAA
ACTCCGGAGTCGCCCTTGAAGACGGGGGAGGACTCGAAGAACTGCTCAACTTCCTTGGTGATCAGGGTGACAT
AGGATCGGAGAGCTTCAGAGGTGAGGCCGAACTTGACGAACTGGTAGACGATTAAATTAGCAATGGAGGCCCA
GGAGATGTAGACGGGAATAAATTGCTGTACACACCTTCTTCTGCTCCATGAGCTTGGAGTTTGGGCAATCATA
CACCACATGTCGTCCGAACACTGGGGTGGTGAGGGGGGAGTAGACATCCTCCGCGCAAACATCCTTGAGCTTG
CCGTTCAAAATGAAATCATTTCCCTTTGTACCGAGAAAAACAGTCGTCTTCTTGCCCAGCAGTATGAATGTGA
AGATGTCTCCATACTAGAGTACCGAAATTATTTATACGTTAGCTTTGCGCAACCTGGTATATAGCTAAGTTGA
ATTGCACCAACTAACCTTCTCCTTGCAGTCGTCAAAGAACTTGTACGGGTCAATTCCATAGGAGATCGTGCTT
CCAATGACCGGAACCCAGTGGAAGACCACCGGAGGCTTTGTAGGGTCCTTGAACAGGAGCTGCTGCAGGACGT
TGATAACAATGGAGAGGACGATAAAAGCCGATACGGCACTTGCGACGAGCGCGGCGGTGGACAGGGTCGAGCA
GTTCTCGCAGAAACGAGAGACAATGTCGGCTAAGAGGCC**CAT**GGTGATCGTCTTATCCTCCTGCTTCCGCTCA
AGACTTGTTCGTTGTTTGTGATGAATGCCTTGAAGCTGCTGCGGACGAAGCAAGGGAAAAAAAAAGTTGCATC
TTGAGATCGAACTCGCATTTTATGCAAGGGGGCGTACGGTACTGTAAGATAATGCATTGTTCCTGGTATTTTA
CGTCGTTTTTTATTGGTTTACAGCTGCATCTCCAATACCAAAATTACCGCTGCCCTTCTGCCCCCCTTTTTCT
CCCTTGCGGCGAGCAGGCCAACGGCCGTCGAGATGATTACAATTCGAGCTCGCGACGGGGCGGAGACGGTCCC
TGGACGGTGTTCGTGCAGGGCGTCATCTCCTCCGCTGGATTCTCTGCTGCCTGAGCGAGCGAGACCTGACGGT
GGGCGCATCATCAGGCCATCGCGGCTTCAGGGAGGCCGGCTTAGTCATGGCAGGTTAAGCTATCAACGGTCCG
TCAATATTAATATTGTTATTATTGTCCGGTGGTGAGTGAGAAGAGACTGTAATTACATTGCCCAGTGGACGCT
ATTGTACCCATAGTTATATCACTCAAAAATGAAATCCTAAACACGAGAATCGCCGTCGTCTCCAAGGCAGCGA
CCAAGCCGGTTTTTCTGCTGCTTTTCGGTTAGTTGGCCCCCTATCACCTTTCAACCGGGGGGTATAGTTAAAG
AAAAAATTAAGAAAGATCACAATCAATATAAACGAAAAAGGAATTGGAAACAAAAGTCCGGAATAGCACGGTA
TAGCTAGCAGAATACCATCCCAAGACGGATCGGGCTTCTG**CTA**CCTCTTTCTTTCTCGTGGAGTCTTGTCTGC
GTCGTCTTGGTGGACAACTGGCTTCCGTGCCTCACTCTCAAGCTCCAGCGAGTCGTCAGACTGCTCATCGAAT
AACACCTGGTCTCCACTTCCACCGAGTTCCATGAACTCCTTTGAGTTCCGGTTGCCAGCCGGTTTGGGCTCCT
GGGCTCGAGGCACAGGATGATTCTGGTATATAGTTATGTCCTGGGAAGCAGGGTCGTCTTCCTTGGTCTTCAC
CACCTGCTTGACCGTGACTTCATGTATGTCACTCAACAGCCTGCGGACCATAGTTGGCCGGCGTTCTTTGGAC
ACCCTCAGGCCACCGATGGCCCCGGCTAGGTCATCGGTGCTGTCTGCCACCCTAGGCTCATCGGGGTCGATGC
AGTGATCCATGGGTGCTGGAGACACTCATCTACTTTCAAACGCTCCTTCACGTCGACTTCGATCATGGAGTCG
ATAAGGTCCATGGCTCTATAATCGATTGTGTAGTGGAGTCCCAGTATGGAAGAGGATACTTAAATATTCCGAG

CTGGATCTGCTCTTTCTGAGTGTATGGGTAGTCTCTTGGGTGGTACAGTTCATCTGAGAAAGGAGGGAATCCG
CACAGGCAGATGTAGAGTACCACTCCGAGTGACCAGATGTCAACCGCTCGGGTGTACATTCTGGCTTCAGCGT
TTTCTGCCAGTATCTCAGGAGCAACATCTGATAAGAATTGTTAGATTCACATTTGGTGGATGAAAGCACCAGC
GTTAACAGAGCAGCAGGGAAACTTACAGCCAGGCGTTCCACATCTAGATTTTATGATCTCTTAGTAAGATTGA
CCCCAGATTTGAACGGCAGAAGGTAGGTGAACTTACAGCGTGGTGGTAAAGGAGTGTTCGCCGACAATCTTTG
CAAGGCCGAAGTCTGCTAATTTTACGGTAAGATCTTTGTCGACAACCAGAATGTTCTCTGGCTTTATGTCACG
GTGAATGATGTTTCGTTCATGCTACGACAACGGTTAGCCATATACACTGCCATGAGTCTGATCAACCAGAAAA
CGAACGACATACCAGGTATCTCAAACCTTCAAAAAGCTGAATGAATACTTTTCGAGTCTCCTCCTCGCTGAGT
TTCTGATGCCTAATGATCCAGTTGAACAGTTCGCCCTCTGGCGCAAGCTCAAGAATCAGGAAGACACCATCGC
TCTCGTCAAAGGTATCCTTTAGGCACAGCATGTTCGGATGGTTGACACTCATCAGCATTGAAATCTCTTGCTG
TAGTGCTTCACGGTCTAGCCTCTGAACTGCGCCCGGGCGTTTCTCGAAATGTTTGACGGCATACTTGATCCCC
GTAGAACGTTCAGCGCATAGATAAACCGAGGCAAAGTGTCCCCTACCCAGCATATCAAAGAGCTTATACTGCT
GTCGGAACTTGCTTGTGTTCCGACTCCGCGGGTAGTTGAAGATGAATCTGGATTCTTGCAGGATGGTAATCTC
GTCGCCGTTTTCGAGCTCTCGGCGTTTGTTCTTGCCGATGATGGCGTCGTTGATGAAAGTGCCATTGATAGAG
AGATCTTCCACAATTGCAACAAAGTCTCCTCCCCTGTTTTCGTGGAATATCAGGCAATGGCGGTTGGATATCG
TGGGGAGCTTGATCCTGAAATCTGCAGTACCCAGATGGTTAGCAATTGATCACTAGACGGCAGAGAGTATGCA
ATGGGTAAAGGCATACCGCACTCTGCATGCCGGCCTATAAGATATCCAGCGCGGTTTATCTCTCTAGTTCCAC
GTTTTGAGGCGTTGCTTTCCGGCGTTGATATGTCTGATGCTGCTCCAATTACCGGTGTAGTATTTGTGCTGTC
CCGCTTCTTCAGAACCAATTGGCCTCCAAATTGTTATCTAGCGGAGTAAGATATCCCCATATGCCCTGGGCCA
CCTCGTCTTCCACATCGTCAATGGATTGGATTGGGGGATACTGAGAGAAACCCTGGGTGGGACATGACGACTC
GTCTGGCGATTGACCTCGAGGCGCCTGGTTTCGGCGGCCTTCTGGGGGAGAAACTGTAGCAACTTCACGCTTT
TCTTCTGAGATTACTTCATTAGCTAGGGGAGAAGGTAGATATCCGGCATCCTGCTGCAATTGAGATGAGATTC
GAGGAGATCTCCGTGGCTTCTTTGATTCATGGTCTGGAGCTGCGTTGCCCTGCAGGTTTGGGAAAAGAAGTTA
GCAGCTTGAATATTTTCATCCTTCTGAGAAACTAATTGGATATAACGTGTTCATATCTTACCCTTTCTCTCTT
CAGATTGCTTCTCTCCCTCTGCGGAGG**CAT**GTTTTGGCCCGGCGTGAAGAAGTTGACGGCAGTTACGAATATC
CCGGATGGACTGCCAATGGCTTCGCAACTTCAACTCCAAGTCGATGGCAGGCAAGATCAAGCCCCAACACACT
CTACCGTAGTTGCAAGACCGAACAGTGTCGCTGTCGAAGGTGGTAGAAGCAGTTCAGCAGAGAGAGGCAGCGT
GTGAGTCTACCTGCTACCCCGTACTATGTATATCCCGACTATGAGACAGCCGCCGAGGTGTGCGGAGACGAAA
TAAATAACTAGTTATCTTTCGGCTCCCTGAGTTCCAGGCCGGACAAGCCCAGGCCAAGCTGCAGCAAGCCCCC
AAGCAAGAGAACGACGGCGCTTAGAGTCAATGGCAGGCCAGGCTGAGCGAACTTCCCTTGCTCGACCTTTTTT
TTCTGTCGAAAGATGCGAAGTCGCTCTTTATTAGCGAAGTGGATCATCTCGTAGATGGCCTAGGTAAACAACT
GCATATGTACCTTCACCGAGCTTCTTATCTTTCAGAATAACCAAATGTTAGCATTATGGGGTCCCAATGGTTG
GGGTGTATTTGCCCGCCCACCTTTGACATATTTCCGCCGTACATCAGCGTTTAACTGCTCCGCCAGCTCTGGT
TCGGGCATGGAGGGCTTAGGAACTGCAGGATCACCACTAGGCGCCGTCCGAGGCTCTTGAGAAGCAATATTTC
TCATCGCTAACGACTTTGGAGCACCGGTAGTAGTGATGGGAGGGAATTGAGAGGGAAGCGGCGACACTGCCAT
AATGTCAAGCGAAATTGGCTTCCACCAATTTCGCGAGACAAAGGATAGAGTGGCACAAGAGTGAGACACTGCT
CTGAGCAGCTCTCCTAGTATTCAATCAACAATACAGCTTTGTTTGTTATCAAAATATATATCGTCGTCTTTCA

TCTTTGAATGTTGCGATGGAAGCTTCGGCTGGCCCGAGCTCACCGGAGATCGGCGTTTTGTTGATCCGAGCTA
AGGCCGTCGGACCTAATTCAAAATTTTGGTGCAAAATCTTGAACGAAGTCGAAGAACCAGTTGAACATCTTCT
ATACAGCGCCATTGAGCATTGGATTGACTCGCCTTGAGTCCCTTACCGGTTTACCTGGGCCCTTGGTGCCGTG
GCTTTTTTCGCTTTCGTCACGCCCGCCGATGGTGTTCTTACACCACGAGCTAAATTAAAATCTTCACCCCCCA
GATCTCGACATTGAACGGTTTGATATAAGGCGATATAACCACT**ATG**GCTGCGGCGCGCATACCGGGCTTTAAT
GCCAGAACATCCCTTCCACTACGGCTCTTCACTTCCGTCACAAACCCCCATTATCAGCCATGCGTACGGTACC
TTCATCAATCTCGAAAACCCGCAACAGTGCCATCACCTACCCCATTCGTGCCCGATGTCGAAACATTCCTACG
CCTAATTGGGCGAAACATGTCCAGATATTCCAGCAAAATCACCGGATGGAAGCAATTGTTTACAGCGGACTCG
GCTGAGCTTCGAGAATTGGGCATAGAAATGACAAGGGACAGACGATACATACTGCGGTGGAGAGAGAAATTCA
GACAGCAGGACTACGGCCCAGGAGGTGATTTCGAAAACGTTGTTGACGGTGTTGCTGAGCTGAGAGCTGTTGA
GGTCCCCGTCGAAAAGTCAAAGAGTGCAAAAACTACCAATGCCACCGACGGAGAGTCCCTAGATGTTAGCACA
ATGGGCACCGCTACCCTTACACCTGGAATGCGTTATGCGATTGTCAATCTACCACCAGGCGAAACAGAGCTCA
AGGATACTTCCAAGCCACTCAAAAAGTTTGCCCATTATAAATTGCACCACGGGAATATGATCAAGGGGCCGTA
TGCTCAGATTATTCCAAACACATGGAGTACCGCCGTCAAGGTTCAGGTTACTGATGGCATGTGGGAGCATAAG
CGTGGCAGAAAGATTGACGGTGGTCAGAGAAGACAGAAGGAGATCAGAGCGAAGAGAGCGTTGGCGGAGAGGA
GGAATGCTCAGGCAGCTGCT**TAG**GCGTTTTGTAGCGTCGGAGTTGAAAGGGGGCACGGGATTAACGACGTTGC
TGGCTGGTTACTTCCTGACATTACTGGCTTGGATATCGCTGTTTAGATGCTAGATTATCCCAGCTACGGGAAG
ATGTACTATTTCCAATGTATATTACCATTTTGTTTCTGCACCAAAGCTAACGGAGTAACTGCAACTTTCCTGT
TCATCTAGAGTATTGTAATAAATGGCTGTATTAGAATAATACTATTATCTACATCCCCCATCTTCAAAAGCAT
GGTCAAAAGAAAGTTTTTATTACATATGCTCTATTCCGAAAACCTATTTGCGTGACCATAAAGTGCAAATATG
GCTCGGTGTATAAACGTAAGAAAAGAGAAGAAATAAATTAGCAAGAAGCAAGAAATTTAACATGATTCAATCC
GCAGTCGTGATTA**TCA**TTTCTGGGAAGGCTGCTTTCGCTTCTCCCACGAGACCATTGGGTCGCCAAGGGGCTT
CGAGAAGAGGGACTATTAATGCAAAGTTAGTAAAATCGAGCAAAATAAAAAGGAGCGTAATATGCCAAACTTA
CCGAATAGTCAGTTGGCGGCACAACATCTTTGGTGCCTTCAACTTGCTTCCATTTCATTAGTCTGGCTAACGT
AGCTGTTACGGTTCCAAGCTGAACATATGCAAATTGTTCACCGATGCATCTGTGTCTGCCTGAGCCGAATGGG
AGGTAAGGGCTGTTGGCACCCTTGCTAACCAAACCGTATCCGTAATCTGTCTTTTCTTCACTGGCCTCGACTA
CGGTGACACCGACGTTGTTCTCCCATCGGTGAGGGTTCCAAACAAGAGGGTCAGGGAAGTACTGAGGGTCTCG
TGAGGGAACACCAGGAGCGGCAAGGAGGTTGTGAGAGGTTGGGACAACATAGTTAGTTCCTTCAACGGGCATA
GGGGATTTAACAGCACGGAGGATAGAGTGGATGGGAGCGTGGAGGCGGAGAGTCTCCTTGATTACATTGTTGT
GAAGATCAAGTTTCTGGAGAGATTCGTAGGTGAGTGGTGGAAGATCCTCGCCGAGAACACGTTTCTGTTCCTC
GTAGAGTTCCTCGAGAATATCTGGACGGCTGGCAAGGCGGAGAACGATCCAGGAGCCGGTGGAGGAAGAAGAG
TGCTGGCCAGCCATAAGAAGAGCAATCATCATGTGGGCGACTTCGATATCTGGAATTGGCGTTCCATTCTTGT
ACACGGACGACATCAAGTTCCATACCATGTCCTCGGAGTCTTTCTCTCCACCAGCCTCACGTCGCTGACGGAT
GATGTCGGTGTAAACCTGGGCCATCTTCTTTTGAGCACGGTCACGCTTGCGGTTGTGTGGGAAGGGGAACCAT
GGGAACATGAAGTTGATGGCGGCGAAGCCCATGTCGAGATCGGAGTAGAGTTCCGCAAAGCTGGAATCGAACT
TTCCTCGCACCTCCTTGCCCTGTAGAGATCGAGAGGCGGTGTAGATGGTGATTTCAGCCATGACCTTGCTGAC
GTTGAAAACTCCGGAGTCGCCCTTGAAGACGGGGGAGGACTCGAAGAACTGCTCAACTTCCTTGGTGATCAGG

GTGACATAGGATCGGAGAGCTTCAGAGGTGAGGCCGAACTTGACGAACTGGTAGACGATTAAATTAGCAATGG
AGGCCCAGGAGATGTAGACGGGAATAAATTGCTGTACACACCTTCTTCTGCTCCATGAGCTTGGAGTTTGGGC
AATCATACACCACATGTCGTCCGAACACTGGGGTGGTGAGGGGGGAGTAGACATCCTCCGCGCAAACATCCTT
GAGCTTGCCGTTCAAAATGAAATCATTTCCCTTTGTACCGAGAAAAACAGTCGTCTTCTTGCCCAGCAGTATG
AATGTGAAGATGTCTCCATACTAGAGTACCGAAATTATTTATACGTTAGCTTTGCGCAACCTGGTATATAGCT
AAGTTGAATTGCACCAACTAACCTTCTCCTTGCAGTCGTCAAAGAACTTGTACGGGTCAATTCCATAGGAGAT
CGTGCTTCCAATGACCGGAACCCAGTGGAAGACCACCGGAGGCTTTGTAGGGTCCTTGAACAGGAGCTGCTGC
AGGACGTTGATAACAATGGAGAGGACGATAAAAGCCGATACGGCACTTGCGACGAGCGCGGCGGTGGACAGGG
TCGAGCAGTTCTCGCAGAAACGAGAGACAATGTCGGCTAAGAGGCC**CAT**GGTGATCGTCTTATCCTCCTGCTT
CCGCTCAAGACTTGTTCGTTGTTTGTGATGAATGCCTTGAAGCTGCTGCGGACGAAGCAAGGGAAAAAAAAAG
TTGCATCTTGAGATCGAACTCGCATTTTATGCAAGGGGCGTACGGTACTGTAAGATAATGCATTGTTCCTGGT
ATTTTACGTCGTTTTTATTGGTTTACAGCTGCATCTCCAATACCAAAATTACCGCTGCCCTTCTGCCCCCCT
TTTTCTCCCTTGCGGCGAGCAGGCCAACGGCCGTCGAGATGATTACAATTCGAGCTCGCGACGGGGCGGAGAC
GGTCCCTGGACGGTGTTCGTGCAGGGCGTCATCTCCTCCGCTGGATTCTCTGCTGCCTGAGCGAGCGAGACCT
GACGGTGGGCGCATCATCAGGCCATCGCGGCTTCAGGGAGGCCGGCTTAGTCATGGCAGGTTAAGCTATCAAC
GGTCCGTCAATATTAATATTGTTATTATTGTCCGGTGGTGAGTGAGAAGAGACTGTAATTACATTGCCCAGTG
GACGCTATTGTACCCATAGTTATATCACTCAAAAATGAAATCCTAAACACGAGAATCGCCGTCGTCTCCAAGG
CAGCGACCAAGCCGGTTTTTCTGCTGCTTTTCGGTTAGTTGGCCCCCTATCACCTTTCAACCGGGGGGTATAG
TTAAAGAAAAAATTAAGAAAGATCACAATCAATATAAACGAAAAAGGAATTGGAAACAAAAGTCCGGAATAGC
ACGGTATAGCTAGCAGAATACCATCCCAAGACGGATCGGGCTTCTG**CTA**CCTCTTTCTTTCTCGTGGAGTCTT
GTCTGCGTCGTCTTGGTGGACAACTGGCTTCCGTGCCTCACTCTCAAGCTCCAGCGAGTCGTCAGACTGCTCA
TCGAATAACACCTGGTCTCCACTTCCACCGAGTTCCATGAACTCCTTTGAGTTCCGGTTGCCAGCCGGTTTGG
GCTCCTGGGCTCGAGGCACAGGATGATTCTGGTATATAGTTATGTCCTGGGAAGCAGGGTCGTCTTCCTTGGT
CTTCACCACCTGCTTGACCGTGACTTCATGTATGTCACTCAACAGCCTGCGGACCATAGTTGGCCGGCGTTCT
TTGGACACCCTCAGGCCACCGATGGCCCCGGCTAGGTCATCGGTGCTGTCTGCCACCCTAGGCTCATCGGGGT
CGATGCCAGTGATCCATGGGTGCTGGAGACACTCATCTACTTTCAAACGCTCCTTCACGTCGACTTCGATCAT
GGAGTCGATAAGGTCCATGGCTCTATAATCGATTGAGTCCCAGTATGGAAGAGGATACTTAAATATTCCGAGC
TGGATCTGCTCTTTCTGAGTGTATGGGTAGTCTCTTGGGTGGTACAGTTCATCTGAGAAAGGAGGGAATCCGC
ACAGGCAGATGTAGAGTACCACTCCGAGTGACCAGATGTCAACCGCTCGGGTGTACATTCTGGCTTCAGCGTT
TTCTGCCAGTATCTCAGGAGCAACATCTGATAAGAATTGTTAGATTCACATTTGGTGGATGAAAGCACCAGCG
TTAACAGAGCAGCAGGGAAACTTACAGCCAGGCGTTCCACATCTAGATTTTATGATCTCTTAGTAAGATTGAC
CCCAGATTTGAACGGCAGAAGGTAGGTGAACTTACAGCGTGGTGGTAAAGGAGTGTTCGCCGACAATCTTTGC
AAGGCCGAAGTCTGCTAATTTTACGGTAAGATCTTTGTCGACAACCAGAATGTTCTCTGGCTTTATGTCACGG
TGAATGATGTTTCGTTCATGCTACGACAACGGTTAGCCATATACACTGCCATGAGTCTGATCAACCAGAAAAC
GAACGACATACCAGGTATCTCAAACCTTCAAAAAGCTGAATGAATACTTTTCGAGTCTCCTCCTCGCTGAGTT
TCTGATGCCTAATGATCCAGTTGAACAGTTCGCCCTCTGGCGCAAGCTCAAGAATCAGGAAGACACCATCGCT
CTCGTCAAAGGTATCCTTTAGGCACAGCATGTTCGGATGGTTGACACTCATCAGCATTGAAATCTCTTGCTGT

AGTGCTTCACGGTCTAGCCTCTGAACTGCGCCCGGGCGTTTCTCGAAATGTTTGACGGCATACTTGATCCCCG
TAGAACGTTCAGCGCATAGATAAACCGAGGCAAAGTGTCCCCTACCCAGCATATCAAAGAGCTTATACTGCTG
TCGGAACTTGCTTGTGTTCCGACTCCGCGGGTAGTTGAAGATGAATCTGGATTCTTGCAGGATGGTAATCTCG
TCGCCGTTTTCGAGCTCTCGGCGTTTGTTCTTGCCGATGATGGCGTCGTTGATGAAAGTGCCATTGATAGAGA
GATCTTCCACAATTGCAACAAAGTCTCCTCCCCTGTTTTCGTGGAATATCAGGCAATGGCGGTTGGATATCGT
GGGGAGCTTGATCCTGAAATCTGCAGTACCCAGATGGTTAGCAATTGATCACTAGACGGCAGAGAGTATGCAA
TGGGTAAAGGCATACCGCACTCTGCATGCCGGCCTATAAGATATCCAGCGCGGTTTATCTCTCTAGTTCCACG
TTTTGAGGCGTTGCTTTCCGGCGTTGATATGTCTGATGCTGCTCCAATTACCGGTGTAGTATTTGTGCTGTCC
CGCTTCTTCAGAACCAATTGGCCTCCAAAATTGTTATCTAGCGGAGTAAGATATCCCCATATGCCCTGGGCCA
CCTCGTCTTCCACATCGTCAATGGATTGGATTGGGGGATACTGAGAGAAACCCTGGGTGGGACATGACGACTC
GTCTGGCGATTGACCTCGAGGCGCCTGGTTTCGGCGGCCTTCTGGGGGAGAAACTGTAGCAACTTCACGCTTT
TCTTCTGAGATTACTTCATTAGCTAGGGGAGAAGGTAGATATCCGGCATCCTGCTGCAATTGAGATGAGATTC
GAGGAGATCTCCGTGGCTTCTTTGATTCATGGTCTGGAGCTGCGTTGCCCTGCAGGTTTGGGAAAAGAAGTTA
GCAGCTTGAATATTTTCATCCTTCTGAGAAACTAATTGGATATAACGTGTTCATATCTTACCCTTTCTCTCTT
CAGATTGCTTCTCTCCCTCTGCGGAGG**CAT**GTTTTGGCCCGGCGTGAAGAAGTTGACGGCAGTTACGAATATC
CCGGATGGACTGCCAATGGCTTCGCAACTTCAACTCCAAGTCGATGGCAGGCAAGATCAAGCCCCAACACACT
CTACCGTAGTTGCAAGACCGAACAGTGTCGCTGTCGAAGGTGGTAGAAGCAGTTCAGCAGAGAGAGGCAGCGT
GTGAGTCTACCTGCTACCCCGTACTATGTATATCCCGACTATGAGACAGCCGCCGAGGTGTGCGGAGACGAAA
TAAATAACTAGTTATCTTTCGGCTCCCTGAGTTCCAGGCCGGACAAGCCCAGGCCAAGCTGCAGCAAGCCCCC
AAGCAAGAGAACGACGGCGCTTAGAGTCAATGGCAGGCCAGGCTGAGCGAACTTCCCTTGCTCGACCTTTTTT
TTCTGTCGAAAGATGCGAAGTCGCTCTTTATTAGCGAAGTGGGATCATCTCGTAGATGGCCTAGGTAAACAAC
TGCATATGTACCTTCACCGAGCTTCTTATCTTTCAGAATAACCAAATGTTAGCATTATGGGGTCCCAATGGTT
GGGGTGTATTTGCCCGCCCACCTTTGACATATTTCCGCCGTACATCAGCGTTTAACTGCTCCGCCAGCTCTGG
TTCGGGCATGGAGGGCTTAGGAACTGCAGGATCACCACTAGGCGCCGTCCGAGGCTCTTGAGAAGCAATATTT
CTCATCGCTAACGACTTTGGAGCACCGGTAGTAGTGATGGGAGGGAATTGAGAGGGAAGCGGCGACACTGCCA
TAATGTCAAGCGAAATTGGCTTCCACCAATTTCGCGAGACAAAGGATAGAGTGGCACAAGAGTGAGACACTGC
TCTGAGCAGCTCTCCTAGTATTCAATCAACAATACAGCTTTGTTTGTTATCAAAATATATATCGTCGTCTTTC
ATCTTTGAATGTTGCGATGGAAGCTTCGGCTGGCCCGAGCTCACCGGAGATCGGCGTTTTGTTGATCCGAGCT
AAGGCCGTCGGACCTAATTCAAAATTTTGGTGCAAAATCTTGAACGAAGTCGAAGAACCAGTTGAACATCTTC
TATACAGCGCCATTGAGCATTGGATTGACTCGCCTTGAGTCCCTTACCGGTTTACCTGGGCCCTTGGTGCCGT
GGCTTTTTTCGCTTTCGTCACGCCCGCCGATGGTGTTCTTACACCACGAGCTAAATTAAAATCTTCACCCCCC
AGATCTCGACATTGAACGGTTTGATATAAGGCGATATAACCACT**ATG**GCTGCGGCGCGCATACCGGGCTTTAA
TGCCAGAACATCCCTTCCACTACGGCTCTTCACTTCCGTCACAAACCCCCATTATCAGCCATGCGTACGGTAC
CTTCATCAATCTCGAAAACCCGCAACAGTGCCATCACCTACCCCATTCGTGCCCGATGTCGAAACATTCCTAC
GCCTAATTGGGCGAAACATGTCCAGATATTCCAGCAAAATCACCGGATGGAAGCAATTGTTTACAGCGGACTC
GGCTGAGCTTCGAGA

[Read No.m64281e_230311_112734/128582552/ccs] [Antisense (-) strand]

GGT~ATT (5'UTR)

CTA~CAT (*chkB* protein kinase genomic DNA) (Reverse sequence)

TCA~CAT (*TinCYP51B* gene) (Reverse sequence)

AGC~TTA (3'UTR)

ATG~TAG (*FYV4* genomic DNA) (Forward sequence)

~ACG (Downstream sequence of the 7374 block)

GCTCTCTTAACCAACTTCCTGAGGATCCTACCATAGTTGCTGTTGGATTTCAAGAGATTGTTGAGCTTAGCCC
CCAGCAGATTATGTCCACTGACCCAAGCACGCGCAAGATATGGGAACACGCAGTTATATCATCACTCAATGCC
AGGACCACGAAGAGAGGGACGACTGAGTACGTTTTGCTGAGAAGTGGTCAGCTAGTGGGTGCAGCACTCTTGC
TGTTCGTAAAGAAGGATGTTATAAATAAGATCAAGAATGCGGAAGGCAGCTTGAAGAAGGTATCCATCCTTTG
AATATAATATCTTTTGAGAAGCAGGGCTAACTGGACTGAGTAACAGACTGGCTTATCTGGCATGGGAGGGAAT
AAGGGTGCTGTTGCTATCCGCTTGGACTATAGTAATACGTCAATATGCTTCATCACTGCTCATCTAGCTGCTG
GCTTCTCGAACTACGAGGAAAGAAACAGAGACTACCACACCATAGCTCGGGGTCTCAGGTTCCAACGAAACAG
ACCTATTGTCGGACATGATGCAACAATCTGGTTCGGTGATTTCAACTATAGGATTGGCCTTAGCAATGAGCGG
GTGAGGCCTCTAATTGAATCGGGAGATATAGATGTACTATATCAGCATGACCAGGTATGTCTCTTGATGCGCG
AATACATGCCCTGACTACTACTAACTTTCAATAGCTCAACCTCCAAATGGTTGCTGGCCTTGCATTCCAGTAT
TATATGGAAGGGCCTGTCACATTCCCTCCAACATATAGATACGACAATGGCACAGATGAATATGACACCTCGT
AAGTTTGAGAGGCAGTCCATCCATTTAGACCTGTAAATACTAATAATACTTCTAGAGAAAAGCAAAGGATTCC
CGCCTGGTGCGACAGGATCCTTTGGAGAGGAAGGATTCTACGACAGCTAGCCTATACGACTGCACCTTTGAAA
TTTTCGGACCATCGACCTGTTTATGCAACATTCGAGTGCGATATATCGACGGTAGATGAGAAGCGAAAGGAAG
AAATAAGCTGTCGACTTTATGAAAAGCTGAAGCAACAACCTGCCACCACAACTACAAACCCGTTGTCTGATGA
TGAGGAAGATGATAATGAACAATACTCGATAAAGCTAGGCCTTCCACCTCCCAGCTCCGAAAAGCGTAAGTGG
TGGCTTGATCATGGTGAGTATATTTGACTTAGTTAACTAGTCGGCTCTTTAGCATTGATATGTTAAAGCTAAC
AATTTTATAGGCCTTCCTGCTCGATCAACTATTACTCCACCACTTGGTGCTGTGAGCAATGTTCGGCGAAGTG
CCAATCCTTTTTCATTTTCTCGGGGGTCGGATTGGATTACTGTCGGAAGAGATGATGCTTTGAGCGAAATGAG
CGAGCAGGACCTACGAGAGTCGAAAAAGGCATCCTCCACATACAGAGAGCCATTTAGATCGGTGGGAGAAGTT
ATGGGCGGTCCTGAATCGTCTGCCACAGACATGCCTCAAACCTCTAAGGTTGTCGTCAGCAGCAGAACTGTAA
AATCGGTAAAAATACCACCTCCGGTACCTAGGAAACCAGCAAAATTGAGACGACATTCATCAGGCGGAACCGA
TCGCAAAACCGCCTCAAGTAATGGTGTCTCGAGTGGATACGAAATGGTTAACCCTGCCGAGGATTTCCAAGAA
CAAGCATATACTGGCGGGGCTGGCGCAGCCACTACCTCATCCGTATCACAAGCTACCAAATCACTACCAACGA
ACGAGCTCATGGATTCAGATGCAGGAACGAGAGTAACTTCGTGGGAGCCCCTTCAACCGAAAAAGTGACCGAA
TGGTTAGGTGTTTTACGGCTAGCCAAAGGCTTTTAAGTTGGAAAGCGGTAACTCCCATAGAATCACTTTGAAA
CCATATATCATCAACGTCTCTCTCTATGCATGGCACGCCCAAGGCCTATTCTACCAGATTATATATGATGTCA
AGTACACAATGAATCCTGCAGTACTAAAGAGGGTAGGCGTTATCAAGTAGGGTGCTGCTTTTTGAATTTCAAC
CCATTACTTTGAGGGTGGGCTTCAAATTAGGAGATATTCCCCCTTTTTGATAGATCAACCCCAAGGTCAAACA
ATGCCGCCTGGTTTGTGGCCATCAGGGAACTGCCTGGGCTCATGAAAGCATAGATTTACGCGTATCGGCTTCC

TGCTGAGTGTGGTATAGGGGCCTGCTACTGACGTCTCCTCCGTATTGGTAAGGCTGCAGCTCATTAACCAAAT
TGGGCTCTCATTGAGGCCGATGTTTACACAGCATTGCACCCACAAAGAGGAGCGCCCAGAATGTTGTGTATCA
CTTCTTCTTGTAGCATATATTTGTCGGTACGCGATATCTGCTCGCTTGCTAACCAAGGAGGCGAGAATTTTTG
AGTATTATATTCCGGATGGCAAGTCGGATCCTCTCATTTCTTCATAGCTCCGAAATCAAGTTGACGGACACT
CCTTTAAATTTTGACTCGTCAATCTCACCAACTCGTCTGCCCATATCGTTGCCCATTTTCTTTTCTCCTCCTG
ATTTCCTAGGCAGGTCCTCGTTCTTAGTTGGTTTAGGCTCAGCAAGCCACCACTGATGCTGCAAGACTTGAGT
GGCCGTAATTCGTTTCCTTGGATCAAGTTTTAACATGGAGGCAAGTAGATCGGCCCCCAAAGGTCCAGCTGTC
GGAAATTGGCGGAGGAAGAATTCGCGGCCTTGTAGGGGTATGACATGGTTTTCCTCGATTGGGACGTAGTTGG
GCAGCTTTGTCACCCCAGGCCAGTTTTCTTCCGTAGGTGCCCCAAATGCAGCACAAATTTTAGATATTTGGTC
CATATCCGTGTTTCCAGCGGCGAATGGAACGCGCAGAATAAGCTCAGCGAACACCATTCCCATTAGACCATAT
GTCGACTGCACCGGAATATTGCCTGGCGCCGAAGAGTAGTTCTAATGGCCTGTACCATCTTGTGATGACTTGA
TGTGTCATGTTGAGATATGGATCGGCAAAGGATCTGGCAAGACCAAAATCCGCTAATTTAACTTCTCCATCCG
ATGCAATTAATAAGTTGTTAGGCTTAATATCACGGTGTAGAATGAAATTCTTATGACAGAACCACACTCCACG
TGCAAGCATGCTTATCCATGCTTTTATATCAGCCACACCGTACTGGATAGAGTTGTCTTTGATCAACATTTCA
AGATCTCCAAGGGGTAAAAATTCAAGGACGAGGTTTAGATTTTGATCCTTTGAGGAGAAAACGTCATGCAACG
CAATTACGTTCGGGTGAGACAGTTCCTGAAGGTACTTTACTTCACGGATGGCGTCCATGGATAGCCCGTCCTT
GTACTCGGCGTTTAGTTTTATTTTCTTGATTGCAACGAGCGAAGTGGGATCATCTCGTAGATGGCCTAGGTAA
ACAACTGCATATGTACCTTCACCGAGCTTCTTATCTTTCAGAATAACCAAATGTTAGCATTATGGGGTCCCAA
TGGTTGGGGTGTATTTGCCCGCCCACCTTTGACATATTTCCGCCGTACATCAGCGTTTAACTGCTCCGCCAGC
TCTGGTTCGGGCATGGAGGGCTTAGGAACTGCAGGATCACCACTAGGCGCCGTCCGAGGCTCTTGAGAAGCAA
TATTTCTCATCGCTAACGACTTTGGAGCACCGGTAGTAGTGATGGGAGGGAATTGAGAGGGAAGCGGCGACAC
TGCCATAATGTCAAGCGAAATTGGCTTCCACCAATTTCGCGAGACAAAGGATAGAGTGGCACAAGAGTGAGAC
ACTGCTCTGAGCAGCTCTCCTAGTATTCAATCAACAATACAGCTTTGTTTGTTATCAAAATATATATCGTCGT
CTTTCATCTTTGAATGTTGCGATGGAAGCTTCGGCTGGCCCGAGCTCACCGGAGATCGGCGTTTTGTTGATCC
GAGCTAAGGCCGTCGGACCTAATTCAAAATTTTGGTGCAAAATCTTGAACGAAGTCGAAGAACCAGTTGAACA
TCTTCTATACAGCGCCATTGAGCATTGGATTGACTCGCCTTGAGTCCCTTACCGGTTTACCTGGGCCCTTGGT
GCCGTGGCTTTTTTCGCTTTCGTCACGCCCGCCGATGGTGTTCTTACACCACGAGCTAAATTAAAATCTTCAC
CCCCCAGATCTCGACATTGAACGGTTTGATATAAGGCGATATAACCACT**ATG**GCTGCGGCGCGCATACCGGGC
TTTAATGCCAGAACATCCCTTCCACTACGGCTCTTCACTTCCGTCACAAACCCCCATTATCAGCCATGCGTAC
GGTACCTTCATCAATCTCGAAAACCCGCAACAGTGCCATCACCTACCCCATTCGTGCCCGATGTCGAAACATT
CCTACGCCTAATTGGGCGAAACATGTCCAGATATTCCAGCAAAATCACCGGATGGAAGCAATTGTTTACAGCG
GACTCGGCTGAGCTTCGAGAATTGGGCATAGAAATGACAAGGGACAGACGATACATACTGCGGTGGAGAGAGA
AATTCAGACAGCAGGACTACGGCCCAGGAGGTGATTTCGAAAACGTTGTTGACGGTGTTGCTGAGCTGAGAGC
TGTTGAGGTCCCCCGTCGAAAAGTCAAAGAGTGCAAAAACTACCAATGCCACCGACGGAGAGTCCCTAGATGT
TAGCACAATGGGCACCGCTACCCTTACACCTGGAATGCGTTATGCGATTGTCAATCTACCACCAGGCGAAACA
GAGCTCAAGGATACTTCCAAGCCACTCAAAAAGTTTGCCCATTATAAATTGCACCACGGGAATATGATCAAGG
GGCCGTATGCTCAGATTATTCCAAACACATGGAGTACCGCCGTCAAGGTTCAGGTTACTGATGGCATGTGGGA
GCATAAGCGTGGCAGAAAGATTGACGGTGGTCAGAGAAGACAGAAGGAGATCAGAGCGAAGAGAGCGTTGGCG

GAGAGGAGGAATGCTCAGGCAGCTGCT**TAG**GCGTTTTGTAGCGTCGGAGTTGAAAGGGGGCACGGGATTAACG
ACGTTGCTGGCTGGTTACTTCCTGACATTACTGGCTTGGATATCGCTGTTTAGATGCTAGATTATCCCAGCTA
CGGGAAGATGTACTATTTCCAATGTATATTACCATTTTGTTTCTGCACCAAAGCTAACGGAGTAACTGCAACT
TTCCTGTTCATCTAGAGTATTGTAATAAATGGCTGTATTAGAATAATACTATTATCTACATCCCCCATCTTCA
AAAGCATGGTCAAAAGAAAGTTTTTATTACATATGCTCTATTCCGAAAACCTATTTGCGTGACCATAAAGTGC
AAATATGGCTCGGTGTATAAACGTAAGAAAAGAGAAGAAATAAATTAGCAAGAAGCAAGAAATTTAACATGAT
TCAATCCGCAGTCGTGATTA**TCA**TTTCTGGGAAGGCTGCTTTCGCTTCTCCCACGAGACCATTGGGTCGCCAA
GGGGCTTCGAGAAGAGGGACTATTAATGCAAAGTTAGTAAAATCGAGCAAAATAAAAAGGAGCGTAATATGCC
AAACTTACCGAATAGTCAGTTGGCGGCACAACATCTTTGGTGCCTTCAACTTGCTTCCATTTCATTAGTCTGG
CTAACGTAGCTGTTACGGTTCCAAGCTGAACATATGCAAATTGTTCACCGATGCATCTGTGTCTGCCTGAGCC
GAATGGGAGGTAAGGGCTGTTGGCACCCTTGCTAACCAAACCGTATCCGTAATCTGTCTTTTCTTCACTGGCC
TCGACTACGGTGACACCGACGTTGTTCTCCCATCGGTGAGGGTTCCAAACAAGAGGGTCAGGGAAGTACTGAG
GGTCTCGTGAGGGAACACCAGGAGCGGCAAGGAGGTTGTGAGAGGTTGGGACAACATAGTTAGTTCCTTCAAC
GGGCATAGGGGATTTAACAGCACGGAGGATAGAGTGGATGGGAGCGTGGAGGCGGAGAGTCTCCTTGATTACA
TTGTTGTGAAGATCAAGTTTCTGGAGAGATTCGTAGGTGAGTGGTGGAAGATCCTCGCCGAGAACACGTTTCT
GTTCCTCGTAGAGTTCCTCGAGAATATCTGGACGGCTGGCAAGGCGGAGAACGATCCAGGAGCCGGTGGAGGA
AGAAGAGTGCTGGCCAGCCATAAGAAGAGCAATCATCATGTGGGCGACTTCGATATCTGGAATTGGCGTTCCA
TTCTTGTACACGGACGACATCAAGTTCCATACCATGTCCTCGGAGTCTTTCTCTCCACCAGCTCACGTCGCTG
ACGGATGATGTCGGTGTAAACCTGGGCCATCTTCTTTTGAGCACGGTCACGCTTGCGGTTGTGTGGGAAGGGG
AACCATGGGAACATGAAGTTGATGGCGGCGAAGCCCATGTCGAGATCGGAGTAGAGTTCCGCAAAGCTGGAAT
CGAACTTTCCTCGCACCTCCTTGCCCTGTAGAGATCGAGAGGCGGTGTAGATGGTGATTTCAGCCATGACCTT
GCTGACGTTGAAAACTCCGGAGTCGCCCTTGAAGACGGGGGAGGACTCGAAGAACTGCTCAACTTCCTTGGTG
ATCAGGGTGACATAGGATCGGAGAGCTTCAGAGGTGAGGCCGAACTTGACGAACTGGTAGACGATTAAATTAG
CAATGGAGGCCCAGGAGATGTAGACGGGAATAAATTGCTGTACACACCTTCTTCTGCTCCATGAGCTTGGAGT
TTGGGCAATCATACACCACATGTCGTCCGAACACTGGGGTGGTGAGGGGGGAGTAGACATCCTCCGCGCAAAC
ATCCTTGAGCTTGCCGTTCAAAATGAAATCATTTCCCTTTGTACCGAGAAAAACAGTCGTCTTCTTGCCCAGC
AGTATGAATGTGAAGATGTCTCCATACTAGAGTACCGAAATTATTTATACGTTAGCTTTGCGCAACCTGGTAT
ATAGCTAAGTTGAATTGCACCAACTAACCTTCTCCTTGCAGTCGTCAAAGAACTTGTACGGGTCAATTCCATA
GGAGATCGTGCTTCCAATGACCGGAACCCAGTGGAAGACCACCGGAGGCTTTGTAGGGTCCTTGAACAGGAGC
TGCTGCAGGACGTTGATAACAATGGAGAGGACGATAAAAGCCGATACGGCACTTGCGACGAGCGCGGCGGTGG
ACAGGGTCGAGCAGTTCTCGCAGAAACGAGAGACAATGTCGGCTAAGAGGCC**CAT**GGTGATCGTCTTATCCTC
CTGCTTCCGCTCAAGACTTGTTCGTTGTTTGTGATGAATGCCTTGAAGCTGCTGCGGACGAAGCAAGGGAAAA
AAAAAGTTGCATCTTGAGATCGAACTCGCATTTTATGCAAGGGGGCGTACGGTACTGTAAGATAATGCATTGT
TCCTGGTATTTTACGTCGTTTTTTATTGTTTACAGCTGCATCTCCAATACCAAAATTACCGCTGCCCTTCTGC
CCCCCTTTTTCTCCCTTGCGGCGAGCAGGCCAACGGCCGTCGAGATGATTACAATTCGAGCTCGCGACGGGGC
GGAGACGGTCCCTGGACGGTGTTCGTGCAGGGCGTCATCTCCTCCGCTGGATTCTCTGCTGCCTGAGCGAGCG
AGACCTGACGGTGGGCGCATCATCAGGCCATCGCGGCTTCAGGGAGGCCGGCTTAGTCATGGCAGGTTAAGCT
ATCAACGGTCCGTCAATATTAATATTGTTATTATTGTCCGGTGGTGAGTGAGAAGAGACTGTAATTACATTGC

CCAGTGGACGCTATTGTACCCATAGTTATATCACTCAAAAATGAAATCCTAAACACGAGAATCGCCGTCGTCT
CCAAGGCAGCGACCAAGCCGGTTTTTCTGCTGCTTTTCGGTTAGTTGGCCCCCTATCACCTTTCAACCGGGGG
GTATAGTTAAAGAAAAAATTAAGAAAGATCACAATCAATATAAACGAAAAAGGAATTGGAAACAAAAGTCCGG
AATAGCACGGTATAGCTAGCAGAATACCATCCCAAGACGGATCGGGCTTCTG**CTA**CCTCTTTCTTTCTCGTGG
AGTCTTGTCTGCGTCGTCTTGGTGGACAACTGGCTTCCGTGCCTCACTCTCAAGCTCCAGCGAGTCGTCAGAC
TGCTCATCGAATAACACCTGGTCTCCACTTCCACCGAGTTCCATGAACTCCTTTGAGTTCGGTTGCCAGCCGT
TTGGGCTCCTGGGCTCGAGGCACAGGATGATTCTGGTATATAGTTATGTCCTGGGAAGCAGGGTCGTCTTCCT
TGGTCTTCACCACCTGCTTGACCGTGACTTCATGTATGTCACTCAACAGCCTGCGGACCATAGTTGGCCGGCG
TTCTTTGGACACCCTCAGGCCACCGATGGCCCCGGCTAGGTCATCGGTGCTGTCTGCCACCCTAGGCTCATCG
GGGTCGATGCCAGTGATCCATGGGTGCTGGAGACACTCATCTACTTTCAAACGCTCCTTCACGTCGACTTCGA
TCATGGAGTCGATAAGGTCCATGGCTCTATAATCGATTGAGTCCCAGTATGGAAGAGGATACTTAAATATTCC
GAGCTGGATCTGCTCTTTCTGAGTGTATGGGTAGTCTCTTGGGTGGTACAGTTCATCTGAGAAAGGAGGGAAT
CCGCACAGGCAGATGTAGAGTACCACTCCGAGTGACCAGATGTCAACCGCTCGGGTGTACATTCTGGCTTCAG
CGTTTTCTGCCAGTATCTCAGGAGCAACATCTGATAAGAATTGTTAGATTCACATTTGGTGGATGAAAGCACC
AGCGTTAACAGAGCAGCAGGGAAACTTACAGCCAGGCGTTCCACATCTAGATTTTATGATCTCTTAGTAAGAT
TGACCCCAGATTTGAACGCAGAAGGTAGGTGAACTTACAGCGTGGTGGTAAAGGAGTGTTCGCCGACAATCTT
TGCAAGGCCGAAGTCTGCTAATTTTACGGTAAGATCTTTGTCGACAACCAGAATGTTCTCTGGCTTTATGTCA
CGGTGAATGATGTTTCGTTCATGCTACGACAACGGTTAGCCATATACACTGCCATGAGTCTGATCAACCAGAA
AACGAACGACATACCAGGTATCTCAAACCTTCAAAAAGCTGAATGAATACTTTTCGAGTCTCCTCCTCGCTGA
GTTTCTGATGCCTAATGATCCAGTTGAACAGTTCGCCCTCTGGCGCAAGCTCAAGAATCAGGAAGACACCATC
GCTCTCGTCAAAGGTATCCTTTAGGCACAGCATGTTCGGATGGTTGACACTCATCAGCATTGAAATCTCTTGC
TGTAGTGCTTCACGGTCTAGCCTCTGAACTGCGCCCGGGCGTTTCTCGAAATGTTTGACGGCATACTTGATCC
CCGTAGAACGTTCAGCGCATAGATAAACCGAGGCAAAGTGTCCCCTACCCAGCATATCAAAGAGCTTATACTG
CTGTCGGAACTTGCTTGTGTTCCGACTCCGCGGGTAGTTGAAGATGAATCTGGATTCTTGCAGGATGGTAATC
TCGTCGCCGTTTTCGAGCTCTCGGCGTTTGTTCTTGCCGATGATGGCGTCGTTGATGAAAGTGCCATTGATAG
AGAGATCTTCCACAATTGCAACAAAGTCTCCTCCCTGTTTTCGTGGAATATCAGGCAATGGCGGTTGGATATC
GTGGGGAGCTTGATCCTGAAATCTGCAGTACCCAGATGGTTAGCAATTGATCACTAGACGGCAGAGAGTATGC
AATGGGTAAAGGCATACCGCACTCTGCATGCCGCCTATAAGATATCCAGCGCGGTTTATCTCTCTAGTTCCAC
GTTTTGAGGCGTTGCTTTCCGGCGTTGATATGTCTGATGCTGCTCCAATTACCGGTGTAGTATTTGTGCTGTC
CCGCTTCTTCAGAACCAATTGGCCTCCAAAATTGTTATCTAGCGGAGTAAGATATCCCCATATGCCCTGGGCC
ACCTCGTCTTCCACATCGTCAATGGATTGGATTGGGGGATACTGAGAGAAACCCTGGGTGGGACATGACGACT
CGTCTGGCGATTGACCTCGAGGCGCCTGGTTTCGGCGGCCTTCTGGGGGAGAAACTGTAGCAACTTCACGCTT
TTCTTCTGAGATTACTTCATTAGCTAGGGGAGAAGGTAGATATCCGGCATCCTGCTGCAATTGAGATGAGATT
CGAGGAGATCTCCGTGGCTTCTTTGATTCATGGTCTGGAGCTGCGTTGCCCTGCAGGTTTGGGAAAAGAAGTT
AGCAGCTTGAATATTTTCATCCTTCTGAGAAACTAATTGGATATAACGTGTTCATATCTTACCCTTTCTCTCT
TCAGATTGCTTCTCTCCCTCTGCGGAGG**CAT**GTTTTGGCCGGCGTGAAGAAGTTGACGGCAGTTACGAATATC
CCGGATGGACTGCCAATGGCTTCGCAACTTCAACTCCAAGTCGATGGCAGGCAAGATCAAGCCCCAACACACT
CTACCGTAGTTGCAAGACCGAACAGTGTCGCTGTCGAAGGTGGTAGAAGCAGTTCAGCAGAGAGAGGCAGCGT

GTGAGTCTACCTGCTACCCCGTACTATGTATATCCCGACTATGAGACAGCCGCCGAGGTGTGCGGAGACGAAA
TAAATAACTAGTTATCTTTCGGCTCCTGAGTTCCAGGCCGGACAAGCCCAGGCCAAGCTGCAGCAAGCCCCCA
AGCAAGAGAACGACGGCGCTTAGAGTCAATTGGCAGGCCAGGCTGAGCGAACTTCCCTTGCTCGACCTTTTTT
TTCTGTCGAAAGATGCGAAGTCGCTCTTTATTAGCGAAGTGGGATCATCTCGTAGATGGCCTAGGTAAACAAC
TGCATATGTACCTTCACCGAGCTTCTTATCTTTCAGAATAACCAAATGTTAGCATTATGGGGTCCCAATGGTT
GGGGTGTATTTGCCCGCCCACCTTTGACATATTTCCGCCGTACATCAGCGTTTAACTGCTCCGCCAGCTCTGG
TTCGGGCATGGAGGGCTTAGGAACTGCAGGATCACCACTAGGCGCCGTCCGAGGCTCTTGAGAAGCAATATTT
CTCATCGCTAACGACTTTGGAGCACCGGTAGT
