# Supplementary material for: Two different types of tandem sequences mediate the overexpression of TinCYP51B in azole-resistant Trichophyton indotineae
Source: Antimicrob Agents Chemother. 2023 Oct 12;67(11):e00933-23. doi: 10.1128/aac.00933-23 (PMC10648874; doi:10.1128/aac.00933-23)
Supplement: NGS raw sequence reads — Nucleotide sequence of the TinCYP51B locus in TIMM20122. [file aac.00933-23-s0002.pdf]

***TinCYP51B* locus in TIMM20122**

[Read No.m64281e_230107_125321/117836369/ccs] [Antisense (-) strand]

ATA~ (Upstream sequence of the 7374 block)

GGT~ATT (5'UTR)

CTA~CAT (*chkB* protein kinase genomic DNA) (Reverse sequence)

TCA~CAT (*TinCYP51B* gene) (Reverse sequence)

AGC~TTA (3'UTR)

ATG~TAG (*FYV4* genomic DNA) (Forward sequence)

CATTAGCTAGGGGAGAAGGTAGATATCCGGCATCCTGCTGCAATTGAGATGAGATTCGAGGAGATCTCCGTGG
CTTCTTTGATTCATGGTCTGGAGCTGCGTTGCCCTGCAGGTTTGGGAAAAGAAGTTAGCAGCTTGAATATTTT
CATCCTTCTGAGAAACTAATTGGATATAACGTGTTCATATCTTACCCTTTCTCTCTTCAGATTGCTTCTCTCC
CTCTGCGGAGG**CAT**GTTTTGGCCCGGCGTGAAGAAGTTGACGGCAGTTACGAATATCCCGGATGGACTGCCAA
TGGCTTCGCAACTTCAACTCCAAGTCGATGGCAGGCAAGATCAAGCCCCAACACACTCTACCGTAGTTGCAAG
ACCGAACAGTGTCGCTGTCGAAGGTGGTAGAAGCAGTTCAGCAGAGAGAGGCAGCGTGTGAGTCTACCTGCTA
CCCCGTACTATGTATATCCCGACTATGAGACAGCCGCCGAGGTGTGCGGAGACGAAATAAATAACTAGTTATC
TTTCGGCTCCCTGAGTTCCAGGCCGGACAAGCCCAGGCCAAGCTGCAGCAAGCCCCCAAGCAAGAGAACGACG
GCGCTTAGAGTCAATGGCAGGCCAGGCTGAGCGAACTTCCCTTGCTCGACCTTTTTTTTCTGTCGAAAGATGC
GAAGTCGCTCTTTATTAGCGAAGTGGGATCATCTCGTAGATGGCCTAGGTAAACAACTGCATATGTACCTTCA
CCGAGCTTCTTATCTTTCAGAATAACCAAATGTTAGCATTATGGGGTCCCAATGGTTGGGGTGTATTTGCCCG
CCCACCTTTGACATATTTCCGCCGTACATCAGCGTTTAACTGCTCCGCCAGCTCTGGTTCGGGCATGGAGGGC
TTAGGAACTGCAGGATCACCACTAGGCGCCGTCCGAGGCTCTTGAGAAGCAATATTTCTCATCGCTAACGACT
TTGGAGCACCGGTAGTAGTGATGGGAGGGAATTGAGAGGGAAGCGGCGACACTGCCATAATGTCAAGCGAAAT
TGGCTTCCACCAATTTCGCGAGACAAAGGATAGAGTGGCACAAGAGTGAGACACTGCTCTGAGCAGCTCTCCT
AGTATTCAATCAACAATACAGCTTTGTTTGTTATCAAAATATATATCGTCGTCTTTCATCTTTGAATGTTGCG
ATGGAAGCTTCGGCTGGCCCGAGCTCACCGGAGATCGGCGTTTTGTTGATCCGAGCTAAGGCCGTCGGACCTA
ATTCAAAATTTTGGTGCAAAATCTTGAACGAAGTCGAAGAACCAGTTGAACATCTTCTATACAGCGCCATTGA
GCATTGGATTGACTCGCCTTGAGTCCCTTACCGGTTTACCTGGGCCCTTGGTGCCGTGGCTTTTTTCGCTTTC
GTCACGCCCGCCGATGGTGTTCTTACACCACGAGCTAAATTAAAATCTTCACCCCCCAGATCTCGACATTGAA
CGGTTTGATATAAGGCGATATAACCACT**ATG**GCTGCGGCGCGCATACCGGGCTTTAATGCCAGAACATCCCTT
CCACTACGGCTCTTCACTTCCGTCACAAACCCCCATTATCAGCCATGCGTACGGTACCTTCATCAATCTCGAA
AACCCGCAACAGTGCCATCACCTACCCCATTCGTGCCCGATGTCGAAACATTCCTACGCCTAATTGGGCGAAA
CATGTCCAGATATTCCAGCAAAATCACCGGATGGAAGCAATTGTTTACAGCGGACTCGGCTGAGCTTCGAGAA
TTGGGCATAGAAATGACAAGGGACAGACGATACATACTGCGGTGGAGAGAGAAATTCAGACAGCAGGACTACG
GCCCAGGAGGTGATTTCGAAAACGTTGTTGACGGTGTTGCTGAGCTGAGAGCTGTTGAGGTCCCCGTCGAAAA
GTCAAAGAGTGCAAAAACTACCAATGCCACCGACGGAGAGTCCCTAGATGTTAGCACAATGGGCACCGCTACC
CTTACACCTGGAATGCGTTATGCGATTGTCAATCTACCACCAGGCGAAACAGAGCTCAAGGATACTTCCAAGC
CACTCAAAAAGTTTGCCCATTATAAATTGCACCACGGGAATATGATCAAGGGGCCGTATGCTCAGATTATTCC

AAACACATGGAGTACCGCCGTCAAGGTTCAGGTTACTGATGGCATGTGGGAGCATAAGCGTGGCAGAAAGATT
GACGGTGGTCAGAGAAGACAGAAGGAGATCAGAGCGAAGAGAGCGTTGGCGGAGAGGAGGAATGCTCAGGCAG
CTGCT**TAG**GCGTTTTGTAGCGTCGGAGTTGAAAGGGGGCACGGGATTAACGACGTTGCTGGCTGGTTACTTCC
TGACATTACTGGCTTGGATATCGCTGTTTAGATGCTAGATTATCCCAGCTACGGGAAGATGTACTATTTCCAA
TGTATATTACCATTTTGTTTCTGCACCAAAGCTAACGGAGTAACTGCAACTTTCCTGTTCATCTAGAGTATTG
TAATAAATGGCTGTATTAGAATAATACTATTATCTACATCCCCCATCTTCAAAAGCATGGTCAAAAGAAAGTT
TTTATTACATATGCTCTATTCCGAAAACCTATTTGCGTGACCATAAAGTGCAAATATGGCTCGGTGTATAAAC
GTAAGAAAAGAGAAGAAATAAATTAGCAAGAAGCAAGAAATTTAACATGATTCAATCCGCAGTCGTGATTA**TC**
ATTTCTGGGAAGGCTGCTTTCGCTTCTCCCACGAGACCATTGGGTCGCCAAGGGGCTTCGAGAAGAGGGACTA
TTAATGCAAAGTTAGTAAAATCGAGCAAAATAAAAAGGAGCGTAATATGCCAAACTTACCGAATAGTCAGTTG
GCGGCACAACATCTTTGGTGCCTTCAACTTGCTTCCATTTCATTAGTCTGGCTAACGTAGCTGTTACGGTTCC
AAGCTGAACATATGCAAATTGTTCACCGATGCATCTGTGTCTGCCTGAGCCGAATGGGAGGTAAGGGCTGTTG
GCACCCTTGCTAACCAAACCGTATCCGTAATCTGTCTTTTCTTCACTGGCCTCGACTACGGTGACACCGACGT
TGTTCTCCCATCGGTGAGGGTTCCAAACAAGAGGGTCAGGGAAGTACTGAGGGTCTCGTGAGGGAACACCAGG
AGCGGCAAGGAGGTTGTGAGAGGTTGGGACAACATAGTTAGTTCCTTCAACGGGCATAGGGGATTTAACAGCA
CGGAGGATAGAGTGGATGGGAGCGTGGAGGCGGAGAGTCTCCTTGATTACATTGTTGTGAAGATCAAGTTTCT
GGAGAGATTCGTAGGTGAGTGGTGGAAGATCCTCGCCGAGAACACGTTTCTGTTCCTCGTAGAGTTCCTCGAG
AATATCTGGACGGCTGGCAAGGCGGAGAACGATCCAGGAGCCGGTGGAGGAAGAAGAGTGCTGGCCAGCCATA
AGAAGAGCAATCATCATGTGGGCGACTTCGATATCTGGAATTGGCGTTCCATTCTTGTACACGGACGACATCA
AGTTCCATACCATGTCCTCGGAGTCTTTCTCTCCACCAGCCTCACGTCGCTGACGGATGATGTCGGTGTAAAC
CTGGGCCATCTTCTTTTGAGCACGGTCACGCTTGCGGTTGTGTGGGAAGGGGAACCATGGGAACATGAAGTTG
ATGGCGGCGAAGCCCATGTCGAGATCGGAGTAGAGTTCCGCAAAGCTGGAATCGAACTTTCCTCGCACCTCCT
TGCCCTGTAGAGATCGAGAGGCGGTGTAGATGGTGATTTCAGCCATGACCTTGCTGACGTTGAAAACTCCGGA
GTCGCCCTTGAAGACGGGGGAGGACTCGAAGAACTGCTCAACTTCCTTGGTGATCAGGGTGACATAGGATCGG
AGAGCTTCAGAGGTGAGGCCGAACTTGACGAACTGGTAGACGATTAAATTAGCAATGGAGGCCCAGGAGATGT
AGACGGGAATAAATTGCTGTACACACCTTCTTCTGCTCCATGAGCTTGGAGTTTGGGCAATCATACACCACAT
GTCGTCCGAACACTGGGGTGGTGAGGGGGGAGTAGACATCCTCCGCGCAAACATCCTTGAGCTTGCCGTTCAA
AATGAAATCATTTCCCTTTGTACCGAGAAAAACAGTCGTCTTCTTGCCCAGCAGTATGAATGTGAAGATGTCT
CCATACTAGAGTACCGAAATTATTTATACGTTAGCTTTGCGCAACCTGGTATATAGCTAAGTTGAATTGCACC
AACTAACCTTCTCCTTGCAGTCGTCAAAGAACTTGTACGGGTCAATTCCATAGGAGATCGTGCTTCCAATGAC
CGGAACCCAGTGGAAGACCACCGGAGGCTTTGTAGGGTCCTTGAACAGGAGCTGCTGCAGGACGTTGATAACA
ATGGAGAGGACGATAAAAGCCGATACGGCACTTGCGACGAGCGCGGCGGTGGACAGGGTCGAGCAGTTCTCGC
AGAAACGAGAGACAATGTCGGCTAAGAGGCC**CAT**GGTGATCGTCTTATCCTCCTGCTTCCGCTCAAGACTTGT
TCGTTGTTTGTGATGAATGCCTTGAAGCTGCTGCGGACGAAGCAAGGGAAAAAAAAAGTTGCATCTTGAGATC
GAACTCGCATTTTATGCAAGGGGCGTACGGTACTGTAAGATAATGCATTGTTCCTGGTATTTTACGTCGTTTT
TTATTGGTTTACAGCTGCATCTCCAATACCAAAATTACCGCTGCCCTTCTGCCCCCCTTTTTCTCCCTTGCGG
CGAGCAGGCCAACGGCCGTCGAGATGATTACAATTCGAGCTCGCGACGGGGCGGAGACGGTCCCTGGACGGTG
TTCGTGCAGGGCGTCATCTCCTCCGCTGGATTCTCTGCTGCCTGAGCGAGCGAGACCTGACGGTGGGCGCATC

ATCAGGCCATCGCGGCTTCAGGGAGGCCGGCTTAGTCATGGCAGGTTAAGCTATCAACGGTCCGTCAATATTA
ATATTGTTATTATTGTCCGGTGGTGAGTGAGAAGAGACTGTAATTACATTGCCCAGTGGACGCTATTGTACCC
ATAGTTATATCACTCAAAAATGAAATCCTAAACACGAGAATCGCCGTCGTCTCCAAGGCAGCGACCAAGCCGG
TTTTTCTGCTGCTTTTCGGTTAGTTGGCCCCCTATCACCTTTCAACCGGGGGGTATAGTTAAAGAAAAAATTA
AGAAAGATCACAATCAATATAAACGAAAAAGGAATTGGAAACAAAAGTCCGGAATAGCACGGTATAGCTAGCA
GAATACCATCCCAAGACGGATCGGGCTTCTG**CTA**CCTCTTTCTTTCTCGTGGAGTCTTGTCTGCGTCGTCTTG
GTGGACAACTGGCTTCCGTGCCTCACTCTCAAGCTCCAGCGAGTCGTCAGACTGCTCATCGAATAACACCTGG
TCTCCACTTCCACCGAGTTCCATGAACTCCTTTGAGTTCCGGTTGCCAGCCGGTTTGGGCTCCTGGGCTCGAG
GCACAGGATGATTCTGGTATATAGTTATGTCCTGGGAAGCAGGGTCGTCTTCCTTGGTCTTCACCACCTGCTT
GACCGTGACTTCATGTATGTCACTCAACAGCCTGCGGACCATAGTTGGCCGGCGTTCTTTGGACACCCTCAGG
CCACCGATGGCCCCGGCTAGGTCATCGGTGCTGTCTGCCACCCTAGGCTCATCGGGGTCGATGCCAGTGATCC
ATGGGTGCTGGAGACACTCATCTACTTTCAAACGCTCCTTCACGTCGACTTCGATCATGGAGTCGATAAGGTC
CATGGCTCTATAATCGATTGAGTCCCAGTATGGAAGAGGATACTTAAATATTCCGAGCTGGATCTGCTCTTTC
TGAGTGTATGGGTAGTCTCTTGGGTGGTACAGTTCATCTGAGAAAGGAGGGAATCCGCACAGGCAGATGTAGA
GTACCACTCCGAGTGACCAGATGTCAACCGCTCGGGTGTACATTCTGGCTTCAGCGTTTTCTGCCAGTATCTC
AGGAGCAACATCTGATAAGAATTGTTAGATTCACATTTGGTGGATGAAAGCACCAGCGTTAACAGAGCAGCAG
GGAAACTTACAGCCAGGCGTTCCACATCTAGATTTTATGATCTCTTAGTAAGATTGACCCCAGATTTGAACGG
CAGAAGGTAGGTGAACTTACAGCGTGGTGGTAAAGGAGTGTTCGCCGACAATCTTTGCAAGGCCGAAGTCTGC
TAATTTTACGGTAAGATCTTTGTCGACAACCAGAATGTTCTCTGGCTTTATGTCACGGTGAATGATGTTTCGT
TCATGCTACGACAACGGTTAGCCATATACACTGCCATGAGTCTGATCAACCAGAAAACGAACGACATACCAGG
TATCTCAAACCTTCAAAAAGCTGAATGAATACTTTTCGAGTCTCCTCCTCGCTGAGTTTCTGATGCCTAATGA
TCCAGTTGAACAGTTCGCCCTCTGGCGCAAGCTCAAGAATCAGGAAGACACCATCGCTCTCGTCAAAGGTATC
CTTTAGGCACAGCATGTTCGGATGGTTGACACTCATCAGCATTGAAATCTCTTGCTGTAGTGCTTCACGGTCT
AGCCTCTGAACTGCGCCCGGGCGTTTCTCGAAATGTTTGACGGCATACTTGATCCCCGTAGAACGTTCAGCGC
ATAGATAAACCGAGGCAAAGTGTCCCCTACCCAGCATATCAAAGAGCTTATACTGCTGTCGGAACTTGCTTGT
GTTCCGACTCCGCGGGTAGTTGAAGATGAATCTGGATTCTTGCAGGATGGTAATCTCGTCGCCGTTTTCGAGC
TCTCGGCGTTTGTTCTTGCCGATGATGGCGTCGTTGATGAAAGTGCCATTGATAGAGAGATCTTCCACAATTG
CAACAAAGTCTCCTCCCCTGTTTTCGTGGAATATCAGGCAATGGCGGTTGGATATCGTGGGGAGCTTGATCCT
GAAATCTGCAGTACCCAGATGGTTAGCAATTGATCACTAGACGGCAGAGAGTATGCAATGGGTAAAGGCATAC
CGCACTCTGCATGCCGGCCTATAAGATATCCAGCGCGGTTTATCTCTCTAGTTCCACGTTTTGAGGCGTTGCT
TTCCGGCGTTGATATGTCTGATGCTGCTCCAATTACCGGTGTAGTATTTGTGCTGTCCCGCTTCTTCAGAACC
AATTGGCCTCCAAAATTGTTATCTAGCGGAGTAAGATATCCCCATATGCCCTGGGCCACCTCGTCTTCCACAT
CGTCAATGGATTGGATTGGGGGATACTGAGAGAAACCCTGGGTGGGACATGACGACTCGTCTGGCGATTGACC
TCGAGGCGCCTGGTTTCGGCGGCCTTCTGGGGGAGAAACTGTAGCAACTTCACGCTTTTCTTCTGAGATTACT
TCATTAGCTAGGGGAGAAGGTAGATATCCGGCATCCTGCTGCAATTGAGATGAGATTCGAGGAGATCTCCGTG
GCTTCTTTGATTCATGGTCTGGAGCTGCGTTGCCCTGCAGGTTTGGGAAAAGAAGTTAGCAGCTTGAATATTT
TCATCCTTCTGAGAAACTAATTGGATATAACGTGTTCATATCTTACCCTTTCTCTCTTCAGATTGCTTCTCTC
CCTCTGCGGAGG**CAT**GTTTTGGCCCGGCGTGAAGAAGTTGACGGCAGTTACGAATATCCCGGATGGACTGCCA

ATGGCTTCGCAACTTCAACTCCAAGTCGATGGCAGGCAAGATCAAGCCCCAACACACTCTACCGTAGTTGCAA
GACCGAACAGTGTCGCTGTCGAAGGTGGTAGAAGCAGTTCAGCAGAGAGAGGCAGCGTGTGAGTCTACCTGCT
ACCCCGTACTATGTATATCCCGACTATGAGACAGCCGCCGAGGTGTGCGGAGACGAAATAAATAACTAGTTAT
CTTTCGGCTCCCTGAGTTCCAGGCCGGACAAGCCCAGGCCAAGCTGCAGCAAGCCCCCAAGCAAGAGAACGAC
GGCGCTTAGAGTCAATGGCAGGCCAGGCTGAGCGAACTTCCCTTGCTCGACCTTTTTTTTCTGTCGAAAGATG
CGAAGTCGCTCTTTATATAAGGTTGGCCGCCCGTTAGAGCCGCATCCCGCAGTTTCTTATTATTTTTTGCCTT
CGTCACTTTGTGTTATTTCAACTCCTGCTTGGATGCAAATATCCAGCTATCTTGTAAAAGCATTTCCGTATCA
TAGCCTAAGGCACAAAAGTCGATCATCGAGGTTTTCTCGCGCAGACTGTTTTTCAATGCCTGGCTTGCATGTA
GCGGGCTCAACCCCCCGGGAGACAGAGACAGGCCCGGAGGCGCAAGAGAAAAACCCTGTAGAGGAAGAGTGGC
TTGTCCAGAGTAATTCAACAAGTTGGCAACCCTGGCTGCGTCCGTATCGAGTGTATCAAAATGCCCTTTCATG
CATCTCTCTCGCTTTGCCGTCTTGCTGCCCAGTCGTTTCTCTGCCCCGGCGTCACTAAACGGGCCTCGGCTTG
GCGGGTTGATCCTACCAGCGACTCGCATCTCCAGCAGGCAGAGCACTTCTGATACACTCCTGACCACGCCCCC
AAAACACACGACTGCACGTTCGGTGCATCCCCACCCCTTGGAACCACGAAAAAGAAAGGCCTCCTGGATGACG
GCGAGGCTGGTTGACCGGATATGTAGGTGTTCTCGCCCTGTGTTGGTGCTACGTTGGGCGCAGACATTTGCTA
ACTAACCGCCGGGGGAGAGCTTCAGGCATCATTCACCATCGTATATCCGGCGTGCGGCGCTCGAGAAACTTGC
GATACTAGCCACAAAACACCCCCGGCAGGACGATGATCAGGGCAATTCAGACGTCGAGAGGCTCATAAGTGCC
TGCAATGGCTCCAAGGCGGCTTCCAGTGGCTCCCAGGCTCGCTGCGCCTCGAGTGTACTGCCCCCCCCCTTTT
ACTGCCTCAATACATACACACACCCCTCCTTCAATGGCATATGCGCGAGAAAGTGGCTAATGAGGAAGATAGG
GGGTTCGAGAACTAGACGTACTGCTTGCTCTGTGCAAGGCAGCTGGCTCTGTTCAGCGTGGTGACCATGCTGC
AAAGCTGGTTTCTCTACTGGTCGGATACCTGCTTACGCTCCATACGCTCCGCATCCATTCGTCTCCTTTCCTA
CATATTTACAAACCCTCGCCGTGGGAGACCTTGACCCATGATGTGGTTACGGCGTTGCTTTCACTGGGCTCTA
GGCACAAGTCGCTGCAGGATGACGTCCACGAAGCCATTGACGAGTACTTGGAGAATTGTCACCAGGCAACAGA
CCGCCTGCCGGCCGTTTTTGATGCAGATGGGACAGGGATACAGGTCGAGAGCCATGAATCTACGGATGTGACT
ACTCTAATGGTATCGCTAGTGGGATTTCTCCGGGCCTCGTCCGAATTCCCCCATTTTTGGGCACCGCTGGATC
GCCTTGCTATTGTAAAGCAGTTACACATCATTCTATCAGAGCGGTTCCTGGTAGCTGTGGAGACCATCTCTTC
CGGCATACGGAACTCTCGCTCTCCAGATCCTCTGCTGCACGACTGGAGACGGTACACCCGCCAGTATGCGGCT
GACGGACGACCCCTTGGCTCCATGTTGCTACAACAGGCTTTCATGGGCTTTCTAAAGTCCTGCACATCTCCTG
TTGAAAATGATCAAACCCCACCGCTGCCAGATGATACCCTTCTCTCCCGATATGCAGACAGTGGTTATGTAGC
TAAAGCCTCAGACCGCGGGATGATTAGCCTGGTAAAATACGCCGCAGAAATCGCGGGTGACCAGATCAATCTC
CTCGAAGAAGAGTCGGATTACCTACAAGTCAGCTCCACTTGGCAACAACGCCTTGCATTCTTGGTCAAGGCTT
TGTCGATAATGTGCTCGATCAACTGCATCATGGTTGACCAGGATTCAGTGGATCTAGACCTGCTCTTCACATC
CCTGGACAATACCATTTCAGATCCAGACCAAATGATGAACAAGGACCTAGCTACCGTTGTCCTTAAGGCTGCT
GTCATTCTAGCAAGGCTATCCCGAGGCAGTGCAAACGGCCTAACCCGCTCTCTTCTTCGCTTCATTGTCCAAA
ATGGCCAAAGCAGTCCTGTCGTCGCACTGGCTGCCCAATGTTTGGCCCAGAACCTCCGGCTTCTTTCCCAAGA
TACCGTCATAGGCACCCTATATTCTCTCGGAAACGTCCTTAGCTCCGGATCGGCAGCTGATAAGGCTGCCACC
GCTCTAACTGCCGAGTCAGGCACTGGCTATTCTCATTCGATAGAGAACCGTGCTCCCCCCACGAATGAGAGCT
TCTTATCCCTTGCTATTGATGGGGAGGGGGATACTTCCGTCACAGCTAGAAATGTGGTACATGCCATTGTAAC
AATCGCCACTTATAGTGAAGATAGCAAGATCGTTGCCCTTGCACAGTCCATTCTGGTGCAGAAGATTGGCAAG

GCGAATGTCATAGTTGATGCATACATTATCCAGGAGACTGCGGCTCTTTCGCTGAAGAGTGAGCCTGCGGAGT
TCCGTCTCTTGCTAAAGTTCTATTCTCGTCTACTGCGAGATACTGTTGCTCAGGGGAATAACCTCATGCTGGA
GGCGGTATGTAGTCCAAGCCCCCCTTTCATCTAGCTGTTCGACCTTGTCAGTGGGAATTCTAACGTATACATA
GATACACAAAGCAAGAAATTACCTTGCAGCCAACCTCGACCGAAACAGTGAAGTCTTCAAGATTTATCTTGTG
TTCCTCCTCGAGAGCTTAGTTAACCAGGGAGCTGCGGTCGAACGCGAAAAGGTTCATCCAGAGGATCTTGAGG
CAACTGCTAAACAAATTGCCCCAATATTGAAACCTCTTGCTATACTACTAGCCCGGGCAGGCCGCTTAGATCA
ATTAGCCTCTGGGGTGTGTGACGATGATGATATCACAGATATGCTCCGGGATGCGTGGTTCAACATCGCAGCC
CATGATATATCACTTCACTCAGAGCTTGGAAAGGCGAACTACGAACAACTACGTATCCTCGCCGAACATTCTC
CTCCGCTAGTCTCCGAGAACCGTGCTGAACTGCTCGAGAGTGACATTGAATTAAATACAATTCTAAGACGCGG
CCAGAGCCCTCAGACAACCGTTGAAAAGAAAAAGAACCTGACTGCTGAACTGCCGGCACAAGAACATGATATC
CGACGCCTGAACTACCCCAAGGCGCTCTTTCTCAATGCAACGCTACTCATAGAAACCCTCCGTGCCAATTCTG
GTGATTGCACCAAAGTGTTCACATACTTCCTCGACCCAACCTTGGGTGCCTCGGAAATGGGCAATTGCATGAG
AGCAATTGCGGACAAGGTTGTGACCGTCTACCTTGAGAAAACTCTCCGAAGCACATCCGTGAAATTCTCGGCC
CCTTATATTGCCAAACAGCTAGCTGATACTTTCACTGCTTGTTGCCATCGTATCATCCAAGTCCAACAAACTG
CTACTCTAGCTGCAAATAAGATAATCATGCAATGCCCGTCAGCACTTTGCCAGAAACTCTCGTTGTTTACTCT
GCTTGATTTGATTACTTTGCTCTGGTCATCGTGCATGGACGAGGAAATGGATGAGTTTGCATGGAACTCTACT
TTCACATCGAGCAGGCGGACGGTCCAGGTTGTTTTATCAGACAATTATGATTTCAGGAGACGTACCTTGAAGA
ATCTCCATAACTCAGCGCGCACTTGGATCTCAAATGTTATGAACATCGCACCATTAGATATCAAGGGGCTACT
GCAGGTGAGTTATTATTCCCACCGATGCCATCAACTTCACTAATATATGTTCTTTCATAGACGTACCTTTCTG
ATTACGATGATGATGGCGTGTACGGCAATATAAACATGGGCCGTTCGTTAGCACTTGAGATGGGCTCAGCAAT
CCCTTTTGCCGACCAGCGACTAGGTATATCAGTTCTTATATCAATAATTTATGCTCTGAGCTTTCATTAACAG
CTGATAGGATCAATTGATAACCCAGGTGGCACTGTTCTCAATGTTGCTTCCGACTTCATTTCTCAGTATACCA
CTCGACAAGAGTACCGGTACTCTGAAGCCGCCCCAGCTCGCAGAGGAAAGTCTCCTCTAGCGATATCCTATGG
TATTTATATGTCTGATTTGAGCCATTGAACCAACCTTCTAATATCTTCAATAGGTCAAAGCACCACCGCCCGG
GTGACTGATTACCCTGACACTGTACAAGAAGCTCTCGCAGCTCTCGAGCATCAAATTAGAACAGAGAAGCATA
TCTCGCTGGATCTTCCTCGGCATATTCTCCGCCGCACTGCTGCTCTCTTGTGTAATACTGATGATAGCCATCC
GGCAATTATTGCTTACCTAGTCAGCATTCCCTTCCAGATATTTACCAAGGAGTCCATCAGGATGGGAATTGCT
CTGTGGCTAGGAGTTATCAATGAAAAACCAGCTATGGAATCCAGAATATTGGTAGAAGTAGCGCAGGCATGGG
AACGTACCATTCGTCGCAAGCAAGGAATCTTCGATCCGAACTTCAAGTAAGCTATCTTCTCTCAGTTTCCTTG
CATCCTGCCCTGACTAACTAACGTTTTTTAGACATAAAAATCCCTTTATGAACAAGCTTGAATCATCCCCATC
CGATAAGGAAGCGATTGTCCGGCGTCATAAGAAAGCCCAAAACATCATATCTCCTCATTTCTTGGTTTTGCAG
TTCCTAGAAAGCCATTTCAGCGCAAGTAGGCTAGGCAGCTTAAATACGCAACAGGTATTTAGACGCCTGGTCA
GTGAGACGTTGGCGGCCTTGAGGGATTCTACTGGCCACCCGCTAGCTCGCGAGATTCACTTCCATATCATCCT
TTTCAGTCTCAAGGTGCTCCATTCATTCTCCGGACAGAGTAATGTTGGTTCGTGGAAGCTCAAAGATCAAATT
CTCTCTGCTGCACTGAGCTGGTTCAACCACTCGCCAAGGTACGTTATCATTTTAATCTCACTTCCTAGGAAGA
TAGCTAACCAAGCATTTTTTAGGTGGTCGTTTGGAGGGAATCGCTTGCAGCTCAAAGCTGAAGATCAGGTCCT
CCACGATATTGAATCTATGTTGGAGACCTTGTCTTGGAAAGAGGTAGTGGACTCAAAGTCTCATAAATCACTA
CAAGCAAAGCACGATCTACTCCTCATTCTGATTGGAAATGAACGCTCTCGACTCAAAGTCTGGCTTTCTCCAC

TAGACCTAGAAAAGAAATCGTACTTCTTCTATGGAACTGGTAACAAAACCGTCACCGAGGTACGTTCCTTACA
TCACTGTTTCGGGCCAACCAGTGGCTAATTATATGCTTTAGGAGACCGTTGCGGGACTTCTTCGATTGGCATG
GAATGAAAACCCAAGCCTAGCACTCCAGCTAGGATCACGGTTCCCATCTGCTAAACTGCGAACTGATATCCGA
TGGCTTCTACTTAACTTCCCGGAAAAAGCACTTAAGGAGCCAAATGCTTTGGAAATTCTACTTGGGTCAGCTC
TTCCAGAGGACGTTTCATTCCAGCTCAAGGTAAGTTTCGCGTTTTACCCCCAGGTGTGTCTTCACTCTCCTAA
CGGCAATGCAGTACCTCCTCTATTGGGAGCCGATCGATCCGATCTCAGCCGTAACTTATCTGTTACCAGCATA
TGGTAACCATCCGTTCATCTTGCAATATGCCCTTCGATCTCTCGAGAGCTTCCCCGTGGATGTTGGCCTTTTC
TATGTTCCGCAGCTCGTGCAGGCCCTTCGCTATGATGCACTGGGATATATAGAACGTTACCTTTCAAATACAG
CTCAGCTGTCACAGATGTTTGCCCACCAAGTCATCTGGAATATGAAGGCAAACGCCTACAAGGACGAAGACTC
TCAGATTGTAAGTACCTTGATTAGTGTTATGGATATCCAAATGTCATGCTAACGAGAAAATAGCCTGATCCAC
TTAAACCGGTGCTGGACCGATTCATGGACTCTGTTATTGCAAGCTTCACGGACGAAGACAAAGAATTCTATGA
GAGGGAATTCGCCTTCTTCAACGAGATCACCGGTATTTCGGGCAAACTACGGCCATACATTAAACGCACCAAA
CAAGAGAAGAAGGAGAAGATTACGGAGGAGCTGCGAAAGATTAAGGTTGAGGTTGGCGTGTACCTGCCAAGTA
ACCCAGATGGGGTTGTTGTTGGCATCGATCGAAAGTCCGGAAAGCCCTTGCAGAGTCACGCAAAGGCACCTTA
TATGGCAACGTTCCGAATCAAGAAGACGAGGAATATTGAAGACGGCAGCAACGCCGTTACTCCTGCCGAAGAG
GCTAGCCATGAGGAAGCGAATTTGCAGCTGTCCCAAGTTAAGGAATCCGCCGTACAAAGCAAACGCGAGAGCC
TAGCTGCGTCTCAAGCGGGGACATATGAGGTATGGCAGTCTGCTATCTTCAAGGTCGGAGATGACTGCCGTCA
AGATGTCCTCGCACTTCAAATGATTGCTGCATTCCGCGAAATATTTGCGAGCGTTGGATTAGACGTATTTGTC
TTCCCGTACCGTGTTGTGGCCACCGCCCCTGGATGCGGCGTGATCGATGTCTTACCCAACTCGATATCTCGAG
ACATGCTGGGCCGTGAAGCTGTCAACGGCCTGTACGACTACTTCGTATCGAAGTACGGAGGAGAGCAGTCGAT
CAAATTCCAAGAAGCCCGAAGCAACTTCGTGAAGAGTATGGCTGCTTATTCAGTCATTTCATACCTGCTGCAG
TTCAAAGACCGACATAACGGCAACATCATGATTGATGACGCAGGACATATCATCCACATTGACTTTGGATTCT
GTTTCGATATTGCGCCTGGTGGTGTACGGTTCGAGCGTGCTCCGTTCAAACTGACCACGGAGATGATTGCTGT
CATGGGAGGATACCATCCGTCTTCGTCAACTGCTGCCTCAGCCACAGCTTCTACAGCTACTACATCCACCACT
CATTCGGCATCGTACAACAACTCTCAATCCTACCGTTGGTTCGAATCACTTGTGGTTAAAGCTTTCCTTGCTT
CGAGACCTTATACTATGAAGCTTTCAAACATCGTAACCCTCATGCTGGACAGCGGTCTTCCTTGCTTCACACC
CCATACGCTGACCAACTTCCGCGACCGATTTGTCCTAGAGAAGAGTGAACAAGAGGCAGCGCAGTATATGAGA
GAACTCATCAGGAAGAGTTACGCCAGCATGTCGACCAAGGGCTATGATCAGTTCCAACTCATGACGAACAACA
TCC

[Read No.m64281e_230107_125321/106759815/ccs] [Sense (+) strand]

AAT~ACC (5'UTR)

ATG~TAG (*chkB* protein kinase genomic DNA) (Forward sequence)

ATG~TGA (*TinCYP51B* gene) (Forward sequence)

TAA~GCT (3'UTR)

CTA~CAT (*FYV4* genomic DNA) (Reverse sequence)

TTAATATTGACGGACCGTTGATAGCTTAACCTGCCATGACTAAGCCGGCCTCCCTGAAGCCGCGATGGCCTGA
TGATGCGCCCACCGTCAGGTCTCGCTCGCTCAGGCAGCAGAGAATCCAGCGGAGGAGATGACGCCCTGCACGA
ACACCGTCCAGGGACCGTCTCCGCCCCGTCGCGAGCTCGAATTGTAATCATCTCGACGGCCGTTGGCCTGCTC
GCCGCAAGGGAGAAAAAGGGGGGCAGAAGGGCAGCGGTAATTTTGGTATTGGAGATGCAGCTGTAAACCAATA
AAAAACGACGTAAAATACCAGGAACAATGCATTATCTTACAGTACCGTACGCCCCTTGCATAAAATGCGAGTT
CGATCTCAAGATGCAACTTTTTTTTTCCCTTGCTTCGTCCGCAGCAGCTTCAAGGCATTCATCACAAACAACG
AACAAGTCTTGAGCGGAAGCAGGAGGATAAGACGATCACC**ATG**GGCCTCTTAGCCGACATTGTCTCTCGTTTC
TGCGAGAACTGCTCGACCCTGTCCACCGCCGCGCTCGTCGCAAGTGCCGTATCGGCTTTTATCGTCCTCTCCA
TTGTTATCAACGTCCTGCAGCAGCTCCTGTTCAAGGACCCTACAAAGCCTCCGGTGGTCTTCCACTGGGTTCC
GGTCATTGGAAGCACGATCTCCTATGGAATTGACCCGTACAAGTTCTTTGACGACTGCAAGGAGAAGGTTAGT
TGGTGCAATTCAACTTAGCTATATACCAGGTTGCGCAAAGCTAACGTATAAATAATTTCGGTACTCTAGTATG
GAGACATCTTCACATTCATACTGCTGGGCAAGAAGACGACTGTTTTTCTCGGTACAAAGGGAAATGATTTCAT
TTTGAACGGCAAGCTCAAGGATGTTTGCGCGGAGGATGTCTACTCCCCCCCTCACCACCCCAGTGTTCGGACG
ACATGTGGTGTATGATTGCCCAAACTCCAAGCTCATGGAGCAGAAGAAGGTGTGTACAGCAATTTATTCCCGT
CTACATCTCCTGGGCCTCCATTGCTAATTTAATCGTCTACCAGTTCGTCAAGTTCGGCCTCACCTCTGAAGCT
CTCCGATCCTATGTCACCCTGATCACCAAGGAAGTTGAGCAGTTCTTCGAGTCCTCCCCCGTCTTCAAGGGCG
ACTCCGGAGTTTTCAACGTCAGCAAGGTCATGGCTGAAATCACCATCTACACCGCCTCTCGATCTCTACAGGG
CAAGGAGGTGCGAGGAAAGTTCGATTCCAGCTTTGCGGAACTCTACTCCGATCTCGACATGGGCTTCGCCGCC
ATCAACTTCATGTTCCCATGGTTCCCCTTCCCACACAACCGCAAGCGTGACCGTGCTCAAAAGAAGATGGCCC
AGGTTTACACCGACATCATCCGTCAGCGACGTGAGGCTGGTGGAGAGAAAGACTCCGAGGACATGGTATGGAA
CTTGATGTCGTCCGTGTACAAGAATGGAACGCCAATTCCAGATATCGAAAGTCGCCCACATGATGATTGCTCT
TCTTATGGCTGGCCAGCACTCTTCTTCCTCCACCGGCTCCTGGATCGTTCTCCGCCTTGCCAGCCGTCCAGAT
ATTCTCGAGGAACTCTACGAGGAACAGAAACGTGTTCTCGGCGAGGATCTTCCACCACTCACCTACGAATCTC
TCCAGAAACTTGATCTTCACAACAATGTAATCAAGGAGACTCTCCGCCTCCACGCTCCCATCCACTCTATCCT
CCGTGCTGTTAAATCCCCTATGCCCGTTGAAGGAACTAACTATGTTGTCCCAACCTCTCACAACCTCCTTGCC
GCTCCTGGTGTTCCCTCACGAGACCCTCAGTACTTCCCTGACCCTCTTGTTTGGAACCCTCACCGATGGGAGA
ACAACGTCGGTGTCACCGTAGTCGAGGCCAGTGAAGAAAAGACAGATTACGGATACGGTTTGGTTAGCAAGGG
TGCCAACAGCCCTTACCTCCCATTCGGCTCAGGCAGACACAGATGCATCGGTGAACAATTTGCATATGTTCAG
CTTGGAACCGTAACAGCTACGTTAGCCAGACTAATGAAATGGAAGCAAGTTGAAGGCACCAAAGATGTTGTGC
CGCCAACTGACTATTCGGTAAGTTTGGCATATTACGCTCCTTTTTATTTTGCTCGATTTTACTAACTTTGCAT
TAATAGTCCCTCTTCTCGAAGCCCCTTGGCGACCCAATGGTCTCGTGGGAGAAGCGAAAGCAGCCTTCCCAGA
AA**TGA**TAATCACGACTGCGGATTGAATCATGTTAAATTTCTTGCTTCTTGCTAATTTATTTCTTCTCTTTTCT
TACGTTTATACACCGAGCCATATTTGCACTTTATGGTCACGCAAATAGGTTTTCGGAATAGAGCATATGTAAT
AAAAACTTTCTTTTGACCATGCTTTTGAAGATGGGGGATGTAGATAATAGTATTATTCTAATACAGCCATTTA
TTACAATACTCTAGATGAACAGGAAAGTTGCAGTTACTCCGTTAGCTTTGGTGCAGAAACAAAATGGTAATAT
ACATTGGAAATAGTACATCTTCCCGTAGCTGGGATAATCTAGCATCTAAACAGCGATATCCAAGCCAGTAATG
TCAGGAAGTAACCAGCCAGCAACGTCGTTAATCCCGTGCCCCCCTTTCAACTCCGACGCTACAAAACGC**CTA**A
GCAGCTGCCTGAGCATTCCTCCTCTCCGCCAACGCTCTCTTCGCTCTGATCTCCTTCTGTCTTCTCTGACCAC

CGTCAATCTTTCTGCCACGCTTATGCTCCCACATGCCATCAGTAACCTGAACCTTGACGGCGGTACTCCATGT
GTTTGGAATAATCTGAGCATACGGCCCCTTGATCATATTCCCGTGGTGCAATTTATAATGGGCAAACTTTTTG
AGTGGCTTGGAAGTATCCTTGAGCTCTGTTTCGCCTGGTGGTAGATTGACAATCGCATAACGCATTCCAGGTG
TAAGGGTAGCGGTGCCCATTGTGCTAACATCTAGGGACTCTCCGTCGGTGGCATTGGTAGTTTTTGCACTCTT
TGACTTTTCGACGGGGACCTCAACAGCTCTCAGCTCAGCAACACCGTCAACAACGTTTTCGAAATCACCTCCT
GGGCCGTAGTCCTGCTGTCTGAATTTCTCTCTCCACCGCAGTATGTATCGTCTGTCCCTTGTCATTTCTATGC
CCAATTCTCGAAGCTCAGCCGAGTCCGCTGTAAACAATTGCTTCCATCCGGTGATTTTGCTGGAATATCTGGA
CATGTTTCGCCCAATTAGGCGTAGGAATGTTTCGACATCGGGCACGAATGGGGTAGGTGATGGCACTGTTGCG
GGTTTTCGAGATTGATGAAGGTACCGTACGCATGGCTGATAATGGGGGTTTGTGACGGAAGTGAAGAGCCGTA
GTGGAAGGGATGTTCTGGCATTAAAGCCCGGTATGCGCGCCGCAGC**CAT**AGTGGTTATATCGCCTTATATCAA
ACCGTTCAATGTCGAGATCTGGGGGGTGAAGATTTTAATTTAGCTCGTGGTGTAAGAACACCATCGGCGGGCG
TGACGAAAGCGAAAAAAGCCACGGCACCAAGGGCCCAGGTAAACCGGTAAGGGACTCAAGGCGAGTCAATCCA
ATGCTCAATGGCGCTGTATAGAAGATGTTCAACTGGTTCTTCGACTTCGTTCAAGATTTTGCACCAAAATTTT
GAATTAGGTCCGACGGCCTTAGCTCGGATCAACAAAACGCCGATCTCCGGTGAGCTCGGGCCAGCCGAAGCTT
CCATCGCAACATTCAAAGATGAAAGACGACGATATATATTTTGATAACAAACAAAGCTGTATTGTTGATTGAA
TACTAGGAGAGCTGCTCAGAGCAGTGTCTCACTCTTGTGCCACTCTATCCTTTGTCTCGCGAAATTGGTGGAA
GCCAATTTCGCTTGACATTATGGCAGTGTCGCCGCTTCCCTCTCAATTCCCTCCCATCACTACTACCGGTGCT
CCAAAGTCGTTAGCGATGAGAAATATTGCTTCTCAAGAGCCTCGGACGGCGCCTAGTGGTGATCCTGCAGTTC
CTAAGCCCTCCATGCCCGAACCAGAGCTGGCGGAGCAGTTAAACGCTGATGTACGGCGGAAATATGTCAAAGG
TGGGCGGGCAAATACACCCCAACCATTGGGACCCCATAATGCTAACATTTGGTTATTCTGAAAGATAAGAAGC
TCGGTGAAGGTACATATGCAGTTGTTTACCTAGGCCATCTACGAGATGATCCCACTTCGCTAATAAAGAGCGA
CTTCGCATCTTTCGACAGAAAAAAAAGGTCGAGCAAGGGAAGTTCGCTCAGCCTGGCCTGCCATTGACTCTAA
GCGCCGTCGTTCTCTTGCTTGGGGGCTTGCTGCAGCTTGGCCTGGGCTTGTCCGGCCTGGAACTCAGGGAGCC
GAAAGATAACTAGTTATTTATTTCGTCTCCGCACACCTCGGCGGCTGTCTCATAGTCGGGATATACATAGTAC
GGGGTAGCAGGTAGACTCACACGCTGCCTCTCTCTGCTGAACTGCTTCTACCACCTTCGACAGCGACACTGTT
CGGTCTTGCAACTACGGTAGAGTGTGTTGGGGCTTGATCTTGCCTGCCATCGACTTGGAGTTGAAGTTGCGAA
GCCATTGGCAGTCCATCCGGGATATTCGTAACTGCCGTCAACTTCTTCACGCCGGGCCAAAAC**ATG**CCTCCGC
AGAGGGAGAGAAGCAATCTGAAGAGAGAAAGGGTAAGATATGAACACGTTATATCCAATTAGTTTCTCAGAAG
GATGAAAATATTCAAGCTGCTAACTTCTTTTCCCAAACCTGCAGGGCAACGCAGCTCCAGACCATGAATCAAA
GAAGCCACGGAGATCTCCTCGAATCTCATCTCAATTGCAGCAGGATGCCGGATATCTACCTTCTCCCCTAGCT
AATGAAGTAATCTCAGAAGAAAAGCGTGAAGTTGCTACAGTTTCTCCCCCAGAAGGCCGCCGAAACCAGGCGC
CTCGAGGTCAATCGCCAGACGAGTCGTCATGTCCCACCCAGGGTTTCTCTCAGTATCCCCCAATCCAATCCAT
TGACGATGTGGAAGACGAGGTGGCCCAGGGCATATGGGGATATCTTACTCCGCTAGATAACAATTTTGGAGGC
CAATTGGTTCTGAAGAAGCGGGACAGCACAAATACTACACCGGTAATTGGAGCAGCATCAGACATATCAACGC
CGGGAAAGCAACGCCTCAAACGTGGAACTAGAGAGATAAACCGCGCTGGATATCTTATAGGCCGGCATGCAGA
GTGCGGTATGCCTTTACCCATTGCATACTCTCTGCCGTCTAGTGATCAATTGCTAACCATCTGGGTACTGCAG
ATTTCAGGATCAAGCTCCCCACGATATCCAACCGCCATTGCCTGATATTCCACGAAAACAGGGGAGGAGACTT
TGTTGCAATTGTGGAAGATCTCTCTATCAATGGCACTTTCATCAACGACGCCATCATCGGCAAGAACAAACGC

CGAGAGCTCGAAAACGGCGACGAGATTACCATCCTGCAAGAATCCAGATTCATCTTCAACTACCCGCGGAGTC
GGAACACAAGCAAGTTCCGACAGCAGTATAAGCTCTTTGATATGCTGGGTAGGGGACACTTTGCCTCGGTTTA
TCTATGCGCTGAACGTTCTACGGGGATCAAGTATGCCGTCAAACATTTCGAGAAACGCCCGGGCGCAGTTCAG
AGGCTAGACCGTGAAGCACTACAGCAAGAGATTTCAATGCTGATGAGTGTCAACCATCCGAACATGCTGTGCC
TAAAGGATACCTTTGACGAGAGCGATGGTGTCTTCCTGATTCTTGAGCTTGCGCCAGAGGGCGAACTGTTCAA
CTGGATCATTAGGCATCAGAAACTCAGCGAGGAGGAGACTCGAAAAGTATTCATTCAGCTTTTTGAAGGTTTG
AGATACCTGGTATGTCGTTCGTTTTCTGGTTGATCAGACTCATGGCAGTGTATATGGCTAACCGTTGTCGTAG
CATGAACGAAACATCATTCACCGTGACATAAAGCCAGAGAACATTCTGGTTGTCGACAAAGATCTTACCGTAA
AATTAGCAGACTTCGGCCTTGCAAAGATTGTCGGCGAACACTCCTTTACCACCACGCTGTAAGTTCACCTACC
TTCTGCCGTTCAAATCTGGGGTCAATCTTACTAAGAGATCATAAAATCTAGATGTGGAACGCCTGGCTGTAAG
TTTCCCTGCTGCTCTGTTAACGCTGGTGCTTTCATCCACCAAATGTGAATCTAACAATTCTTATCAGATGTTG
CTCCTGAGATACTGGCAGAAAACGCTGAAGCCAGAATGTACACCCGAGCGGTTGACATCTGGTCACTCGGAGT
GGTACTCTACATCTGCCTGTGCGGATTCCCTCCTTTCTCAGATGAACTGTACCACCCAAGAGACTACCCATAC
ACTCAGAAAGAGCAGATCCAGCTCGGAATATTTAAGTATCCTCTTCCATACTGGGACTCAATCGATTATAGAG
CCATGGACCTTATCGACTCCATGATCGAAGTCGACGTGAAGGAGCGTTTGAAAGTAGATGAGTGTCTCCAGCA
CCCATGGATCACTGGCATCGACCCCGATGAGCCTAGGGTGGCAGACAGCACCGATGACCTAGCCGGGGCCATC
GGTGGCCTGAGGTGTCCAAAGAACGCCGGCCAACTATGGTCCGCAGGCTGTTGAGTGACATACATGAAGTCAC
GGTCAAGCAGGTGGTGAAGACCAAGGAAGACGACCCTGCTTCCCAGGACATAACTATATACCAGAATCATCCT
GTGCCTCGAGCCCAGGAGCCCAAACCGGCTGGCAACCGGAACTCAAAGGAGTTCATGGAACTCGGTGGAAGTG
GAGACCAGGTGTTATTCGATGAGCAGTCTGACGACTCGCTGGAGCTTGAGAGTGAGGCACGGAAGCCAGTTGT
CCACCAAGACGACGCAGACAAGACTCCACGAGAAAGAAAGAGG**TAG**CAGAAGCCCGATCCGTCTTGGGATGGT
ATTCTGCTAGCTATACCGTGCTATTCCGGACTTTTGTTTCCAATTCCTTTTTCGTTTATATTGATTGTGATCT
TTCTTAATTTTTTCTTTAACTATACCCCCCGGTTGAAAGGTGATAGGGGGCCAACTAACCGAAAAGCAGCAGA
AAAACCGGCTTGGTCGCTGCCTTGGAGACGACGGCGATTCTCGTGTTTAGGATTTCATTTTTGAGTGATATAA
CTATGGGTACAATAGCGTCCACTGGGCAATGTAATTACAGTCTCTTCTCACTCACCACCGGACAATAATAACA
ATATTAATATTGACGGACCGTTGATAGCTTAACCTGCCATGACTAAGCCGGCCTCCCTGAAGCCGCGATGGCC
TGATGATGCGCCCACCGTCAGGTCTCGCTCGCTCAGGCAGCAGAGAATCCAGCGGAGGAGATGACGCCCCTGC
ACGAACACCGTCCAGGGACCGTCTCCGCCCCGTCGCGAGCTCGAATTGTAATCATCTCGACGGCCGTTGGCCT
GCTCGCCGCAAGGGAGAAAAAGGGGGGCAGAAGGGCAGCGGTAATTTTGGTATTGGAGATGCAGCTGTAAACC
AATAAAAAACGACGTAAAATACCAGGAACAATGCATTATCTTACAGTACCGTACGCCCCTTGCATAAAATGCG
AGTTCGATCTCAAGATGCAACTTTTTTTTTCCCTTGCTTCGTCCGCAGCAGCTTCAAGGCATTCATCACAAAC
AACGAACAAGTCTTGAGCGGAAGCAGGAGGATAAGACGATCACC**ATG**GGCCTCTTAGCCGACATTGTCTCTCG
TTTCTGCGAGAACTGCTCGACCCTGTCCACCGCCGCGCTCGTCGCAAGTGCCGTATCGGCTTTTATCGTCCTC
TCCATTGTTATCAACGTCCTGCAGCAGCTCCTGTTCAAGGACCCTACAAAGCCTCCGGTGGTCTTCCACTGGG
TTCCGGTCATTGGAAGCACGATCTCCTATGGAATTGACCCGTACAAGTTCTTTGACGACTGCAAGGAGAAGGT
TAGTTGGTGCAATTCAACTTAGCTATATACCAGGTTGCGCAAAGCTAACGTATAAATAATTTCGGTACTCTAG
TATGGAGACATCTTCACATTCATACTGCTGGGCAAGAAGACGACTGTTTTTCTCGGTACAAAGGGAAATGATT
TCATTTTGAACGGCAAGCTCAAGGATGTTTGCGCGGAGGATGTCTACTCCCCCTCACCACCCCCAGTGTTCGG

ACGACATGTGGTGTATGATTGCCCAAACTCCAAGCTCATGGAGCAGAAGAAGGTGTGTACAGCAATTTATTCC
CGTCTACATCTCCTGGGCCTCCATTGCTAATTTAATCGTTCTACCAGTTCGTCAAGTTCGGCCTCACCTCTGA
AGCTCTCCGATCCTATGTCACCCTGATCACCAAGGAAGTTGAGCAGTTCTTCGAGTCCTCCCCCGTCTTCAAG
GGCGACTCCGGAGTTTTCAACGTCAGCAAGGTCATGGCTGAAATCACCATCTACACCGCCTCTCGATCTCTAC
AGGGCAAGGAGGTGCGAGGAAAGTTCGATTCCAGCTTTGCGGAACTCTACTCCGATCTCGACATGGCTTCGCC
GCCATCAACTTCATGTTCCCATGGTTCCCCTTCCCACACAACCGCAAGCGTGACCGTGCTCAAAAGAAGATGG
CCCAGGTTTACACCGACATCATCCGTCAGCGACGTGAGGCTGGTGGAGAGAAAGACTCCGAGGACATGGTATG
GAACTTGATGTCGTCCGTGTACAAGAATGGAACGCCAATTCCAGATATCGAAGTCGCCCACATGATGATTGCT
CTTCTTATGGCTGGCCAGCACTCTTCTTCCTCCACCGGCTCCTGGATCGTTCTCCGCCTTGCCAGCCGTCCAG
ATATTCTCGAGGAACTCTACGAGGAACAGAAACGTGTTCTCGGCGAGGATCTTCCACCACTCACCTACGAATC
TCTCCAGAAACTTGATCTTCACAACAATGTAATCAAGGAGACTCTCCGCCTCCACGCTCCCATCCACTCTATC
CTCCGTGCTGTTAAATCCCCTATGCCCGTTGAAGGAACTAACTATGTTGTCCCAACCTCTCACAACCTCCTTG
CCGCTCCTGGTGTTCCCTCACGAGACCCTCAGTACTTCCCTGACCCTCTTGTTTGGAACCCTCACCGATGGGA
GAACAACGTCGGTGTCACCGTAGTCGAGGCCAGTGAAGAAAAGACAGATTACGGATACGGTTTGGTTAGCAAG
GGTGCCAACAGCCCTTACCTCCCATTCGGCTCAGGCAGACACAGATGCATCGGTGAACAATTTGCATATGTTC
AGCTTGGAACCGTAACAGCTACGTTAGCCAGACTAATGAAATGGAAGCAAGTTGAAGGCACCAAAGATGTTGT
GCCGCCAACTGACTATTCGGTAAGTTTGGCATATTACGCTCCTTTTTATTTTGCTCGATTTTACTAACTTTGC
ATTAATAGTCCCTCTTCTCGAAGCCCCTTGGCGACCCAATGGTCTCGTGGGAGAAGCGAAAGCAGCCTTCCCA
GAAA**TGA**TAATCACGACTGCGGATTGAATCATGTTAAATTTCTTGCTTCTTGCTAATTTATTTCTTCTCTTTT
CTTACGTTTATACACCGAGCCATATTTGCACTTTATGGTCACGCAAATAGGTTTTCGGAATAGAGCATATGTA
ATAAAAACTTTCTTTTGACCATGCTTTTGAAGATGGGGGATGTAGATAATAGTATTATTCTAATACAGCCATT
TATTACAATACTCTAGATGAACAGGAAAGTTGCAGTTACTCCGTTAGCTTTGGTGCAGAAACAAAATGGTAAT
ATACATTGGAAATAGTACATCTTCCGTAGCTGGGATAATCTAGCATCTAAACAGCGATATCCAAGCCAGTAAT
GTCAGGAAGTAACCAGCCAGCAACGTCGTTAATCCCGTGCCCCCTTTCAACTCCGACGCTACAAAACGC**CTA**A
GCAGCTGCCTGAGCATTCCTCCTCTCCGCCAACGCTCTCTTCGCTCTGATCTCCTTCTGTCTTCTCTGACCAC
CGTCAATCTTTCTGCCACGCTTATGCTCCCACATGCCATCAGTAACCTGAACCTTGACGGCGGTACTCCATGT
GTTTGGAATAATCTGAGCATACGGCCCCTTGATCATATTCCCGTGGTGCAATTTATAATGGGCAAACTTTTTG
AGTGGCTTGGAAGTATCCTTGAGCTCTGTTTCGCCTGGTGGTAGATTGACAATCGCATAACGCATTCCAGGTG
TAAGGGTAGCGGTGCCCATTGTGCTAACATCTAGGGACTCTCCGTCGGTGGCATTGGTAGTTTTTGCACTCTT
TGACTTTTCGACGGGGACCTCAACAGCTCTCAGCTCAGCAACACCGTCAACAACGTTTTCGAAATCACCTCCT
GGGCCGTAGTCCTGCTGTCTGAATTTCTCTCTCCACCGCAGTATGTATCGTCTGTCCCTTGTCATTTCTATGC
CCAATTCTCGAAGCTCAGCCGAGTCCGCTGTAAACAATTGCTTCCATCCGGTGATTTTGCTGGAATATCTGGA
CATGTTTCGCCCAATTAGGCGTAGGAATGTTTCGACATCGGGCACGAATGGGGTAGGTGATGGCACTGTTGCG
GGTTTTCGAGATTGATGAAGGTACCGTACGCATGGCTGATAATGGGGGTTTGTGACGGAAGTGAAGAGCCGTA
GTGGAAGGGATGTTCTGGCATTAAAGCCCGGTATGCGCGCCGCAGC**CAT**AGTGGTTATATCGCCTTATATCAA
ACCGTTCAATGTCGAGATCTGGGGGGTGAAGATTTTAATTTAGCTCGTGGTGTAAGAACACCATCGGCGGGCG
TGACGAAAGCGAAAAAAGCCACGGCACCAAGGGCCCAGGTAAACCGGTAAGGGACTCAAGGCGAGTCAATCCA
ATGCTCAATGGCGCTGTATAGAAGATGTTCAACTGGTTCTTCGACTTCGTTCAAGATTTTGCACCAAAATTTT

GAATTAGGTCCGACGGCCTTAGCTCGGATCAACAAAACGCCGATCTCCGGTGAGCTCGGGCCAGCCGAAGCTT
CCATCGCAACATTCAAAGATGAAAGACGACGATATATATTTTGATAACAAACAAAGCTGTATTGTTGATTGAA
TACTAGGAGAGCTGCTCAGAGCAGTGTCTCACTCTTGTGCCACTCTATCCTTTGTCTCGCGAAATTGGTGGAA
GCCAATTTCGCTTGACATTATGGCAGTGTCGCCGCTTCCCTCTCAATTCCCTCCCATCACTACTACCGGTGCT
CCAAAGTCGTTAGCGATGAGAAATATTGCTTCTCAAGAGCCTCGGACGGCGCCTAGTGGTGATCCTGCAGTTC
CTAAGCCCTCCATGCCCGAACCAGAGCTGGCGGAGCAGTTAAACGCTGATGTACGGCGGAAATATGTCAAAGG
TGGGCGGGCAAATACACCCCAACCATTGGGACCCCATAATGCTAACATTTGGTTATTCTGAAAGATAAGAAGC
TCGGTGAAGGTACATATGCAGTTGTTTACCTAGGCCATCTACGAGATGATCCCACTTCGCTAATAAAGAGCGA
CTTCGCATCTTTCGACAGAAAAAAAAGGTCGAGCAAGGGAAGTTCGCTCAGCCTGGCCTGCCATTGACTCTAA
GCGCCGTCGTTCTCTTGCTTGGGGGCTTGCTGCAGCTTGGCCTGGGCTTGTCCGGCCTGGAACTCAGGGAGCC
GAAAGATAACTAGTTATTTATTTCGTCTCCGCACACCTCGGCGGCTGTCTCATAGTCGGGATATACATAGTAC
GGGGTAGCAGGTAGACTCACACGCTGCCTCTCTCTGCTGAACTGCTTCTACCACCTTCGACAGCGACACTGTT
CGGTCTTGCAACTACGGTAGAGTGTGTTGGGGCTTGATCTTGCCTGCCATCGACTTGGAGTTGAAGTTGCGAA
GCCATTGGCAGTCCATCCGGGATATTCGTAACTGCCGTCAACTTCTTCACGCCGGGCCAAAAC**ATG**CCTCCGC
AGAGGGAGAGAAGCAATCTGAAGAGAGAAAGGGTAAGATATGAACACGTTATATCCAATTAGTTTCTCAGAAG
GATGAAAATATTCAAGCTGCTAACTTCTTTTCCCAAACCTGCAGGGCAACGCAGCTCCAGACCATGAATCAAA
GAAGCCACGGAGATCTCCTCGAATCTCATCTCAATTGCAGCAGGATGCCGGATATCTACCTTCTCCCCTAGCT
AATGAAGTAATCTCAGAAGAAAAGCGTGAAGTTGCTACAGTTTCTCCCCCAGAAGGCCGCCGAAACCAGGCGC
CTCGAGGTCAATCGCCAGACGAGTCGTCATGTCCCACCCAGGGTTTCTCTCAGTATCCCCCAATCCAATCCAT
TGACGATGTGGAAGACGAGGTGGCCCAGGGCATATGGGGATATCTTACTCCGCTAGATAACAATTTTGGAGGC
CAATTGGTTCTGAAGAAGCGGGACAGCACAAATACTACACCGGTAATTGGAGCAGCATCAGACATATCAACGC
CGGAAAGCAACGCCTCAAAACGTGGAACTAGAGAGATAAACCGCGCTGGATATCTTATAGGCCGGCATGCAGA
GTGCGGTATGCCTTTACCCATTGCATACTCTCTGCCGTCTAGTGATCAATTGCTAACCATCTGGGTACTGCAG
ATTTCAGGATCAAGCTCCCCACGATATCCAACCGCCATTGCCTGATATTCCACGAAAACAGGGGAGGAGACTT
TGTTGCAATTGTGGAAGATCTCTCTATCAATGGCACTTTCATCAACGACGCCATCATCGGCAAGAACAAACGC
CGAGAGCTCGAAAACGGCGACGAGATTACCATCCTGCAAGAATCCAGATTCATCTTCAACTACCGCGGAGTCG
GAACACAAGCAAGTTCCGACAGCAGTATAAGCTCTTTGATATGCTGGGTAGGGGACACTTTGCCTCGGTTTAT
CTATGCGCTGAACGTTCTACGGGGATCAAGTATGCCGTCAAACATTTCGAGAAACGCCCGGGCGCAGTTCAGA
GGCTAGACCGTGAAGCACTACAGCAAGAGATTTCAATGCTGATGAGTGTCAACCATCCGAACATGCTGTGCCT
AAAGGATACCTTTGACGAGAGCGATGGTGTCTTCCTGATTCTTGAGCTTGCGCCAGAGGGCGAACTGTTCAAC
TGGATCATTAGGCATCAGAAACTCAGCGAGGAGGAGACTCGAAAAGTATTCATTCAGCTTTTTGAAGGTTTGA
GATACCTGGTATGTCGTTCGTTTTCTGGTTGATCAGACTCATGGCAGTGTATATGGCTAACCGTTGTCGTAGC
ATGAACGAAACATCATTCACCGTGACATAAAGCCAGAGAACATTCTGGTTGTCGACAAAGATCTTACCGTAAA
ATTAGCAGACTTCGGCCTTGCAAAGATTGTCGGCGAACACTCCTTTACCACCACGCTGTAAGTTCACCTACCT
TCTGCCGTTCAAATCTGGGGTCAATCTTACTAAGAGATCATAAAATCTAGATGTGGAACGCCTGGCTGTAAGT
TTCCCTGCTGCTCTGTTAACGCTGGTGCTTTCATCCACCAAATGTGAATCTAACAATTCTTATCAGATGTTGC
TCCTGAGATACTGGCAGAAAACGCTGAAGCCAGAATGTACACCCGAGCGGTTGACATCTGGTCACTCGGAGTG
GTACTCTACATCTGCCTGTGCGGATTCCCTCCTTTCTCAGATGAACTGTACCACCCAAGAGACTACCCATACA

CTCAGAAAGAGCAGATCCAGCTCGGAATATTTAAGTATCCTCTTCCATACTGGGACTCAATCGATTATAGAGC
CATGGACCTTATCGACTCCATGATCGAAGTCGACGTGAAGGAGCGTTTGAAAGTAGATGAGTGTCTCCAGCAC
CCATGGATCACTGGCATCGACCCCGATGAGCCTAGGGTGGCAGACAGCACCGATGACCTAGCCGGGGCCATCG
GTGGCCTGAGGGTGTCCAAAGAACGCCGGCCAACTATGGTCCGCAGGCTGTTGAGTGACATACATGAAGTCAC
GGTCAAGCAGGTGGTGAAGACCAAGGAAGACGACCCTGCTTCCCAGGACATAACTATATACCAGAATCATCCT
GTGCCTCGAGCCCAGGAGCCCAAACCGGCTGGCAACCGGAACTCAAAGGAGTTCATGGAACTCGGTGGAAGTG
GAGACCAGGTGTTATTCGATGAGCAGTCTGACGACTCGCTGGAGCTTGAGAGTGAGGCACGGAAGCCAGTTGT
CCACCAAGACGACGCAGACAAGACTCCACGAGAAAGAAAGAGG**TAG**CAGAAGCCCGATCCGTCTTGGGATGGT
ATTCTGCTAGCTATACCGTGCTATTCCGGACTTTTGTTTCCAATTCCTTTTTCGTTTATATTGATTGTGATCT
TTCTTAATTTTTTCTTTAACTATACCCCCCGGTTGAAAGGTGATAGGGGGCCAACTAACCGAAAAGCAGCAGA
AAAACCGGCTTGGTCGCTGCCTTGGAGACGACGGCGATTCTCGTGTTTAGGATTTCATTTTGAGTGATATAAC
TATGGGTACAATAGCGTCCACTGGGCAATGTAATTACAGTCTCTTCTCACTCACCACCGGACAATAATAACAA
TATTAATATTGACGGACCGTTGATAGCTTAACCTGCCATGACTAAGCCGGCCTCCCTGAAGCCGCGATGGCCT
GATGATGCGCCCACCGTCAGGTCTCGCTCGCTCAGGCAGCAGAGAATCCAGCGGAGGAGATGACGCCCTGCAC
GAACACCGTCCAGGGACCGTCTCCGCCCCGTCGCGAGCTCGAATTGTAATCATCTCGACGGCCGTTGGCCTGC
TCGCCGCAAGGGAGAAAAAGGGGGGCAGAAGGGCAGCGGTAATTTTGGTATTGGAGATGCAGCTGTAAACCAA
TAAAAAACGACGTAAAATACCAGGAACAATGCATTATCTTACAGTACCGTACGCCCCTTGCATAAAATGCGAG
TTCGATCTCAAGATGCAACTTTTTTTTTCCCTTGCTTCGTCCGCAGCAGCTTCAAGGCATTCATCACAAACAA
CGAACAAGTCTTGAGCGGAAGCAGGAGGATAAGACGATCACC**ATG**GGCCTCTTAGCCGACATTGTCTCTCGTT
TCTGCGAGAACTGCTCGACCCTGTCCACCGCCGCGCTCGTCGCAAGTGCCGTATCGGCTTTTATCGTCCTCTC
CATTGTTATCAACGTCCTGCAGCAGCTCCTGTTCAAGGACCCTACAAAGCCTCCGGTGGTCTTCCACTGGGTT
CCGGTCATTGGAAGCACGATCTCCTATGGAATTGACCCGTACAAGTTCTTTGACGACTGCAAGGAGAAGGTTA
GTTGGTGCAATTCAACTTAGCTATATACCAGGTTGCGCAAAGCTAACGTATAAATAATTTCGGTACTCTAGTA
TGGAGACATCTTCACATTCATACTGCTGGGCAAGAAGACGACTGTTTTTCTCGGTACAAAGGGAAATGATTTC
ATTTTGAACGGCAAGCTCAAGGATGTTTGCGCGGAGGATGTCTACTCCCCCCTCACCACCCCAGTGTTCGGAC
GACATGTGGTGTATGATTGCCCAAACTCCAAGCTCATGGAGCAGAAGAAGGTGTGTACAGCAATTTATTCCCG
TCTACATCTCCTGGGCCTCCATTGCTAATTTAATCGTCTACCAGTTCGTCAAGTTCGGCCTCACCTCTGAAGC
TCTCCGATCCTATGTCACCCTGATCACCAAGGAAGTTGAGCAGTTCTTCGAGTCCTCCCCCGTCTTCAAGGGC
GACTCCGGAGTTTTCAACGTCAGCAAGGTCATGGCTGAAATCACCATCTACACCGCCTCTCGATCTCTACAGG
GCAAGGAGGTGCGAGGAAAGTTCGATTCCAGCTTTGCGGAACTCTACTCCGATCTCGACATGGGCTTCGCCGC
CATCAACTTCATGTTCCCATGGTTCCCCTTCCCACACAACCGCAAGCGTGACCGTGCTCAAAAGAAGATGGCC
CAGGTTTACACCGACATCATCCGTCAGCGACGTGAGGCTGGTGGAGAGAAAGACTCCGAGGACATGGTATGGA
ACTTGATGTCGTCCGTGTACAAGAATGGAACGCCAATTCCAGATATCGAAGTCGCCCACATGATGATTGCTCT
TCTTATGGCTGGCCAGCACTCTTCTTCCTCCACCGGCTCCTGGATCGTTCTCCGCCTTGCCAGCCGTCCAGAT
ATTCTCGAGGAACTCTACGAGGAACAGAAACGTGTTCTCGGCGAGGATCTTCCACCACTCACCTACGAATCTC
TCCAGAAACTTGATCTTCACAACAATGTAATCAAGGAGACTCTCCGCCTCCACGCTCCCATCCACTCTATCCT
CCGTGCTGTTAAATCCCCTATGCCCGTTGAAGGAACTAACTATGTTGTCCCAACCTCTCACAACCTCCTTGCC
GCTCCTGGTGTTCCCTCACGAGACCCTCAGTACTTCCCTGACCCTCTTGTTTGGAACCCTCACCGATGGGAGA

ACAACGTCGGTGTCACCGTAGTCGAGGCCAGTGAAGAAAAGACAGATTACGGATACGGTTTGGTTAGCAAGGG
TGCCAACAGCCCTTACCTCCCATTCGGCTCAGGCAGACACAGATGCATCGGTGAACAATTTGCATATGTTCAG
CTTGGAACCGTAACAGCTACGTTAGCCAGACTAATGAAATGGAAGCAAGTTGAAGGCACCAAAGATGTTGTGC
CGCCAACTGACTATTCGGTAAGTTTGGCATATTACGCTCCTTTTTATTTTGCTCGATTTTACTAACTTTGCAT
TAATAGTCCCTCTTCTCGAAGCCCCTTGGCGACCCAATGGTCTCGTGGGAGAAGCGAAAGCAGCCTTCCCAGA
AA**TGA**TAATCACGACTGCGGATTGAATCATGTTAAATTTCTTGCTTCTTGCTAATTTATTTCTTCTCTTTTCT
TACGTTTATACACCGAGCCATATTTGCACTTTATGGTCACGCAAATAGGTTTTCGGAATAGAGCATATGTAAT
AAAAACTTTCTTTTGACCATGCTTTTGAAGATGGGGGATGTAGATAATAGTATTATTCTAATACAGCCATTTA
TTACAATACTCTAGATGAACAGGAAAGTTGCAGTTACTCCGTTAGCTTTGGTGCAGAAACAAAATGGTAATAT
ACATTGGAAATAGTACATCTTCCCGTAGCTGGGATAATCTAGCATCTAAACAGCGATATCCAAGCCAGTAATG
TCAGGAAGTAACCAGCCAGCAACGTCGTTAATCCCGTGCCCCCTTTCAACTCCGACGCTACAAAAACGC**CTA**A
GCAGCTGCCTGAGCATTCCTCCTCTCCGCCAACGCTCTCTTCGCTCTGATCTCCTTCTGTCTTCTCTGACCAC
CGTCAATCTTTCTGCCACGCTTATGCTCCCACATGCCATCAGTAACCTGAACCTTGACGGCGGTACTCCATGT
GTTTGGAATAATCTGAGCATACGGCCCCTTGATCATATTCCCGTGGTGCAATTTATAATGGGCAAACTTTTTG
AGTGGCTTGGAAGTATCCTTGAGCTCTGTTTCGCCTGGTGGTAGATTGACAATCGCATAACGCATTCCAGGTG
TAAGGGTAGCGGTGCCCATTGTGCTAACATCTAGGGACTCTCCGTCGGTGGCATTGGTAGTTTTTGCACTCTT
TGACTTTTCGACGGGGACCTCAACAGCTCTCAGCTCAGCAACACCGTCAACAACGTTTTCGAAATCACCTCCT
GGGCCGTAGTCCTGCTGTCTGAATTTCTCTCTCCACCGCAGTATGTATCGTCTGTCCCTTGTCATTTCTATGC
CCAATTCTCGAAGCTCAGCCGAGTCCGCTGTAAACAATTGCTTCCATCCGGTGATTTTGCTGGAATATCTGGA
CATGTTTCGCCCAATTAGGCGTAGGAATGTTTCGACATCGGGCACGAATGGGGTAGGTGATGGCACTGTTGCG
GGTTTTCGAGATTGATGAAGGTACCGTACGCATGGCTGATAATGGGGGTTTGTGACGGAAGTGAAGAGCCGTA
GTGGAAGGGATGTTCTGGCATTAAAGCCCGGTATGCGCGCCGCAGC**CAT**AGTGGTTATATCGCCTTATATCAA
ACCGTTCAATGTCGAGATCTGGGGGGTGAAGATTTTAATTTAGCTCGTGGTGTAAGAACACCATCGGCGGGCG
TGACGAAAGCGAAAAAAGCCACGGCACCAAGGGCCCAGGTAAACCGGTAAGGGACTCAAGGCGAGTCAATCCA
ATGCTCAATGGCGCTGTATAGAAGATGTTCAACTGGTTCTTCGACTTCGTTCAAGATTTTGCACCAAAATTTT
GAATTAGGTCCGACGGCCTTAGCTCGGATCAACAAAACGCCGATCTCCGGTGAGCTCGGGCCAGCCGAAGCTT
CCATCGCAACATTCAAAGATGAAAGACGACGATATATATTTTGATAACAAACAAAGCTGTATTGTTGATTGAA
TACTAGGAGAGCTGCTCAGAGCAGTGTCTCACTCTTGTGCCACTCTATCCTTTGTCTCGCGAAATTGGTGGAA
GCCAATTTCGCTTGACATTATGGCAGTGTCGCCGCTTCCCTCTCAATTCCCTCCCATCACTACTACCGGTGCT
CCAAAGTCGTTAGCGATGAGAAATATTGCTTCTCAAGAGCCTCGGACGGCGCCTAGTGGTGATCCTGCAGTTC
CTAAGCCCTCCATGCCCGAACCAGAGCTGGCGGAGCAGTTAAACGCTGATGTACGGCGGAAATATGTCAAAGG
TGGGCGGGCAAATACACCCCAACCATTGGGACCCCATAATGCTAACATTTGGTTATTCTGAAAGATAAGAAGC
TCGGTGAAGGTACATATGCAGTTGTTTACCTAGGCCATCTACGAGATGATCCCACTTCGCTAATAAAGAGCGA
CTTCGCATCTTTCGACAGAAAAAAAAGGTCGAGCAAGGGAAGTTCGCTCAGCCTGGCCTGCCATTGACTCTAA
GCGCCGTCGTTCTCTTGCTTGGGGGCTTGCTGCAGCTTGGCCTGGGCTTGTCCGGCCTGGAACTCAGGGAGCC
GAAAGATAACTAGTTATTTATTTCGTCTCCGCACACCTCGGCGGCTGTCTCATAGTCGGGATATACATAGTAC
GGGGTAGCAGGTAGACTCACACGCTGCCTCTCTCTGCTGAACTGCTTCTACCACCTTCGACAGCGACACTGTT
CGGTCTTGCAACTACGGTAGAGTGTGTTGGGGCTTGATCTTGCCTGCCATCGACTTGGAGTTGAAGTTGCGAA

GCCATTGGCAGTCCATCCGGGATATTCGTAACTGCCGTCAACTTCTTCACGCCGGGCCAAAAC**ATG**CCTCCGC
AGAGGGAGAGAAGCAATCTGAAGAGAGAAAGGGGTAAGATATGAACACGTTATATCCAATTAGTTTCTCAGAA
GGATGAAAATATTCAAGCTGCTAACTTCTTTTCCCAAACCTGCAGGGCAACGCAGCTCCAGACCATGAATCAA
AGAAGCCACGGAGATCTCCTCGAATCTCATCTCAATTGCAGCAGGATGCCGGATATCTACCTTCTCCCCTAGC
TAATGAAGTAATCTCAGAAGAAAAGCGTGAAGTTGCTACAGTTTCTCCCCCAGAAGGCCGCCGAAACCAGGCG
CCTCGAGGTCAATCGCCAGACGAGTCGTCATGTCCCACCCAGGGTTTCTCTCAGTATCCCCCAATCCAATCCA
TTGACGATGTGGAAGACGAGGTGGCCCAGGGCATATGGGGATATCTTACTCCGCTAGATAACAATTTTGGAGG
CCAATTGGTTCTGAAGAAGCGGGACAGCACAAATACTACACCGGTAATTGGAGCAGCATCAGACATATCAACG
CCGGAAAGCAACGCCTCAAAACGTGGAACTAGAGAGATAAACCGCGCTGGATATCTTATAGGCCGGCATGCAG
AGTGCGGTATGCCTTTACCCATTGCATACTCTCTGCCGTCTAGTGATCAATTGCTAACCATCTGGGTACTGCA
GATTTCAGGATCAAGCTCCCCACGATATCCAACCGCCATTGCCTGATATTCCACGAAAACAGGGGAGGAGACT
TTGTTGCAATTGTGGAAGATCTCTCTATCAATGGCACTTTCATCAACGACGCCATCATCGGCAAGAACAAACG
CCGAGAGCTCGAAAACGGCGACGAGATTACCATCCTGCAAGAATCCAGATTCATCTTCAACTACCCGCGGAGT
CGGAACACAAGCAAGTTCCGACAGCAGTATAAGCTCTTTGATATGCTGGGTAGGGGACACTTTGCCTCGGTTT
ATCTATGCGCTGAACGTTCTACGGGGATCAAGTATGCCGTCAAACATTTCGAGAAACGCCCGGGCGCAGTTCA
GAGGCTAGACCGTGAAGCACTACAGCAAGAGATTTCAATGCTGATGAGTGTCAACCATCCGAACATGCTGTGC
CTAAAGGATACCTTTGACGAGAGCGATGGTGTCTTCCTGATTCTTGAGCTTGCGCCAGAGGGCGAACTGTTCA
ACTGGATCATTAGGCATCAGAAACTCAGCGAGGAGGAGACTCGAAAAGTATTCATTCAGCTTTTTGAAGGTTT
GAGATACCTGGTATGTCGTTCGTTTTCTGGTTGATCAGACTCATGGCAGTGTATATGGCTAACCGTTGTCGTA
GCATGAACGAAACATCATTCACCGTGACATAAAGCCAGAGAACATTCTGGTTGTCGACAAAGATCTTACCGTA
AAATTAGCAGACTTCGGCCTTGCAAAGATTGTCGGCGAACACTCCTTTACCACCACGCTGTAAGTTCACCTAC
CTTCTGCCGTTCAAATCTGGGGTCAATCTTACTAAGAGATCATAAAATCTAGATGTGGAACGCCTGGCTGTAA
GTTTCCCTGCTGCTCTGTTAACGCTGGTGCTTTCATCCACCAAATGTGAATCTAACAATTCTTATCAGATGTT
GCTCCTGAGATACTGGCAGAAAACGCTGAAGCCAGAATGTACACCCGAGCGGTTGACATCTGGTCACTCGGAG
TGGTACTCTACATCTGCCTGTGCGGATTCCCTCCTTTCTCAGATGAACTGTACCACCCAAGAGACTACCCATA
CACTCAGAAAGAGCAGATCCAGCTCGGAATATTTAAGTATCCTCTTCCATACTGGGACTCAATCGATTATAGA
GCCATGGACCTTATCGACTCCATGATCGAAGTCGACGTGAAGGAGCGTTTGAAAGTAGATGAGTGTCTCCAGC
ACCCATGGATCACTGGCATCGACCCCGATGAGCCTAGGGTGGCAGACAGCACCGATGACCTAGCCGGGGCCAT
CGGTGGCCTGAGGGTGTCCAAAGAACGCCGGCCAACTATGGTCCGCAGGCTGTTGAGTGACATACATGAAGTC
ACGGTCAAGCAGGTGGTGAAGACCAAGGAAGACGACCCTGCTTCCCAGGACATAACTATATACCAGAATCATC
CTGTGCCTCGAGCCAGGAGCCCAAACCGGCTGGCAACCGGAACTCAAAGGAGTTCATGGAACTCGGTGGAAGT
GGAGACCAGGTGTTATTCGATGAGCAGTCTGACGACTCGCTGGAGCTTGAGAGTGAGGCACGGAAGCCAGTTG
TCCACCAAGACGACGCAGACAAGACTCCACGAGAAAGAAAGAGG**TAG**CAGAAGCCCGATCCGTCTTGGGATGG
TATTCTGCTAGCTATACCGTGCTATTCCGGACTTTTGTTTCCAATTCCTTTTTCGTTTATATTGATTGTGATC
TTTCTTAATTTTTTCTTTAACTATACCCCCGGTTGAAAGGTGATAGGGGGCCAACTAACCGAAAAGCAGCAGA
AAAACCGGCTTGGTCGCTGCCTTGGAGACGACGGCGATTCTCGTGTTTAGGATTTCATTTTTGAGTGATATAA
CTATGGGTACAATAGCGTCCACTGGGCAATGTAATTACAGTCTCTTCTCACTCACCACCGGACAATAATAACA
ATATTAATATTGACGGACCGTTGATAGCTTAACCTGCCATGACTAAGCCGGCCTCCCTGAAGCCGCGATGGCC

TGATGATGCGCCCACCGTCAGGTCTCGCTCGCTCAGGCAGCAGAGAATCCAGCGGAGGAGATGACGCCCTGCA
CGAACACCGTCCAGGGACCGTCTCCGCCCCGTCGCGAGCTCGAATTGTAATCATCTCGACGGCCGTTGGCCTG
CTCGCCGCAAGGGAGAAAAAGGGGGGGCAGAAGGGCAGCGGTAATTTTGGTATTGGAGATGCAGCTGTAAACC
AATAAAAAACGACGTAAAATACCAGGAACAATGCATTATCTTACAGTACCGTACGCCCCTTGCATAAAATGCG
AGTTCGATCTCAAGATGCAACTTTTTTTTTCCCTTGCTTCGTCCGCAGCAGCTTCAAGGCATTCATCACAAAC
AACGAACAAGTCTTGAGCGGAAGCAGGAGGATAAGACGATCACC**ATG**GGCCTCTTAGCCGACATTGTCTCTCG
TTTCTGCGAGAACTGCTCGACCCTGTCCACCGCCGCGCTCGTCGCAAGTGCCGTATCGGCTTTTATCGTCCTC
TCCATTGTTATCAACGTCCTGCAGCAGCTCCTGTTCAAGGACCCTACAAAGCCTCCGGTGGTCTTCCACTGGG
TTCCGGTCATTGGAAGCACGATCTCCTATGGAATTGACCCGTACAAGTTCTTTGACGACTGCAAGGAGAAGGT
TAGTTGGTGCAATTCAACTTAGCTATATACCAGGTTGCGCAAAGCTAACGTATAAATAATTTCGGTACTCTAG
TATGGAGACATCTTCACATTCATACTGCTGGGCAAGAAGACGACTGTTTTTCTCGGTACAAAGGGAAATGATT
TCATTTTGAACGGCAAGCTCAAGGATGTTTGCGCGGAGGATGTCTACTCCCCCCTCACCACCCCAGTGTTCGG
ACGACATGTGGTGTATGATTGCCCAAACTCCAAGCTCATGGAGCAGAAGAAGGTGTGTACAGCAATTTATTCC
CGTCTACATCTCCTGGGCCTCCATTGCTAATTTAATCGTCTACCAGTTCGTCAAGTTCGGCCTCACCTCTGAA
GCTCTCCGATCCTATGTCACCCTGATCACCAAGGAAGTTGAGCAGTTCTTCGAGTCCTCCCCCCGTCTTCAAG
GGCGACTCCGGAGTTTTCAACGTCAGCAAGGTCATGGCTGAAATCACCATCTACACCGCCTCTCGATCTCTAC
AGGGCAAGGAGGTGCGAGGAAAGTTCGATTCCAGCTTTGCGGAACTCTACTCCGATCTCGACATGGGCTTCGC
CGCCATCAACTTCATGTTCCCATGGTTCCCCTTCCCACACAACCGCAAGCGTGACCGTGCTCAAAAGAAGATG
GCCCAGGTTTACACCGACATCATCCGTCAGCGACGTGAGGCTGGTGGAGAGAAAGACTCCGAGGACATGGTAT
GGAACTTGATGTCGTCCGTGTACAAGAATGGAACGCCAATTCCAGATATCGAAGTCGCCCACATGATGATTGC
TCTTCTTATGGCTGGCCAGCACTCTTCTTCCTCCACCGGCTCCTGGATCGTTCTCCGCCTTGCCAGCCGTCCA
GATATTCTCGAGGAACTCTACGAGGAACAGAAACGTGTTCTCGGCGAGGATCTTCCACCACTCACCTACGAAT
CTCTCCAGAAACTTGATCTTCACAACAATGTAATCAAGGAGACTCTCCGCCTCCACGCTCCCATCCACTCTAT
CCTCCGTGCTGTTAAATCCCCTATGCCCGTTGAAGGAACTAACTATGTTGTCCCAACCTCTCACAACCTCCTT
GCCGCTCCTGGTGTTCCCTCACGAGACCCTCAGTACTTCCCTGACCCTCTTGTTTGGAACCCTCACCGATGGG
AGAACAACGTCGGTGTCACCGTAGTCGAGGCCAGTGAAGAAAAGACAGATTACGGATACGGTTTGGTTAGCAA
GGGTGCCAACAGCCCTTACCTCCCATTCGGCTCAGGCAGACACAGATGCATCGGTGAACAATTTGCATATGTT
CAGCTTGGAACCGTAACAGCTACGTTAGCCAGACTAATGAAATGGAAGCAAGTTGAAGGCACCAAAGATGTTG
TGCCGCCAACTGACTATTCGGTAAGTTTGGCATATTACGCTCCTTTTTATTTTGCTCGATTTTACTAACTTTG
CATTAATAGTCCCTCTTCTCGAAGCCCCTTGGCGACCCAATGGTCTCGTGGGAGAAGCGAAAGCAGCCTTCCC
AGAAA**TGA**TAATCACGACTGCGGATTGAATCATGTTAAATTTCTTGCTTCTTGCTAATTTATTTCTTCTCTTT
TCTTACGTTTATACACCGAGCCATATTTGCACTTTATGGTCACGCAAATAGGTTTTCGGAATAGAGCATATGT
AATAAAAACTTTCTTTTGACCATGCTTTGAAGATGGGGATGTAGATAATAGTATTATTCTAATACAGCCATT
TATTACAATACTCTAGATGAACAGGAAAGTTGCAGTTACTCCGTTAGCTTTGGTGCAGAAACAAAATGGTAAT
ATACATTGGAAATAGTACATCTTCCCGTAGCTGGGATAATCTAGCATCTAAACAGCGATATCCAAGCCAGTAA
TGTCAGGAAGTAACCAGCCAGCAACGTCGTTAATCCGTGCCCCCTTTCAACTCCGACGCTACAAAACGC**CTA**A
GCAGCTGCCTGAGCATTCCTCCTCTCCGCCAACGCTCTCTTCGCTCTGATCTCCTTCTGTCTTCTCTGACCAC
CGTCAATCTTTCTGCCACGCTTATGCTCCCACATGCCATCAGTAACCTGAACCTTGACGGCGGTACTCCATGT

GTTTGGAATAATCTGAGCATACGGCCCCTTGATCATATTCCGTGGTGCAATTTATAATGGGCAAACTTTTTGA
GTGGCTTGGAAGTATCCTTGAGCTCTGTTTCGCCTGTGGTAGATTGACAATCGCATAACGCATTCCAGGTGTA
AGGGTAGCGGTGCCCATTGTGCTAACATCTAGGGACTCTCCGTCGGTGGCATTGGTAGTTTTTGCACTCTTTG
ACTTTTCGACGGGGACCTCAACAGCTCTCAGCTCAGCAACACCGTCAACAACGTTTCGAAATCACCTCCTGGG
CCGTAGTCCTGCTGTCTGAATTTCTCTCTCCACCGCAGTATGTATCGTCTGTCCCTTGTCATTTCTATGCCCA
ATTCTCGAAGCTCAGCCGAGTCCGCTGTAAACAATTGCTTCCATCCGGTGATTTTGCTGGAATATCTGGACAT
GTTTCGCCCAATTAGGCGTAGGAATGTTTCGACATCGGGCACGAATGGGGTAGGTGATGGCACTGTTGCGGGT
TTTCGAGATTGATGAAGGTACCGTACGCATGGCTGATAATGGGGGTTTGTGACGGAAGTGAAGAGCCGTAGTG
GAAGGGATGTTCTGGCATTAAAGCCCGGTATGCGCGCCGCAGC**CAT**AGTGGTTATATCGCCTTATATCAAACC
GTTCAATGTCGAGATCTGGGGGGTGAAGATTTTAATTTAGCTCGTGGTGTAAGAACACCATCGGCGGGCGTGA
CGAAAGCGAAAAAAGCCACGGCACCAAGGGCCCAGGTAAACCGGTAAGGGACTCAAGGCGAGTCAATCCAATG
CTCAATGGCGCTGTATAGAAGATGTTCAACTGGTTCTTCGACTTCGTTCAAGATTTTGCACCAAAATTTTGAA
TTAGGTCCGACGGCCTTAGCTCGGATCAACAAAACGCCGATCTCCGGTGAGCTCGGGCCAGCCGAAGCTTCCA
TCGCAACATTCAAAGATGAAAGACGACGATATATATTTTGATAACAAACAAAGCTGTATTGTTGATTGAATAC
TAGGAGAGCTGCTCAGAGCAGTGTCTCACTCTTGTGCCACTCTATCCTTTGTCTCGCGAAATTGGTGGAAGCC
AATTTCGCTTGACATTATGGCAGTGTCGCCGCTTCCCTCTCAATTCCCTCCATCACTACTACCGGTGCTCAAA
GTCGTTAGCGATGAGAAATATTGCTTCTCAAGAGCCTCGGACGGCGCCTAGTGGTGATCCTGCAGTTCCTAAG
CCCTCCATGCCCGAACCAGAGCTGGCGGAGCAGTTAAACGCTGATGTACGGCGGAAATATGTCAAAGGTGGGC
GGGCAAATACACCCCAACCATTGGGACCCCATAATGCTAACATTTGGTTATTCTGAAAGATAAGAAGCTCGGT
GAAGGTACATATGCAGTTGTTTACCTAGGCCATCTACGAGATGATCCCACTTCGCTAATAAAGAGCGACTTCG
CATCTTTCGACAGAAAAAAAAGGTCGAGCAAGGGAAGTTCGCTCAGCCTGGCCTGCCATTGACTCTAAGCGCC
GTCGTTCTCTTGCTTGGGGGCTTGCTGCAGCTTGGCCTGGGCTTGTCCGGCCTGGAACTCAGGGAGCCGAAAG
ATAACTAGTTATTTATTTCGTCTCCGCACACCTCGGCGGCTGTCTCATAGTCGGGATATACATAGTACGGGGT
AGCAGGTAGACTCACACGCTGCCTCTCTCTGCTGAACT

[Read No.m64281e_230107_125321/89260242/ccs] [Sense (+) strand]

AAT~ACC (5'UTR)

ATG~TAG (*chkB* protein kinase genomic DNA) (Forward sequence)

ATG~TGA (*TinCYP51B* gene) (Forward sequence)

TAA~GCT (3'UTR)

CTA~CAT (*FYV4* genomic DNA) (Reverse sequence)

CGT~ (Downstream sequence of the 7374 block)

CTCCTGGGCCGTAGTCCTGCTGTCTGAATTTCTCTCTCCACCGCAGTATGTATCGTCTGTCCCTTGTCATTTC
TATGCCCAATTCTCGAAGCTCAGCCGAGTCCGCTGTAAACAATTGCTTCCATCCGGTGATTTTGCTGGAATAT
CTGGACATGTTTCGCCCAATTAGGCGTAGGAATGTTTCGACATCGGGCACGAATGGGGTAGGTGATGGCACTG
TTGCGGGTTTTCGAGATTGATGAAGGTACCGTACGCATGGCTGATAATGGGGGTTTGTGACGGAAGTGAAGAG

CCGTAGTGGAAGGGATGTTCTGGCATTAAAGCCCGGTATGCGCGCCGCAGC**CAT**AGTGGTTATATCGCCTTAT
ATCAAACCGTTCAATGTCGAGATCTGGGGGGTGAAGATTTTAATTTAGCTCGTGGTGTAAGAACACCATCGGC
GGGCGTGACGAAAGCGAAAAAAGCCACGGCACCAAGGGCCCAGGTAAACCGGTAAGGGACTCAAGGCGAGTCA
ATCCAATGCTCAATGGCGCTGTATAGAAGATGTTCAACTGGTTCTTCGACTTCGTTCAAGATTTTGCACCAAA
ATTTTGAATTAGGTCCGACGGCCTTAGCTCGGATCAACAAAACGCCGATCTCCGGTGAGCTCGGGCCAGCCGA
AGCTTCCATCGCAACATTCAAAGATGAAAGACGACGATATATATTTTGATAACAAACAAAGCTGTATTGTTGA
TTGAATACTAGGAGAGCTGCTCAGAGCAGTGTCTCACTCTTGTGCCACTCTATCCTTTGTCTCGCGAAATTGG
TGGAAGCCAATTTCGCTTGACATTATGGCAGTGTCGCCGCTTCCCTCTCAATTCCCTCCCATCACTACTACCG
GTGCTCCAAAGTCGTTAGCGATGAGAAATATTGCTTCTCAAGAGCCTCGGACGGCGCCTAGTGGTGATCCTGC
AGTTCCTAAGCCCTCCATGCCCGAACCAGAGCTGGCGGAGCAGTTAAACGCTGATGTACGGCGGAAATATGTC
AAAGGTGGGCGGGCAAATACACCCCAACCATTGGGACCCCATAATGCTAACATTTGGTTATTCTGAAAGATAA
GAAGCTCGGTGAAGGTACATATGCAGTTGTTTACCTAGGCCATCTACGAGATGATCCCACTTCGCTAATAAAG
AGCGACTTCGCATCTTTCGACAGAAAAAAAAGGTCGAGCAAGGGAAGTTCGCTCAGCCTGGCCTGCCATTGAC
TCTAAGCGCCGTCGTTCTCTTGCTTGGGGGCTTGCTGCAGCTTGGCCTGGGCTTGTCCGGCCTGGAACTCAGG
GAGCCGAAAGATAACTAGTTATTTATTTCGTCTCCGCACACCTCGGCGGCTGTCTCATAGTCGGGATATACAT
AGTACGGGGTAGCAGGTAGACTCACACGCTGCCTCTCTCTGCTGAACTGCTTCTACCACCTTCGACAGCGACA
CTGTTCGGTCTTGCAACTACGGTAGAGTGTGTTGGGGCTTGATCTTGCCTGCCATCGACTTGGAGTTGAAGTT
GCGAAGCCATTGGCAGTCCATCCGGGATATTCGTAACTGCCGTCAACTTCTTCACGCCGGGCCAAAAC**ATG**CC
TCCGCAGAGGGAGAGAAGCAATCTGAAGAGAGAAAGGGTAAGATATGAACACGTTATATCCAATTAGTTTCTC
AGAAGGATGAAAATATTCAAGCTGCTAACTTCTTTTCCCAAACCTGCAGGGCAACGCAGCTCCAGACCATGAA
TCAAAGAAGCCACGGAGATCTCCTCGAATCTCATCTCAATTGCAGCAGGATGCCGGATATCTACCTTCTCCCC
TAGCTAATGAAGTAATCTCAGAAGAAAAGCGTGAAGTTGCTACAGTTTCTCCCCCAGAAGGCCGCCGAAACCA
GGCGCCTCGAGGTCAATCGCCAGACGAGTCGTCATGTCCCACCCAGGGTTTCTCTCAGTATCCCCCAATCCAA
TCCATTGACGATGTGGAAGACGAGGTGGCCCAGGGCATATGGGGATATCTTACTCCGCTAGATAACAATTTTG
GAGGCCAATTGGTTCTGAAGAAGCGGGACAGCACAAATACTACACCGGTAATTGGAGCAGCATCAGACATATC
AACGCCGGAAAGCAACGCCTCAAAACGTGGAACTAGAGAGATAAACCGCGCTGGATATCTTATAGGCCGGCAT
GCAGAGTGCGGTATGCCTTTACCCATTGCATACTCTCTGCCGTCTAGTGATCAATTGCTAACCATCTGGGTAC
TGCAGATTTCAGGATCAAGCTCCCCACGATATCCAACCGCCATTGCCTGATATTCCACGAAAACAGGGGAGGA
GACTTTGTTGCAATTGTGGAAGATCTCTCTATCAATGGCACTTTCATCAACGACGCCATCATCGGCAAGAACA
AACGCCGAGAGCTCGAAAACGGCGACGAGATTACCATCCTGCAAGAATCCAGATTCATCTTCAACTACCCGCG
GAGTCGGAACACAAGCAAGTTCCGACAGCAGTATAAGCTCTTTGATATGCTGGGTAGGGGACACTTTGCCTCG
GTTTATCTATGCGCTGAACGTTCTACGGGGATCAAGTATGCCGTCAAACATTTCGAGAAACGCCCGGGCGCAG
TTCAGAGGCTAGACCGTGAAGCACTACAGCAAGAGATTTCAATGCTGATGAGTGTCAACCATCCGAACATGCT
GTGCCTAAAGGATACCTTTGACGAGAGCGATGGTGTCTTCCTGATTCTTGAGCTTGCGCCAGAGGGCGAACTG
TTCAACTGGATCATTAGGCATCAGAAACTCAGCGAGGAGGAGACTCGAAAAGTATTCATTCAGCTTTTTGAAG
GTTTGAGATACCTGGTATGTCGTTCGTTTTCTGGTTGATCAGACTCATGGCAGTGTATATGGCTAACCGTTGT
CGTAGCATGAACGAAACATCATTCACCGTGACATAAAGCCAGAGAACATTCTGGTTGTCGACAAAGATCTTAC
CGTAAAATTAGCAGACTTCGGCCTTGCAAAGATTGTCGGCGAACACTCCTTTACCACCACGCTGTAAGTTCAC

CTACCTTCTGCCGTTCAAATCTGGGGTCAATCTTACTAAGAGATCATAAAATCTAGATGTGGAACGCCTGGCT
GTAAGTTTCCCTGCTGCTCTGTTAACGCTGGTGCTTTCATCCACCAAATGTGAATCTAACAATTCTTATCAGA
TGTTGCTCCTGAGATACTGGCAGAAAACGCTGAAGCCAGAATGTACACCCGAGCGGTTGACATCTGGTCACTC
GGAGTGGTACTCTACATCTGCCTGTGCGGATTCCCTCCTTTCTCAGATGAACTGTACCACCCAAGAGACTACC
CATACACTCAGAAAGAGCAGATCCAGCTCGGAATATTTAAGTATCCTCTTCCATACTGGGACTCAATCGATTA
TAGAGCCATGGACCTTATCGACTCCATGATCGAAGTCGACGTGAAGGAGCGTTTGAAAGTAGATGAGTGTCTC
CAGCACCCATGGATCACTGGCATCGACCCCGATGAGCCTAGGGTGGCAGACAGCACCGATGACCTAGCCGGGG
CCATCGGTGGCCTGAGGGTGTCCAAAGAACGCCGGCCAACTATGGTCCGCAGGCTGTTGAGTGACATACATGA
AGTCACGGTCAAGCAGGTGGTGAAGACCAAGGAAGACGACCCTGCTTCCCAGGACATAACTATATACCAGAAT
CATCCTGTGCCTCGAGCCCAGGAGCCCAAACCGGCTGGCAACCGGAACTCAAAGGAGTTCATGGAACTCGGTG
GAAGTGGAGACCAGGTGTTATTCGATGAGCAGTCTGACGACTCGCTGGAGCTTGAGAGTGAGGCACGGAAGCC
AGTTGTCCACCAAGACGACGCAGACAAGACTCCACGAGAAAGAAAGAGG**TAG**CAGAAGCCCGATCCGTCTTGG
GATGGTATTCTGCTAGCTATACCGTGCTATTCCGGACTTTTGTTTCCAATTCCTTTTTCGTTTATATTGATTG
TGATCTTTCTTAATTTTTTCTTTAACTATACCCCCCGGTTGAAAGGTGATAGGGGGCCAACTAACCGAAAAGC
AGCAGAAAAACCGGCTTGGTCGCTGCCTTGGAGACGACGGCGATTCTCGTGTTTAGGATTTCATTTTTGAGTG
ATATAACTATGGGTACAATAGCGTCCACTGGGCAATGTAATTACAGTCTCTTCTCACTCACCACCGGACAATA
ATAACAATATTAATATTGACGGACCGTTGATAGCTTAACCTGCCATGACTAAGCCGGCCTCCCTGAAGCCGCG
ATGGCCTGATGATGCGCCCACCGTCAGGTCTCGCTCGCTCAGGCAGCAGAGAATCCAGCGGAGGAGATGACGC
CCTGCACGAACACCGTCCAGGGACCGTCTCCGCCCCGTCGCGAGCTCGAATTGTAATCATCTCGACGGCCGTT
GGCCTGCTCGCCGCAAGGGAGAAAAAGGGGGGCAGAAGGGCAGCGGTAATTTTGGTATTGGAGATGCAGCTGT
AAACCAATAAAAAACGACGTAAAATACCAGGAACAATGCATTATCTTACAGTACCGTACGCCCCTTGCATAAA
ATGCGAGTTCGATCTCAAGATGCAACTTTTTTTTTCCCTTGCTTCGTCCGCAGCAGCTTCAAGGCATTCATCA
CAAACAACGAACAAGTCTTGAGCGGAAGCAGGAGGATAAGACGATCACC**ATG**GGCCTCTTAGCCGACATTGTC
TCTCGTTTCTGCGAGAACTGCTCGACCCTGTCCACCGCCGCGCTCGTCGCAAGTGCCGTATCGGCTTTTATCG
TCCTCTCCATTGTTATCAACGTCCTGCAGCAGCTCCTGTTCAAGGACCCTACAAAGCCTCCGGTGGTCTTCCA
CTGGGTTCCGGTCATTGGAAGCACGATCTCCTATGGAATTGACCCGTACAAGTTCTTTGACGACTGCAAGGAG
AAGGTTAGTTGGTGCAATTCAACTTAGCTATATACCAGGTTGCGCAAAGCTAACGTATAAATAATTTCGGTAC
TCTAGTATGGAGACATCTTCACATTCATACTGCTGGGCAAGAAGACGACTGTTTTTCTCGGTACAAAGGGAAA
TGATTTCATTTTGAACGGCAAGCTCAAGGATGTTTGCGCGGAGGATGTCTACTCCCCCCTCACCACCCCAGTG
TTCGGACGACATGTGGTGTATGATTGCCCAAACTCCAAGCTCATGGAGCAGAAGAAGGTGTGTACAGCAATTT
ATTCCCGTCTACATCTCCTGGGCCTCCATTGCTAATTTAATCGTCTACCAGTTCGTCAAGTTCGGCCTCACCT
CTGAAGCTCTCCGATCCTATGTCACCCTGATCACCAAGGAAGTTGAGCAGTTCTTCGAGTCCTCCCCCGTCTT
CAAGGGCGACTCCGGAGTTTTCAACGTCAGCAAGGTCATGGCTGAAATCACCATCTACACCGCCTCTCGATCT
CTACAGGGCAAGGAGGTGCGAGGAAAGTTCGATTCCAGCTTTGCGGAACTCTACTCCGATCTCGACATGGGCT
TCGCCGCCATCAACTTCATGTTCCCATGGTTCCCCTTCCCACACAACCGCAAGCGTGACCGTGCTCAAAAGAA
GATGGCCCAGGTTTACACCGACATCATCCGTCAGCGACGTGAGGCTGGTGGAGAGAAAGACTCCGAGGACATG
GTATGGAACTTGATGTCGTCCGTGTACAAGAATGGAACGCCAATTCCAGATATCGAAGTCGCCCACATGATGA
TTGCTCTTCTTATGGCTGGCCAGCACTCTTCTTCCTCCACCGGCTCCTGGATCGTTCTCCGCCTTGCCAGCCG

TCCAGATATTCTCGAGGAACTCTACGAGGAACAGAAACGTGTTCTCGGCGAGGATCTTCCACCACTCACCTAC
GAATCTCTCCAGAAACTTGATCTTCACAACAATGTAATCAAGGAGACTCTCCGCCTCCACGCTCCCATCCACT
CTATCCTCCGTGCTGTTAAATCCCCTATGCCCGTTGAAGGAACTAACTATGTTGTCCCAACCTCTCACAACCT
CCTTGCCGCTCCTGGTGTTCCCTCACGAGACCCTCAGTACTTCCCTGACCCTCTTGTTTGGAACCCTCACCGA
TGGGAGAACAACGTCGGTGTCACCGTAGTCGAGGCCAGTGAAGAAAAGACAGATTACGGATACGGTTTGGTTA
GCAAGGGTGCCAACAGCCCTTACCTCCCATTCGGCTCAGGCAGACACAGATGCATCGGTGAACAATTTGCATA
TGTTCAGCTTGGAACCGTAACAGCTACGTTAGCCAGACTAATGAAATGGAAGCAAGTTGAAGGCACCAAAGAT
GTTGTGCCGCCAACTGACTATTCGGTAAGTTTGGCATATTACGCTCCTTTTTATTTTGCTCGATTTTACTAAC
TTTGCATTAATAGTCCCTCTTCTCGAAGCCCCTTGGCGACCCAATGGTCTCGTGGGAGAAGCGAAAGCAGCCT
TCCCAGAAA**TGA**TAATCACGACTGCGGATTGAATCATGTTAAATTTCTTGCTTCTTGCTAATTTATTTCTTCT
CTTTTCTTACGTTTATACACCGAGCCATATTTGCACTTTATGGTCACGCAAATAGGTTTTCGGAATAGAGCAT
ATGTAATAAAAACTTTCTTTTGACCATGCTTTTGAAGATGGGGGATGTAGATAATAGTATTATTCTAATACAG
CCATTTATTACAATACTCTAGATGAACAGGAAAGTTGCAGTTACTCCGTTAGCTTTGGTGCAGAAACAAAATG
GTAATATACATTGGAAATAGTACATCTTCCCGTAGCTGGGATAATCTAGCATCTAAACAGCGATATCCAAGCC
AGTAATGTCAGGAAGTAACCAGCCAGCAACGTCGTTAATCCCGTGCCCCCTTTCAACTCCGACGCTACAAAAC
GC**CTA**AGCAGCTGCCTGAGCATTCCTCCTCTCCGCCAACGCTCTCTTCGCTCTGATCTCCTTCTGTCTTCTCT
GACCACCGTCAATCTTTCTGCCACGCTTATGCTCCCACATGCCATCAGTAACCTGAACCTTGACGGCGGTACT
CCATGTGTTTGGAATAATCTGAGCATACGGCCCCTTGATCATATTCCCGTGGTGCAATTTATAATGGGCAAAC
TTTTTGAGTGGCTTGGAAGTATCCTTGAGCTCTGTTTCGCCTGGTGGTAGATTGACAATCGCATAACGCATTC
CAGGTGTAAGGGTAGCGGTGCCCATTGTGCTAACATCTAGGGACTCTCCGTCGGTGGCATTGGTAGTTTTTGC
ACTCTTTGACTTTTCGACGGGGACCTCAACAGCTCTCAGCTCAGCAACACCGTCAACAACGTTTTCGAAATCA
CCTCCTGGGCCGTAGTCCTGCTGTCTGAATTTCTCTCTCCACCGCAGTATGTATCGTCTGTCCCTTGTCATTT
CTATGCCCAATTCTCGAAGCTCAGCCGAGTCCGCTGTAAACAATTGCTTCCATCCGGTGATTTTGCTGGAATA
TCTGGACATGTTTCGCCCAATTAGGCGTAGGAATGTTTCGACATCGGGCACGAATGGGGTAGGTGATGGCACT
GTTGCGGGTTTTCGAGATTGATGAAGGTACCGTACGCATGGCTGATAATGGGGGTTTGTGACGGAAGTGAAGA
GCCGTAGTGGAAGGGATGTTCTGGCATTAAAGCCCGGTATGCGCGCCGCAGC**CAT**AGTGGTTATATCGCCTTA
TATCAAACCGTTCAATGTCGAGATCTGGGGGGTGAAGATTTTAATTTAGCTCGTGGTGTAAGAACACCATCGG
CGGGCGTGACGAAAGCGAAAAAAGCCACGGCACCAAGGGCCCAGGTAAACCGGTAAGGGACTCAAGGCGAGTC
AATCCAATGCTCAATGGCGCTGTATAGAAGATGTTCAACTGGTTCTTCGACTTCGTTCAAGATTTTGCACCAA
AATTTTGAATTAGGTCCGACGGCCTTAGCTCGGATCAACAAAACGCCGATCTCCGGTGAGCTCGGGCCAGCCG
AAGCTTCCATCGCAACATTCAAAGATGAAAGACGACGATATATATTTTGATAACAAACAAAGCTGTATTGTTG
ATTGAATACTAGGAGAGCTGCTCAGAGCAGTGTCTCACTCTTGTGCCACTCTATCCTTTGTCTCGCGAAATTG
GTGGAAGCCAATTTCGCTTGACATTATGGCAGTGTCGCCGCTTCCCTCTCAATTCCCTCCCATCACTACTACC
GGTGCTCCAAAGTCGTTAGCGATGAGAAATATTGCTTCTCAAGAGCCTCGGACGGCGCCTAGTGGTGATCCTG
CAGTTCCTAAGCCCTCCATGCCCGAACCAGAGCTGGCGGAGCAGTTAAACGCTGATGTACGGCGGAAATATGT
CAAAGGTGGGCGGGCAAATACACCCCAACCATTGGGACCCCATAATGCTAACATTTGGTTATTCTGAAAGATA
AGAAGCTCGGTGAAGGTACATATGCAGTTGTTTACCTAGGCCATCTACGAGATGATCCCACTTCGCTCGTTGC
AATCAAGAAAATAAAACTAAACGCCGAGTACAAGGACGGGCTATCCATGGACGCCATCCGTGAAGTAAAGTAC

CTTCAGGAACTGTCTCACCCGAACGTAATTGCGTTGCATGACGTTTTCTCCTCAAAGGATCAAAATCTAAACC
TCGTCCTTGAATTTTTACCCTTGGAGATCTTGAAATGTTGATCAAAGACAACTCTATCCAGTACGGTGTGGCT
GATATAAAAGCATGGATAAGCATGCTTGCACGTGGAGTGTGGTTCTGTCATAAGAATTTCATTCTACACCGTG
ATATTAAGCCTAACAACTTATTAATTGCATCGGATGGAGAAGTTAAATTAGCGGATTTTGGTCTTGCCAGATC
CTTTGCCGATCCATATCTCAACATGACACATCAAGTCATCACAAGATGGTACAGGCCATTAGAACTACTCTTC
GGCGCCAGGCAATATTCCGGTGCAGTCGACATATGGTCTATGGGAATGGTGTTCGCTGAGCTTATTCTGCGCG
TTCCATTCGCCGCTGGAAACACGGATATGGACCAAATATCTAAAATTTGTGCTGCATTTGGGGCACCTACGGA
AGAAAACTGGCCTGGGGTGACAAAGCTGCCCAACTACGTCCCAATCGAGGAAAACCATGTCATACCCCTACAA
GGCCGCGAATTCTTCCTCCGCCAATTTCCGACAGCTGGACCTTTGGGGGCCGATCTACTTGCCTCCATGTTAA
AACTTGATCCAAGGAAACGAATTACGGCCACTCAAGTCTTGCAGCATCAGTGGTGGCTTGCTGAGCCTAAACC
AACTAAGAACGAGGACCTGCCTAGGAAATCAGGAGGAGAAAAGAAAATGGGCAACGATATGGGCAGACGAGTT
GGTGAGATTGACGAGTCAAAATTTAAAGGAGTGTCCCGTCAACTTGATTTCGGAGCTATGAAGAAATGAGAGG
ATCCGACTTGCCATCCGGAATATAATACTCAAAAATTATCGCCTCCTTGGTTAGCAAGCGAGCAGATATCGCG
TACCGACAAATATATGCTACAAGAAGAAGTGATACACAACATTCTGGGCCGCTCCTCTTTGTGGGTGCAATGC
TGTGTAAACATCGGCCTCAATGAGAGCCCAATTTGGTTAATGAGCTGCAGCCTTACCAATACGGAGGAGACGT
CAGTAGCAGGCCCCTATACCACACTCAGCAGGAAGCCGATACGCGTAAATCTATGCTTTCATGAGCCAGGCAG
TTCCCTGATGGCCACAAACCAGGCGGCATTGTTTGACCTTGGGGTTGATCTATCAAAAAGGGGGAATATCTCC
TAATTTGAAGCCCACCCTCAAAGTAATGGGTTGAAATTCAAAAAGCAGCACCCTACTTGATAACGCCTACCCT
CTTTAGTACTGCAGGATTCATTGTGTACTTGACATCATATATAATCTGGTAGAATAGGCCTTGGGCGTGCCAT
GCATAGAGAGAGACGTTGATGATATATGGTTTCAAAGTGATTCTATGGGAGTTACCGCTTTCCAACTTAAAAG
CCTTTGGCTAGCCGTAAAACACCTAACCATTCGGTCACTTTTTCGGTTGAAGGGGCTCCCACGAAGTTACTCT
CGTTCCTGCATCTGAATCCATGAGCTCGTTCGTTGGTAGTGATTTGGTAGCTTGTGATACGGATGAGGTAGTG
GCTGCGCCAGCCCCGCCAGTATATGCTTGTTCTTGGAAATCCTCGGCAGGGTTAACCATTTCGTATCCACTCG
AGACACCATTACTTGAGGCGGTTTTGCGATCGGTTCCGCCTGATGAATGTCGTCTCAATTTTGCTGGTTTCCT
AGGTACCGGAGGTGGTATTTTTACCGATTTTACAGTTCTGCTGCTGACGACAACCTTAGAGGTTTGAGGCATG
TCTGTGGCAGACGATTCAGGACCGCCCATAACTTCTCCCACCGATCTAAATGGCTCTCTGTATGTGGAGGATG
CCTTTTTCGACTCTCGTAGGTCCTGCTCGCTCATTTCGCTCAAAGCATCATCTCTTCCGACAGTAATCCAATC
CGACCCCCGAGAAAATGAAAAAGGATTGGCACTTCGCCGAACATTGCTCACAGCACCAAGTGGTGGAGTAATA
GTTGATCGAGCAGGAAGGCCTATAAAATTGTTAGCTTTAACATATCAATGCTAAAGAGCCGACTAGTTAACTA
AGTCAAATATACTCACCATGATCAAGCCACCACTTACGCTTTTCGGAGCTGGGAGGTGGAAGGCCTAGCTTTA
TCGAGTATTGTTCATTATCATCTTCCTCATCATCAGACAACGGGTTTGTAGTTGTGGTGGCAGGTTGTTGCTT
CAGCTTTTCATAAAGTCGACAGCTTATTTCTTCCTTTCGCTTCTCATCTACCGTCGATATATCGCACTCGAAT
GTTGCATAAACAGGTCGATGGTCCGAAAATTTCAAAGGTGCAGTCGTATAGGCTAGCTGTCGTAGAATCCTTC
CTCTCCAAAGGATCCTGTCGCACCAGGCGGGAATCCTTTGCTTTTCTCTAGAAGTATTATTAGTATTTACAGG
TCTAAATGGATGGACTGCCTCTCAAACTTACGAGGTGTCATATTCATCTGTGCCATTGTCGTATCTATATGTT
GGAGGGAATGTGACAGGCCCTTCCATATAATACTGGAATGCAAGGCCAGCAACCATTTGGAGGTTGAGCTATT
GAAAGTTAGTAGTAGTCAGGGCATGTATTCGCGCATCAAGAGACATACCTGGTCATGCTGATATAGTACATCT
ATATCTCCCGATTCAATTAGAGGCCTCACCCGCTCATTGCTAAGGCCAATCCTATAGTTGAAATCACCGAACC

AGATTGTTGCATCATGTCCGACAATAGGTCTGTTTCGTTGGAACCTGAGACCCCGAGCTATGGTGTGGTAGTC
TCTGTTTCTTTCCTCGTAGTTCGAGAAGCCAGCAGCTAGATGAGCAGTGATGAAGCATATTGACGTATTACTA
TAGTCCAAGCGGATAGCAACAGCACCCTTATTCCCTCCCATGCCAGATAAGCCAGTCTGTTACTCAGTCCAGT
TAGCCCTGCTTCTCAAAAGATATTATATTCAAAGGATGGATACCTTCTTCAAGCTGCCTTCCGCATTCTTGAT
CTTATTTATAACATCCTTCTTTACGAACAGCAAGAGTGCTGCACCCACTAGCTGACCACTTCTCAGCAAAACG
TACTCAGTCGTCCCTCTCTTCGTGGTCCTGGCATTGAGTGATGATATAACTGCGTGTTCCCATATCTTGCGCG
TGCTTGGGTCAGTGGACATAATCTGCTGGGGGCTAAGCTCAACAATCTCTTGAAATCCAACAGCAACTATGGT
AGGATCCTCAGGAAGTTGGTTAAGAGAGCCGTGCAACCATGGACCCAGTCCTTCAGTAGCTCCTTCACGACGT
CCGTTGATGTTAAATGTTCCGACCCATATATAGATCTTTTCAGATGAGGTAAACTCGTTTACTCTCCGGGTAA
GCTCAGCATTCACTGCATCTTTCACCGGATCAAACAGATACACCGACTCCTGATTATTCGAAAGGCCTAGAAG
GGTATCTATAGTGATTTGTTTCGCTGGGTCGGTAAAGTTGTTAACGAAGAGGCGAGTCGCACTCTTCCTTATG
TCTGCAAAGGAACCAGCAACTGACATTGCTCCATGTCTGGTAAACGAAGACTTCAAGGCTCCAGTTCCAGCAT
ATATTCTTGACAACATATCTCCGCTATCAGCCCATAGAGTCGAGTGCTTCATCCAGAAATCCGGTGACGCATG
TCCATTCCACTGGGCAAAAAACATGTCAATTGCCATGCTACTAATCATGGTTTGGACAAGATTAGTCCTGTCC
AAGCAGTCCAGACAATTTGTTCGAAAGACACCTTCCTGTTGTAGTACAATGTTCTTTTGTCTGAGAAGTCTGA
GTTCTGCTGCTGATAGATCCTTTTCATTGCCCTGAGGTGCCTCGGATAGGAAATAGGCAAATCCTTCGACAGA
GTCTGCTATCACATCCTGAATGGAACGGGCAGCCTCATACCCCGTTGTTTTCGTTTCAGCATGGAAGTCGTAT
TCTGTTAGTTTCAATATATCATGCTCTGAGTTCGAATTTACATCACGATTCTGGCGCAAGGGACTGGCTCGAA
CATGATACATATATCTTTGCGACAGCTCCACTTCTCCAGACTTTGCTTTGGCAAGCAGGTTAATTGCATGCAC
CGCCCCATATTTCTCAGCTAAAGCTTCAAAGTGTTTATCAAATGCGGGCTGCGTAGCGCCTGAAGAGCGGCCG
ATGGCAATTTTCTGCTGTCCAGGTATATAACCAGCTTCCTGTTCCCAGAATATGGGTACAGAACCTCGAATCT
GGGTATAAGAAAAGCACATGGCAGGAGGTATCCATAGGATGGTTTCTGTCTCAACAAAGTTGGCAACGTTTCC
ATTGTCGTCAATTCCCCTGGCGTTGAATCTAGTTCCGGCACGGCGAGAGGAAAGGCGCGATATCACTGTTAAG
CTAGAGGGCATATTAGATTCAATGTGTGGGAACAGAGGTGATGAAGCAGGTATGGTTAAGGACTGAGCGAAGC
CCCTAATGACGGATATAAGAAGCTGAGATGAGTCTAGGTTCTGCCTATCATTCAGACTCAGTCCACTTCTGAG
CTGTAGTAAAGGCTTGATCATGAATGAATTCCACAGGAAATCTTCATCCTGGCTCTCAACATCGTAAGCAACC
GGGTCATCAATTCTTGCGAAAAAAAAAATTACGGAATTAGCTGATGTCAATTTCAGTCTGTTGAGGACACGCG
TTCTTACCTGTCCTGAATGCGTTCGGTCAAATTAAAGTCAGAGCTGTAATAGAAGCTTCCGTCACTCAGAAGC
TTTTTCAATGCTAGAGATGGATTCTCTGCAGGGGATTCGTATCCTTGGTAACCTCTTCCGGAAGGGTTATTAT
ACGTATCAGAGTCAAGATTCTGGACCAGGTCATAATCGGATCTATTCAAGCAATCTGTATTCTATCTGTCAGC
CTCATCCGATCAATGGAATGCCGCAAGAATTAGGAGGCTAGAGTTCTCGTACAAAAGTCAACACTGTCTATCC
TTTGGACTGTTTCTCCAGGCCTCACGCTGGCGACATTGGTTGCTTGGTTGACGATGCATATAAAAACCTCCCC
AGCCGCGAGCGCGATGAGACCGAGCGTTCCAAGTCCAGTTCCCAACGTGCGATACTCAGCCAGGGGTATCGAA
TCGGCCGTAGTAAACTCGACAATACATTTTGGAATGCTCAGGACCTGTACGTTCCCGGATACTTCTTCGCTTG
ACTGGTGATGGCGCATGGTGAGGGCGTAGCCATTTGCCACGAGGACAACTGTGCGGTCCGGCGCATCCTTGCA
AAGGACTCGCACAGCCATCGCTCCAGGTAGATTTTTTTTTTTTTTTTTTTTTTTTTTTTGGGCTAGAATATAG
CGGTTATAGCGAAGACTGCGAGCGCATGGGCTGTCTGTTCAGCAGAGGTATAGCTGCATATCCTGTTGCGCAG
AGAGCTGTGTCCAGTGGTTGCATGTTTTTCCTTCACCCATCCGCGGCACCAGAAACGTCGCAAGTGAATTTCC

CTTTTATTTCCCCTGCTATTTGCCTTTCCTCCTAGCAAGTGACAAATATCACAGGAAGGCGTAAGTGACGGCT
GCAAGTTTATATATCTTTGGGTTCAAAAAGTAGGATGCTGCTTCCAGAGTTCCAGCTGGATTTTGATAGTTAT
AAAATTTGTTAGTTAACTAACTAGTTAGTTTTTCTGTTGGGAAAAAAGGTGAAAAGAAAAGGGCCTAAAAGGA
AGCAATTTAAACATGATGTAGCTGAAGACAGGCAACTTTTCTTTATTTAGTCTTAAAGGAAGTGTTTGGATAA
GAAGATGGAGAGTTCCAGTTCACGTTATACTCAGTCGATAATAGTAATAGGAAATACACAACACTGCAGTAAC
TGTATTAATTTGAGGTGTATTATTATATTCTATAAGAAAGCAGGCACATCTGGTCAAAGACGGCCTTTTCAGA
CAGTTGTTTGATAATAAAAGATAGCAATCAATCAGTCAGTCAGTTGGTCAGTCAGTTGGCCACTGTAGAAACA
AACGCCATTGTTTTCCCTCTTCCTCTATGTTCTACTTTATTCCTTAGATGAACAATGCAACAATAAAGACAGC
TGCTCCATCCTTCTTCTCACTCCTGGAGAGTTTCGATATTTTCTTGGAGCGTGGCCTGTACGGTTCGGTTTGC
TTCCTTGTACTTCTCCTCCAGCGATAGCACACGCTTAGCTATCTCATTTTCAACCTCGAGTCTTCTCTTTACC
AGTTCCTGGAGCTGGTCAAGCTGTGCATTACGATATTCCAGACTATTTAACAGGTCAATAAACCAGCGATGAA
AGAGTCTTTGAGTGGGAATTATTGGATACGCAACCCGGGCACAATACTAGAGGTAGAAATCTAAATTCAAGTA
CAGCACCTACATGGCTTGCTTGTTCGATCGAGAGACTTCATTTAACCTGTTCTCAATCTTTCTGACACCCGCT
TTGTGTCGAGCTTGAATCTCCTGGCATCCGTCATTAGCTGACTTCCGTGCTGCTACCTGGAGAGGCAAAGAGA
CATTGGTTAATACCCGGGAAGCTAGTATCGCCTTCGCAGACATCAACGAAACAAGCAGAGCTAATGACAGCAT
TTGCTACTCATGGAATTGTTTACCTTCTTGGGTGGATTGGTCATTTCTGCGCCTGGATCGGCAGATATCCCGC
TCTCGCTCTCCGCCGTGTTTGCCCTTCGGCTGCTTCTGGCGCGCGTATTTGCTTGTCCCCGTTTTTGTGATTG
TTTGGCAGCCATGTTTTACCCCCTCTGACGACCGGTTTCAAGGATATGCAACAGGATACTCAAACTCCTTTGA
GCTGCCTCTGAGCGCCTTTGGATCCCTTTTTGATTCTGTCAAATCTCCTGCAGGTTGAAGACATTGATTGAAT
AGCGCTGGGAAGTCCAATATTACCCAAAAGGGACAAACCAAAACAAAGTTATTACATAATATATTATGTAATT
CTGTTATTGCGGCGTCACTTTCTGCTTGAGTCGTAAACAGCGATATTGACATCCTCGAGGACGACGACGACAT
GGAAGATACCAGTTTAACCTACATGGTTCAAGCAATTGTACTCTTGTTTGTAGTATTTCCTGGGAATAAACGT
TGCTGTTGGGAGAGAGACAGACCACACTCCTCACCTACAAACACTACTTAAAGCGGCAACATGGGAATATCTT
ATCAAACTTCGACCTTTTTTTTTTTTTCTGAGGAAATAAAAAAAAAATAAAATCGATTTCGTCTATAGCTTTTAT
CCAGTAGACACAGTCCTCCGACCGTCACAATGGGTCTAGCCGCTCCTAAGAAGTAAGTCTACCGACTGCCACT
GTCAATTAATGCCATAGTGCCAATTTAACCGTTTATCAGGAGGACCAAAATTTCAAATGACCCGAATAACACA
ACGTGGACTCGATCAACGTCCGGCTATGGACACAAAATCATGACCTCCCAGGGCTGGGCGCCTGGCGACTTTT
TGGGTGCAGCAAACAGTAACCGGACCGATACATATACCGCTGCCAGCTTTGGCCACATCCGAGTATCTCTCAA
AGACGATAACCTAGGGCTGGGGGCTAAACCCCGCCGGCCACTGATAGATGATGAGCCCACCGGATTAGACGCA
TTCCAGGGCCTGCTGGGGCGACTCAATGGAAGGAGTGAAGTTGAAATAGAAAAGGAAATGAAGGTTAAACGGG
ATATCAAAGCCATGACTTATATTGAGCGGAGATGGGGTTGCATGAACTTCATCGGTGGCGGACTCCTGGTACC
AGACAAGGTCAATAAAATACCAAACGAAGAAGAAAATAAGAGCGCAGATGGCCCTGCAGATGCTAAGCCTGCT
GAGGGATCGGTGAACGAAGATATAGATACGAAGGAAAAGAGGAAGCGGGACAAGAAGGAGAAAAAGGAACGAA
AAGAGAAGAAAGAGAAATCCAAGGAGAAGAAGAAGCGGAAAAAGGAAACGCAAGAGTCCGAGGAAATTGCAGA
TAGCGGATTTGCAACGGAGATATCTGCTCCCGCTTCAAGAGCGGAGACCTCTGACGATGCCGAGACCGATTCA
AGGACCGAGAAACAAAAGTCGAAGGATAAGAAGAAATCGAAAAAGCGGAAACGAAAAGAAGAAGCGCAAGCTG
ATAAGACTGAAACTTCATCAAAACCTAGTGAAGAAGATACAAAGGACACCAAGAGAGTGGAGGCATCCCCATC
AGCACCAGCACCACCTATTGCTGTCAAGGAAAGGGTTATCCCTATTTCTCGACAGCTTCTCCGAGGACGGTAT

ATCCAGCAAAAGAGACGAGCAGTCATGGACTCCAAGTCATTGAACGAGGTATTATATCCTGCTACCTGTTACA
TCAAGACCTTCTAGCTGACAAAAAGACAGATATTTATGACCACAAGTTGATACCATCTACTTTGCCCAGATCT
CCCCCACCTGGACAGGCTCCTTTTTCAATATTCTCTGTTATAGATCCAACTAAAAAGTGACTAGATGATCTTA
TATACCCATAAAGCGCTATAATTCTACCTTATTTCTGAAAATTTGCTTTTTTTTTGGTATCTCGATTCTATCT
ATCTATACCTATGTTGAATATTTTTTGAAATGCCTGGAATCATCTTTACAGACGCATTTTTGACCTGAGAAGA
AGAGTCTATAAAACTTCTTTACACTTCACTGGTTGCCATGACGTCTCTGTTATAGTTTAACATAGACCTTGGA
TATATTCACCCTCCCTTTTTTTTTCGCTGCCTACTATATTCCCAACTTCAGGTTAGGTTTTTAATGAATGGGC
CTTTAAAGCATTAACTTATATATCACTGCTTTATCCTGAGTAGTGGAAATTGAGAGTGATGTCAGCACCTGTC
CTGCTCCTAGGCTCTTTGTGCAGTAACCAGTCTCCTAGGCTTGGTTCTTGACCATGATTGACTCTGAAAGAGC
TGGCTTGAGCCAGATTGAGGCAGCTTTATCTGAGTTGAATGTGGAGGATGCTGCTCACTTGACTCCCGCTCTC
GACTTTGAAGGCGCCCTGCGCGAAAGACTCCAATATACTAGCGTTGAGCTCTACGCTGTTGACGGCGACGGCA
TGATCCGCGTCCCGGCAGTTCTCTTCTGTGGTGATGCAAACAAGGTATCTACTCTGCTGCCTTTTTGTTTTTC
TCTCGAGTATTTGGGCCACATAGACGCGCAAGGCCTTATTATACTCACTTTGAGCAATAGGGGCTTCCAGGGA
TCATCCTTACTCCAACTACTCCATCTGAACCTGAGACTCCCAGCACGGTATCATCTTCGCCAACATCGCTGGA
ATCACCAGCAATGCCCTCCCTGCCGTCGCCGATAACGCCGCCGCCTCCCACTCCACCAGGGTTTGAGTTCAAA
AACAATACTGAACGGTACTTCTTTGCCATTGCTGCACGCGCACTCCAGGAGGCAACAAAATTACCTGAACAAC
CACCACCAGCTGGCTGGCTACCTAGCCAGAAATGCCCTATCTTCTATTCTTCGATACTTGACCAATTTACTGT
CTGTTTCCGCGAGAGACTCCAAAGCCTAGGCCGCTACCAACAGGACACATTCATTGAAGAATGTCAACATCAC
TATGTCAGCATTTTCAACAACATCCGCTCTAGATGGCATTACTCCCCGTTCGGCTCGATTGATGGTTTCAGAA
CTACCATCAATACTCCCAACTGTGCAATTGCTGTCCAAGCACTGCAACACGCAGTCAGAAAGCCGACTTCCCC
TCCGCCAGCTAACTGGAAGGTCAACGATCCCTTACACACATACTATGTTATCATGATTGCTAGGTTCCGTACC
CATCTGCGCAACATGCTGAGGAACTACGACTCCCTCTTGATCGATGAACCAATTGATATCCACGAGTCTACCT
GCCGATTCATCGAGTTAGTTATTCAAGAGTACCGCCGCACCTGGATCACAAACTACGGGCTAGAGTGAAGGAG
TAAATTGTCTGTTTCATTATCTCTTAAACCTGTCGAAAGGGGTTAAACAAAAAAAAAAAAAAAAAAAAAAGAG
TAAAAGGAATAAAAGGAGGCTCATGCATGTGAATAAATAGACGCGAGTAACAAAGTTCAATAGCCGCGATATG
AAGTCAAGCTATTACGTCACCACAAGAGCAAAGCCCAACCTCGTAGTTAGAAACAAGCGCTGCTTCACCTTCA
CCAACGCCATCCCTTCTACGCATCTTCACTCAGTAGTAGCTAGGCGACAGTCTATCCTTCGTTCTCGAGTCAC
TTTAATCTTCACCTGCGGCTCGGGGACGCTCGCATATATCTCTGAGCCCAAGAAGACGACTTCCAACCTCGCG
CCCCCAAACTGCGATCCTGAAACACCCGCACGAGACATCACGGAAGCACCGCCCTCGATCCACCGGTCCAAAT
AAACGCATCTACGTCACCGTATCCCCATGCCCGACATTGATAGAGTGTTCGCTGAAATGAAGGGTGAAGCGGG
GGAAAACTATTCGGGCTCTATGCGACAGTTCCGGAGCCTAGTAAACTCGGCTCCAGAGTCGCTATTCAATGAC
TCCTCTTCTACGGGCAGTGTAGCAAGCGCTATGCCTAGCTCTCTGCCGGCTCGAAGAAGCCATGGCCTAGTCA
GGACGAGGTCAGACCCGTTCAAGCCGAATACTACGACAACTCTCTTACCTCAGTCTGAGAGTGACTATACTCA
GCGTATGGGGTACGTTTGCCTGGGTCAACCCGCAGGGAAATTGGTTAGCGAAACTAACAGACAGTTCCTTTTT
GTTATGATAGAATTCGATGGGCTCCGTTGAATATCAGTCTTAAACGGCGGCTCCAGACCCTCGTCGTCTCCTT
CATACCATTTCGATCGCATTTTGCTTGATGTCTTTCTTTTTCTGCTGCGCCATCCCCTTATTCTGGCCCATCC
TATTCCCATACGTGGTTTACATATCACTGTTTTCCAAGGCTGCCACAGATGGAAGACTTTCAAGGAGAAGTAA
CTTCATTCGATCCCTACCCATATGGAAGCTTTTTGGCTCGTACTTCCCGGCTCGCCTTCACCGAACAGTCCTC

TCCCACCAACTAGGAAGTATATTTTTGGCTACCACCCACATGGGATTATATCACATGGAGCATTCGCGGCTTT
CGCTACTGAAGGGCTAGGCTTTTCAAAGCTCTTCCCGGGAATCACAAATACCCTACTTACCCTGGACTCCAAT
TTCCGACTTCCTTTCTACCGAGACTGGGCATTAGCAATGGGATTAGGTAGCGTATCCCGTGAATCCTGCGAAA
ATATCCTTTCAAAGGGTGGAATGAATAAGGAAGGCATGGGCCGTGCAATTACCATTGTGATAGGTGGTGCACG
AGAATCTCTTGATGCCCAGCCGCATACCCTCCGATTGATCCTGCGCCGACGAAAGGGGTTCGTCAAGCTTGCA
ATCCGCACTGGCGCAGACCTTGTTCCCGTACTCGCATTTGGTGAAAACGAGCT
