# Supplementary material for: Two different types of tandem sequences mediate the overexpression of TinCYP51B in azole-resistant Trichophyton indotineae
Source: Antimicrob Agents Chemother. 2023 Oct 12;67(11):e00933-23. doi: 10.1128/aac.00933-23 (PMC10648874; doi:10.1128/aac.00933-23)
Supplement: NGS raw sequence reads — Nucleotide sequence of the TinCYP51B locus in TIMM20123. [file aac.00933-23-s0003.pdf]

***TinCYP51B* locus in TIMM20123**

[Read No.m64281e_230311_112734/12322574/ccs] [Sense (+) strand]

~TAT (Upstream sequence of the 7374 block)

AAT~ACC (5'UTR)

ATG~TAG (*chkB* protein kinase genomic DNA) (Forward sequence)

ATG~TGA (*TinCYP51B* gene) (Forward sequence)

TAA~GCT (3'UTR)

CTA~CAT (*FYV4* genomic DNA) (Reverse sequence)

GAAGATAGCTTACTTGAAGTTCGGATCGAAGATTCCTTGCTTGCGACGAATGGTACGTTCCCATGCCTGCGCT
ACTTCTACCAATATTCTGGATTCCATAGCTGGTTTTTCATTGATAACTCCTAGCCACAGAGCAATTCCCATCC
TGATGGACTCCTTGGTAAATATCTGGAAGGGAATGCTGACTAGGTAAGCAATAATTGCCGGATGGCTATCATC
AGTATTACACAAGAGAGCAGCAGTGCGGCGGAGAATATGCCGAGGAAGATCCAGCGAGATATGCTTCTCTGTT
CTAATTTGATGCTCGAGAGCTGCGAGAGCTTCTTGTACAGTGTCAGGGTAATCAGTCACCCGGGCGGTGGTGC
TTTGACCTATTGAAGATATTAGAAGGTTGGTTCAATGGCTCAAATCAGACATATAAATACCATAGGATATCGC
TAGAGGAGACTTTCCTCTGCGAGCTGGGGCGGCTTCAGAGTACCGGTACTCTTGTCGAGTGGTATACTGAGAA
ATGAAGTCGGAAGCAACATTGAGAACAGTGCCACCTGGGTTATCAATTGATCCTATCAGCTGTTAATGAAAGC
TCAGAGCATAAATTATTGATATAAGAACTGATATACCTAGTCGCTGGTCGGCAAAAGGGATTGCTGAGCCCAT
CTCAAGTGCTAACGAACGGCCCATGTTTATATTGCCGTACACGCCATCATCATCGTAATCAGAAAGGTACGTC
TATGAAAGAACATATATTAGTGAAGTTGATGGCATCGGTGGGAATAATAACTCACCTGCAGTAGCCCTTGATA
TCTAATGGTGCGATGTTCATAACATTTGAGATCCAAGTGCGCGCTGAGTTATGGAGATTCTTCAAGGTACGTC
TCCTGAAATCATAATTGTCTGATAAAACAACCTGGACCGTCCGCCTGCTCGATGTGAAAGTAGAGTTCCATGC
AAACTCATCCATTTCCTCGTCCATGCACGATGACCAGAGCAAAGTAATCAAATCAAGCAGAGTAAACAACGAG
AGTTTCTGGCAAAGTGCTGACGGGCATTGCATGATTATCTTATTTGCAGCTAGAGTAGCAGTTTGTTGGACTT
GGATGATACGATGGCAACAAGCAGTGAAAGTATCAGCTAGCTGTTTGGCAATATAAGGGGCCGAGAATTTCAC
GGATGTGCTTCGGAGAGTTTTCTCAAGGTAGACGGTCACAACCTTGTCCGCAATTGCTCTCATGCAATTGCCC
ATTTCCGAGGCACCCAAGGTTGGGTCGAGGAAGTATGTGAACACTTTGGTGCAATCACCAGAATTGGCACGGA
GGGTTTCTATGAGTAGCGTTGCATTGAGAAAGAGCGCCTTGGGGTAGTTCAGGCGTCGGATATCATGTTCTTG
TGCCGGCAGTTCAGCAGTCAGGTTCTTTTTCTTTTCAACGGTTGTCTGAGGGCTCTGGCCGCGTCTTAGAATT
GTATTTAATTCAATGTCACTCTCGAGCAGTTCAGCACGGTTCTCGGAGACTAGCGGAGGAGAATGTTCGGCGA
GGATACGTAGTTGTTCGTAGTTCGCCTTTCCAAGCTCTGAGTGAAGTGATATATCATGGGCTGCGATGTTGAA
CCACGCATCCCGGAGCATATCTGTGATATCATCATCGTCACACACCCCAGAGGCTAATTGATCTAAGCGGCCT
GCCCGGGCTAGTAGTATAGCAAGAGGTTTCAATATTGGGGCAATTTGTTTAGCAGTTGCCTCAAGATCCTCTG
GATGAACCTTTTCGCGTTCGACCGCAGCTCCCTGGTTAACTAAGCTCTCGAGGAGGAACACAAGATAAATCTT
GAAGACTTCACTGTTTCGGTCGAGGTTGGCTGCAAGGTAATTTCTTGCTTTGTGTATCTATGTATACGTTAGA
ATTCCCACTGACAAGGTCGAACAGCTAGATGAAAGGGGGGCTTGGACTACATACCGCCTCCAGCATGAGGTTA
TTCCCCTGAGCAACAGTATCTCGCAGTAGACGAGAATAGAACTTTAGCAAGAGACGGAACTCCGCAGGCTCAC
TCTTCAGCGAAAGAGCCGCAGTCTCCTGGATAATGTATGCATCAACTATGACATTCGCCTTGCCAATCTTCTG

CACCAGAATGGACTGTGCAAGGGCAACGATCTTGCTATCTTCACTATAAGTGGCGATTGTTACAATGGCATGT
ACCACATTTCTAGCTGTGACGGAAGTATCCCCCTCCCCATCAATAGCAAGGGATAAGAAGCTCTCATTCGTGG
GGGGAGCACGGTTCTCTATCGAATGAGAATAGCCAGTGCCTGACTCGGCAGTTAGAGCGGTGGCAGCCTTATC
AGCTGCCGATCCGGAGCTAAGGACGTTTCCGAGAGAATATAGGGTGCCTATGACGGTATCTTGGGAAAGAAGC
CGGAGGTTCTGGGCCAAACATTGGGCAGCCAGTGCGACGACAGGACTGCTTTGGCCATTTTGGACAATGAAGC
GAAGAAGAGAGCGGGTTAGGCCGTTTGCACTGCCTCGGGATAGCCTTGCTAGAATGACAGCAGCCTTAAGGAC
AACGGTAGCTAGGTCCTTGTTCATCATTTGGTCTGGATCTGAAATGGTATTGTCCAGGGATGTGAAGAGCAGG
TCTAGATCCACTGAATCCTGGTCAACCATGATGCAGTTGATCGAGCACATTATCGACAAAGCCTTGACCAAGA
ATGCAAGGCGTTGTTGCCAAGTGGAGCTGACTTGTAGGTAATCCGACTCTTCTTCGAGGAGATTGATCTGGTC
ACCCGCGATTTCTGCGGCGTATTTTACCAGGCTAATCATCCCGCGGTCTGAGGCTTTAGCTACATAACCACTG
TCTGCATATCGGGAGAGAAGGGTATCATCTGGCAGCGGTGGGGTTTGATCATTTTCAACAGGAGATGTGCAGG
ACTTTAGAAAGCCCATGAAAGCCTGTTGTAGCAACATGGAGCCAAGGGGTCGTCCGTCAGCCGCATACTGGCG
GGTGTACCGTCTCCAGTCGTGCAGCAGAGGATCTGGAGAGCGAGAGTTCCGTATGCCGGAAGAGATGGTCTCC
ACAGCTACCAGGAACCGCTCTGATAGAATGATGTGTAACTGCTTTACAATAGCAAGGCGATCCAGCGGTGCCC
AAAAATGGGGGAATTCGGACGAGGCCCGGAGAAATCCCACTAGCGATACCATTAGAGTAGTCACATCCGTAGA
TTCATGGCTCTCGACCTGTATCCCTGTCCCATCTGCATCAAAAACGGCCGGCAGGCGGTCTGTTGCCTGGTGA
CAATTCTCCAAGTACTCGTCAATGGCTTCGTGGACGTCATCCTGCAGCGACTTGTGCCTAGAGCCCAGTGAAA
GCAACGCCGTAACCACATCATGGGTCAAGGTCTCCCACGGCGAGGGTTTGTAAATATGTAGGAAAGGAGACGA
ATGGATGCGGAGCGTATGGAGCGTAAGCAGGTATCCGACCAGTAGAGAAACCAGCTTTGCAGCATGGTCACCA
CGCTGAACAGAGCCAGCTGCCTTGCACAGAGCAAGCAGTACGTCTAGTTCTCGAACCCCCTATCTTCCTCATT
AGCCACTTTCTCGCGCATATGCCATTGAAGGAGGGGTGTGTGTATGTATTGAGGCAGTAAAAGGGGGGGGGCA
GTACACTCGAGGCGCAGCGAGCCTGGGAGCCACTGGAAGCCGCCTTGGAGCCATTGCAGGCACTTATGAGCCT
CTCGACGTCTGAATTGCCCTGATCATCGTCCTGCCGGGGGTGTTTTGTGGCTAGTATCGCAAGTTTCTCGAGC
GCCGCACGCCGGATATACGATGGTGAATGATGCCTGAAGCTCTCCCCCGGCGGTTAGTTAGCAAATGTCTGCG
CCCAACGTAGCACCAACACAGGGCGAGAACACCTACATATCCGGTCAACCAGCCTCGCCGTCATCCAGGAGGC
CTTTCTTTTTCGTGGTTCCAAGGGGTGGGGATGCACCGAACGTGCAGTCGTGTGTTTTGGGGGCGTGGTCAGG
AGTGTATCAGAAGTGCTCTGCCTGCTGGAGATGCGAGTCGCTGGTAGGATCAACCCGCCAAGCCGAGGCCCGT
TTAGTGACGCCGGGGCAGAGAAACGACTGGGCAGCAAGACGGCAAAGCGAGAGAGATGCATGAAAGGGCATTT
TGATACACTCGATACGGACGCAGCCAGGGTTGCCAACTTGTTGAATTACTCTGGACAAGCCACTCTTCCTCTA
CAGGGTTTTTCTCTTGCGCCTCCGGGCCTGTCTCTGTCTCCCGGGGGGTTGAGCCCGCTACATGCAAGCCAGG
CATTGAAAAACAGTCTGCGCGAGAAAACCTCGATGATCGACTTTTGTGCCTTAGGCTATGATACGGAAATGCT
TTTACAAGATAGCTGGATATTTGCATCCAAGCAGGAGTTGAAATAACACAAAGTGACGAAGGCAAAAAATAAT
AAGAAACTGCGGGATGCGGCTCTAACGGGCGGCCAACCTTATATAAAGAGCGACTTCGCATCTTTCGACAGAA
AAAAAAGGTCGAGCAAGGGAAGTTCGCTCAGCCTGGCCTGCCATTGACTCTAAGCGCCGTCGTTCTCTTGCTT
GGGGGCTTGCTGCAGCTTGGCCTGGGCTTGTCCGGCCTGGAACTCAGGGAGCCGAAAGATAACTAGTTATTTA
TTTCGTCTCCGCACACCTCGGCGGCTGTCTCATAGTCGGGATATACATAGTACGGGGTAGCAGGTAGACTCAC
ACGCTGCCTCTCTCTGCTGAACTGCTTCTACCACCTTCGACAGCGACACTGTTCGGTCTTGCAACTACGGTAG
AGTGTGTTGGGGCTTGATCTTGCCTGCCATCGACTTGGAGTTGAAGTTGCGAAGCCATTGGCAGTCCATCCGG

GATATTCGTAACTGCCGTCAACTTCTTCACGCCGGGCCAAAAC**ATG**CCTCCGCAGAGGGAGAGAAGCAATCTG
AAGAGAGAAAGGGTAAGATATGAACACGTTATATCCAATTAGTTTCTCAGAAGGATGAAAATATTCAAGCTGC
TAACTTCTTTCCCAAACCTGCAGGGCAACGCAGCTCCAGACCATGAATCAAAGAAGCCACGGAGATCTCCTCG
AATCTCATCTCAATTGCAGCAGGATGCCGGATATCTACCTTCTCCCCTAGCTAATGAAGTAATCTCAGAAGAA
AAGCGTGAAGTTGCTACAGTTTCTCCCCCAGAAGGCCGCCGAAACCAGGCGCCTCGAGGTCAATCGCCAGACG
AGTCGTCATGTCCCACCCAGGGTTTCTCTCAGTATCCCCCAATCCAATCCATTGACGATGTGGAAGACGAGGT
GGCCCAGGGCATATGGGGATATCTTACTCCGCTAGATAACAATTTTGGAGGCCAATTGGTTCTGAAGAAGCGG
GACAGCACAAATACTACACCGGTAATTGGAGCAGCATCAGACATATCAACGCCGGAAAGCAACGCCTCAAAAC
GTGGAACTAGAGAGATAAACCGCGCTGGATATCTTATAGGCCGGCATGCAGAGTGCGGTATGCCTTTACCCAT
TGCATACTCTCTGCCGTCTAGTGATCAATTGCTAACCATCTGGGTACTGCAGATTTCAGGATCAAGCTCCCCA
CGATATCCAACCGCCATTGCCTGATATTCCACGAAAACAGGGGAGGAGACTTTGTTGCAATTGTGGAAGATCT
CTCTATCAATGGCACTTTCATCAACGACGCCATCATCGGCAAGAACAAACGCCGAGAGCTCGAAAACGGCGAC
GAGATTACCATCCTGCAAGAATCCAGATTCATCTTCAACTACCCGCGGAGTCGGAACACAAGCAAGTTCCGAC
AGCAGTATAAGCTCTTTGATATGCTGGGTAGGGGACACTTTGCCTCGGTTTATCTATGCGCTGAACGTTCTAC
GGGGATCAAGTATGCCGTCAAACATTTCGAGAAACGCCCGGGCGCAGTTCAGAGGCTAGACCGTGAAGCACTA
CAGCAAGAGATTTCAATGCTGATGAGTGTCAACCATCCGAACATGCTGTGCCTAAAGGATACCTTTGACGAGA
GCGATGGTGTCTTCCTGATTCTTGAGCTTGCGCCAGAGGGCGAACTGTTCAACTGGATCATTAGGCATCAGAA
ACTCAGCGAGGAGGAGACTCGAAAAGTATTCATTCAGCTTTTTGAAGGTTTGAGATACCTGGTATGTCGTTCG
TTTTCTGGTTGATCAGACTCATGGCAGTGTATATGGCTAACCGTTGTCGTAGCATGAACGAAACATCATTCAC
CGTGACATAAAGCCAGAGAACATTCTGGTTGTCGACAAAGATCTTACCGTAAAATTAGCAGACTTCGGCCTTG
CAAAGATTGTCGGCGAACACTCCTTTACCACCACGCTGTAAGTTCACCTACCTTCTGCCGTTCAAATCTGGGG
TCAATCTTACTAAGAGATCATAAAATCTAGATGTGGAACGCCTGGCTGTAAGTTTCCCTGCTGCTCTGTTAAC
GCTGGTGCTTTCATCCACCAAATGTGAATCTAACAATTCTTATCAGATGTTGCTCCTGAGATACTGGCAGAAA
ACGCTGAAGCCAGAATGTACACCCGAGCGGTTGACATCTGGTCACTCGGAGTGGTACTCTACATCTGCCTGTG
CGGATTCCCTCCTTTCTCAGATGAACTGTACCACCCAAGAGACTACCCATACACTCAGAAAGAGCAGATCCAG
CTCGGAATATTTAAGTATCCTCTTCCATACTGGGACTCAATCGATTATAGAGCCATGGACCTTATCGACTCCA
TGATCGAAGTCGACGTGAAGGAGCGTTTGAAAGTAGATGAGTGTCTCCAGCACCCATGGATCACTGGCATCGA
CCCCGATGAGCCTAGGGTGGCAGACAGCACCGATGACCTAGCCGGGGCCATCGGTGGCCTGAGGGTGTCCAAA
GAACGCCGGCCAACTATGGTCCGCAGGCTGTTGAGTGACATACATGAAGTCACGGTCAAGCAGGTGGTGAAGA
CCAAGGAAGACGACCCTGCTTCCCAGGACATAACTATATACCAGAATCATCCTGTGCCTCGAGCCCAGGAGCC
CAAACCGGCTGGCAACCGGAACTCAAAGGAGTTCATGGAACTCGGTGGAAGTGGAGACCAGGTGTTATTCGAT
GAGCAGTCTGACGACTCGCTGGAGCTTGAGAGTGAGGCACGGAAGCCAGTTGTCCACCAAGACGACGCAGACA
AGACTCCACGAGAAAGAAAGAGG**TAG**CAGAAGCCCGATCCGTCTTGGGATGGTATTCTGCTAGCTATACCGTG
CTATTCCGGACTTTTGTTTCCAATTCCTTTTTCGTTTATATTGATTGTGATCTTTCTTAATTTTTTCTTTAAC
TATACCCCCCGGTTGAAAGGTGATAGGGGGCCAACTAACCGAAAAGCAGCAGAAAAACCGGGCTTGGTCGCTG
CCTTGGAGACGACGGCGATTCTCGTGTTTAGGATTTCATTTTTGAGTGATATAACTATGGGTACAATAGCGTC
CACTGGGCAATGTAATTACAGTCTCTTCTCACTCACCACCGGACAATAATAACAATATTAATATTGACGGACC
GTTGATAGCTTAACCTGCCATGACTAAGCCGGCCTCCCTGAAGCCGCGATGGCCTGATGATGCGCCCACCGTC

AGGTCTCGCTCGCTCAGGCAGCAGAGAATCCAGCGGAGGAGATGACGCCCTGCACGAACACCGTCCAGGGACC

GTCTCCGCCCCGTCGCGAGCTCGAATTGTAATCATCTCGACGGCCGTTGGCCTGCTCGCCGCAAGGGAGAAAA

AGGGGGGCAGAAGGGCAGCGGTAATTTTGGTATTGGAGATGCAGCTGTAAACCAATAAAAAACGACGTAAAAT

ACCAGGAACAATGCATTATCTTACAGTACCGTACGCCCCTTGCATAAAATGCGAGTTCGATCTCAAGATGCAA

CTTTTTTTTTCCCTTGCTTCGTCCGCAGCAGCTTCAAGGCATTCATCACAAACAACGAACAAGTCTTGAGCGG

AAGCAGGAGGATAAGACGATCACC**ATG**GGCCTCTTAGCCGACATTGTCTCTCGTTTCTGCGAGAACTGCTCGA

CCCTGTCCACCGCCGCGCTCGTCGCAAGTGCCGTATCGGCTTTTATCGTCCTCTCCATTGTTATCAACGTCCT

GCAGCAGCTCCTGTTCAAGGACCCTACAAAGCCTCCGGTGGTCTTCCACTGGGTTCCGGTCATTGGAAGCACG

ATCTCCTATGGAATTGACCCGTACAAGTTCTTTGACGACTGCAAGGAGAAGGTTAGTTGGTGCAATTCAACTT

AGCTATATACCAGGTTGCGCAAAGCTAACGTATAAATAATTTCGGTACTCTAGTATGGAGACATCTTCACATT

CATACTGCTGGGCAAGAAGACGACTGTTTTTCTCGGTACAAAGGGAAATGATTTCATTTTGAACGGCAAGCTC

AAGGATGTTTGCGCGGAGGATGTCTACTCCCCCCTCACCACCCCAGTGTTCGGACGACATGTGGTGTATGATT

GCCCAAACTCCAAGCTCATGGAGCAGAAGAAGGTGTGTACAGCAATTTATTCCCGTCTACATCTCCTGGGCCT

CCATTGCTAATTTAATCGTCTACCAGTTCGTCAAGTTCGGCCTCACCTCTGAAGCTCTCCGATCCTATGTCAC

CCTGATCACCAAGGAAGTTGAGCAGTTCTTCGAGTCCTCCCCCGTCTTCAAGGGCGACTCCGGAGTTTTCAAC

GTCAGCAAGGTCATGGCTGAAATCACCATCTACACCGCCTCTCGATCTCTACAGGGCAAGGAGGTGCGAGGAA

AGTTCGATTCCAGCTTTGCGGAACTCTACTCCGATCTCGACATGGGCTTCGCCGCCATCAACTTCATGTTCCC

ATGGTTCCCCTTCCCACACAACCGCAAGCGTGACCGTGCTCAAAAGAAGATGGCCCAGGTTTACACCGACATC

ATCCGTCAGCGACGTGAGGCTGGTGGAGAGAAAGACTCCGAGGACATGGTATGGAACTTGATGTCGTCCGTGT

ACAAGAATGGAACGCCAATTCCAGATATCGAAGTCGCCCACATGATGATTGCTCTTCTTATGGCTGGCCAGCA

CTCTTCTTCCTCCACCGGCTCCTGGATCGTTCTCCGCCTTGCCAGCCGTCCAGATATTCTCGAGGAACTCTAC

GAGGAACAGAAACGTGTTCTCGGCGAGGATCTTCCACCACTCACCTACGAATCTCTCCAGAAACTTGATCTTC

ACAACAATGTAATCAAGGAGACTCTCCGCCTCCACGCTCCCATCCACTCTATCCTCCGTGCTGTTAAATCCCC

TATGCCCGTTGAAGGAACTAACTATGTTGTCCCAACCTCTCACAACCTCCTTGCCGCTCCTGGTGTTCCCTCA

CGAGACCCTCAGTACTTCCCTGACCCTCTTGTTTGGAACCCTCACCGATGGGAGAACAACGTCGGTGTCACCG

TAGTCGAGGCCAGTGAAGAAAAGACAGATTACGGATACGGTTTGGTTAGCAAGGGTGCCAACAGCCCTTACCT

CCCATTCGGCTCAGGCAGACACAGATGCATCGGTGAACAATTTGCATATGTTCAGCTTGGAACCGTAACAGCT

ACGTTAGCCAGACTAATGAAATGGAAGCAAGTTGAAGGCACCAAAGATGTTGTGCCGCCAACTGACTATTCGG

TAAGTTTGGCATATTACGCTCCTTTTTATTTTGCTCGATTTTACTAACTTTGCATTAATAGTCCCTCTTCTCG

AAGCCCCTTGGCGACCCAATGGTCTCGTGGGAGAAGCGAAAGCAGCCTTCCCAGAAA**TGA**TAATCACGACTGC

GGATTGAATCATGTTAAATTTCTTGCTTCTTGCTAATTTATTTCTTCTCTTTTCTTACGTTTATACACCGAGC

CATATTTGCACTTTATGGTCACGCAAATAGGTTTTCGGAATAGAGCATATGTAATAAAAACTTTCTTTTGACC

ATGCTTTTGAAGATGGGGGATGTAGATAATAGTATTATTCTAATACAGCCATTTATTACAATACTCTAGATGA

ACAGGAAAGTTGCAGTTACTCCGTTAGCTTTGGTGCAGAAACAAAATGGTAATATACATTGGAAATAGTACAT

CTTCCCGTAGCTGGGATAATCTAGCATCTAAACAGCGATATCCAAGCCAGTAATGTCAGGAAGTAACCAGCCA

GCAACGTCGTTAATCCCGTGCCCCCTTTCAACTCCGACGCTACAAAACGC**CTA**AGCAGCTGCCTGAGCATTCC

TCCTCTCCGCCAACGCTCTCTTCGCTCTGATCTCCTTCTGTCTTCTCTGACCACCGTCAATCTTTCTGCCACG

CTTATGCTCCCACATGCCATCAGTAACCTGAACCTTGACGGCGGTACTCCATGTGTTTGGAATAATCTGAGCA

TACGGCCCCTTGATCATATTCCCGTGGTGCAATTTATAATGGGCAAACTTTTTGAGTGGCTTGGAAGTATCCT
TGAGCTCTGTTTCGCCTGGTGGTAGATTGACAATCGCATAACGCATTCCAGGTGTAAGGGTAGCGGTGCCCAT
TGTGCTAACATCTAGGGACTCTCCGTCGGTGGCATTGGTAGTTTTTGCACTCTTTGACTTTTCGACGGGGACC
TCAACAGCTCTCAGCTCAGCAACACCGTCAACAACGTTTTCGAAATCACCTCCTGGGCCGTAGTCCTGCTGTC
TGAATTTCTCTCTCCACCGCAGTATGTATCGTCTGTCCCTTGTCATTTCTATGCCCAATTCTCGAAGCTCAGC
CGAGTCCGCTGTAAACAATTGCTTCCATCCGGTGATTTTGCTGGAATATCTGGACATGTTTCGCCCAATTAGG
CGTAGGAATGTTTCGACATCGGGCACGAATGGGGTAGGTGATGGCACTGTTGCGGGTTTTCGAGATTGATGAA
GGTACCGTACGCATGGCTGATAATGGGGGTTTGTGACGGAAGTGAAGAGCCGTAGTGGAAGGGATGTTCTGGC
ATTAAAGCCCGGTATGCGCGCCGCAGC**CAT**AGTGGTTATATCGCCTTATATCAAACCGTTCAATGTCGAGATC
TGGGGGGTGAAGATTTTAATTTAGCTCGTGGTGTAAGAACACCATCGGCGGGCGTGACGAAAGCGAAAAAAGC
CACGGCACCAAGGGCCCAGGTAAACCGGTAAGGGACTCAAGGCGAGTCAATCCAATGCTCAATGGCGCTGTAT
AGAAGATGTTCAACTGGTTCTTCGACTTCGTTCAAGATTTTGCACCAAAATTTTGAATTAGGTCCGACGGCCT
TAGCTCGGATCAACAAAACGCCGATCTCCGGTGAGCTCGGGCCAGCCGAAGCTTCCATCGCAACATTCAAAGA
TGAAAGACGACGATATATATTTTGATAACAAACAAAGCTGTATTGTTGATTGAATACTAGGAGAGCTGCTCAG
AGCAGTGTCTCACTCTTGTGCCACTCTATCCTTTGTCTCGCGAAATTGGTGGAAGCCAATTTCGCTTGACATT
ATGGCAGTGTCGCCGCTTCCCTCTCAATTCCCTTCCCATCACTACTACCGGTGCTCCAAAGTCGTTAGCGATG
AGAAATATTGCTTCTCAAGAGCCTCGGACGGCGCCTAGTGGTGATCCTGCAGTTCCTAAGCCCTCCATGCCCG
AACCAGAGCTGGCGGAGCAGTTAAACGCTGATGTACGGCGGAAATATGTCAAAGGTGGGCGGGCAAATACACC
CCAACCATTGGGACCCCATAATGCTAACATTTGGTTATTCTGAAAGATAAGAAGCTCGGTGAAGGTACATATG
CAGTTGTTTACCTAGGCCATCTACGAGATGATCCCACTTCGCTAATAAAGAGCGACTTCGCATCTTTCGACAG
AAAAAAAAGGTCGAGCAAGGGAAGTTCGCTCAGCCTGGCCTGCCATTGACTCTAAGCGCCGTCGTTCTCTTGC
TTGGGGGCTTGCTGCAGCTTGGCCTGGGCTTGTCCGGCCTGGAACTCAGGGAGCCGAAAGATAACTAGTTATT
TATTTCGTCTCCGCACACCTCGGCGGCTGTCTCATAGTCGGGATATACATAGTACGGGGTAGCAGGTAGACTC
ACACGCTGCCTCTCTCTGCTGAACTGCTTCTACCACCTTCGACAGCGACACTGTTCGGTCTTGCAACTACGGT
AGAGTGTGTTGGGGCTTGATCTTGCCTGCCATCGACTTGGAGTTGAAGTTGCGAAGCCATTGGCAGTCCATCC
GGGATATTCGTAACTGCCGTCAACTTCTTCACGCCGGGCCAAAAC**ATG**CCTCCGCAGAGGGAGAGAAGCAATC
TGAAGAGAGAAAGGGTAAGATATGAACACGTTATATCCAATTAGTTTCTCAGAAGGATGAAAATATTCAAGCT
GCTAACTTCTTTTCCCAAACCTGCAGGGCAACGCAGCTCCAGACCATGAATCAAAGAAGCCACGGAGATCTCC
TCGAATCTCATCTCAATTGCAGCAGGATGCCGGATATCTACCTTCTCCCCTAGCTAATGAAGTAATCTCAGAA
GAAAAGCGTGAAGTTGCTACAGTTTCTCCCCCAGAAGGCCGCCGAAACCAGGCGCCTCGAGGTCAATCGCCAG
ACGAGTCGTCATGTCCCACCCAGGGTTTCTCTCAGTATCCCCCAATCCAATCCATTGACGATGTGGAAGACGA
GGTGGCCCAGGGCATATGGGGATATCTTACTCCGCTAGATAACAATTTTGGAGGCCAATTGGTTCTGAAGAAG
CGGGACAGCACAAATACTACACCGGTAATTGGAGCAGCATCAGACATATCAACGCCGGAAAGCAACGCCTCAA
AACGTGGAACTAGAGAGATAAACCGCGCTGGATATCTTATAGGCCGGCATGCAGAGTGCGGTATGCCTTTACC
CATTGCATACTCTCTGCCGTCTAGTGATCAATTGCTAACCATCTGGGTACTGCAGATTTCAGGATCAAGCTCC
CCACGATATCCAACCGCCATTGCCTGATATTCCACGAAAACAGGGGAGGAGACTTTGTTGCAATTGTGGAAGA
TCTCTCTATCAATGGCACTTTCATCAACGACGCCATCATCGGCAAGAACAAACGCCGAGAGCTCGAAAACGGC
GACGAGATTACCATCCTGCAAGAATCCAGATTCATCTTCAACTACCCGCGGAGTCGGAACACAAGCAAGTTCC

GACAGCAGTATAAGCTCTTTGATATGCTGGGTAGGGGACACTTTGCCTCGGTTTATCTATGCGCTGAACGTTC
TACGGGGATCAAGTATGCCGTCAAACATTTCGAGAAACGCCCGGGCGCAGTTCAGAGGCTAGACCGTGAAGCA
CTACAGCAAGAGATTTCAATGCTGATGAGTGTCAACCATCCGAACATGCTGTGCCTAAAGGATACCTTTGACG
AGAGCGATGGTGTCTTCCTGATTCTTGAGCTTGCGCCAGAGGGCGAACTGTTCAACTGGATCATTAGGCATCA
GAAACTCAGCGAGGAGGAGACTCGAAAAGTATTCATTCAGCTTTTTGAAGGTTTGAGATACCTGGTATGTCGT
TCGTTTTCTGGTTGATCAGACTCATGGCAGTGTATATGGCTAACCGTTGTCGTAGCATGAACGAAACATCATT
CACCGTGACATAAAGCCAGAGAACATTCTGGTTGTCGACAAAGATCTTACCGTAAAATTAGCAGACTTCGGCC
TTGCAAAGATTGTCGGCGAACACTCCTTTACCACCACGCTGTAAGTTCACCTACCTTCTGCCGTTCAAATCTG
GGGTCAATCTTACTAAGAGATCATAAAATCTAGATGTGGAACGCCTGGCTGTAAGTTTCCCTGCTGCTCTGTT
AACGCTGGTGCTTTCATCCACCAAATGTGAATCTAACAATTCTTATCAGATGTTGCTCCTGAGATACTGGCAG
AAAACGCTGAAGCCAGAATGTACACCCGAGCGGTTGACATCTGGTCACTCGGAGTGGTACTCTACATCTGCCT
GTGCGGATTCCCTCCTTTCTCAGATGAACTGTACCACCCAAGAGACTACCCATACACTCAGAAAGAGCAGATC
CAGCTCGGAATATTTAAGTATCCTCTTCCATACTGGGACTCAATCGATTATAGAGCCATGGACCTTATCGACT
CCATGATCGAAGTCGACGTGAAGGAGCGTTTGAAAGTAGATGAGTGTCTCCAGCACCCATGGATCACTGGCAT
CGACCCCGATGAGCCTAGGGTGGCAGACAGCACCGATGACCTAGCCGGGGCCATCGGTGGCCTGAGGGTGTCC
AAAGAACGCCGGCCAACTATGGTCCGCAGGCTGTTGAGTGACATACATGAAGTCACGGTCAAGCAGGTGGTGA
AGACCAAGGAAGACGACCCTGCTTCCCAGGACATAACTATATACCAGAATCATCCTGTGCCTCGAGCCCAGGA
GCCCAAACCGGCTGGCAACCGGAACTCAAAGGAGTTCATGGAACTCGGTGGAAGTGGAGACCAGGTGTTATTC
GATGAGCAGTCTGACGACTCGCTGGAGCTTGAGAGTGAGGCACGGAAGCCAGTTGTCCACCAAGACGACGCAG
ACAAGACTCCACGAGAAAGAAAGAGG**TAG**CAGAAGCCCGATCCGTCTTGGGATGGTATTCTGCTAGCTATACC
GTGCTATTCCGGACTTTTGTTTCCAATTCCTTTTTCGTTTATATTGATTGTGATCTTTCTTAATTTTTTCTTT
AACTATACCCCCCGGTTGAAAGGTGATAGGGGGCCAACTAACCGAAAAGCAGCAGAAAAACCGGCTTGGTCGC
TGCCTTGGAGACGACGGCGATTCTCGTGTTTAGGATTTCATTTTTGAGTGATATAACTATGGGTACAATAGCG
TCCACTGGGCAATGTAATTACAGTCTCTTCTCACTCACCACCGGACAATAATAACAATATTAATATTGACGGA
CCGTTGATAGCTTAACCTGCCATGACTAAGCCGGCCTCCCTGAAGCCGCGATGGCCTGATGATGCGCCCACCG
TCAGGTCTCGCTCGCTCAGGCAGCAGAGAATCCAGCGGAGGAGATGACGCCCTGCACGAACACCGTCCAGGGA
CCGTCTCCGCCCCGTCGCGAGCTCGAATTGTAATCATCTCGACGGCCGTTGGCCTGCTCGCCGCAAGGGAGAA
AAAGGGGGGCAGAAGGGCAGCGGTAATTTTGGTATTGGAGATGCAGCTGTAAACCAATAAAAAACGACGTAAA
ATACCAGGAACAATGCATTATCTTACAGTACCGTACGCCCCTTGCATAAAATGCGAGTTCGATCTCAAGATGC
AACTTTTTTTTTCCCTTGCTTCGTCCGCAGCAGCTTCAAGGCATTCATCACAAACAACGAACAAGTCTTGAGC
GGAAGCAGGAGGATAAGACGATCACC**ATG**GGCCTCTTAGCCGACATTGTCTCTCGTTTCTGCGAGAACTGCTC
GACCCTGTCCACCGCCGCGCTCGTCGCAAGTGCCGTATCGGCTTTTATCGTCCTCTCCATTGTTATCAACGTC
CTGCAGCAGCTCCTGTTCAAGGACCCTACAAAGCCTCCGGTGGTCTTCCACTGGGTTCCGGTCATTGGAAGCA
CGATCTCCTATGGAATTGACCCGTACAAGTTCTTTGACGACTGCAAGGAGAAGGTTAGTTGGTGCAATTCAAC
TTAGCTATATACCAGGTTGCGCAAAGCTAACGTATAAATAATTTCGGTACTCTAGTATGGAGACATCTTCACA
TTCATACTGCTGGGCAAGAAGACGACTGTTTTTCTCGGTACAAAGGGAAATGATTTCATTTTGAACGGCAAGC
TCAAGGATGTTTGCGCGGAGGATGTCTACTCCCCCCTCACCACCCCAGTGTTCGGACGACATGTGGTGTATGA
TTGCCCAAACTCCAAGCTCATGGAGCAGAAGAAGGTGTGTACAGCAATTTATTCCCGTCTACATCTCCTGGGC

CTCCATTGCTAATTTAATCGTCTACCAGTTCGTCAAGTTCGGCCTCACCTCTGAAGCTCTCCGATCCTATGTC

ACCCTGATCACCAAGGAAGTTGAGCAGTTCTTCGAGTCCTCCCCCGTCTTCAAGGGCGACTCCGGAGTTTTCA

ACGTCAGCAAGGTCATGGCTGAAATCACCATCTACACCGCCTCTCGATCTCTACAGGGCAAGGAGGTGCGAGG

AAAGTTCGATTCCAGCTTTGCGGAACTCTACTCCGATCTCGACATGGGCTTCGCCGCCATCAACTTCATGTTC

CCATGGTTCCCCTTCCCACACAACCGCAAGCGTGACCGTGCTCAAAAGAAGATGGCCCAGGTTTACACCGACA

TCATCCGTCAGCGACGTGAGGCTGGTGGAGAGAAAGACTCCGAGGACATGGTATGGAACTTGATGTCGTCCGT

GTACAAGAATGGAACGCCAATTCCAGATATCGAAGTCGCCCACATGATGATTGCTCTTCTTATGGCTGGCCAG

CACTCTTCTTCCTCCACCGGCTCCTGGATCGTTCTCCGCCTTGCCAGCCGTCCAGATATTCTCGAGGAACTCT

ACGAGGAACAGAAACGTGTTCTCGGCGAGGATCTTCCACCACTCACCTACGAATCTCTCCAGAAACTTGATCT

TCACAACAATGTAATCAAGGAGACTCTCCGCCTCCACGCTCCCATCCACTCTATCCTCCGTGCTGTTAAATCC

CCTATGCCCGTTGAAGGAACTAACTATGTTGTCCCAACCTCTCACAACCTCCTTGCCGCTCCTGGTGTTCCCT

CACGAGACCCTCAGTACTTCCCTGACCCTCTTGTTTGGAACCCTCACCGATGGGAGAACAACGTCGGTGTCAC

CGTAGTCGAGGCCAGTGAAGAAAAGACAGATTACGGATACGGTTTGGTTAGCAAGGGTGCCAACAGCCCTTAC

CTCCCATTCGGCTCAGGCAGACACAGATGCATCGGTGAACAATTTGCATATGTTCAGCTTGGAACCGTAACAG

CTACGTTAGCCAGACTAATGAAATGGAAGCAAGTTGAAGGCACCAAAGATGTTGTGCCGCCAACTGACTATTC

GGTAAGTTTGGCATATTACGCTCCTTTTTATTTTGCTCGATTTTACTAACTTTGCATTAATAGTCCCTCTTCT

CGAAGCCCCTTGGCGACCCAATGGTCTCGTGGGAGAAGCGAAAGCAGCCTTCCCAGAAA**TGA**TAATCACGACT

GCGGATTGAATCATGTTAAATTTCTTGCTTCTTGCTAATTTATTTCTTCTCTTTTCTTACGTTTATACACCGA

GCCATATTTGCACTTTATGGTCACGCAAATAGGTTTTCGGAATAGAGCATATGTAATAAAAACTTTCTTTTGA

CCATGCTTTTGAAGATGGGGGATGTAGATAATAGTATTATTCTAATACAGCCATTTATTACAATACTCTAGAT

GAACAGGAAAGTTGCAGTTACTCCGTTAGCTTTGGTGCAGAAACAAAATGGTAATATACATTGGAAATAGTAC

ATCTTCCCGTAGCTGGGATAATCTAGCATCTAAACAGCGATATCCAAGCCAGTAATGTCAGGAAGTAACCAGC

CAGCAACGTCGTTAATCCCGTGCCCCCTTTCAACTCCGACGCTACAAAACGC**CTA**AGCAGCTGCCTGAGCATT

CCTCCTCTCCGCCAACGCTCTCTTCGCTCTGATCTCCTTCTGTCTTCTCTGACCACCGTCAATCTTTCTGCCA

CGCTTATGCTCCCACATGCCATCAGTAACCTGAACCTTGACGGCGGTACTCCATGTGTTTGGAATAATCTGAG

CATACGGCCCCTTGATCATATTCCCGTGGTGCAATTTATAATGGGCAAACTTTTTGAGTGGCTTGGAAGTATC

CTTGAGCTCTGTTTCGCCTGGTGGTAGATTGACAATCGCATAACGCATTCCAGGTGTAAGGGTAGCGGTGCCC

ATTGTGCTAACATCTAGGGACTCTCCGTCGGTGGCATTGGTAGTTTTTGCACTCTTTGACTTTTCGACGGGGA

CCTCAACAGCTCTCAGCTCAGCAACACCGTCAACAACGTTTTCGAAATCACCTCCTGGGCCGTAGTCCTGCTG

TCTGAATTTCTCTCTCCACCGCAGTATGTATCGTCTGTCCCTTGTCATTTCTATGCCCAATTCTCGAAGCTCA

GCCGAGTCCGCTGTAAACAATTGCTTCCATCCGGTGATTTTGCTGGAATATCTGGACATGTTTCGCCCAATTA

GGCGTAGGAATGTTTCGACATCGGGCACGAATGGGGTAGGTGATGGCACTGTTGCGGGTTTTCGAGATTGATG

AAGGTACCGTACGCATGGCTGATAATGGGGGTTTGTGACGGAAGTGAAGAGCCGTAGTGGAAGGGATGTTCTG

GCATTAAAGCCCGGTATGCGCGCCGCAGC**CAT**AGTGGTTATATCGCCTTATATCAAACCGTTCAATGTCGAGA

TCTGGGGGGTGAAGATTTTAATTTAGCTCGTGGTGTAAGAACACCATCGGCGGGCGTGACGAAAGCGAAAAAA

GCCACGGCACCAAGGGCCCAGGTAAACCGGTAAGGGACTCAAGGCGAGTCAATCCAATGCTCAATGGCGCTGT

ATAGAAGATGTTCAACTGGTTCTTCGACTTCGTTCAAGATTTTGCACCAAAATTTTGAATTAGGTCCGACGGC

CTTAGCTCGGATCAACAAAACGCCGATCTCCGGTGAGCTCGGGCCAGCCGAAGCTTCCATCGCAACATTCAAA

GATGAAAGACGACGATATATATTTTGATAACAAACAAAGCTGTATTGTTGATTGAATACTAGGAGAGCTGCTC
AGAGCAGTGTCTCACTCTTGTGCCACTCTATCCTTTGTCTCGCGAAATTGGTGGAAGCCAATTTCGCTTGACA
TTATGGCAGTGTCGCCGCTTCCCTCTCAATTCCCTCCCATCACTACTACCGGTGCTCCAAAGTCGTTAGCGAT
GAGAAATATTGCTTCTCAAGAGCCTCGGACGGCGCCTAGTGGTGATCCTGCAGTTCCTAAGCCCTCCATGCCC
GAACCAGAGCTGGCGGAGCAGTTAAACGCTGATGTACGGCGGAAATATGTCAAAGGTGGGCGGGCAAATACAC
CCCAACCATTGGGACCCCATAATGCTAACATTTGGTTATTCTGAAAGATAAGAAGCTCGGTGAAGGTACATAT
GCAGTTGTTTACCTAGGCCATCTACGAGATGATCCCACTTCGCTAATAAAGAGCGACTTCGCATCTTTCGACA
GAAAAAAAAGGTCGAGCAAGGGAAGTTCGCTCAGCCTGGCCTGCCATTGACTCTAAGCGCCGTCGTTCTCTTG
CTTGGGGGCTTGCTGCAGCTTGGCCTGGGCTTGTCCGGCCTGGAACTCAGGGAGCCGAAAGATAACTAGTTAT
TTATTTCGTCTCCGCACACCTCGGCGGCTGTCTCATAGTCGGGATATACATAGTACGGGGTAGCAGGTAGACT
CACACGCTGCCTCTCTCTGCTGAACTGCTTCTACCACCTTCGACAGCGACACTGTTCGGTCTTGCAACTACGG
TAGAGTGTGTTGGGGCTTGATCTTGCCTGCCATCGACTTGGAGTTGAAGTTGCGAAGCCATTGGCAGTCCATC
CGGGATATTCGTAACTGCCGTCAACTTCTTCACGCCGGGCCAAAAC**ATG**CCTCCGCAGAGGGAGAGAAGCAAT
CTGAAGAGAGAAAGGGTAAGATATGAACACGTTATATCCAATTAGTTTCTCAGAAGGATGAAAATATTCAAGC
TGCTAACTTCTTTTCCCAAACCTGCAGGGCAACGCAGCTCCAGACCATGAATCAAAGAAGCCACGGAGATCTC
CTCGAATCTCATCTCAATTGCAGCAGGATGCCGGATATCTACCTTCTCCCCTAGCTAATGAAGTAATCTCAGA
AGAAAAGCGTGAAGTTGCTACAGTTTCTCCCCCAGAAGGCCGCCGAAACCAGGCGCCTCGAGGTCAATCGCCA
GACGAGTCGTCATGTCCCACCCAGGGTTTCTCTCAGTATCCCCCAATCCAATCCATTGACGATGTGGAAGACG
AGGTGGCCCAGGGCATATGGGGATATCTTACTCCGCTAGATAACAATTTTGGAGGCCAATTGGTTCTGAAGAA
GCGGGACAGCACAAATACTACACCGGTAATTGGAGCAGCATCAGACATATCAACGCCGGAAAGCAACGCCTCA
AAACGTGGAACTAGAGAGATAAACCGCGCTGGATATCTTATAGGCCGGCATGCAGAGTGCGGTATGCCTTTAC
CCATTGCATACTCTCTGCCGTCTAGTGATCAA

[Read No.m64281e_230311_112734/165807379/ccs] [Antisense (-) strand]

GGT~ATT (5'UTR)

CTA~CAT (*chkB* protein kinase genomic DNA) (Reverse sequence)

TCA~CAT (*TinCYP51B* gene) (Reverse sequence)

AGC~TTA (3'UTR)

ATG~TAG (*FYV4* genomic DNA) (Forward sequence)

CACATTTGGTGGATGAAAGCACCAGCGTTAACAGAGCAGCAGGGAAACTTACAGCCAGGCGTTCCACATCTAG
ATTTTATGATCTCTTAGTAAGATTGACCCCAGATTTGAACGGCAGAAGGTAGGTGAACTTACAGCGTGGTGGT
AAAGGAGTGTTCGCCGACAATCTTTGCAAGGCCGAAGTCTGCTAATTTTACGGTAAGATCTTTGTCGACAACC
AGAATGTTCTCTGGCTTTATGTCACGGTGAATGATGTTTCGTTCATGCTACGACAACGGTTAGCCATATACAC
TGCCATGAGTCTGATCAACCAGAAAACGAACGACATACCAGGTATCTCAAACCTTCAAAAAGCTGAATGAATA
CTTTTCGAGTCTCCTCCTCGCTGAGTTTCTGATGCCTAATGATCCAGTTGAACAGTTCGCCCTCTGGCGCAAG
CTCAAGAATCAGGAAGACACCATCGCTCTCGTCAAAGGTATCCTTTAGGCACAGCATGTTCGGATGGTTGACA

CTCATCAGCATTGAAATCTCTTGCTGTAGTGCTTCACGGTCTAGCCTCTGAACTGCGCCCGGGCGTTTCTCGA
AATGTTTGACGGCATACTTGATCCCCGTAGAACGTTCAGCGCATAGATAAACCGAGGCAAAGTGTCCCCTACC
CAGCATATCAAAGAGCTTATACTGCTGTCGGAACTTGCTTGTGTTCCGACTCCGCGGGTAGTTGAAGATGAAT
CTGGATTCTTGCAGGATGGTAATCTCGTCGCCGTTTTCGAGCTCTCGGCGTTTGTTCTTGCCGATGATGGCGT
CGTTGATGAAAGTGCCATTGATAGAGAGATCTTCCACAATTGCAACAAAGTCTCCTCCCCTGTTTTCGTGGAA
TATCAGGCAATGGCGGTTGGATATCGTGGGGAGCTTGATCCTGAAATCTGCAGTACCCAGATGGTTAGCAATT
GATCACTAGACGGCAGAGAGTATGCAATGGGTAAAGGCATACCGCACTCTGCATGCCGGCCTATAAGATATCC
AGCGCGGTTTATCTCTCTAGTTCCACGTTTTGAGGCGTTGCTTTCCGGCGTTGATATGTCTGATGCTGCTCCA
ATTACCGGTGTAGTATTTGTGCTGTCCCGCTTCTTCAGAACCAATTGGCCTCCAAAATTGTTATCTAGCGGAG
TAAGATATCCCCATATGCCCTGGGCCACCTCGTCTTCCACATCGTCAATGGATTGGATTGGGGATACTGAGA
GAAACCCTGGGTGGGACATGACGACTCGTCTGGCGATTGACCTCGAGGCGCCTGGTTTCGGCGGCCTTCTGGG
GGAGAAACTGTAGCAACTTCACGCTTTTCTTCTGAGATTACTTCATTAGCTAGGGGAGAAGGTAGATATCCGG
CATCCTGCTGCAATTGAGATGAGATTCGAGGAGATCTCCGTGGCTTCTTTGATTCATGGTCTGGAGCTGCGTT
GCCCTGCAGGTTTGGGAAAAGAAGTTAGCAGCTTGAATATTTTCATCCTTCTGAGAAACTAATTGGATATAAC
GTGTTCATATCTTACCCTTTCTCTCTTCAGATTGCTTCTCTCCCTCTGCGGAGG**CAT**GTTTTGGCCCGGCGTG
AAGAAGTTGACGGCAGTTACGAATATCCCGGATGGACTGCCAATGGCTTCGCAACTTCAACTCCAAGTCGATG
GCAGGCAAGATCAAGCCCCAACACACTCTACCGTAGTTGCAAGACCGAACAGTGTCGCTGTCGAAGGTGGTAG
AAGCAGTTCAGCAGAGAGAGGCAGCGTGTGAGTCTACCTGCTACCCCGTACTATGTATATCCCGACTATGAGA
CAGCCGCCGAGGTGTGCGGAGACGAAATAAATAACTAGTTATCTTTCGGCTCCCTGAGTTCCAGGCCGGACAA
GCCCAGGCCAAGCTGCAGCAAGCCCCCAAGCAAGAGAACGACGGCGCTTAGAGTCAATGGCAGGCCAGGCTGA
GCGAACTTCCCTTGCTCGACCTTTTTTTTCTGTCGAAAGATGCGAAGTCGCTCTTTATTAGCGAAGTGGGATC
ATCTCGTAGATGGCCTAGGTAAACAACTGCATATGTACCTTCACCGAGCTTCTTATCTTTCAGAATAACCAAA
TGTTAGCATTATGGGGTCCCAATGGTTGGGGTGTATTTGCCCGCCCACCTTTGACATATTTCCGCCGTACATC
AGCGTTTAACTGCTCCGCCAGCTCTGGTTCGGGCATGGAGGGCTTAGGAACTGCAGGATCACCACTAGGCGCC
GTCCGAGGCTCTTGAGAAGCAATATTTCTCATCGCTAACGACTTTGGAGCACCGGTAGTAGTGATGGGAGGGA
ATTGAGAGGGAAGCGGCGACACTGCCATAATGTCAAGCGAAATTGGCTTCCACCAATTTCGCGAGACAAAGGA
TAGAGTGGCACAAGAGTGAGACACTGCTCTGAGCAGCTCTCCTAGTATTCAATCAACAATACAGCTTTGTTTG
TTATCAAAATATATATCGTCGTCTTTCATCTTTGAATGTTGCGATGGAAGCTTCGGCTGGCCCGAGCTCACCG
GAGATCGGCGTTTTGTTGATCCGAGCTAAGGCCGTCGGACCTAATTCAAAATTTTGGTGCAAAATCTTGAACG
AAGTCGAAGAACCAGTTGAACATCTTCTATACAGCGCCATTGAGCATTGGATTGACTCGCCTTGAGTCCCTTA
CCGGTTTACCTGGGCCCTTGGTGCCGTGGCTTTTTTCGCTTTCGTCACGCCCGCCGATGGTGTTCTTACACCA
CGAGCTAAATTAAAATCTTCACCCCCCAGATCTCGACATTGAACGGTTTGATATAAGGCGATATAACCACT**AT**
GGCTGCGGCGCGCATACCGGGCTTTAATGCCAGAACATCCCTTCCACTACGGCTCTTCACTTCCGTCACAACC
CCCATTATCAGCCATGCGTACGGTACCTTCATCAATCTCGAAAACCCGCAACAGTGCCATCACCTACCCCATT
CGTGCCCGATGTCGAAACATTCCTACGCCTAATTGGGCGAAACATGTCCAGATATTCCAGCAAAATCACCGGA
TGGAAGCAATTGTTTACAGCGGACTCGGCTGAGCTTCGAGAATTGGGCATAGAAATGACAAGGGACAGACGAT
ACATACTGCGGTGGAGAGAGAAATTCAGACAGCAGGACTACGGCCCAGGAGGTGATTTCGAAAACGTTGTTGA
CGGTGTTGCTGAGCTGAGAGCTGTTGAGGTCCCCGTCGAAAAGTCAAAGAGTGCAAAAACTACCAATGCCACC

GACGGAGAGTCCCTAGATGTTAGCACAATGGGCACCGCTACCCTTACACCTGGAATGCGTTATGCGATTGTCA
ATCTACCACCAGGCGAAACAGAGCTCAAGGATACTTCCAAGCCACTCAAAAAGTTTGCCCATTATAAATTGCA
CCACGGGAATATGATCAAGGGGCCGTATGCTCAGATTATTCCAAACACATGGAGTACCGCCGTCAAGGTTCAG
GTTACTGATGGCATGTGGGAGCATAAGCGTGGCAGAAAGATTGACGGTGGTCAGAGAAGACAGAAGGAGATCA
GAGCGAAGAGAGCGTTGGCGGAGAGGAGGAATGCTCAGGCAGCTGCT**TAG**GCGTTTTGTAGCGTCGGAGTTGA
AAGGGGCACGGGATTAACGACGTTGCTGGCTGGTTACTTCCTGACATTACTGGCTTGGATATCGCTGTTTAGA
TGCTAGATTATCCCAGCTACGGGAAGATGTACTATTTCCAATGTATATTACCATTTTGTTTCTGCACCAAAGC
TAACGGAGTAACTGCAACTTTCCTGTTCATCTAGAGTATTGTAATAAATGGCTGTATTAGAATAATACTATTA
TCTACATCCCCCATCTTCAAAAGCATGGTCAAAAGAAAGTTTTTATTACATATGCTCTATTCCGAAAACCTAT
TTGCGTGACCATAAAGTGCAAATATGGCTCGGTGTATAAACGTAAGAAAAGAGAAGAAATAAATTAGCAAGAA
GCAAGAAATTTAACATGATTCAATCCGCAGTCGTGATTA**TCA**TTTCTGGGAAGGCTGCTTTCGCTTCTCCCAC
GAGACCATTGGGTCGCCAAGGGGCTTCGAGAAGAGGGACTATTAATGCAAAGTTAGTAAAATCGAGCAAAATA
AAAAGGAGCGTAATATGCCAAACTTACCGAATAGTCAGTTGGCGGCACAACATCTTTGGTGCCTTCAACTTGC
TTCCATTTCATTAGTCTGGCTAACGTAGCTGTTACGGTTCCAAGCTGAACATATGCAAATTGTTCACCGATGC
ATCTGTGTCTGCCTGAGCCGAATGGGAGGTAAGGGCTGTTGGCACCCTTGCTAACCAAACCGTATCCGTAATC
TGTCTTTTCTTCACTGGCCTCGACTACGGTGACACCGACGTTGTTCTCCCATCGGTGAGGGTTCCAAACAAGA
GGGTCAGGGAAGTACTGAGGGTCTCGTGAGGGAACACCAGGAGCGGCAAGGAGGTTGTGAGAGGTTGGGACAA
CATAGTTAGTTCCTTCAACGGGCATAGGGATTTAACAGCACGGAGGATAGAGTGGATGGGAGCGTGGAGGCGG
AGAGTCTCCTTGATTACATTGTTGTGAAGATCAAGTTTCTGGAGAGATTCGTAGGTGAGTGGTGGAAGATCCT
CGCCGAGAACACGTTTCTGTTCCTCGTAGAGTTCCTCGAGAATATCTGGACGGCTGGCAAGGCGGAGAACGAT
CCAGGAGCCGGTGGAGGAAGAAGAGTGCTGGCCAGCCATAAGAAGAGCAATCATCATGTGGGCGACTTCGATA
TCTGGAATTGGCGTTCCATTCTTGTACACGGACGACATCAAGTTCCATACCATGTCCTCGGAGTCTTTCTCTC
CACCAGCCTCACGTCGCTGACGGATGATGTCGGTGTAAACCTGGGCCATCTTCTTTTGAGCACGGTCACGCTT
GCGGTTGTGTGGGAAGGGGAACCATGGGAACATGAAGTTGATGGCGGCGAAGCCCATGTCGAGATCGGAGTAG
AGTTCCGCAAAGCTGGAATCGAACTTTCCTCGCACCTCCTTGCCCTGTAGAGATCGAGAGGCGGTGTAGATGG
TGATTTCAGCCATGACCTTGCTGACGTTGAAAACTCCGGAGTCGCCCTTGAAGACGGGGGAGGACTCGAAGAA
CTGCTCAACTTCCTTGGTGATCAGGGTGACATAGGATCGGAGAGCTTCAGAGGTGAGGCCGAACTTGACGAAC
TGGTAGACGATTAAATTAGCAATGGAGGCCCAGGAGATGTAGACGGGAATAAATTGCTGTACACACCTTCTTC
TGCTCCATGAGCTTGGAGTTTGGGCAATCATACACCACATGTCGTCCGAACACTGGGGTGGTGAGGGGGGAGT
AGACATCCTCCGCGCAAACATCCTTGAGCTTGCCGTTCAAAATGAAATCATTTCCCTTTGTACCGAGAAAAAC
AGTCGTCTTCTTGCCCAGCAGTATGAATGTGAAGATGTCTCCATACTAGAGTACCGAAATTATTTATACGTTA
GCTTTGCGCAACCTGGTATATAGCTAAGTTGAATTGCACCAACTAACCTTCTCCTTGCAGTCGTCAAAGAACT
TGTACGGGTCAATTCCATAGGAGATCGTGCTTCCAATGACCGGAACCCAGTGGAAGACCACCGGAGGCTTTGT
AGGGTCCTTGAACAGGAGCTGCTGCAGGACGTTGATAACAATGGAGAGGACGATAAAAGCCGATACGGCACTT
GCGACGAGCGCGGCGGTGGACAGGGTCGAGCAGTTCTCGCAGAAACGAGAGACAATGTCGGCTAAGAGGCC**CA**
TGGTGATCGTCTTATCCTCCTGCTTCCGCTCAAGACTTGTTCGTTGTTTGTGATGAATGCCTTGAAGCTGCTG
CGGACGAAGCAAGGGAAAAAAAAAGTTGCATCTTGAGATCGAACTCGCATTTTATGCAAGGGGCGTACGGTAC
TGTAAGATAATGCATTGTTCCTGGTATTTTACGTCGTTTTTTTATTGGTTTACAGCTGCATCTCCAATACCAA

AATTACCGCTGCCCTTCTGCCCCCCTTTTTCTCCCTTGCGGCGAGCAGGCCAACGGCCGTCGAGATGATTACA
ATTCGAGCTCGCGACGGGGCGGAGACGGTCCCTGGACGGTGTTCGTGCAGGGCGTCATCTCCTCCGCTGGATT
CTCTGCTGCCTGAGCGAGCGAGACCTGACGGTGGGCGCATCATCAGGCCATCGCGGCTTCAGGGAGGCCGGCT
TAGTCATGGCAGGTTAAGCTATCAACGGTCCGTCAATATTAATATTGTTATTATTGTCCGGTGGTGAGTGAGA
AGAGACTGTAATTACATTGCCCAGTGGACGCTATTGTACCCATAGTTATATCACTCAAAAATGAAATCCTAAA
CACGAGAATCGCCGTCGTCTCCAAGGCAGCGACCAAGCCGGTTTTTCTGCTGCTTTTCGGTTAGTTGGCCCCC
TATCACCTTTCAACCGGGGGGTATAGTTAAAGAAAAAATTAAGAAAGATCACAATCAATATAAACGAAAAAGG
AATTGGAAACAAAAGTCCGGAATAGCACGGTATAGCTAGCAGAATACCATCCCAAGACGGATCGGGCTTCTG**C**
TACCTCTTTCTTTCTCGTGGAGTCTTGTCTGCGTCGTCTTGGTGGACAACTGGCTTCCGTGCCTCACTCTCAA
GCTCCAGCGAGTCGTCAGACTGCTCATCGAATAACACCTGGTCTCCACTTCCACCGAGTTCCATGAACTCCTT
TGAGTTCCGGTTGCCAGCCGGTTTGGGCTCCTGGGCTCGAGGCACAGGATGATTCTGGTATATAGTTATGTCC
TGGGAAGCAGGGTCGTCTTCCTTGGTCTTCACCACCTGCTTGACCGTGACTTCATGTATGTCACTCAACAGCC
TGCGGACCATAGTTGGCCGGCGTTCTTTGGACACCCTCAGGCCACCGATGGCCCCGGCTAGGTCATCGGTGCT
GTCTGCCACCCTAGGCTCATCGGGGTCGATGCCAGTGATCCATGGGTGCTGGAGACACTCATCTACTTTCAAA
CGCTCCTTCACGTCGACTTCGATCATGGAGTCGATAAGGTCCATGGCTCTATAATCGATTGAGTCCCAGTATG
GAAGAGGATACTTAAATATTCCGAGCTGGATCTGCTCTTTCTGAGTGTATGGGTAGTCTCTTGGGTGGTACAG
TTCATCTGAGAAAGGAGGGAATCCGCACAGGCAGATGTAGAGTACCACTCCGAGTGACCAGATGTCAACCGCT
CGGGTGTACATTCTGGCTTCAGCGTTTTCTGCCAGTATCTCAGGAGCAACATCTGATAAGAATTGTTAGATTC
ACATTTGGTGGATGAAAGCACCAGCGTTAACAGAGCAGCAGGGAAACTTACAGCCAGGCGTTCCACATCTAGA
TTTTATGATCTCTTAGTAAGATTGACCCCAGATTTGAACGGCAGAAGGTAGGTGAACTTACAGCGTGGTGGTA
AAGGAGTGTTCGCCGACAATCTTTGCAAGGCCGAAGTCTGCTAATTTTACGGTAAGATCTTTGTCGACAACCA
GAATGTTCTCTGGCTTTATGTCACGGTGAATGATGTTTCGTTCATGCTACGACAACGGTTAGCCATATACACT
GCCATGAGTCTGATCAACCAGAAAACGAACGACATACCAGGTATCTCAAACCTTCAAAAAGCTGAATGAATAC
TTTTCGAGTCTCCTCCTCGCTGAGTTTCTGATGCCTAATGATCCAGTTGAACAGTTCGCCCTCTGGCGCAAGC
TCAAGAATCAGGAAGACACCATCGCTCTCGTCAAAGGTATCCTTTAGGCACAGCATGTTCGGATGGTTGACAC
TCATCAGCATTGAAATCTCTTGCTGTAGTGCTTCACGGTCTAGCCTCTGAACTGCGCCCGGGCGTTTCTCGAA
ATGTTTGACGGCATACTTGATCCCCGTAGAACGTTCAGCGCATAGATAAACCGAGGCAAAGTGTCCCCTACCC
AGCATATCAAAGAGCTTATACTGCTGTCGGAACTTGCTTGTGTTCCGACTCCGCGGGTAGTTGAAGATGAATC
TGGATTCTTGCAGGATGGTAATCTCGTCGCCGTTTTCGAGCTCTCGGCGTTTGTTCTTGCCGATGATGGCGTC
GTTGATGAAAGTGCCATTGATAGAGAGATCTTCCACAATTGCAACAAAGTCTCCTCCCCTGTTTTCGTGGAAT
ATCAGGCAATGGCGGTTGGATATCGTGGGGAGCTTGATCCTGAAATCTGCAGTACCCAGATGGTTAGCAATTG
ATCACTAGACGGCAGAGAGTATGCAATGGGTAAAGGCATACCGCACTCTGCATGCCGGCCTATAAGATATCCA
GCGCGGTTTATCTCTCTAGTTCCACGTTTTGAGGCGTTGCTTTCCGGCGTTGATATGTCTGATGCTGCTCCAA
TTACCGGTGTAGTATTTGTGCTGTCCCGCTTCTTCAGAACCAATTGGCCTCCAAAATTGTTATCTAGCGGAGT
AAGATATCCCCATATGCCCTGGGCCACCTCGTCTTCCACATCGTCAATGGATTGGATTGGGGGATACTGAGAG
AAACCCTGGGTGGGACATGACGACTCGTCTGGCGATTGACCTCGAGGCGCCTGGTTTCGGCGGCCTTCTGGGG
GAGAAACTGTAGCAACTTCACGCTTTTCTTCTGAGATTACTTCATTAGCTAGGGGAGAAGGTAGATATCCGGC
ATCCTGCTGCAATTGAGATGAGATTCGAGGAGATCTCCGTGGCTTCTTTGATTCATGGTCTGGAGCTGCGTTG

CCCTGCAGGTTTGGGAAAAGAAGTTAGCAGCTTGAATATTTTCATCCTTCTGAGAAACTAATTGGATATAACG
TGTTCATATCTTACCCTTTCTCTCTTCAGATTGCTTCTCTCCCTCTGCGGAGG**CAT**GTTTTGGCCCGGCGTGA
AGAAGTTGACGGCAGTTACGAATATCCCGGATGGACTGCCAATGGCTTCGCAACTTCAACTCCAAGTCGATGG
CAGGCAAGATCAAGCCCCAACACACTCTACCGTAGTTGCAAGACCGAACAGTGTCGCTGTCGAAGGTGGTAGA
AGCAGTTCAGCAGAGAGAGGCAGCGTGTGAGTCTACCTGCTACCCCCGTACTATGTATATCCCGACTATGAGA
CAGCCGCCGAGGTGTGCGGAGACGAAATAAATAACTAGTTATCTTTCGGCTCCCTGAGTTCCAGGCCGGACAA
GCCCAGGCCAAGCTGCAGCAAGCCCCCAAGCAAGAGAACGACGGCGCTTAGAGTCAATGGCAGGCCAGGCTGA
GCGAACTTCCCTTGCTCGACCTTTTTTTTCTGTCGAAAGATGCGAAGTCGCTCTTTATTAGCGAAGTGGGATC
ATCTCGTAGATGGCCTAGGTAAACAACTGCATATGTACCTTCACCGAGCTTCTTATCTTTCAGAATAACCAAA
TGTTAGCATTATGGGGTCCCAATGGTTGGGGTGTATTTGCCCGCCCACCTTTGACATATTTCCGCCGTACATC
AGCGTTTAACTGCTCCGCCAGCTCTGGTTCGGGCATGGAGGGCTTAGGAACTGCAGGATCACCACTAGGCGCC
GTCCGAGGCTCTTGAGAAGCAATATTTCTCATCGCTAACGACTTTGGAGCACCGGTAGTAGTGATGGGAGGGA
ATTGAGAGGGAAGCGGCGACACTGCCATAATGTCAAGCGAAATTGGCTTCCACCAATTTCGCGAGACAAAGGA
TAGAGTGGCACAAGAGTGAGACACTGCTCTGAGCAGCTCTCCTAGTATTCAATCAACAATACAGCTTTGTTTG
TTATCAAAATATATATCGTCGTCTTTCATCTTTGAATGTTGCGATGGAAGCTTCGGCTGGCCCGAGCTCACCG
GAGATCGGCGTTTTGTTGATCCGAGCTAAGGCCGTCGGACCTAATTCAAAATTTTGGTGCAAAATCTTGAACG
AAGTCGAAGAACCAGTTGAACATCTTCTATACAGCGCCATTGAGCATTGGATTGACTCGCCTTGAGTCCCTTA
CCGGTTTACCTGGGCCCTTGGTGCCGTGGCTTTTTTCGCTTTCGTCACGCCCGCCGATGGTGTTCTTACACCA
CGAGCTAAATTAAAATCTTCACCCCCCAGATCTCGACATTGAACGGTTTGATATAAGGCGATATAACCACT**AT**
GGCTGCGGCGCGCATACCGGGCTTTAATGCCAGAACATCCCTTCCACTACGGCTCTTCACTTCCGTCACAAAC
CCCCATTATCAGCCATGCGTACGGTACCTTCATCAATCTCGAAAACCCGCAACAGTGCCATCACCTACCCCAT
TCGTGCCCGATGTCGAAACATTCCTACGCCTAATTGGGCGAAACATGTCCAGATATTCCAGCAAAATCACCGG
ATGGAAGCAATTGTTTACAGCGGACTCGGCTGAGCTTCGAGAATTGGGCATAGAAATGACAAGGGACAGACGA
TACATACTGCGGTGGAGAGAGAAATTCAGACAGCAGGACTACGGCCCAGGAGGTGATTTCGAAAACGTTGTTG
ACGGTGTTGCTGAGCTGAGAGCTGTTGAGGTCCCCGTCGAAAAGTCAAAGAGTGCAAAAACTACCAATGCCAC
CGACGGAGAGTCCCTAGATGTTAGCACAATGGGCACCGCTACCCTTACACCTGGAATGCGTTATGCGATTGTC
AATCTACCACCAGGCGAAACAGAGCTCAAGGATACTTCCAAGCCACTCAAAAAGTTTGCCCATTATAAATTGC
ACCACGGGAATATGATCAAGGGGCCGTATGCTCAGATTATTCCAAACACATGGAGTACCGCCGTCAAGGTTCA
GGTTACTGATGGCATGTGGGAGCATAAGCGTGGCAGAAAGATTGACGGTGGTCAGAGAAGACAGAAGGAGATC
AGAGCGAAGAGAGCGTTGGCGGAGAGGAGGAATGCTCAGGCAGCTGCT**TAG**GCGTTTTGTAGCGTCGGAGTTG
AAAGGGGGCACGGGATTAACGACGTTGCTGGCTGGTTACTTCCTGACATTACTGGCTTGGATATCGCTGTTTA
GATGCTAGATTATCCCAGCTACGGGAAGATGTACTATTTCCAATGTATATTACCATTTTGTTTCTGCACCAAA
GCTAACGGAGTAACTGCAACTTTCCTGTTCATCTAGAGTATTGTAATAAATGGCTGTATTAGAATAATACTAT
TATCTACATCCCCCATCTTCAAAAGCATGGTCAAAAGAAAGTTTTTATTACATATGCTCTATTCCGAAAACCT
ATTTGCGTGACCATAAAGTGCAAATATGGCTCGGTGTATAAACGTAAGAAAAGAGAAGAAATAAATTAGCAAG
AAGCAAGAAATTTAACATGATTCAATCCGCAGTCGTGATTA**TCA**TTTCTGGGAAGGCTGCTTTCGCTTCTCCC
ACGAGACCATTGGGTCGCCAAGGGGCTTCGAGAAGAGGGACTATTAATGCAAAGTTAGTAAAATCGAGCAAAA
TAAAAAGGAGCGTAATATGCCAAACTTACCGAATAGTCAGTTGGCGGCACAACATCTTTGGTGCCTTCAACTT

GCTTCCATTTCATTAGTCTGGCTAACGTAGCTGTTACGGTTCCAAGCTGAACATATGCAAATTGTTCACCGAT
GCATCTGTGTCTGCCTGAGCCGAATGGGAGGTAAGGGCTGTTGGCACCCTTGCTAACCAAACCGTATCCGTAA
TCTGTCTTTTCTTCACTGGCCTCGACTACGGTGACACCGACGTTGTTCTCCCATCGGTGAGGGTTCCAAACAA
GAGGGTCAGGGAAGTACTGAGGGTCTCGTGAGGGAACACCAGGAGCGGCAAGGAGGTTGTGAGAGGTTGGGAC
AACATAGTTAGTTCCTTCAACGGGCATAGGGGATTTAACAGCACGGAGGATAGAGTGGATGGGAGCGTGGAGG
CGGAGAGTCTCCTTGATTACATTGTTGTGAAGATCAAGTTTCTGGAGAGATTCGTAGGTGAGTGGTGGAAGAT
CCTCGCCGAGAACACGTTTCTGTTCCTCGTAGAGTTCCTCGAGAATATCTGGACGGCTGGCAAGGCGGAGAAC
GATCCAGGAGCCGGTGGAGGAAGAAGAGTGCTGGCCAGCCATAAGAAGAGCAATCATCATGTGGGCGACTTCG
ATATCTGGAATTGGCGTTCCATTCTTGTACACGGACGACATCAAGTTCCATACCATGTCCTCGGAGTCTTTCT
CTCCACCAGCCTCACGTCGCTGACGGATGATGTCGGTGTAAACCTGGGCCATCTTCTTTTGAGCACGGTCACG
CTTGCGGTTGTGTGGGAAGGGGAACCATGGGAACATGAAGTTGATGGCGGCGAAGCCCATGTCGAGATCGGAG
TAGAGTTCCGCAAAGCTGGAATCGAACTTTCCTCGCACCTCCTTGCCCTGTAGAGATCGAGAGGCGGTGTAGA
TGGTGATTTCAGCCATGACCTTGCTGACGTTGAAAACTCCGGAGTCGCCCTTGAAGACGGGGGAGGACTCGAA
GAACTGCTCAACTTCCTTGGTGATCAGGGTGACATAGGATCGGAGAGCTTCAGAGGTGAGGCCGAACTTGACG
AACTGGTAGACGATTAAATTAGCAATGGAGGCCCAGGAGATGTAGACGGGAATAAATTGCTGTACACACCTTC
TTCTGCTCCATGAGCTTGGAGTTTGGGCAATCATACACCACATGTCGTCCGAACACTGGGGTGGTGAGGGGGG
AGTAGACATCCTCCGCGCAAACATCCTTGAGCTTGCCGTTCAAAATGAAATCATTTCCCTTTGTACCGAGAAA
AACAGTCGTCTTCTTGCCCAGCAGTATGAATGTGAAGATGTCTCCATACTAGAGTACCGAAATTATTTATACG
TTAGCTTTGCGCAACCTGGTATATAGCTAAGTTGAATTGCACCAACTAACCTTCTCCTTGCAGTCGTCAAAGA
ACTTGTACGGGTCAATTCCATAGGAGATCGTGCTTCCAATGACCGGAACCCAGTGGAAGACCACCGGAGGCTT
TGTAGGGTCCTTGAACAGGAGCTGCTGCAGGACGTTGATAACAATGGAGAGGACGATAAAAGCCGATACGGCA
CTTGCGACGAGCGCGGCGGTGGACAGGGTCGAGCAGTTCTCGCAGAAACGAGAGACAATGTCGGCTAAGAGGC
C**CAT**GGTGATCGTCTTATCCTCCTGCTTCCGCTCAAGACTTGTTCGTTGTTTGTGATGAATGCCTTGAAGCTG
CTGCGGACGAAGCAAGGGAAAAAAAAAGTTGCATCTTGAGATCGAACTCGCATTTTATGCAAGGGGCGTACGG
TACTGTAAGATAATGCATTGTTCCTGGTATTTTACGTCGTTTTTTATTGGTTTACAGCTGCATCTCCAATACC
AAAATTACCGCTGCCCTTCTGCCCCCCTTTTTCTCCCTTGCGGCGAGCAGGCCAACGGCCGTCGAGATGATTA
CAATTCGAGCTCGCGACGGGGCGGAGACGGTCCCTGGACGGTGTTCGTGCAGGGCGTCATCTCCTCCGCTGGA
TTCTCTGCTGCCTGAGCGAGCGAGACCTGACGGTGGGCGCATCATCAGGCCATCGCGGCTTCAGGGAGGCCGG
CTTAGTCATGGCAGGTTAAGCTATCAACGGTCCGTCAATATTAATATTGTTATTATTGTCCGGTGGTGAGTGA
GAAGAGACTGTAATTACATTGCCCAGTGGACGCTATTGTACCCATAGTTATATCACTCAAAAATGAAATCCTA
AACACGAGAATCGCCGTCGTCTCCAAGGCAGCGACCAAGCCGGTTTTTCTGCTGCTTTTCGGTTAGTTGGCCC
CCTATCACCTTTCAACCGGGGGGTATAGTTAAAGAAAAAATTAAGAAAGATCACAATCAATATAAACGAAAAA
GGAATTGGAAACAAAAGTCCGGAATAGCACGGTATAGCTAGCAGAATACCATCCCAAGACGGATCGGGCTTCT
G**CTA**CCTCTTTCTTTCTCGTGGAGTCTTGTCTGCGTCGTCTTGGTGGACAACTGGCTTCCGTGCCTCACTCTC
AAGCTCCAGCGAGTCGTCAGACTGCTCATCGAATAACACCTGGTCTCCACTTCCACCGAGTTCCATGAACTCC
TTTGAGTTCCGGTTGCCAGCCGGTTTGGGCTCCTGGGCTCGAGGCACAGGATGATTCTGGTATATAGTTATGT
CCTGGGAAGCAGGGTCGTCTTCCTTGGTCTTCACCACCTGCTTGACCGTGACTTCATGTATGTCACTCAACAG
CCTGCGGACCATAGTTGGCCGGCGTTCTTTGGACACCCTCAGGCCACCGATGGCCCCGGCTAGGTCATCGGTG

CTGTCTGCCACCCTAGGCTCATCGGGGTCGATGCCAGTGATCCATGGGTGCTGGAGACACTCATCTACTTTCA
AACGCTCCTTCACGTCGACTTCGATCATGGAGTCGATAAGGTCCATGGCTCTATAATCGATTGAGTCCCAGTA
TGGAAGAGGATACTTAAATATTCCGAGCTGGATCTGCTCTTTCTGAGTGTATGGGTAGTCTCTTGGGTGGTAC
AGTTCATCTGAGAAAGGAGGGAATCCGCACAGGCAGATGTAGAGTACCACTCCGAGTGACCAGATGTCAACCG
CTCGGGTGTACATTCTGGCTTCAGCGTTTTCTGCCAGTATCTCAGGAGCAACATCTGATAAGAATTGTTAGAT
TCACATTTGGTGGATGAAAGCACCAGCGTTAACAGAGCAGCAGGGAAACTTACAGCCAGGCGTTCCACATCTA
GATTTTATGATCTCTTAGTAAGATTGACCCCAGATTTGAACGGCAGAAGGTAGGTGAACTTACAGCGTGGTGG
TAAAGGAGTGTTCGCCGACAATCTTTGCAAGGCCGAAGTCTGCTAATTTTACGGTAAGATCTTTGTCGACAAC
CAGAATGTTCTCTGGCTTTATGTCACGGTGAATGATGTTTCGTTCATGCTACGACAACGGTTAGCCATATACA
CTGCCATGAGTCTGATCAACCAGAAAACGAACGACATACCAGGTATCTCAAACCTTCAAAAAGCTGAATGAAT
ACTTTTCGAGTCTCCTCCTCGCTGAGTTTCTGATGCCTAATGATCCAGTTGAACAGTTCGCCCTCTGGCGCAA
GCTCAAGAATCAGGAAGACACCATCGCTCTCGTCAAAGGTATCCTTTAGGCACAGCATGTTCGGATGGTTGAC
ACTCATCAGCATTGAAATCTCTTGCTGTAGTGCTTCACGGTCTAGCCTCTGAACTGCGCCCGGGCGTTTCTCG
AAATGTTTGACGGCATACTTGATCCCCGTAGAACGTTCAGCGCATAGATAAACCGAGGCAAAGTGTCCCCTAC
CCAGCATATCAAAGAGCTTATACTGCTGTCGGAACTTGCTTGTGTTCCGACTCCGCGGGTAGTTGAAGATGAA
TCTGGATTCTTGCAGGATGGTAATCTCGTCGCCGTTTTCGAGCTCTCGGCGTTTGTTCTTGCCGATGATGGCG
TCGTTGATGAAAGTGCCATTGATAGAGAGATCTTCCACAATTGCAACAAAGTCTCCTCCCCTGTTTTCGTGGA
ATATCAGGCAATGGCGGTTGGATATCGTGGGGAGCTTGATCCTGAAATCTGCAGTACCCAGATGGTTAGCAAT
TGATCACTAGACGGCAGAGAGTATGCAATGGGTAAAGGCATACCGCACTCTGCATGCCGGCCTATAAGATATC
CAGCGCGGTTTATCTCTCTAGTTCCACGTTTTGAGGCGTTGCTTTCCGGCGTTGATATGTCTGATGCTGCTCC
AATTACCGGTGTAGTATTTGTGCTGTCCCGCTTCTTCAGAACCAATTGGCCTCCAAAATTGTTATCTAGCGGA
GTAAGATATCCCCATATGCCCTGGGCCACCTCGTCTTCCACATCGTCAATGGATTGGATTGGGGGATACTGAG
AGAAACCCTGGGTGGGACATGACGACTCGTCTGGCGATTGACCTCGAGGCGCCTGGTTTCGGCGGCCTTCTGG
GGGAGAAACTGTAGCAACTTCACGCTTTTCTTCTGAGATTACTTCATTAGCTAGGGGAGAAGGTAGATATCCG
GCATCCTGCTGCAATTGAGATGAGATTCGAGGAGATCTCCGTGGCTTCTTTGATTCATGGTCTGGAGCTGCGT
TGCCCTGCAGGTTTGGGAAAAGAAGTTAGCAGCTTGAATATTTTCATCCTTCTGAGAAACTAATTGGATATAA
CGTGTTCATATCTTACCCTTTCTCTCTTCAGATTGCTTCTCTCCCTCTGCGGAGG**CAT**GTTTTGGCCCGGCGT
GAAGAAGTTGACGGCAGTTACGAATATCCCGGATGGACTGCCAATGGCTTCGCAACTTCAACTCCAAGTCGAT
GGCAGGCAAGATCAAGCCCCAACACACTCTACCGTAGTTGCAAGACCGAACAGTGTCGCTGTCGAAGGTGGTA
GAAGCAGTTCAGCAGAGAGAGGCAGCGTGTGAGTCTACCTGCTACCCCGTACTATGTATATCCCGACTATGAG
ACAGCCGCCGAGGTGTGCGGAGACGAAATAAATAACTAGTTATCTTTCGGCTCCCTGAGTTCCAGGCCGGACA
AGCCCAGGCCAAGCTGCAGCAAGCCCCCAAGCAAGAGAACGACGGCGCTTAGAGTCAATGGCAGGCCAGGCTG
AGCGAACTTCCCTTGCTCGACCTTTTTTTTCTGTCGAAAGATGCGAAGTCGCTCTTTATTAGCGAAGTGGGAT
CATCTCGTAGATGGCCTAGGTAAACAACTGCATATGTACCTTCACCGAGCTTCTTATCTTTCAGAATAACCAA
ATGTTAGCATTATGGGGTCCCAATGGTTGGGGTGTATTTGCCCGCCCACCTTTGACATATTTCCGCCGTACAT
CAGCGTTTAACTGCTCCGCCAGCTCTGGTTCGGGCATGGAGGGCTTAGGAACTGCAGGATCACCACTAGGCGC
CGTCCGAGGCTCTTGAGAAGCAATATTTCTCATCGCTAACGACTTTGGAGCACCGGTAGTAGTGATGGGAGG
AATTGAGAGGGAAGCGGCGACACTGCCATAATGTCAAGCGAAATTGGCTTCCACCAATTTCGCGAGACAAAGG

ATAGAGTGGCACAAGAGTGAGACACTGCTCTGAGCAGCTCTCCTAGTATTCAATCAACAATACAGCTTTGTTT
GTTATCAAAATATATATCGTCGTCTTTCATCTTTGAATGTTGCGATGGAAGCTTCGGCTGGCCCGAGCTCACC
GGAGATCGGCGTTTTGTTGATCCGAGCTAAGGCCGTCGGACCTAATTCAAATTTTGGTGCAAAATCTTGAACG
AAGTCGAAGAACCAGTTGAACATCTTCTATACAGCGCCATTGAGCATTGGATTGACTCGCCTTGAGTCCCTTA
CCGGTTTACCTGGGCCCTTGGTGCCGTGGCTTTTTTCGCTTTCGTCACGCCCGCCGATGGTGTTCTTACACCA
CGAGCTAAATTAAAATCTTCACCCCCCAGATCTCGACATTGAACGGTTTGATATAAGGCGATATAACCACT**AT**
GGCTGCGGCGCGCATACCGGGCTTTAATGCCAGAACATCCCTTCCACTACGGCTCTTCACTTCCGTCACAAAC
CCCCATTATCAGCCATGCGTACGGTACCTTCATCAATCTCGAAAACCCGCAACAGTGCCATCACCTACCCCAT
TCGTGCCCGATGTCGAAACATTCCTACGCCTAATTGGGCGAAACATGTCCAGATATTCCAGCAAAATCACCGG
ATGGAAGCAATTGTTTACAGCGGACTCGGCTGAGCTTCGAGAATTGGGCATAGAAATGACAAGGGACAGACGA
TACATACTGCGGTGGAGAGAGAAATTCAGACAGCAGGACTACGGCCCAGGAGGTGATTTCGAAAACGTTGTTG
ACGGTGTTGCTGAGCTGAGAGCTGTTGAGGTCCCCGTCGAAAAGTCAAAGAGTGCAAAAACTACCAATGCCAC
CGACGGAGAGTCCCTAGATGTTAGCACAATGGGCACCGCTACCCTTACACCTGGAATGCGTTATGCGATTGTC
AATCTACCACCAGGCGAAACAGAGCTCAAGGATACTTCCAAGCCACTCAAAAAGTTTGCCCATTATAAATTGC
ACCACGGGAATATGATCAAGGGGCCGTATGCTCAGATTATTCCAAACACATGGAGTACCGCCGTCAAGGTTCA
GGTTACTGATGGCATGTGGGAGCATAAGCGTGGCAGAAAGATTGACGGTGGTCAGAGAAGACAGAAGGAGATC
AGAGCGAAGAGAGCGTTGGCGGAGAGGAGGAATGCTCAGGCAGCTGCT**TAG**GCGTTTTGTAGCGTCGGAGTTG
AAAGGGGGCACGGGATTAACGACGTTGCTGGCTGGTTACTTCCTGACATTACTGGCTTGGATATCGCTGTTTA
GATGCTAGATTATCCCAGCTACGGGAAGATGTACTATTTCCAATGTATATTACCATTTTGTTTCTGCACCAAA
GCTAACGGAGTAACTGCAACTTTCCTGTTCATCTAGAGTATTGTAATAAATGGCTGTATTAGAATAATACTAT
TATCTACATCCCCCATCTTCAAAAGCATGGTCAAAAGAAAGTTTTTATTACATATGCTCTATTCCGAAAACCT
ATTTGCGTGACCATAAAGTGCAAATATGGCTCGGTGTATAAACGTAAGAAAAGAGAAGAAATAAATTAGCAAG
AAGCAAGAAATTTAACATGATTCAATCCGCAGTCGTGATTA**TCA**TTTCTGGGAAGGCTGCTTTCGCTTCTCCC
ACGAGACCATTGGGTCGCCAAGGGGCTTCGAGAAGAGGGACTATTAATGCAAAGTTAGTAAAATCGAGCAAAA
TAAAAAGGAGCGTAATATGCCAAACTTACCGAATAGTCAGTTGGCGGCACAACATCTTTGGTGCCTTCAACTT
GCTTCCATTTCATTAGTCTGGCTAACGTAGCTGTTACGGTTCCAAGCTGAACATATGCAAATTGTTCACCGAT
GCATCTGTGTCTGCCTGAGCCGAATGGGAGGTAAGGGCTGTTGGCACCCTTGCTAACCAAACCGTATCCGTAA
TCTGTCTTTTCTTCACTGGCCTCGACTACGGTGACACCGACGTTGTTCTCCCATCGGTGAGGGTTCCAAACAA
GAGGGTCAGGGAAGTACTGAGGGTCTCGTGAGGGAACACCAGGAGCGGCAAGGAGGTTGTGAGAGGTTGGGAC
AACATAGTTAGTTCCTTCAACGGGCATAGGGGATTTAACAGCACGGAGGATAGAGTGGATGGGAGCGTGGAGG
CGGAGAGTCTCCTTGATTACATTGTTGTGAAGATCAAGTTTCTGGAGAGATTCGTAGGTGAGTGGTGGAAGAT
CCTCGCCGAGAACACGTTTCTGTTCCTCGTAGAGTTCCTCGAGAATATCTGGACGGCTGGCAAGGCGGAGAAC
GATCCAGGAGCCGGTGGAGGAAGAAGAGTGCTGGCCAGCCATAAGAAGAGCAATCATCATGTGGGCGACTTCG
ATATCTGGAATTGGCGTTCCATTCTTGTACACGGACGACATCAAGTTCCATACCATGTCCTCGGAGTCTTTCT
CTCCACCAGCCTCACGTCGCTGACGGATGATGTCGGTGTAAACCTGGGCCATCTTCTTTTGAGCACGGTCACG
CTTGCGGTTGTGTGGGAAGGGGAACCATGGGAACATGAAGTTGATGGCGGCGAAGCCCATGTCGAGATCGGAG
TAGAGTTCCGCAAAGCTGGAATCGAACTTTCCTCGCACCTCCTTGCCCTGTAGAGATCGAGAGGCGGTGTAGA
TGGTGATTTCAGCCATGACCTTGCTGACGTTGAAAACTCCGGAGTCGCCCTTGAAGACGGGGGAGGACTCGAA

GAACTGCTCAACTTCCTTGGTGATCAGGGTGACATAGGATCGGAGAGCTTCAGAGGTGAGGCCGAACTTGACG
AACTGGTAGACGATTAAATTAGCAATGGAGGCCCAGGAGATGTAGACGGGAATAAATTGCTGTACACACCTTC
TTCTGCTCCATGAGCTTGGAGTTTGGGCAATCATACACCACATGTCGTCCGAACACTGGGGTGGTGAGGGGGG
AGTAGACATCCTCCGCGCAAACATCCTTGAGCTTGCCGTTCAAAATGAAATCATTTCCCTTTGTACCGAGAAA
AACAGTCGTCTTCTTGCCCAGCAGTATGAATGTGAAGATGTCTCCATACTAGAGTACCGAAATTATTTATACG
TTAGCTTTGCGCAACCTGGTATATAGCTAAGTTGAATTGCACCAACTAACCTTCTCCTTGCAGTCGTCAAAGA
ACTTGTACGGGTCAATTCCATAGGAGATCGTGCTTCCAATGACCGGAACCCAGTGGAAG

[Read No.m64281e_230311_112734/4915739/ccs] [Sense (+) strand]

AAT~ACC (5'UTR)

ATG~TAG (*chkB* protein kinase genomic DNA) (Forward sequence)

ATG~TGA (*TinCYP51B* gene) (Forward sequence)

TAA~GCT (3'UTR)

CTA~CAT (*FYV4* genomic DNA) (Reverse sequence)

CGT~ (Downstream sequence of the 7374 block)

TACAGTACCGTACGCCCCTTGCATAAAATGCGAGTTCGATCTCAAGATGCAACTTTTTTTTTCCCTTGCTTCG
TCCGCAGCAGCTTCAAGGCATTCATCACAAACAACGAACAAGTCTTGAGCGGAAGCAGGAGGATAAGACGATC
ACC**ATG**GGCCTCTTAGCCGACATTGTCTCTCGTTTCTGCGAGAACTGCTCGACCCTGTCCACCGCCGCGCTCG
TCGCAAGTGCCGTATCGGCTTTTATCGTCCTCTCCATTGTTATCAACGTCCTGCAGCAGCTCCTGTTCAAGGA
CCCTACAAAGCCTCCGGTGGTCTTCCACTGGGTTCCGGTCATTGGAAGCACGATCTCCTATGGAATTGACCCG
TACAAGTTCTTTGACGACTGCAAGGAGAAGGTTAGTTGGTGCAATTCAACTTAGCTATATACCAGGTTGCGCA
AAGCTAACGTATAAATAATTTCGGTACTCTAGTATGGAGACATCTTCACATTCATACTGCTGGGCAAGAAGAC
GACTGTTTTTCTCGGTACAAAGGGAAATGATTTCATTTTGAACGGCAAGCTCAAGGATGTTTGCGCGGAGGAT
GTCTACTCCCCCCTCACCACCCCAGTGTTCGGACGACATGTGGTGTATGATTGCCCAAACTCCAAGCTCATGG
AGCAGAAGAAGGTGTGTACAGCAATTTATTCCCGTCTACATCTCCTGGGCCTCCATTGCTAATTTAATCGTCT
ACCAGTTCGTCAAGTTCGGCCTCACCTCTGAAGCTCTCCGATCCTATGTCACCCTGATCACCAAGGAAGTTGA
GCAGTTCTTCGAGTCCTCCCCCGTCTTCAAGGGCGACTCCGGAGTTTTCAACGTCAGCAAGGTCATGGCTGAA
ATCACCATCTACACCGCCTCTCGATCTCTACAGGGCAAGGAGGTGCGAGGAAAGTTCGATTCCAGCTTTGCGG
AACTCTACTCCGATCTCGACATGGGCTTCGCCGCCATCAACTTCATGTTCCCATGGTTCCCCTTCCCACACAA
CCGCAAGCGTGACCGTGCTCAAAAGAAGATGGCCCAGGTTTACACCGACATCATCCGTCAGCGACGTGAGGCT
GGTGGAGAGAAAGACTCCGAGGACATGGTATGGAACTTGATGTCGTCCGTGTACAAGAATGGAACGCCAATTC
CAGATATCGAAGTCGCCCACATGATGATTGCTCTTCTTATGGCTGGCCAGCACTCTTCTTCCTCCACCGGCTC
CTGGATCGTTCTCCGCCTTGCCAGCCGTCCAGATATTCTCGAGGAACTCTACGAGGAACAGAAACGTGTTCTC
GGCGAGGATCTTCCACCACTCACCTACGAATCTCTCCAGAAACTTGATCTTCACAACAATGTAATCAAGGAGA
CTCTCCGCCTCCACGCTCCCATCCACTCTATCCTCCGTGCTGTTAAATCCCCTATGCCCGTTGAAGGAACTAA
CTATGTTGTCCCAACCTCTCACAACCTCCTTGCCGCTCCTGGTGTTCCCTCACGAGACCCTCAGTACTTCCCT

GACCCTCTTGTTTGGAACCCTCACCGATGGGAGAACAACGTCGGTGTCACCGTAGTCGAGGCCAGTGAAGAAA
AGACAGATTACGGATACGGTTTGGTTAGCAAGGGTGCCAACAGCCCTTACCTCCCATTCGGCTCAGGCAGACA
CAGATGCATCGGTGAACAATTTGCATATGTTCAGCTTGGAACCGTAACAGCTACGTTAGCCAGACTAATGAAA
TGGAAGCAAGTTGAAGGCACCAAAGATGTTGTGCCGCCAACTGACTATTCGGTAAGTTTGGCATATTACGCTC
CTTTTTATTTTGCTCGATTTTACTAACTTTGCATTAATAGTCCCTCTTCTCGAAGCCCCTTGGCGACCCAATG
GTCTCGTGGGAGAAGCGAAAGCAGCCTTCCCAGAAA**TGA**TAATCACGACTGCGGATTGAATCATGTTAAATTT
CTTGCTTCTTGCTAATTTATTTCTTCTCTTTTCTTACGTTTATACACCGAGCCATATTTGCACTTTATGGTCA
CGCAAATAGGTTTTCGGAATAGAGCATATGTAATAAAAACTTTCTTTTGACCATGCTTTTGAAGATGGGGGAT
GTAGATAATAGTATTATTCTAATACAGCCATTTATTACAATACTCTAGATGAACAGGAAAGTTGCAGTTACTC
CGTTAGCTTTGGTGCAGAAACAAAATGGTAATATACATTGGAAATAGTACATCTTCCCGTAGCTGGGATAATC
TAGCATCTAAACAGCGATATCCAAGCCAGTAATGTCAGGAAGTAACCAGCCAGCAACGTCGTTAATCCCGTGC
CCCCTTTCAACTCCGACGCTACAAAACGC**CTA**AGCAGCTGCCTGAGCATTCCTCCTCTCCGCCAACGCTCTCT
TCGCTCTGATCTCCTTCTGTCTTCTCTGACCACCGTCAATCTTTCTGCCACGCTTATGCTCCCACATGCCATC
AGTAACCTGAACCTTGACGGCGGTACTCCATGTGTTTGGAATAATCTGAGCATACGGCCCCTTGATCATATTC
CCGTGGTGCAATTTATAATGGGCAAACTTTTTGAGTGGCTTGGAAGTATCCTTGAGCTCTGTTTCGCCTGGTG
GTAGATTGACAATCGCATAACGCATTCCAGGTGTAAGGGTAGCGGTGCCCATTGTGCTAACATCTAGGGACTC
TCCGTCGGTGGCATTGGTAGTTTTTGCACTCTTTGACTTTTCGACGGGGACCTCAACAGCTCTCAGCTCAGCA
ACACCGTCAACAACGTTTTCGAAATCACCTCCTGGGCCGTAGTCCTGCTGTCTGAATTTCTCTCTCCACCGCA
GTATGTATCGTCTGTCCCTTGTCATTTCTATGCCCAATTCTCGAAGCTCAGCCGAGTCCGCTGTAAACAATTG
CTTCCATCCGGTGATTTTGCTGGAATATCTGGACATGTTTCGCCCAATTAGGCGTAGGAATGTTTCGACATCG
GGCACGAATGGGGTAGGTGATGGCACTGTTGCGGGTTTTCGAGATTGATGAAGGTACCGTACGCATGGCTGAT
AATGGGGGTTTGTGACGGAAGTGAAGAGCCGTAGTGGAAGGGATGTTCTGGCATTAAAGCCCGGTATGCGCGC
CGCAGC**CAT**AGTGGTTATATCGCCTTATATCAAACCGTTCAATGTCGAGATCTGGGGGGTGAAGATTTTAATT
TAGCTCGTGGTGTAAGAACACCATCGGCGGGCGTGACGAAAGCGAAAAAAGCCACGGCACCAAGGGCCCAGGT
AAACCGGTAAGGGACTCAAGGCGAGTCAATCCAATGCTCAATGGCGCTGTATAGAAGATGTTCAACTGGTTCT
TCGACTTCGTTCAAGATTTTGCACCAAAATTTTGAATTAGGTCCGACGGCCTTAGCTCGGATCAACAAAACGC
CGATCTCCGGTGAGCTCGGGCCAGCCGAAGCTTCCATCGCAACATTCAAAGATGAAAGACGACGATATATATT
TTGATAACAAACAAAGCTGTATTGTTGATTGAATACTAGGAGAGCTGCTCAGAGCAGTGTCTCACTCTTGTGC
CACTCTATCCTTTGTCTCGCGAAATTGGTGGAAGCCAATTTCGCTTGACATTATGGCAGTGTCGCCGCTTCCC
TCTCAATTCCCTCCCATCACTACTACCGGTGCTCCAAAGTCGTTAGCGATGAGAAATATTGCTTCTCAAGAGC
CTCGGACGGCGCCTAGTGGTGATCCTGCAGTTCCTAAGCCCTCCATGCCCGAACCAGAGCTGGCGGAGCAGTT
AAACGCTGATGTACGGCGGAAATATGTCAAAGGTGGGCGGGCAAATACACCCCAACCATTGGGACCCCATAAT
GCTAACATTTGGTTATTCTGAAAGATAAGAAGCTCGGTGAAGGTACATATGCAGTTGTTTACCTAGGCCATCT
ACGAGATGATCCCACTTCGCTAATAAAGAGCGACTTCGCATCTTTCGACAGAAAAAAAAGGTCGAGCAAGGGA
AGTTCGCTCAGCCTGGCCTGCCATTGACTCTAAGCGCCGTCGTTCTCTTGCTTGGGGGCTTGCTGCAGCTTGG
CCTGGGCTTGTCCGGCCTGGAACTCAGGGAGCCGAAAGATAACTAGTTATTTATTTCGTCTCCGCACACCTCG
GCGGCTGTCTCATAGTCGGGATATACATAGTACGGGGTAGCAGGTAGACTCACACGCTGCCTCTCTCTGCTGA
ACTGCTTCTACCACCTTCGACAGCGACACTGTTCGGTCTTGCAACTACGGTAGAGTGTGTTGGGGCTTGATCT

TGCCTGCCATCGACTTGGAGTTGAAGTTGCGAAGCCATTGGCAGTCCATCCGGGATATTCGTAACTGCCGTCA
ACTTCTTCACGCCGGGCTAAAAC**ATG**CCTCCGCAGAGGGAGAGAAGCAATCTGAAGAGAGAAAGGGTAAGATA
TGAACACGTTATATCCAATTAGTTTCTCAGAAGGATGAAAATATTCAAGCTGCTAACTTCTTTTCCCAAACCT
GCAGGGCAACGCAGCTCCAGACCATGAATCAAAGAAGCCACGGAGATCTCCTCGAATCTCATCTCAATTGCAG
CAGGATGCCGGATATCTACCTTCTCCCCTAGCTAATGAAGTAATCTCAGAAGAAAAGCGTGAAGTTGCTACAG
TTTCTCCCCCAGAAGGCCGCCGAAACCAGGCGCCTCGAGGTCAATCGCCAGACGAGTCGTCATGTCCCACCCA
GGGTTTCTCTCAGTATCCCCCAATCCAATCCATTGACGATGTGGAAGACGAGGTGGCCCAGGGCATATGGGGA
TATCTTACTCCGCTAGATAACAATTTTGGAGGCCAATTGGTTCTGAAGAAGCGGGACAGCACAAATACTACAC
CGGTAATTGGAGCAGCATCAGACATATCAACGCCGGAAAGCAACGCCTCAAAACGTGGAACTAGAGAGATAAA
CCGCGCTGGATATCTTATAGGCCGGCATGCAGAGTGCGGTATGCCTTTACCCATTGCATACTCTCTGCCGTCT
AGTGATCAATTGCTAACCATCTGGGTACTGCAGATTTCAGGATCAAGCTCCCCACGATATCCAACCGCCATTG
CCTGATATTCCACGAAAACAGGGGAGGAGACTTTGTTGCAATTGTGGAAGATCTCTCTATCAATGGCACTTTC
ATCAACGACGCCATCATCGGCAAGAACAAACGCCGAGAGCTCGAAAACGGCGACGAGATTACCATCCTGCAAG
AATCCAGATTCATCTTCAACTACCCGCGGAGTCGGAACACAAGCAAGTTCCGACAGCAGTATAAGCTCTTTGA
TATGCTGGGTAGGGGACACTTTGCCTCGGTTTATCTATGCGCTGAACGTTCTACGGGGATCAAGTATGCCGTC
AAACATTTCGAGAAACGCCCGGGCGCAGTTCAGAGGCTAGACCGTGAAGCACTACAGCAAGAGATTTCAATGC
TGATGAGTGTCAACCATCCGAACATGCTGTGCCTAAAGGATACCTTTGACGAGAGCGATGGTGTCTTCCTGAT
TCTTGAGCTTGCGCCAGAGGGCGAACTGTTCAACTGGATCATTAGGCATCAGAAACTCAGCGAGGAGGAGACT
CGAAAAGTATTCATTCAGCTTTTTGAAGGTTTGAGATACCTGGTATGTCGTTCGTTTTCTGGTTGATCAGACT
CATGGCAGTGTATATGGCTAACCGTTGTCGTAGCATGAACGAAACATCATTCACCGTGACATAAAGCCAGAGA
ACATTCTGGTTGTCGACAAAGATCTTACCGTAAAATTAGCAGACTTCGGCCTTGCAAAGATTGTCGGCGAACA
CTCCTTTACCACCACGCTGTAAGTTCACCTACCTTCTGCCGTTCAAATCTGGGGTCAATCTTACTAAGAGATC
ATAAAATCTAGATGTGGAACGCCTGGCTGTAAGTTTCCCTGCTGCTCTGTTAACGCTGGTGCTTTCATCCACC
AAATGTGAATCTAACAATTCTTATCAGATGTTGCTCCTGAGATACTGGCAGAAAACGCTGAAGCCAGAATGTA
CACCCGAGCGGTTGACATCTGGTCACTCGGAGTGGTACTCTACATCTGCCTGTGCGGATTCCCTCCTTTCTCA
GATGAACTGTACCACCCAAGAGACTACCCATACACTCAGAAAGAGCAGATCCAGCTCGGAATATTTAAGTATC
CTCTTCCATACTGGGACTCAATCGATTATAGAGCCATGGACCTTATCGACTCCATGATCGAAGTCGACGTGAA
GGAGCGTTTGAAAGTAGATGAGTGTCTCCAGCACCCATGGATCACTGGCATCGACCCCGATGAGCCTAGGGTG
GCAGACAGCACCGATGACCTAGCCGGGGCCATCGGTGGCCTGAGGGTGTCCAAAGAACGCCGGCCAACTATGG
TCCGCAGGCTGTTGAGTGACATACATGAAGTCACGGTCAAGCAGGTGGTGAAGACCAAGGAAGACGACCCTGC
TTCCCAGGACATAACTATATACCAGAATCATCCTGTGCCTCGAGCCCAGGAGCCCAAACCGGCTGGCAACCGG
AACTCAAAGGAGTTCATGGAACTCGGTGGAAGTGGAGACCAGGTGTTATTCGATGAGCAGTCTGACGACTCGC
TGGAGCTTGAGAGTGAGGCACGGAAGCCAGTTGTCCACCAAGACGACGCAGACAAGACTCCACGAGAAAGAAA
GAGG**TAG**CAGAAGCCCGATCCGTCTTGGGATGGTATTCTGCTAGCTATACCGTGCTATTCCGGACTTTTGTTT
CCAATTCCTTTTTCGTTTATATTGATTGTGATCTTTCTTAATTTTTTCTTTAACTATACCCCCCGGTTGAAAG
GTGATAGGGGGCCAACTAACCGAAAAGCAGCAGAAAAACCGGCTTGGTCGCTGCCTTGGAGACGACGGCGATT
CTCGTGTTTAGGATTTCATTTTTGAGTGATATAACTATGGGTACAATAGCGTCCACTGGGCAATGTAATTACA
GTCTCTTCTCACTCACCACCGGACAATAATAACAATATTAATATTGACGGACCGTTGATAGCTTAACCTGCCA

TGACTAAGCCGGCCTCCCTGAAGCCGCGATGGCCTGATGATGCGCCCACCGTCAGGTCTCGCTCGCTCAGGCA
GCAGAGAATCCAGCGGAGGAGATGACGCCCTGCACGAACACCGTCCAGGGACCGTCTCCGCCCCGTCGCGAGC
TCGAATTGTAATCATCTCGACGGCCGTTGGCCTGCTCGCCGCAAGGGAGAAAAAGGGGGGCAGAAGGGCAGCG
GTAATTTTGGTATTGGAGATGCAGCTGTAAACCAATAAAAAACGACGTAAAATACCAGGAACAATGCATTATC
TTACAGTACCGTACGCCCCTTGCATAAAATGCGAGTTCGATCTCAAGATGCAACTTTTTTTTTCCCTTGCTTC
GTCCGCAGCAGCTTCAAGGCATTCATCACAAACAACGAACAAGTCTTGAGCGGAAGCAGGAGGATAAGACGAT
CACC**ATG**GGCCTCTTAGCCGACATTGTCTCTCGTTTCTGCGAGAACTGCTCGACCCTGTCCACCGCCGCGCTC
GTCGCAAGTGCCGTATCGGCTTTTATCGTCCTCTCCATTGTTATCAACGTCCTGCAGCAGCTCCTGTTCAAGG
ACCCTACAAAGCCTCCGGTGGTCTTCCACTGGGTTCCGGTCATTGGAAGCACGATCTCCTATGGAATTGACCC
GTACAAGTTCTTTGACGACTGCAAGGAGAAGGTTAGTTGGTGCAATTCAACTTAGCTATATACCAGGTTGCGC
AAAGCTAACGTATAAATAATTTCGGTACTCTAGTATGGAGACATCTTCACATTCATACTGCTGGGCAAGAAGA
CGACTGTTTTTCTCGGTACAAAGGGAAATGATTTCATTTTGAACGGCAAGCTCAAGGATGTTTGCGCGGAGGA
TGTCTACTCCCCCCTCACCACCCCAGTGTTCGGACGACATGTGGTGTATGATTGCCCAAACTCCAAGCTCATG
GAGCAGAAGAAGGTGTGTACAGCAATTTATTCCCGTCTACATCTCCTGGGCCTCCATTGCTAATTTAATCGTC
TACCAGTTCGTCAAGTTCGGCCTCACCTCTGAAGCTCTCCGATCCTATGTCACCCTGATCACCAAGGAAGTTG
AGCAGTTCTTCGAGTCCTCCCCCGTCTTCAAGGGCGACTCCGGAGTTTTCAACGTCAGCAAGGTCATGGCTGA
AATCACCATCTACACCGCCTCTCGATCTCTACAGGGCAAGGAGGTGCGAGGAAAGTTCGATTCCAGCTTTGCG
GAACTCTACTCCGATCTCGACATGGGCTTCGCCGCCATCAACTTCATGTTCCCATGGTTCCCCTTCCCACACA
ACCGCAAGCGTGACCGTGCTCAAAAGAAGATGGCCCAGGTTTACACCGACATCATCCGTCAGCGACGTGAGGC
TGGTGGAGAGAAAGACTCCGAGGACATGGTATGGAACTTGATGTCGTCCGTGTACAAGAATGGAACGCCAATT
CCAGATATCGAAGTCGCCCACATGATGATTGCTCTTCTTATGGCTGGCCAGCACTCTTCTTCCTCCACCGGCT
CCTGGATCGTTCTCCGCCTTGCCAGCCGTCCAGATATTCTCGAGGAACTCTACGAGGAACAGAAACGTGTTCT
CGGCGAGGATCTTCCACCACTCACCTACGAATCTCTCCAGAAACTTGATCTTCACAACAATGTAATCAAGGAG
ACTCTCCGCCTCCACGCTCCCATCCACTCTATCCTCCGTGCTGTTAAATCCCCTATGCCCGTTGAAGGAACTA
ACTATGTTGTCCCAACCTCTCACAACCTCCTTGCCGCTCCTGGTGTTCCCTCACGAGACCCTCAGTACTTCCC
TGACCCTCTTGTTTGGAACCCTCACCGATGGGAGAACAACGTCGGTGTCACCGTAGTCGAGGCCAGTGAAGAA
AAGACAGATTACGGATACGGTTTGGTTAGCAAGGGTGCCAACAGCCCTTACCTCCCATTCGGCTCAGGCAGAC
ACAGATGCATCGGTGAACAATTTGCATATGTTCAGCTTGGAACCGTAACAGCTACGTTAGCCAGACTAATGAA
ATGGAAGCAAGTTGAAGGCACCAAAGATGTTGTGCCGCCAACTGACTATTCGGTAAGTTTGGCATATTACGCT
CCTTTTTATTTTGCTCGATTTTACTAACTTTGCATTAATAGTCCCTCTTCTCGAAGCCCCTTGGCGACCCAAT
GGTCTCGTGGGAGAAGCGAAAGCAGCCTTCCCAGAAA**TGA**TAATCACGACTGCGGATTGAATCATGTTAAATT
TCTTGCTTCTTGCTAATTTATTTCTTCTCTTTTCTTACGTTTATACACCGAGCCATATTTGCACTTTATGGTC
ACGCAAATAGGTTTTCGGAATAGAGCATATGTAATAAAAACTTTCTTTTGACCATGCTTTTGAAGATGGGGGA
TGTAGATAATAGTATTATTCTAATACAGCCATTTATTACAATACTCTAGATGAACAGGAAAGTTGCAGTTACT
CCGTTAGCTTTGGTGCAGAAACAAAATGGTAATATACATTGGAAATAGTACATCTTCCCGTAGCTGGGATAAT
CTAGCATCTAAACAGCGATATCCAAGCCAGTAATGTCAGGAAGTAACCAGCCAGCAACGTCGTTAATCCCGTG
CCCCCTTTCAACTCCGACGCTACAAAACGC**CTA**AGCAGCTGCCTGAGCATTCCTCCTCTCCGCCAACGCTCTC
TTCGCTCTGATCTCCTTCTGTCTTCTCTGACCACCGTCAATCTTTCTGCCACGCTTATGCTCCCACATGCCAT

CAGTAACCTGAACCTTGACGGCGGTACTCCATGTGTTTGGAATAATCTGAGCATACGGCCCCTTGATCATATT
CCCGTGGTGCAATTTATAATGGGCAAACTTTTTGAGTGGCTTGGAAGTATCCTTGAGCTCTGTTTCGCCTGGT
GGTAGATTGACAATCGCATAACGCATTCCAGGTGTAAGGGTAGCGGTGCCCATTGTGCTAACATCTAGGGACT
CTCCGTCGGTGGCATTGGTAGTTTTTGCACTCTTTGACTTTTCGACGGGGACCTCAACAGCTCTCAGCTCAGC
AACACCGTCAACAACGTTTTCGAAATCACCTCCTGGGCCGTAGTCCTGCTGTCTGAATTTCTCTCTCCACCGC
AGTATGTATCGTCTGTCCCTTGTCATTTCTATGCCCAATTCTCGAAGCTCAGCCGAGTCCGCTGTAAACAATT
GCTTCCATCCGGTGATTTTGCTGGAATATCTGGACATGTTTCGCCCAATTAGGCGTAGGAATGTTTCGACATC
GGGCACGAATGGGGTAGGTGATGGCACTGTTGCGGGTTTTCGAGATTGATGAAGGTACCGTACGCATGGCTGA
TAATGGGGGTTTGTGACGGAAGTGAAGAGCCGTAGTGGAAGGGATGTTCTGGCATTAAAGCCCGGTATGCGCG
CCGCAGC**CAT**AGTGGTTATATCGCCTTATATCAAACCGTTCAATGTCGAGATCTGGGGGGTGAAGATTTTAAT
TTAGCTCGTGGTGTAAGAACACCATCGGCGGGCGTGACGAAAGCGAAAAAAGCCACGGCACCAAGGGCCCAGG
TAAACCGGTAAGGGACTCAAGGCGAGTCAATCCAATGCTCAATGGCGCTGTATAGAAGATGTTCAACTGGTTC
TTCGACTTCGTTCAAGATTTTGCACCAAAATTTTGAATTAGGTCCGACGGCCTTAGCTCGGATCAACAAAACG
CCGATCTCCGGTGAGCTCGGGCCAGCCGAAGCTTCCATCGCAACATTCAAAGATGAAAGACGACGATATATAT
TTTGATAACAAACAAAGCTGTATTGTTGATTGAATACTAGGAGAGCTGCTCAGAGCAGTGTCTCACTCTTGTG
CCACTCTATCCTTTGTCTCGCGAAATTGGTGGAAGCCAATTTCGCTTGACATTATGGCAGTGTCGCCGCTTCC
CTCTCAATTCCCTCCCATCACTACTACCGGTGCTCCAAAGTCGTTAGCGATGAGAAATATTGCTTCTCAAGAG
CCTCGGACGGCGCCTAGTGGTGATCCTGCAGTTCCTAAGCCCTCCATGCCCGAACCAGAGCTGGCGGAGCAGT
TAAACGCTGATGTACGGCGGAAATATGTCAAAGGTGGGCGGGCAAATACACCCCAACCATTGGGACCCCATAA
TGCTAACATTTGGTTATTCTGAAAGATAAGAAGCTCGGTGAAGGTACATATGCAGTTGTTTACCTAGGCCATC
TACGAGATGATCCCACTTCGCTCGTTGCAATCAAGAAAATAAAACTAAACGCCGAGTACAAGGACGGGCTATC
CATGGACGCCATCCGTGAAGTAAAGTACCTTCAGGAACTGTCTCACCCGAACGTAATTGCGTTGCATGACGTT
TTCTCCTCAAAGGATCAAAATCTAAACCTCGTCCTTGAATTTTTACCCCTTGGAGATCTTGAAATGTTGATCA
AAGACAACTCTATCCAGTACGGTGTGGCTGATATAAAAGCATGGATAAGCATGCTTGCACGTGGAGTGTGGTT
CTGTCATAAGAATTTCATTCTACACCGTGATATTAAGCCTAACAACTTATTAATTGCATCGGATGGAGAAGTT
AAATTAGCGGATTTTGGTCTTGCCAGATCCTTTGCCGATCCATATCTCAACATGACACATCAAGTCATCACAA
GATGGTACAGGCCATTAGAACTACTCTTCGGCGCCAGGCAATATTCCGGTGCAGTCGACATATGGTCTATGGG
AATGGTGTTCGCTGAGCTTATTCTGCGCGTTCCATTCGCCGCTGGAAACACGGATATGGACCAAATATCTAAA
ATTTGTGCTGCATTTGGGGCACCTACGGAAGAAAACTGGCCTGGGGTGACAAAGCTGCCCAACTACGTCCCAA
TCGAGGAAAACCATGTCATACCCCTACAAGGCCGCGAATTCTTCCTCCGCCAATTTCCGACAGCTGGACCTTT
GGGGGCCGATCTACTTGCCTCCATGTTAAAACTTGATCCAAGGAAACGAATTACGGCCACTCAAGTCTTGCAG
CATCAGTGGTGGCTTGCTGAGCCTAAACCAACTAAGAACGAGGACCTGCCTAGGAAATCAGGAGGAGAAAAGA
AAATGGGCAACGATATGGGCAGACGAGTTGGTGAGATTGACGAGTCAAAATTTAAAGGAGTGTCCCGTCAACT
TGATTTCGGAGCTATGAAGAAATGAGAGGATCCGACTTGCCATCCGGAATATAATACTCAAAAATTCTCGCCT
CCTTGGTTAGCAAGCGAGCAGATATCGCGTACCGACAAATATATGCTACAAGAAGAAGTGATACACAACATTC
TGGGCCGCTCCTCTTTGTGGGTGCAATGCTGTGTAAACATCGGCCTCAATGAGAGCCCAATTTGGTTAATGAG
CTGCAGCCTTACCAATACGGAGGAGACGTCAGTAGC
