# Supplementary material for: Two different types of tandem sequences mediate the overexpression of TinCYP51B in azole-resistant Trichophyton indotineae
Source: Antimicrob Agents Chemother. 2023 Oct 12;67(11):e00933-23. doi: 10.1128/aac.00933-23 (PMC10648874; doi:10.1128/aac.00933-23)
Supplement: Supplementary file 4 [file aac.00933-23-s0004.docx]

TIMM20115 (**UKJ1700/17II**; IFM67093)	8.0	F397L	0.06	0.03
**UKJ 1701/17**	8.0	F397L	0.06	0.03
**UKJ 1703/17**	8.0	F397L	0.125	0.03
**UKJ 1706/17#**	8.0	F397L	0.06	0.03
**UKJ 1746/17**	16.0	L393F	0.125	0.062
**200083/18#**	0.125	-	0.25	0.0625
**250063/18#**	8.0	F397L A448T	0.125	0.125
**250074/18**	0.125	A448T	0.125	0.125
Strains deemed to be resistant to azole compounds (ITC MIC_{80} ≥ 0.25 µg/mL; VRC MIC_{80}				
TIMM20116 (**UKJ1708/17**; IFM 67094)	0.125	A448T	0.5	0.5
TIMM20118 (**UKJ1687/17**; IFM 67096)	8.0	F397L	0.5	1.0
TIMM20119 (**200123/18**; IFM 67097)	8.0	F397L	1.0	1.0
**UKJ 1729/17***	0.125	A448T	0.25	0.5
**UKJ 1729/17II***	0.125	A448T	0.5	1
**200078/18***	0.125	A448T	0.25	0.25
**200086/18***	0.125	A448T	0.25	0. 5
TIMM20117 (**200087/18**; IFM 67095)*****	0.125	A448T	0.5	0.5
**200099/18***	0.125	A448T	0.25	0. 5
**200104/18***	0.125	A448T	0.25	0. 5
**200126/18***	0.125	A448T	0.25	0. 25
**200127/18**	0.125	A448T	0.25	0. 5
**200128/18***	0.125	A448T	0.25	0. 25
**200129/18***	8.0	F397L	0.125	0. 25
**216510/17***	8.0	F397L	0.5	1.0
**250064/18**	8.0	F397L	0.25	0.5
**250066/18**	8.0	F397L A448T	0.25	0.25
**250075/18**	0.125	A448T	0.25	0.25
TIMM20120 (**250082/18**)*****	0.125	A448T	0.25	1.0
TIMM20121 (**250084/18**)*****	8.0	F397L A448T	0.5	1.0
**250092/18**	1.0	Q408L A448T	0.5	1.0
**250097/18**	0.125	A448T	0.25	0.25
**250105/18**	8.0	F397L A448T	0.5	1.0
TIMM20122 (**250108/18**)*****	8.0	F397L A448T	0.5	0.5
**250136/18 (UKJ1392/18)**	8.0	F397L A448T	0.25	0.5
**600047/19**	8.0	F397L	0.5	0.25
**600061/19**	8.0	F397L A448T	0.25	0.5
**600084/19**	0.125	A448T	0.5	0.5
TIMM20123 (**600097/19**)*****	0.125	A448T	1.0	0.5
**600108/19**	0.125	A448T	0.5	0.5
**600125/19**	0.125	A448T	> 1.0	0.5
**600128/19**	0.125	F397L A448T	1.0	0.5

[a]All strains were from a previously published resistance study in India, with the numbering in bold (
were then preserved in the TIMM culture collection. Some of the strains preserved in the TIMM cu
also preserved in the IFM culture collection, through the National Bio-Resource Project, Japan (http
*: PCR results were shown in Fig. 1A (lanes 6 to 20).
#: PCR results were shown in Fig. 1A (lanes 21 to 29).
