# Supplementary material for: Two different types of tandem sequences mediate the overexpression of TinCYP51B in azole-resistant Trichophyton indotineae
Source: Antimicrob Agents Chemother. 2023 Oct 12;67(11):e00933-23. doi: 10.1128/aac.00933-23 (PMC10648874; doi:10.1128/aac.00933-23)
Supplement: Supplementary file 5 [file aac.00933-23-s0005.docx]

TABLE S2 Oligonucleotide primers used in this study

Primer name	Sequence (5'-3')	Chain	Purpose	Refere
P1	GCAAGTTGAAGGCACCAAAG	Sense	Rapid PCR strain typing of azole resistant *T. indotineae*	This st
P2	CCGGAGGCTTTGTAGGGTCC	Anti-sense	Rapid PCR strain typing of azole resistant *T. indotineae*	This st
P3	CAAATACACCCCAACCATTGGG	Sense	Rapid PCR strain typing of azole resistant *T. indotineae*	This st
P4	GACCGAACAGTGTCGCTGTCG	Anti-sense	Rapid PCR strain typing of azole resistant *T. indotineae*	This st
P5	AAGTTCGTCAAGTTCGGCCT	Sense	qPCR (*TinCYP51B*)	12
P6	GAGAGGCGGTGTAGATGGTG	Anti-sense	qPCR (*TinCYP51B*)	12
P7	TGTTGGTGA TGAGGCACAGT	Sense	qPCR (*TinACTIN*)	12
P8	CCATGTCATCCCAGTTTGTG	Anti-sense	qPCR (*TinACTIN*)	12
P9	GCGTGACCCAGCCAACA	Sense	qPCR (*TinGAPDH*)	12
P10	TTGGCACCTCCCTTCAAGT	Anti-sense	qPCR (*TinGAPDH*)	12
P11	GATCTTACCTGCCATCGACTTGGAGTTGAAGTTG	Sense	Amplification of the 5' UTR and ORF of *TinCYP51B* gene	This st
P12	ACGAGAGACgATaTCtGCTAAGAGGCCCATGGTGA	Anti-sense	Amplification of the 5' UTR and ORF of *TinCYP51B* gene<br>Inactivation of the Cas9/gRNA1 RNP cleavage site by overlap extension PCR	This st
P13	TAGCAgATaTcGTCTCTCGTTTCTGCGAGAACTGC	Sense	Amplification of the 5' UTR and ORF of *TinCYP51B* gene<br>Inactivation of the Cas9/gRNA1 RNP cleavage site by overlap extension PCR	This st
P14	TTATCAGCCATGCGTACcGTACCTTCATCAATCTC	Anti-sense	Amplification of the 5' UTR and ORF of *TinCYP51B* gene	This st
P15	CATG*actAGT*GCGGTATGCCTTTACCCATTGCATA	Sense	Amplification of the 5' UTR and ORF of *TinCYP51B* gene by nested PCR for pAg1-*TinCYP51B*/R	This st
P16	ATTA*gggccc*TCATTTCTGGGAAGGCTGCTTTCGCTTCTCCCAactcACCATTGGGTCGCCAAG	Anti-sense	Amplification of the 5' UTR and ORF of *TinCYP51B* gene by nested PCR for pAg1-*TinCYP51B*/R<br>Inactivation of the Cas9/gRNA2 RNP cleavage site<br>Amplification of the *TinCYP51B*-specific hybridization probe	This st
P17	CCAG*ggATcc*TAATCACGACTGCGGATTGAATCAT	Sense	Amplification of the 3' UTR of *TinCYP51B* gene for pAg1-*TinCYP51B*/R	This st
P18	CCCA*ggTACC*ATATGTCGACTGCACCGGAATATTG	Anti-sense	Amplification of the 3' UTR of *TinCYP51B* gene for pAg1-*TinCYP51B*/R	This st
P19	TTT*ctcgag*ATGGGCCTCTTAGCCGACATTGTCTC	Sense	Amplification of the *TinCYP51B*-specific hybridization probe	This st
P20	TTT*aagctt*CTTGCAGTCGTCAAAGAACTTGTACG	Anti-sense	Amplification of the *TinCYP51B*-specific hybridization probe	This st
P21	TTT*ctcgag*CCTCACCGATGGGAGAACAACGTCGG	Sense	Amplification of the *TinCYP51B*-specific hybridization probe	This st
P22	TTCCCTGACCCTCTTGTTTGGAACCCTCACCGATGG	Sense	Screening of desired transformants by PCR<br>Detection of the type I (2404 bp) duplicated blocks within the genome of transformants	This st
P23	CTTGAACAGGAGCTGCTGCAGGACGTTGATAACAA	Anti-sense	Screening of desired transformants by PCR<br>Detection of the type I (2404 bp) duplicated blocks within the genome of transformants	This st
P24	GAGCTGCTCAGAGCAGTGTCTCACTCTTGTGCCAC	Sense	Screening of desired transformants by PCR<br>Detection of the type II (7374 bp) duplicated blocks within the genome of transformants	This st
P25	GCAACTTCAACTCCAAGTCGATGGCAGGCAAGATC	Anti-sense	Screening of desired transformants by PCR<br>Detection of the type II (7374 bp) duplicated blocks within the genome of transformants	This st
